# Metabolome and transcriptome analyses identify the characteristics and expression of related saponins of the three genealogical plants of bead ginseng

**DOI:** 10.7717/peerj.16034

**Published:** 2023-09-01

**Authors:** Yihan Ye, Nan Ma, Yidan Peng, Ying Chen, Yuqu Zhang, Shuyan Zhao, Wei Ren, Yonggang Yan, Gang Zhang, Xinjie Yang, Xiujuan Peng

**Affiliations:** 1 School of Pharmacy, Shaanxi University of Chinese Medicine, Xianyang, Shaanxi, China; 2 Shaanxi Qinling Application Development and Engineering Center of Chinese Herbal Medicine, Xianyang, Shaanxi, China; 3 Shaanxi Institute of International Trade & Commerce, Xianyang, Shaanxi, China; 4 School of Pharmacy, Health Science Center, Xi’an Jiaotong University, Xi’an, Shaanxi, China

**Keywords:** Metabolome, Transcriptome, Saponins, *Panax japonicas*, *Panax pseudoginseng*, *Panax pseudo-ginseng* var. *elegantior*

## Abstract

**Objective:**

The classification and clinical usage of the different species of bead ginseng are often confused. Therefore, we conducted an integrated metabolomics and transcriptome analysis of three main species of Panax, including *Panax japonicas*, *Panax pseudoginseng*, and *Panax pseudo-ginseng* var. *elegantior*.

**Methods:**

A broad metabolome and transcriptome analysis for three origins of bead ginseng plants was performed using UPLC-ESI-MS/MS, RNA sequencing and annotation, and bioinformatic analysis of transcriptome data.

**Results:**

The levels of 830 metabolites were determined. A total of 291 differentially accumulated metabolites (DAMs) between *Panax pseudo-ginseng* var. *elegantior* and *Panax japonicas* (Group A), with 73 upregulated and 218 downregulated. A total of 331 DAMs (110 upregulated and 221 downregulated) were found between *Panax pseudoginseng* and *Panax japonicas* (group B). There were 160 DAMs (102 up-regulated and 58 down-regulated) between *Panax pseudoginseng* and *Panax pseudo-ginseng* var. *elegantior* (group C). In addition, RNA sequencing was performed in the above three ways. A total of 16,074 differential expression genes (DEGs) were detected between Group A, in which 7,723 genes were upregulated and 8,351 genes were downregulated by RNA sequencing. Similarly, 15,705 genes were differentially expressed between group B, in which 7,436 genes were upregulated and 8,269 genes were downregulated. However, only 1,294 genes were differentially expressed between group C, in which 531 genes were upregulated and 763 genes were downregulated. We performed differential gene analysis on three groups of samples according to the Venn diagram and found that 181 differential genes were present. A total of 3,698 and 2,834 unique genes were in groups A and B, while 130 unique genes were in group C.

**Conclusions:**

This study provides metabolome and transcriptome information for three bead ginseng plants. The analysis of the metabolite content showed differences in the attributes of the three bead ginseng, contained mainly flavonoids, phenolic acids as well as terpenes.

## Introduction

**Figure 1:**
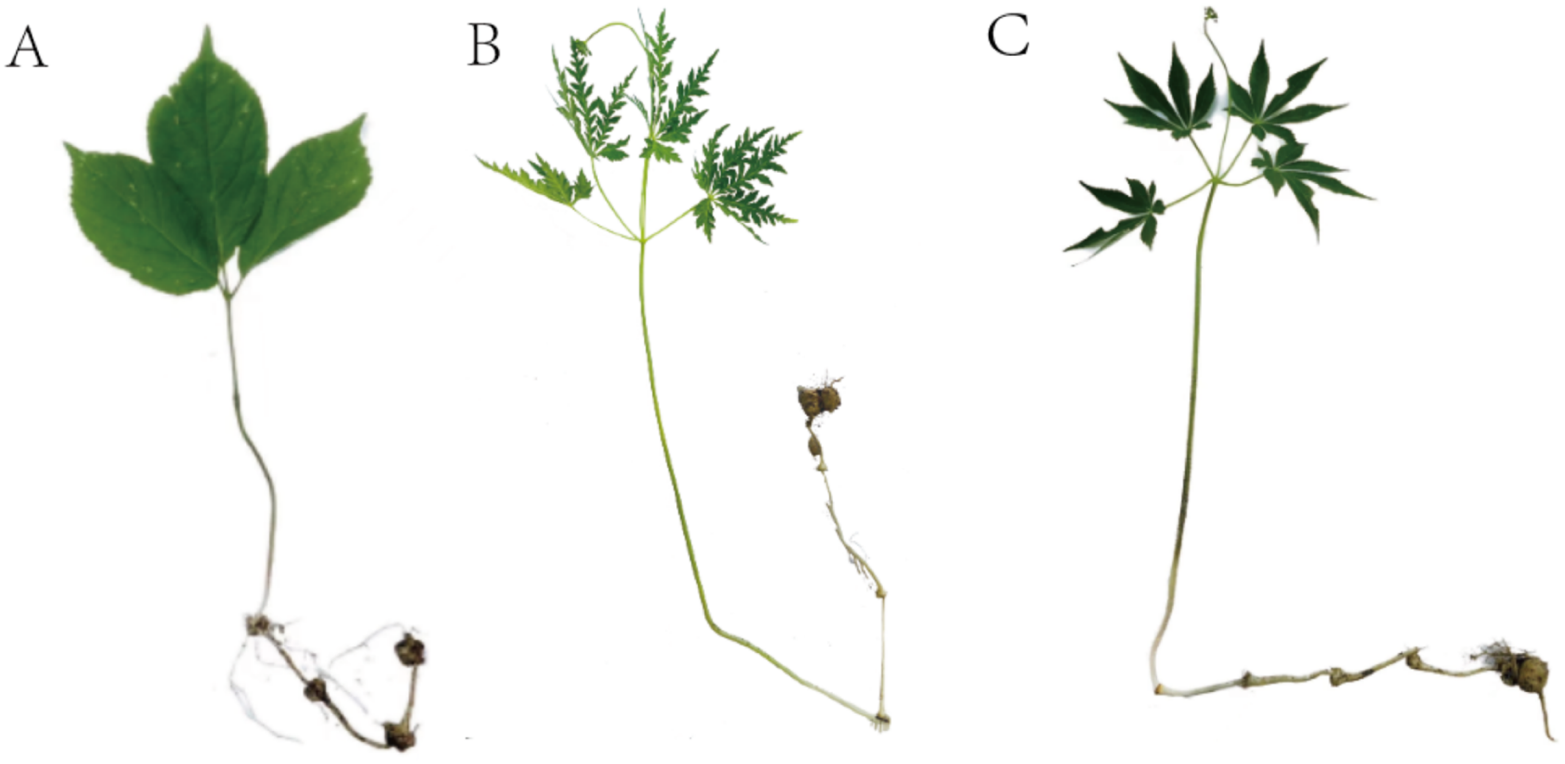
Phenotypical analyses of the plant materials. (A) *Panax japonicas* (B) *Panax pseudoginseng* (C) *Panax pseudo-ginseng* var. *elegantior*.

*Panax Linn* is a rare medicinal plant genus with a history of at least 4,000 years and is widely grown in America, Asia, and Europe. It originally comes from the Himalayan mountains and is mainly distributed in the high mountains of East Asia and North America (Wen & Zimmer, 1996). The different *Panax* (*P*) species have different medicinal functions due to significant differences in the active components. Panacis Majoris Rhizoma (PMR) is the dry rhizome of *Panax japonicus* and *Panax pseudoginseng*. In addition, the dry rhizome of *Panax pseudoginseng* Wall. var. *elegantior* (Burkill) Hoo & Tseng is often used as bead ginseng in folk medicine (Yi, Ren & Liang, 2011). Thus, in China, *Panax japonicus* (DY, Fig. 1A), *Panax pseudoginseng* (YY, Fig. 1B), and *Panax pseudoginseng* var. *elegantior* (XL, Fig. 1C) are used as the same Chinese medicine, bead ginseng.

The resources of bead ginseng are rather limited despite their high medicinal value and long growth cycle. With disorderly mining and overutilization in modern times, the ecosystem of bead ginseng and other P medicinal materials has been seriously damaged, and the output has also been sharply reduced. As a result, it is more precious and has been listed as a rare and endangered species by the state (Chinese Pharmacopoeia Commission, 2020; Wen & Zimmer, 1996). Therefore, accurately identifying bead ginseng is of great significance for the protection, planting and rational utilization of rare *P* medicinal materials.

Previous studies have examined the constituents and pharmacological effects of bead ginseng, showing that terpenes, specifically terpenoid saponin, are the primary chemical compounds found in this plant (Zhang, Luo & Wang, 2019). Its total saponins have pharmacological effects such as hepatoprotection, sedation, analgesia, anti-inflammatory, antitumor, and immune enhancement (Hai-yuan, Xiao-hui & Shuang-xi, 2017; Ying, Yang & Wei, 2015; Jiahong, Haibo & Mengqiong, 2011; Miaomiao, Xuan & Bei, 2014; Ying, Yang & Wei, 2015). For instance, the total saponins of bead ginseng can increase the ATPase activity of damaged brain tissue to delay the breakdown of ATP, thereby enhancing brain energy metabolism and promoting brain energy breakdown (Yi, Yuping & Xiaomei, 2008).

The triterpene saponins from bead ginseng belong to the terpenoid compounds since they are mainly synthesized *via* the methylglutaric acid pathway. It is currently believed in the plant community that there are only two synthetic pathways for terpenoid compounds in plants: the 2-C-methyl-D-erythritol 4-phosphate (MEP)-route in the cytoplasm and the mevalonate (MVA) pathways (Tao & Wei, 2010). The saponins of bead ginseng are mainly triterpenoid saponins, and the MVA pathway is the main pathway for their synthesis (Tian, Zhu & Yao, 2017). In recent years, the synthetic pathway of saponins has been continuously researched. Due to these studies, the upstream and midstream of the saponin biosynthetic pathway in *P* plants have been fundamentally studied, but the downstream is still being gradually uncovered (Zhao-bin, Yue-hong & Shan, 2012). However, the different distribution and biosynthesis of active ingredients in bead ginseng are largely unknown.

In China, the morphological and distribution similarities of these three plants are often confused and misused. Currently, there is relatively little research on bead ginseng. It is unknown whether *Panax pseudo-ginseng* var. *elegantior* can be used as a substitute or expanded source of ginseng beads. Additionally, the content of its main active ingredients and the composition of the overall compounds in these three medicinal plants have not been reported. This study aims to use metabolomics and transcriptomics to elucidate the differential expression of metabolites and genes in these three medicinal plants.

## Materials and Methods

### Plant materials

The rhizomes of DY, YY and XL were collected from Red River Valley Forest Park, Shaanxi Province (N34°16′48″, E107°45′), at an altitude of approximately 1,500 m above sea level, in Shaanxi Baoji, China. The majority of precipitation falls between July and September in this region (Zhiqing, Xuexiang & Dongming, 2011). The specimens were gathered in July, the month in which bead ginseng develops most vigorously. For this study, three groups of samples were selected, and each group consisted of three biological replicates. All samples were stored at −80 °C in an ultra-low temperature refrigerator and frozen in liquid nitrogen. The identification of the three different basal plants of bead ginseng was performed by Xinjie Yang from Shaanxi University of Chinese Medicine, and the sample was deposited at Shaanxi University of Chinese Medicine in Xianyang City, Shaanxi Province.

### Extensively targeted metabolomic analysis with UPLC-ESI-MS/MS

After grinding, 100 mg of the powder was combined with 1.2 mL of a 70% methanol solution, vortexed for 30 s every 30 min for 3 h, and then kept overnight at 4 °C in the refrigerator. The supernatant was filtered using a 0.22 μm membrane (Anpel, Shanghai, China) after 10 min of centrifugation at 12,000 rpm. The UPLC (Ultra-Performance Liquid Chromatography) system (Shimadzu Nexera X2; Shimadzu, Kyoto, Japan) utilized an Agilent SB-C18 column (1.8 μm, 2.1 mm × 100 mm) and a mobile phase consisting of solvents A (pure water with 0.1% formic acid) and B (acetonitrile with 0.1% formic acid) with a gradient algorithm for sample measurements (Cao et al., 2022). The flow rate was a 0.35 mL/min. The AB4500 triple quadrupole-linear ion trap mass spectrometer (Applied Biosystems, Waltham, MA, USA) equipped with an ESI Turbo Ion-Spray interface was used for measurements with a linear ion trap (LIT) and triple quadrupole scans. High collision-activated dissociation (refers to the fragmentation that is set in the collision cell to produce ion fragments with specific properties for further screening and analysis of target molecules) was used at the source temperature of 550 °C. In positive and negative ion modes, the ion spray voltages were 5,500 V and 4,500 V, respectively. The ion source gases I (GSI), II (GSII), and curtain gas (CUR) were set to pressure settings of 50, 60, and 25 psi. High collision-activated dissociation (CAD) gas levels were used. The instrument calibration and mass calibration were performed using solutions of 10 and 100 μmol/L polypropylene glycol in the QQQ (Triple Quadrupole Mass Spectrometry) and LIT (Linear Ion Trap) modes, respectively. In MRM studies, the collision gas (nitrogen) was set to medium when acquiring QQQ scans DP and CE optimization were used to calculate the declustering potential (DP) and collision energy (CE) for individual MRM transitions. A specific set of MRM transitions was monitored for each period according to the metabolites eluted within this period (Li et al., 2021). The high-performance liquid chromatography (HPLC) effluent was coupled alternatively to electrospray ionization (ESI)-QQQ-LIT-MS/MS (electrospray ionization-triple quadrupole-linear ion trap-mass spectrometry/mass spectrometry) system.

### RNA sequencing and annotation

To prepare for sequencing, RNA was extracted from three biological replicates of DY, YY and XL. Total RNA was extracted from three plant tissue samples, and agarose gel electrophoresis (concentration, 1%; voltage, 180 V) was used to analyze the integrity of the RNA and to determine whether there was DNA contamination. The purity, concentration, and integrity of the RNA were evaluated using the NanoPhotometer spectrophotometer (Immplen, Westlake Village, CA, USA), Qubit 2.0 Fluorometer (Life Technologies, Carlsbad, CA, USA), and Agilent 2100 Bioanalyzer (Agilent Technologies, Santa Clara, CA, USA), respectively. Second-strand cDNA (complementary DNA) was created using DNA polymerase, RNase, and dNTPs. After end-repair, A-tailing, and indexing ligation, purification was carried out using AMPure XP (Beckman Coulter, Inc., Brea, CA, USA), and cDNA libraries were created for further amplification. The insert size of the cDNA libraries was measured using the Agilent 2100 Bioanalyzer after dilution to 1.5 ng/µL. RNA sequencing was performed on the Illumina HiSeq platform (Illumina, San Diego, CA, USA). The statistical power of this experimental design was calculated as follows: RNASeqPower of YY_vs_XL is 0.251398, YY_vs_DY is 0.217893, and XL_vs_DY is 0.232748.

### Transcriptome data analysis using bioinformatics

The genes were annotated based on the SwissProt, KEGG (Kyoto Encyclopedia of Genes and Genomes) and GO (Gene Ontology) databases using various tools, including Blast2GO (Götz et al., 2008), Diamond (Buchfink, Xie & Huson, 2015), WGCNA (Langfelder & Horvath, 2008), KAAS (Moriya et al., 2007), HMMscan (Eddy, 2011), and BLAST+(Camacho et al., 2009). The prediction of transcription factors was carried out with iTAK (Zheng et al., 2016), and gene expression was quantified using FPKM (Trapnell et al., 2010) and RSEM software(Li & Dewey, 2011). Differential analysis of the genes was performed using edgeR (Robinson, McCarthy & Smyth, 2009) and a negative binomial generalized log-linear model. The following conditions were used to identify DEGs: log2FC>1 or <−1 and FDR values were <0.05.

## Results

### Comparison of metabolites produced by three originals of bead ginseng

**Figure 2:**
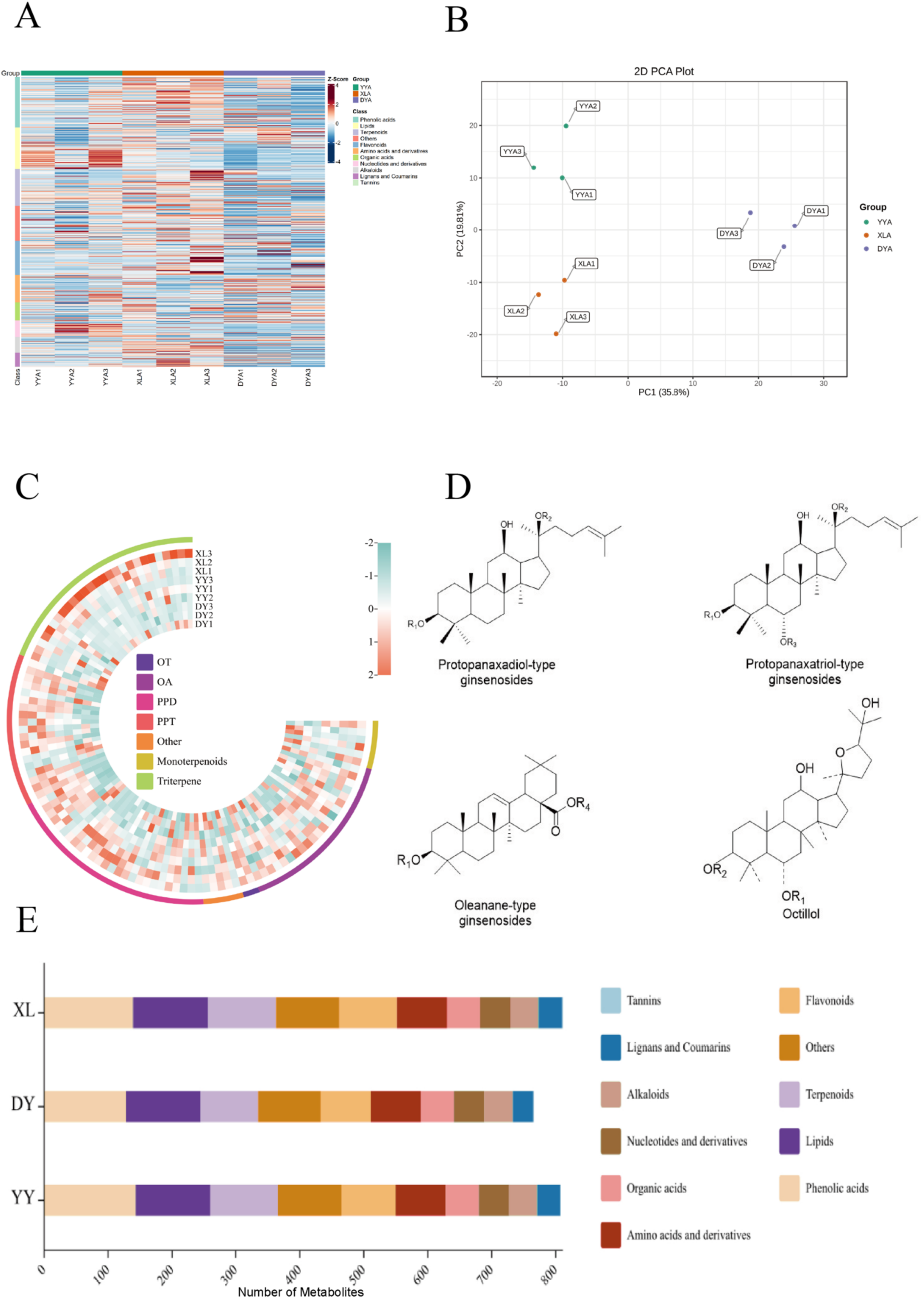
Comparison of metabolites produced by three originals of YY, XL, and DY. For each sample, three biological replicates are denoted by numbers 1, 2, and 3. (A) Clustering heatmap of all metabolites. Each sample is represented by a column and each metabolite is represented by a row. The abundance of each metabolite is represented by a bar with a specific color. The upregulated and downregulated metabolites are indicated by different shades of red and green, respectively. (B) PCA score plot. (C) Clustering heat map of all terpenoid metabolites. (D) Four different terpenoid structures. (E) Distribution of metabolites in different plants.

The UPLC-ESI-MS/MS system was used for broad metabolites analysis to identify and understand the metabolites produced by three original samples. A total of 830 metabolites were quantified, including 145 phenolic acids, 118 lipids, 106 terpenoids, 98 flavonoids, 78 amino acids and derivatives, 52 organic acids, 47 nucleotides and derivatives, 45 alkaloids, 40 lignans, one tannic acid and 100 other metabolites (Table S1). The biological replicates of the three plants clustered together in different regions, as revealed by the PCA analysis findings (Fig. 2B). The metabolomics findings were reliable and repeatable, as indicated by the overlapping TIC of the QC mixtures (Fig. S2) and the OPLS-DA (Worley & Powers, 2016) (Fig. S3). A total of 808, 811 and 765 metabolites were detected in cultivars YY, XL and DY, respectively. Many metabolites were found in all three originals, totaling 748. Similar ratios of each metabolite class were found in all cultivars, with the main metabolites being phenolic acids, lipids, flavonoids, and terpenoids (Fig. 2C). The heatmap revealed that there were lots of flavonoids in the XL. While YY was rich in lipids, nucleotides and derivatives compared to the other original. Meanwhile, XL was rich in phenolic acids, flavonoids and terpenoids (Fig. 2A).

Our findings demonstrated that, while the qualitative makeup of the samples are almost the same as that of the original bead ginseng, there are changes in the representation of particular components in the metabolomic profiles of the samples. Z-score normalization procedures were used to standardize the data, and hierarchical grouping of metabolite abundance data and visualization with heatmaps were performed. Each row in the heatmaps corresponded to a metabolite, and each column to a sample. The colors represented the relative abundances of the metabolites, with red denoting metabolite abundances above the mean and blue representing metabolite abundances below the mean. The XL samples grouped together with YY samples, and relatively small differences were observed between these two samples.

Terpenoids are the active ingredient of bead ginseng. Thus, we then focused on terpenoids. A total of 106 terpenoids have been identified in three species. As shown in Fig. 2C, 69% of the terpenoids were triterpene saponin, including 26 protopanaxadiol (PPD), 19 protopanaxatriol (PPT), 21 oleanolic acids (OA), two octillol (OT), and five other hybrid subtypes (Fig. 2D). The results showed that all three species met the stated criteria and the differences between the results presented here were extremely negligible.

### Identification of metabolites responsible for differences among three original plants

**Figure 3:**
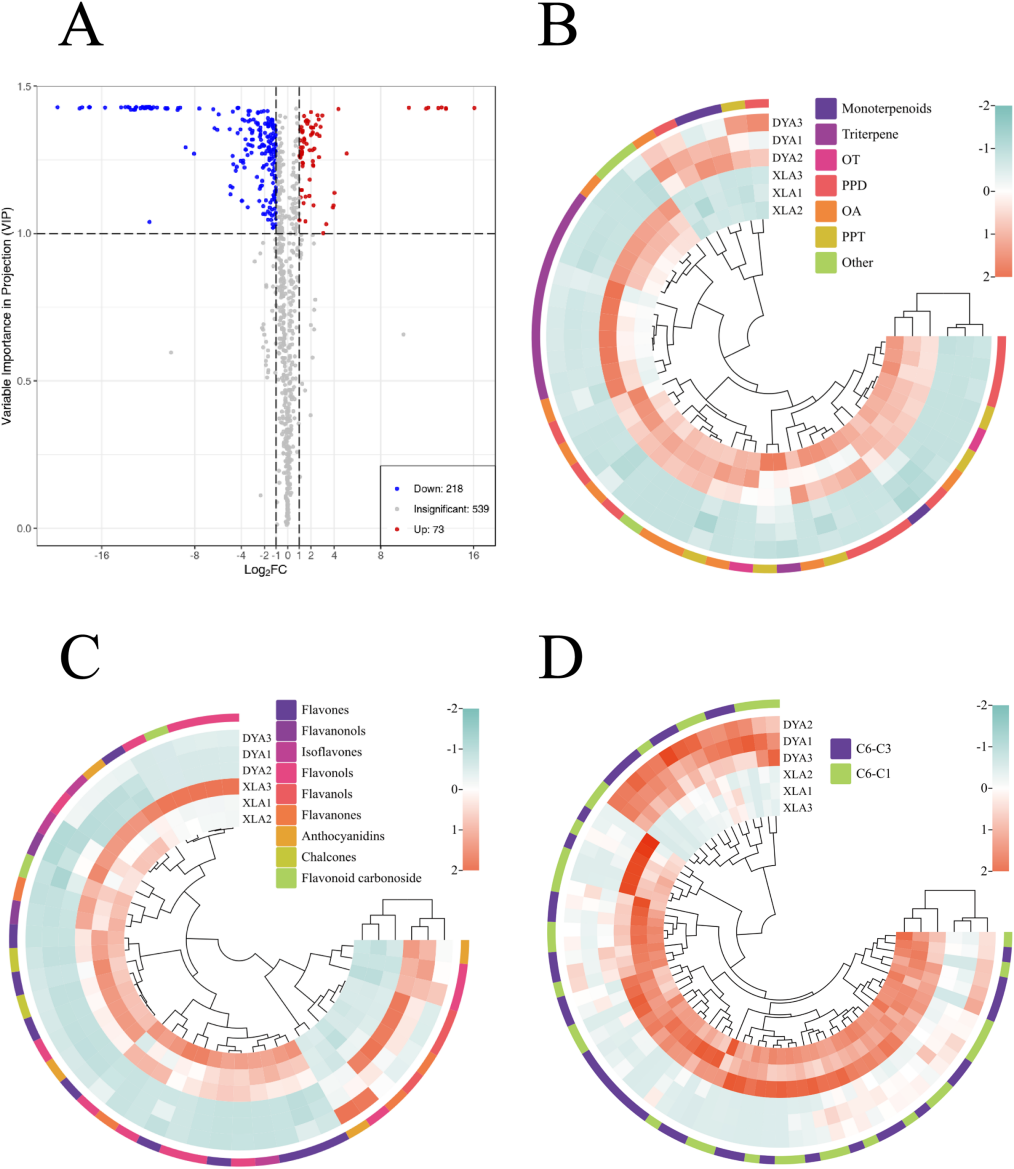
Group A distribution of differentially accumulated metabolites (DAMs) in the broad metabolomes. (A) Volcano plots of differentially accumulated metabolites (DAMs). (B) Differentially accumulated terpenes in the commonly targeted metabolome. (C) Flavonoids differentially accumulated in the metabolome, which is commonly targeted. (D) Differently accumulated phenolic acids in the broad metabolome.

**Figure 4:**
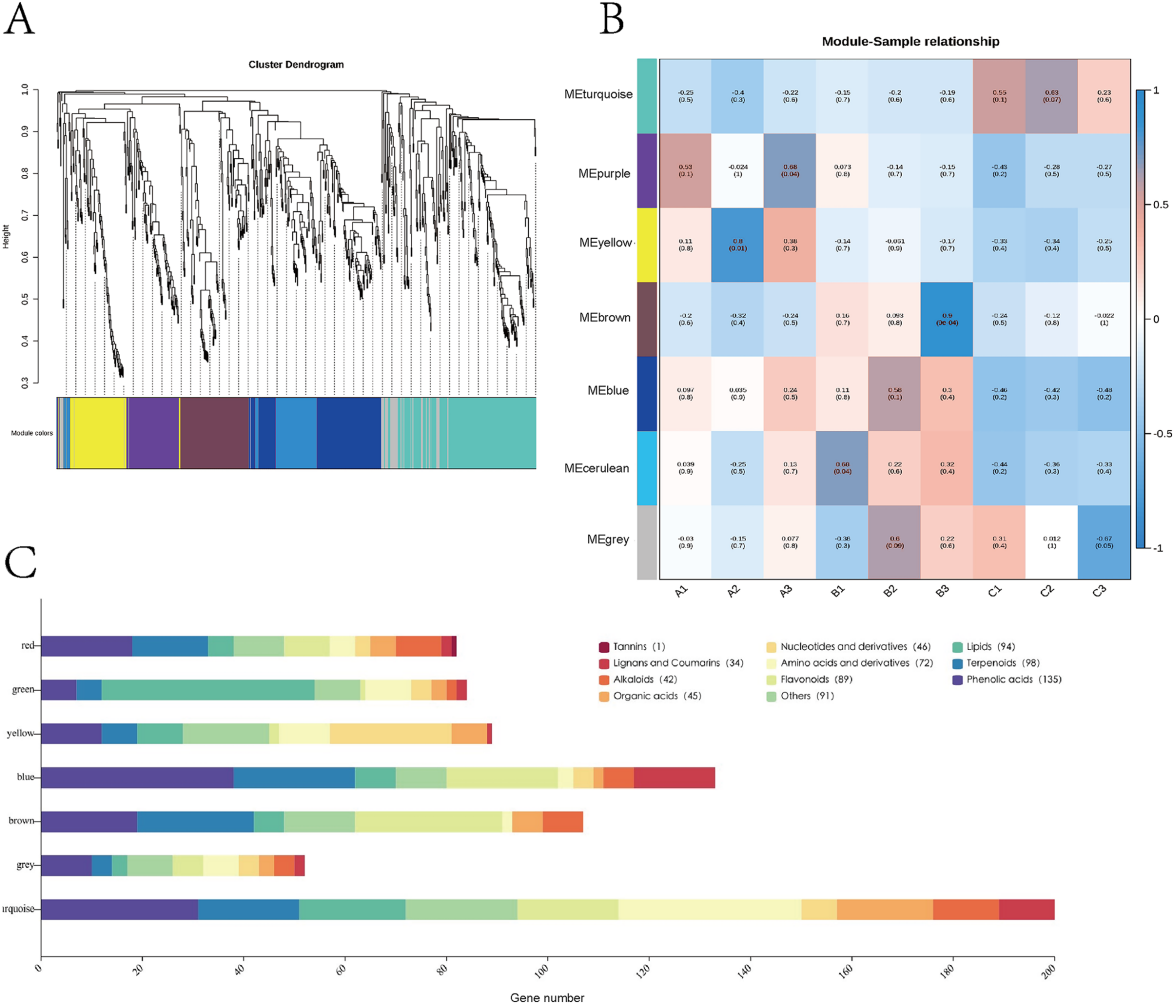
Correlation of metabolites with hemp seed varieties based on WGCNA. (A) Clustering dendogram of average network neighborhood to identify metabolite coexpression modules. Clustering dendogram of metabolites, with dissimilarity based on topological overlap, along with assigned module colors. (B) Module-variety associations. Each row corresponds to a module eigengene, each column to a variety. Each cell contains the corresponding correlation and value of *p*. The table is color-coded by correlation according to the color legend. (C) Distribution of different types of metabolites in six modules.

Based on pairwise comparison between the three plants and VIP values of the OPLS-DA model ≥1 and the |log_2_(fold change)| ≥1, screening results were presented using Venn diagrams (Fig. 3A) and Volcano plots (Figs. 3B–3D). A total of 291 differentially accumulated metabolites (DAMs) were found between XL and DY (group A), with 73 upregulated and 218 downregulated (Fig. 3B). It was found that 331 metabolites were differentially produced (110 upregulated and 221 downregulated) between YY and DY (group B) (Fig. 3C). There were 160 different metabolites (102 upregulated and 58 downregulated) between YY and XL (group C) (Fig. 3D). The three major subcategories of DAMs were phenolic acids, terpenoids, and lipids. The number of phenolic acids that accumulated upward and downward in group A was 14 and 5, respectively. Meanwhile, of the variably accumulated phenolic acids, C6-C1 and C6-C3 kinds made up 47.89% and 52.11%, respectively (Fig. 4A). A total of 46 terpenoid DAMs were identified, with six highly accumulated cases containing ginsenoside Rf, phanoside, chikusetsusaponin LT8, cankanoside A, 2-hydroxy-3-[(2-O-beta-D-glucopyranosyl-beta-D-glucopyranosyl). oxy]-20-[(6-O-alpha-L-rhamnopyranosyl-beta-D-glucopyranosyl) oxy] dammar-24-en-12-one, and genipin-1-O-(2-O-apiosyl) glucoside. The remaining differential metabolites were down-accumulated (Fig. 4B). There were 44 different flavonoid metabolites belonging to nine structural subtypes, among which the flavonols (10) made up the most. The number of phenolic acids that accumulated up and down was 10 and 34, respectively (Fig. 4C). The results clearly showed that XL was the most abundant species in phenolic acids, flavonoids, and terpenes, followed by YY and finally DY.

The Venn diagram shows that a total of 441 differential metabolites were identified in at least one pairwise comparison. Among these, 38 differential metabolites were common to the three groups. These metabolites, considered to be characteristic metabolites, included 15 flavonoids, six nucleotides and derivatives, five terpenoids, three amino acids and derivatives, three phenolic acids, 3 lignans and coumarins, and 3 others (Table S2). These contained 25 metabolites, the observed YY was higher than XL and DY, of which five were flavonoids, five were terpenoids, three were lignans and coumarins and two were phenolic acids. Of these 38 metabolites, six were more common than YY and DY, two phenolic acids, two terpenoids, one amino acid, and derivatives one flavonoid. There were four metabolites more common in XL and YY, including L-ornithine (amino acids and derivatives), darendoside A (phenolic acids), eriodictyol-3-O-glucoside (flavonoids), and grevilloside M (phenolic acids). These findings indicated that the levels of various metabolites in XL and YY were significantly higher than those in DY. These results also show that the most abundant species in marker metabolites was XL, followed by YY and DY.

### KEGG pathway enrichment analysis of differentially expressed metabolites

Pathway analyses were performed based on the KEGG database for the differentially expressed metabolites. Out of all the substances, 242 have KEGG pathway annotations. Among the metabolites in group A, 67 substances were annotated with KEGG pathways (39 down-regulated and 28 up-regulated), with metabolic pathway, secondary metabolite biosynthesis, ABC transporter, amino acid biosynthesis, and aminoacyl-tRNA biosynthesis being the top five pathways with the highest accumulation. In group B, 65 KEGG pathways were annotated among the significantly different metabolites (28 down-regulated and 37 up-regulated), with metabolic pathway, secondary metabolite biosynthesis, ABC transporter, amino acid biosynthesis, and aminoacyl-tRNA biosynthesis being the top five pathways with the highest accumulation. Both groups showed significant enrichment in metabolic pathways, secondary metabolite biosynthesis, ABC transporters, amino acid biosynthesis, and aminoacyl-tRNA biosynthesis. However, group A had a higher proportion of down-regulated sequences compared to group B, which had more up-regulated sequences. Additionally, group B had a broader range of signaling pathways compared to group A. Therefore, while both groups have common pathways, they also have unique differences that suggest distinct biological processes may be occurring in each group. Group C had 51 substances with KEGG pathway annotations (24 down-regulated and 27 up-regulated). The top five pathways with the highest accumulation include metabolic pathways, secondary metabolite biosynthesis, ABC transporters, galactose metabolism, and purine metabolism.

### Identification of modules of closely related metabolites

Weighted gene co-expression network analysis (WGCNA) can identify metabolomes that undergo highly synergistic alterations. WGCNA can focus not only on various metabolites but also on identifying metabolite groups of interest from data containing thousands or even tens of thousands of metabolites, for substantial correlation analysis with phenotype based on the most variable metabolites or all metabolites. Therefore, WGCNA was carried out, and a network was established to study the relationship between metabolites and the origin of Astragalus. Metabolites were divided into six modules (consisting of 52–200 metabolites), and metabolites that did not belong to these modules were marked in gray (Figs. 4A and 4B). Figure 4C shows the composition of the six modules of metabolites. Details of the metabolites in each module can be found in Table S4. For each module, intrinsic metabolites can be calculated, describing metabolite accumulation profiles within the module. Additionally, each co-expression module was correlated with the seed origin of rhubarb kernel by Pearson correlation coefficient analysis (Fig. 3B).

### Identification of metabolite characteristics in different varieties

**Figure 5:**
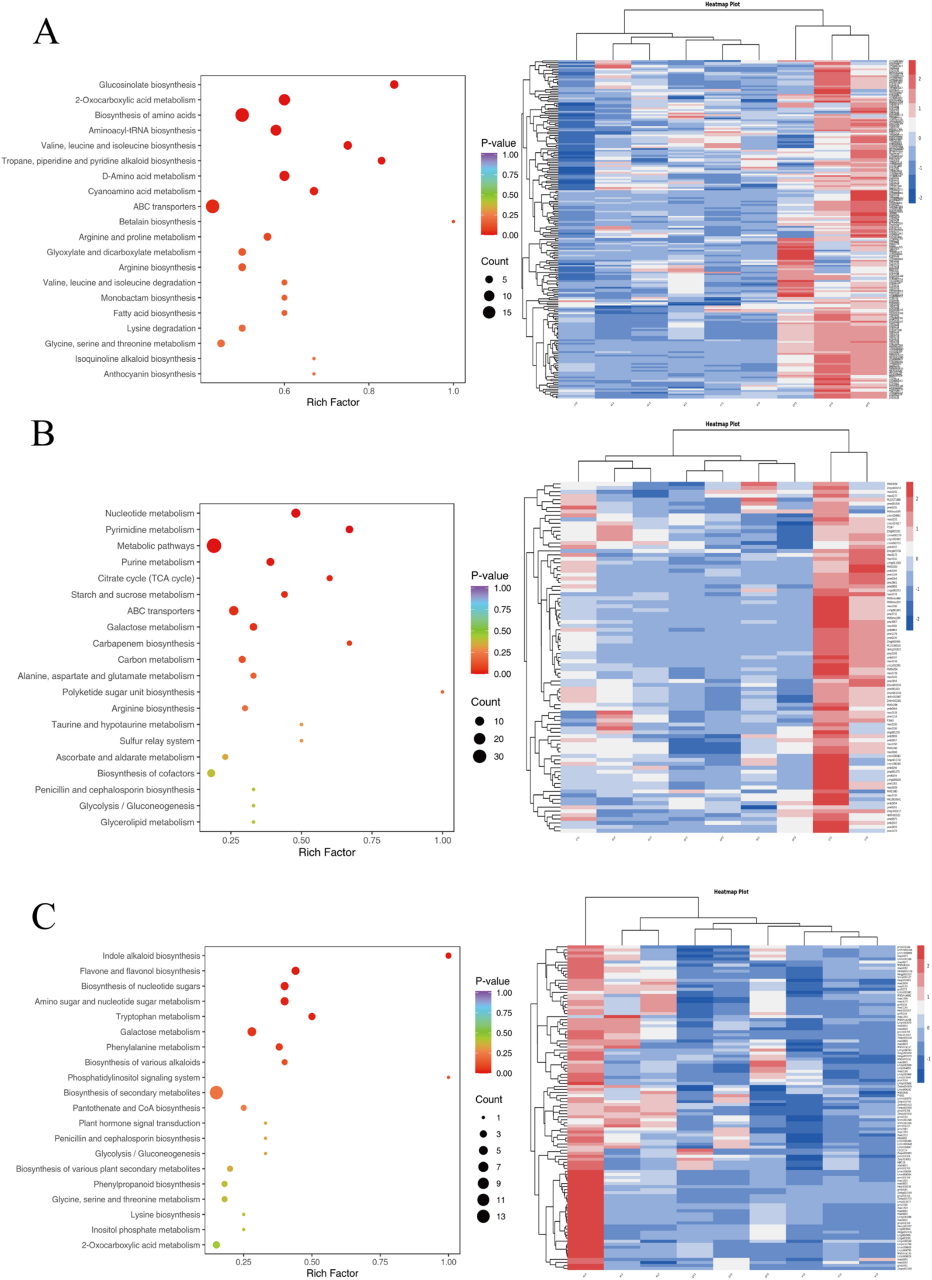
The metabolite properties of YY, XL, and DY plants were analyzed using KEGG. In this analysis, each bubble in the plot represents a metabolic pathway. To visualize the results, a bubble plot can be created to display the top 20 metabolic pathways, sorted by the *p*-value. The size of each bubble in the plot represents the impact factor of the pathway, while the horizontal position indicates the enrichment rate of metabolites within that pathway. The color of the bubbles represents the *p*-value of the enrichment analysis, with lighter colors indicating a higher confidence level. Additionally, heatmaps can be generated to illustrate the accumulation patterns of representative metabolites in the YY, XL, and DY. In the heatmap, red and blue colors indicate high and low expression levels, respectively, enabling a visual comparison of metabolite accumulation across the different modules.

We further identified the hub metabolites of the modules based on the data from WGCNA, and we examined the relationship between modules and bead ginseng cultivars. The 200 metabolites in the turquoise module exhibit a strong connection with cultivar (DY) (Fig. 4B). There are also metabolites involved in the formation of cofactors, secondary metabolites, zeatin biosynthesis, and the metabolic pathways of alpha-linolenic acid (Fig. 4C), as well as 36 amino acids and their derivatives and 31 phenolic acids (Fig. 5A). The yellow module correlates strongly with cultivar A (YY) and contains 89 metabolites, including nucleotides and derivatives (24), amino acids and derivatives (10), phenolic acids (12) and lipids (nine) (Fig. 4C). The top pathways enriched in this module are metabolic pathways, nucleotide metabolism, ABC transporters, galactose metabolism, and purine pathways (Fig. 5B).

The brown module correlates with cultivar B (XL) and is enriched in the following components: flavonoids (29), terpenoids (23), phenolic acids (19), alkaloids (eight), organic acids (six), lipids (six), and other class (Fig. 4C). Galactose metabolism and metabolites related to flavone and flavonol biosynthesis are over-represented in the brown module (Fig. 5C).

### Analysis of triterpene content and gene expression profile in three plants

**Figure 6:**
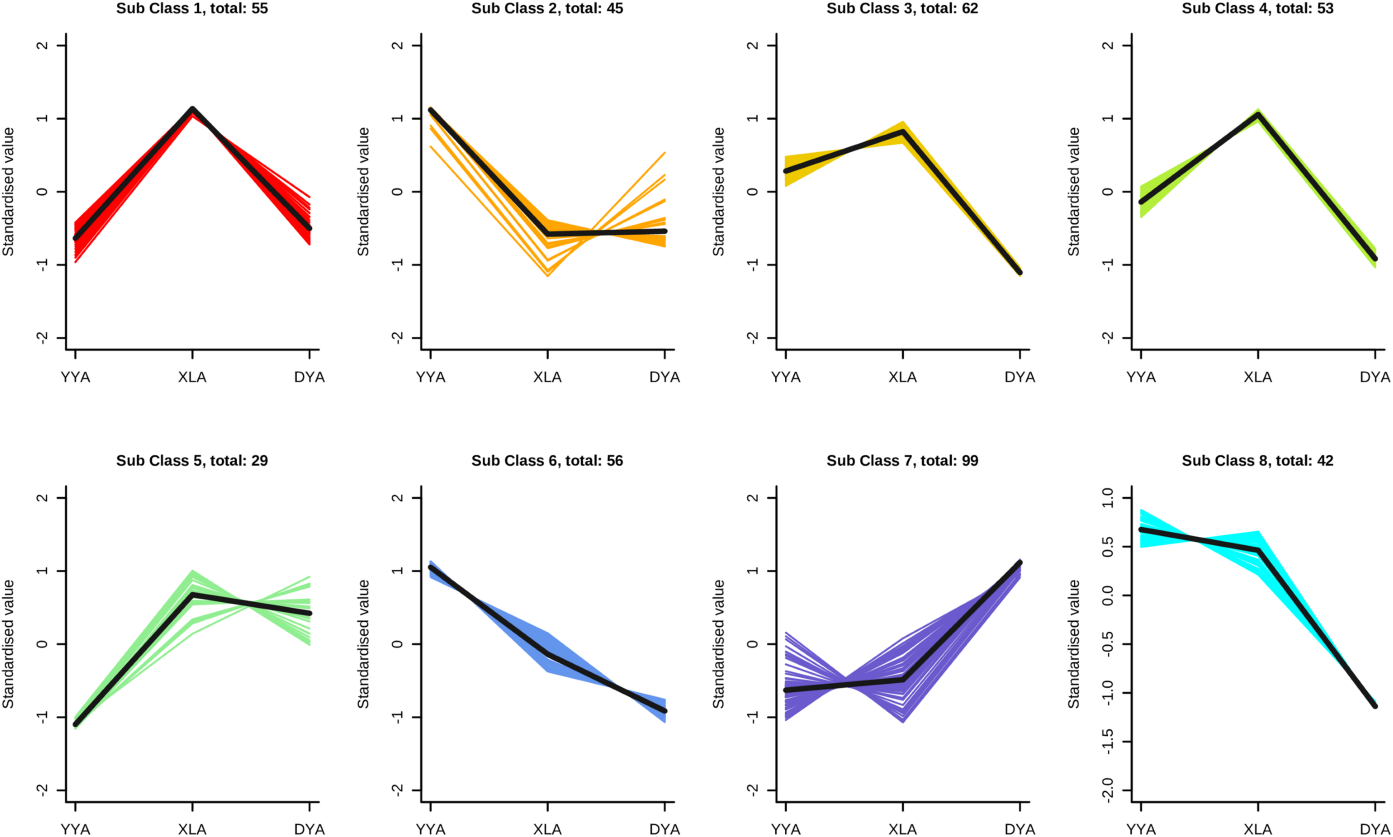
K-means clustering groups of the expression profile of the differential metabolites of three bead ginseng origins. The y-axis represented the standardized content per metabolite and the x-axis represented the different samples.

A Pearson correlation analysis revealed significant differences in metabolites among the three pearl ginseng plants. To understand the pattern of changes in these metabolites in the three plants, K-means clustering was used to group metabolites based on their similarities, resulting in eight main categories. From Fig. 6, it can be seen that DAMs in classes 1 and 5 were significantly upregulated in XL, while in class 7, the expression of DAMs did not differ significantiy between XL and YY, but had significantly higher expression in DY. In contrast, DAMs in DY gradually decreased during grades 3, 4, and 8. The greatest number of differential metabolites was found in class 7 (99). In the five subclusters (3, 5, 6, 7, 8), triterpene saponin content showed different trends of change. In grade seven, the triterpene saponin level was the highest in DY.

### Transcriptome analysis of three original of bead ginseng

Nine RNA samples were taken from the rhizomes of three different plants (DY, XL, and YY) and subjected to high-throughput RNA-Seq, with three biological replicates for each collection. Transcriptome sequencing yielded a total of 58.77 GB of clean data from nine samples, with more than 6 GB of clean data obtained from each sample, resulting in 41559390–45352352 clean reads and 6.23–6.89 clean bases for each library. The GC content varied between 41.99% and 43.77%. Among these, 73.91–80.59% of the clean reads were unambiguously assigned to the predicted coding sequences of the *Panax ginseng* genomic data. Meanwhile, 36.98–38.25% of the clean reads were assigned to the strand of coding sequences, while 36.93–38.10% were assigned to the strand of coding sequences (Table 1). The overall sequencing error rate was 0.03%. A total of 65,870 genes were found in all seven libraries after mapping, and 14,891 novel genes were discovered that were not listed in the reference genome data.

**Table 1:** Summary of the RNA-Seq data from different samples.

Sample	Raw reads	Clean reads	Clean reads rate (%)	Clean base (G)	Error rate (%)	Q20 (%)	Q30 (%)	GC content (%)
YYB1	49264472	43872620	89%	6.58	0.02	98.33	94.81	42.44
YYB2	46640914	44819668	96%	6.72	0.03	97.41	92.5	43.2
YYB3	47918788	45919426	96%	6.89	0.03	97.45	92.6	43.5
XLB1	45114924	43335066	96%	6.5	0.03	97.28	92.25	43.77
XLB2	47325040	45352352	96%	6.8	0.03	97.37	92.43	43.36
XLB3	44680770	42041068	94%	6.31	0.03	97.44	92.58	43.5
DYB1	44766862	42832554	96%	6.42	0.03	97.4	92.49	42.13
DYB2	44949184	41559390	92%	6.23	0.03	97.46	92.66	41.99
DYB3	45502152	42146418	93%	6.32	0.03	97.38	92.49	43.39

Based on the criteria of |log2Fold Change| >= 1 and FDR < 0.05, a total of 33,073 DEGs were identified in all samples, with 16,074, 15,705 and 1,294 being identified in XL *vs* DY(A), YY *vs* DY(B) and YY *vs* XL(C), respectively. In the XL *vs* DY group, 16,074 DEGs were detected, with 7,723 genes being upregulated and 8,351 genes being downregulated. In the YY *vs* DY group, 15,705 genes were differentially expressed, with 7,436 genes being upregulated and 8,269 genes being downregulated. However, for YY *vs* XL, only 1,294 genes were differentially expressed, with 531 genes upregulated and 763 genes downregulated. The differentially expressed genes were visualized in a Venn diagram. Differential gene analysis was performed on the three sample groups, and 181 differential genes were identified. In XL *vs* DY and YY *vs* DY, there were 3,698 and 2,834 unique genes, respectively, while in YY *vs* XL, there were 130 unique genes. The plants were divided into two categories based on whether the expression of three plants was high or low, with XL and YY in one class and DY considered as a separate class.

### Overrepresentation analyses of DEGs

DEG (Database of Essential Genes) profiling was conducted on three original samples of bead ginseng. Overrepresentation analysis and enrichment studies were carried out using GO and KEGG data. Based on the classification of 16,074 differentially expressed genes in group A (Fig. S6), 11,082 differentially expressed genes were identified in GO classification entries. Among these, there were 39,603 DEGs in biological processes, including 19,006 upregulated genes and 20,594 downregulated genes; 13,000 DEGs in cell composition, including 6,095 upregulated genes and 6,905 downregulated genes; and 16,592 DEGs in molecular function, including 7,822 upregulated genes and 8,770 downregulated genes. In group B, 65,630 differentially expressed genes were identified in GO classification entries based on the classification of 15,705 common differential genes (Fig. S6). Among these, there were 32,378 DEGs in biological processes, including 1,754 upregulated genes and 19,626 downregulated genes; 12,477 DEGs in cell composition, including 5,749 upregulated genes and 6,728 downregulated genes; and 15,875 DEGs in molecular function, including 7,485 upregulated genes and 8,390 downregulated genes. In group C, based on the classification of 1,594 common differential genes, 4,454 differentially expressed genes were identified in GO classification entries (Fig. S6). There were 2,441 DEGs in biological processes, including 1,485 upregulated genes and 956 downregulated genes; 851 DEGs in cell composition, including 369 upregulated genes and 482 downregulated genes; and 1,162 DEGs in molecular function, including 615 upregulated genes and 547 downregulated genes.

The most significantly elevated biological process (BP) in response to oxygen levels was observed in the XL group compared to the DY group, suggesting that this process may be more prevalent in the XL group than in the YY *vs* DY and YY *vs* XL groups. The second most crucial BP concept was the response to hypoxia and decreased oxygen levels. According to the major cellular component (CC) terminology displayed in Fig. 3A, the intrinsic and integral components of the mitochondrial inner membranes found to be more active in the XL *vs* DY than in the YY *vs* DY and YY *vs* XL groups. For instance, the most significant upregulated BP term in YY *vs* XL was the response to chitin, and the most significant upregulated CC term in YY *vs* DY was the respirasome, indicating difference across the three groups.

The KEGG Pathway Database can be used to functionally annotate cellular elements and their interactions within diverse biological signaling pathways. This pathway-based annotation enables a deeper comprehension of the biological roles of the unigenes by providing an overview of the numerous active metabolic processes within an organism. After annotating 16,074 group A DEGs into the KEGG database, it was found that 13,249 unigenes were clustered into 144 KEGG pathways (Table S3), while others were not annotated into the KEGG database pathways. The most frequently discovered pathways included secondary metabolite biosynthesis, plant-pathogen interactions, plant hormone signaling, carbon metabolism, and endoplasmic reticulum protein processing. In group B, after annotating of DEGs, it was revealed that 12,535 unigenes belonged to 144 KEGG pathways (Table S4), while the remaining 3,170 unigenes were unclassified. Five KEGG pathways were found to be enriched, with secondary metabolite production, plant-pathogen interaction, plant hormone signaling, protein processing in the endoplasmic reticulum, and ribosome being the most represented pathways. In group C, 911 unigenes were annotated with 109 KEGG pathways (Table S5), while the remaining 683 unigenes were unclassified. The most commonly represented pathways included metabolic pathways, biosynthesis of secondary metabolites, oxidative phosphorylation, plant-pathogen interaction, plant hormone signaling, and ribosome.

### Biosynthetic pathways of triterpene saponin

**Figure 7:**
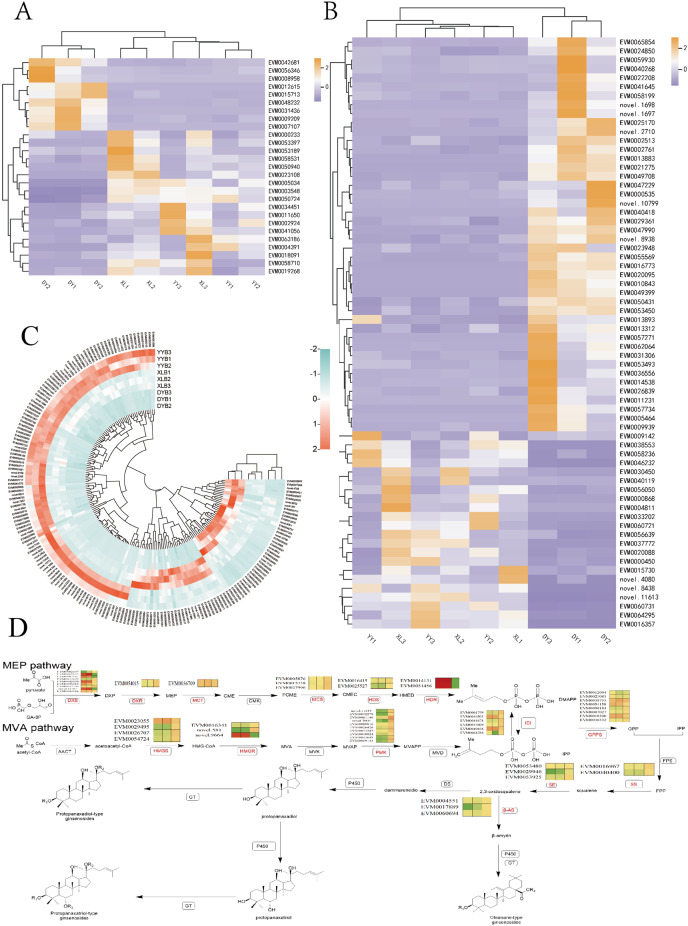
The biosynthesis of triterpene glycosides in the terpenoid skeletal framework. (A) Differential heat map of UDP glycosyltransferase (UGT) genes among DY, YY, and XL. (B) Differential heat map of cytochrome P450 (CYP450) genes among DY, YY, and XL. (C) The differences in the content of all terpene compounds among the three samples are presented in a heat map. (D) MEP (methylerythritol phosphate pathway) and mevalonic acid pathway, along with the pathway heat map for terpenoid biosynthesis.

Based on our metabolome data, it appears that terpenes, particularly triterpene saponin, play a significant role in influencing the therapeutic efficacy of bead ginseng. The differences in the content of all terpene compounds among the three samples are presented in a heat map (Fig. 7C). To elucidate the properties of the molecular biosynthetic pathway for triterpene saponin, we analyzed the differentially expressed genes (DEGs) and downstream pathways associated with terpenoid backbone biosynthesis, and identified 22 unigenes that are involved in this process.

The MVA pathway and the MEP pathway (Fig. 7D) have been identified as the main pathways involved in the biosynthesis of triterpenoid saponins. To examine the differences between the putative genes involved in triterpene saponin biosynthesis, we obtained a three-plant alignment for the three plants. Basic functional information for the transcriptomes from all three plants was processed using the SwissProt database. We found 14 differentially expressed sequences between the XL and DY groups, according to the alignment results. There were eight genes unique to XL, including seven sequences of genes encoding PMK types (novel.11643, EVM0012279). A set of 18 genes were differentially expressed according to B analysis, with four genes unique to YY, including three sequences of genes encoding PMK types (EVM0012279, novel.11842, EVM0043649), and one of the sequences of genes encoding types of HMGR (novel.581), with only two genes differentially expressed in group C. HMGR is a key regulatory site in MVA signaling and showed approximately 1.5-fold higher expression in YY than in XL. The gene (novel.581) expressed in DY was 0. The key rate-limiting enzymes in the endogenous synthesis of the MEP pathway are DXS and DXR.

The downstream pathway of triterpenoid saponins involves a number of modifications, including glycosylation and oxidation, which are catalyzed by Cytochrome P450 (CYP450) and UDP glycosyltransferase (UGT) (Figs. 7A and 7B). In group A, we found a total of 115 unigenes belonging to CYP450, to which 17 CYP450 families were annotated. Among them, 11 unigenes belonged to the CYP71 families, which were the most abundant. This was followed by the CYP72 and CYP82 families, with five and four unigenes, respectively. There were 32 unigenes annotated with UGT, with the UGT74 family being the most annotated six members. In group B, we found 118 unigenes belonging to CYP 450, to which 59 CYP450 families were annotated. Among them, eight unigenes belonged to the CYP 72 families, which were the most abundant. This was followed by CYP 81 and CYP 749 families, with six unigenes, respectively. There were 26 unigenes annotated with UGT, with the UGT74 family being the most abundant with six members. In group C, we found 18 unigenes belonging to CYP450, with 11 CYP450 families annotated. A total of 2 UGT families were found.

## Discussion

Bead ginseng has many pharmacological effects, such as anti-cancer, dual deficiency of qi and yin (World Health Organization, 2022), antimicrobial effect, and blood-enriching and bleeding-stopping properties. In addition to these medicinal values, many of these species also have edible and economic values. However, there are also environmental concerns associated with mass cultivation, and ensuring the quality of medicinal products can be difficult. The current supply of products on the drug market is insufficient, and the quality is often mixed. Accurate speciation is essential to ensure the clinical safety of medicinal products derived from bead ginseng. Therefore, this study is valuable to the future plant research community.

Firstly, transcriptomic analysis methods have significant importance in biological research as they help us understand the gene expression profile of cells or organisms under specific conditions. However, these methods also have some variability (due to differences in sequencing platforms, reagents and experimental errors, biological differences between samples, and differences in data preprocessing and analysis methods) and limitations (limited sample quantity and quality, quality of the reference genome, and biases in the data). To reduce the variability and limitations of transcriptomic analysis methods, the following strategies can be adopted: appropriate experimental design, control groups, and repeated experiments to improve the reliability of the data; standardization of experimental procedures to avoid unnecessary differences; use of appropriate analysis pipelines and reasonable parameter settings, and integration with other omics data (such as metabolomics) to address problems from multiple perspectives. Secondly, metabolomic analysis methods have high rationality in experimental design, selection of reagents and standards, data normalization, and statistical analysis methods. However, potential variability (effects of operator technique and equipment performance on the extraction efficiency and online analysis of metabolites, and variations resulting from different experimenters or time points in the same laboratory) and limitations (scope of biological samples, reagents, and standards only applicable to specific types of experiments, limited generalizability, need for evaluation of the adaptability range, and inherent variability in data normalization and analysis methods such as UV Scaling, which can only partially eliminate endogenous differences but cannot completely eliminate differences caused by operational conditions and preparation environment; and the possibility of extreme values in the sample set affecting statistical models and the screening of differential metabolites using OPLS-DA) also exist. In summary, metabolomic analysis methods should optimize each experimental stage, select appropriate statistical methods, and use high-quality reference databases to reduce the impact of variability and limitations on the results.

To our knowledge, this is the first reported full-length transcriptome and metabolomics study for three species. Our metabolomics study detected a total of 830 metabolomes in all samples, with the metabolome consisting mainly of flavonoids, phenolic acids and terpenoids. The terpenes group was primarily composed of darmmarane-type triterpene saponins, flavonoids were mainly flavonols, phenolic acids were mainly innamic acid and its derivatives. WGCNA analysis showed that the brown module was positively associated with XL, the yellow module correlated strongly with strain YY and contained 89 metabolites, and the turquoise module was positively associated with the DY module. Metabolite-related genes were identified by analyzing a combination of metabolomics and transcriptomics data. It was found that the differential expression of genes between XL-*vs*-DY, YY-*vs*-DY, and YY-*vs*-XL were associated with different secondary metabolic pathways, namely monoterpenoid biosynthesis, terpenoid backbone biosynthesis, sesquiterpenoid and diterpenoid biosynthesis, triterpenoid biosynthesis, and ubiquinone and other terpenoid-quinone biosynthesis. Genes such as HMGR, PMK, CYP450, and UGT were found to be involved in these pathways.

Polyphenols are involved in both plant and human defense mechanisms, particularly against oxidative stress. As a standard for evaluating the quality of bead ginseng a must not be less than 3%. The content of Chikusetsusaponin IVa was detected in all three plants. We compared metabolomic and transcriptomic data from three independent plants and revealed differences in the properties of the three, which mainly contained flavonoids, phenolic acids and terpenes. Phenolic acids have been found to exhibit a variety of pharmacological activities, such as cardiovascular and cerebrovascular effects, anti-tumor, anti-oxidation, anti-inflammation, and anti-fibrosis. In *Lonicera japonica thunb*, phenolic acids have excellent antiviral activity (Lee, Chang & Yeh, 2021; Xin, Zheng-you & Pu, 2020). Previous studies have demonstrated that phenolics exhibit antiviral properties, particularly against RNA viruses. In recent research, ginseng has emerged as a promising candidate for potential therapeutic interventions against SARS-CoV-2. A mini review by Ratan provides insights into the potential of ginseng as a choice for SARS-CoV-2 treatment (Ratan, Rabbi Mashrur & Runa, 2022). The study suggests that ginseng’s bioactive compounds may contribute to its efficacy as a COVID-19 therapeutic. This will help control and treat COVID-19, a serious problem for all humanity (Baden & Rubin, 2020; Kupferschmidt & Cohen, 2020). Patients infected with COVID-19 may experience severe lung damage, acute respiratory distress syndrome, and respiratory failure, which is also an important reason for death from COVID-19. A variety of inflammatory factors (such as TNF- α, IL-6, IL-8, *etc*.) lead to a number of complications (Sahebnasagh, Mojtahedzadeh & Najmeddin, 2020). Honeysuckle phenolic acids have been found to have anti-inflammatory effects and can inhibit the expression of various inflammatory factors (Ya-ling, Hong-mei & Fu-yong, 2015; Jiuling, Xiaoling & Chengwen, 2013; Gao, Wang & Yu, 2019; Shin, Satsu & Bae, 2017; Liang & Kitts, 2018; Jin-qian, Zhao-ping & Heng, 2016; Olmos, Giner & Recio, 2007; Jue, Naili & Xin-sheng, 2006; Olmos, Giner & Recio, 2008; Chen, Hwang & Yu, 2016; Zhicong, An & Ping, 2016), thereby preventing SARS CoV-2 from entering the human body (Yu, Wang & Bao, 2020).

Wei, Xiao-yan & Xiao-bo (2015) created a rat model of learning and memory disorders, studied the effect of Epimedium’s total flavonoids on the model, and suggested that Epimedium’s total flavonoids significantly improved the behavioral errors of rats, enhancing and improving their learning and memory ability. The mechanism was mainly to reduce calcium influx, regulate the expression of Bax protein and Bcl-2 protein in hippocampal tissue, and then inhibit the apoptosis of hippocampal neurons. Yamamoto, Joku Ra & Suzuki (2013) studied the flavonoids hesperidin and hesperidin, which can inhibit the synthesis of TXA2 synthase and TXB2 synthase of vascular endothelial cells and thereby inhibit platelet aggregation. Atherosclerosis can cause coronary artery disease, myocardial ischemia, cerebral infarction and other cardiovascular diseases. Anandhi, Thomasp & Ger Aldin (2014) found that flavonoids such as chrysin improve antioxidant activity by reducing MDA levels in the liver, thereby preventing atherosclerosis. Liu, Yu & Sun (2020) isolated honeysuckle flavonoids and evaluated their anti-inflammatory and antioxidant activities. They produced a 2,4,6-trinitrobenzenesulfonic acid-induced ulcerative colitis model in rats and discovered that it affects the NF-B signaling pathway, superoxide dismutase (SOD), myeloperoxidase (MPO), malondialdehyde (MDA), prostaglandin E2 (PGE2), tumor necrosis factor (TNF), interleukin (IL) and C-reactive protein (CRP), thereby improving ulcerative colitis. Bo-lan, Ting & Lu (2014) found that daidzein can increase the expression level of antioxidant enzymes GST, CAT, SOD, and other genes and induce the expression of the antioxidant element ARE in breast cancer cell MCF7, human ovarian cancer cell SK-OV-3, and cervical cancer cell HeLa, reducing oxidative stress. In summary, flavonoids have pharmacological activities such as gastric ulcer treatment, neuroprotection, myocardial ischemia treatment, antihypertensive effects, enhancing learning and memory, protecting reproductive tissue, anti-inflammatory, antibacterial, antiviral, antitumor and hypoglycemic effects.

We performed transcriptome analysis by RNA sequencing, and the MVA pathway has been identified as the principal biosynthetic route for triterpenoid saponins. Taken together, these findings suggest that the levels of the different metabolites, which vary widely across the three plants, could be due to changes in the expression of these differentially expressed genes (DEGs). These findings are essential for comprehending the molecular mechanism of the principal active chemicals of bead ginseng. By identifying differentially expressed genes that participate in the MVA pathway, we can gain a better understanding of how these active chemicals are synthesized in the plant. This knowledge holds great potential for developing more targeted and effective treatments in clinical settings.

## Conclusions

The medicinal value of bead ginseng is exceptionally high. To our knowledge, there have been no reports of metabolic and transcriptomic comparisons among these three species before this study. In this study, metabolic and transcriptomic analyses of three species were used to investigate drug activity components and gene differential expression. Metabolomics comparisons revealed the characteristics of these three species, including their main metabolites such as phenolic acids, lipids, and terpenoids, and their subtypes. Ultra-high performance liquid chromatography-electrospray ionization mass spectrometry was used to compare and analyze the metabolites in these three species. A total of 830 metabolites were detected, and 748 metabolites were found to be the same among the three species, including phenolic acids, lipids, flavonoids, and terpenoid compounds. Specific analysis of terpenoid compounds in these three species revealed that 69% of them were triterpenoid saponins. RNA sequencing has described the biosynthetic pathway of triterpenoid saponins, and the MVA pathway has been identified as the principal biosynthetic route for these compounds. Furthermore, the quantitative results of the metabolites were reliable and repeatable. Transcriptome analysis identified 33,073 differentially expressed genes, among which some genes were found to be related to differences in metabolite content. Additionally, GO and KEGG pathway analyses revealed that some of these genes were involved in secondary metabolite biosynthesis, plant-pathogen interactions, and plant hormone signal transduction. These findings provide guidance for cultivating and utilizing these plants in medicine. By exploring the metabolites and associated gene expression of three species of primitive plants, *Panax pseudoginseng* and *Panax pseudo-ginseng* var. *elegantior* have many similarities and can be placed into the same category. These results can provide a basis for the identification of *Panax japonicus* and have important implications for the protection, planting, and rational use of rare medicinal ginseng materials.

## Supplemental Information

10.7717/peerj.16034/supp-1 Supplemental Information 1 PCA and OPLS-DA plots of the widely targeted metabolome data.

10.7717/peerj.16034/supp-2 Supplemental Information 2 Overlapping TIC of the QC mixtures in positive and negative ionization modes in the widely targeted metabolom.

10.7717/peerj.16034/supp-3 Supplemental Information 3 Orthogonal partial least squares discriminant analysis (OPLS-DA).

10.7717/peerj.16034/supp-4 Supplemental Information 4 Group B distribution of differentially accumulated metabolites (DAMs) in the broad metabolomes.

10.7717/peerj.16034/supp-5 Supplemental Information 5 Group C distribution of differentially accumulated metabolites (DAMs) in the broad metabolomes.

10.7717/peerj.16034/supp-6 Supplemental Information 6 GO enrichment top50 and statistics of KEGG enrichment.

10.7717/peerj.16034/supp-7 Supplemental Information 7 Metabolome information of the widely-targeted metabolome.

10.7717/peerj.16034/supp-8 Supplemental Information 8 A total of differential metabolites identified in at least one pairwise comparison.

10.7717/peerj.16034/supp-9 Supplemental Information 9 KEGG enrichment outcome of differential genes of XL *vs* DY group.

10.7717/peerj.16034/supp-10 Supplemental Information 10 KEGG enrichment outcome of differential genes of YY *vs* DY group.

10.7717/peerj.16034/supp-11 Supplemental Information 11 KEGG enrichment outcome of differential genes of YY *vs* XL group.

10.7717/peerj.16034/supp-12 Supplemental Information 12 MRM transition list.

## Additional Information and Declarations

### Data Availability

The following information was supplied regarding data availability: The data is available at NCBI: PRJNA946806.
