# Supplementary figures and images for: Metabolome and transcriptome analyses identify the characteristics and expression of related saponins of the three genealogical plants of bead ginseng
Source: PeerJ. 2023 Sep 1;11:e16034. doi: 10.7717/peerj.16034 (PMC10476608; doi:10.7717/peerj.16034)

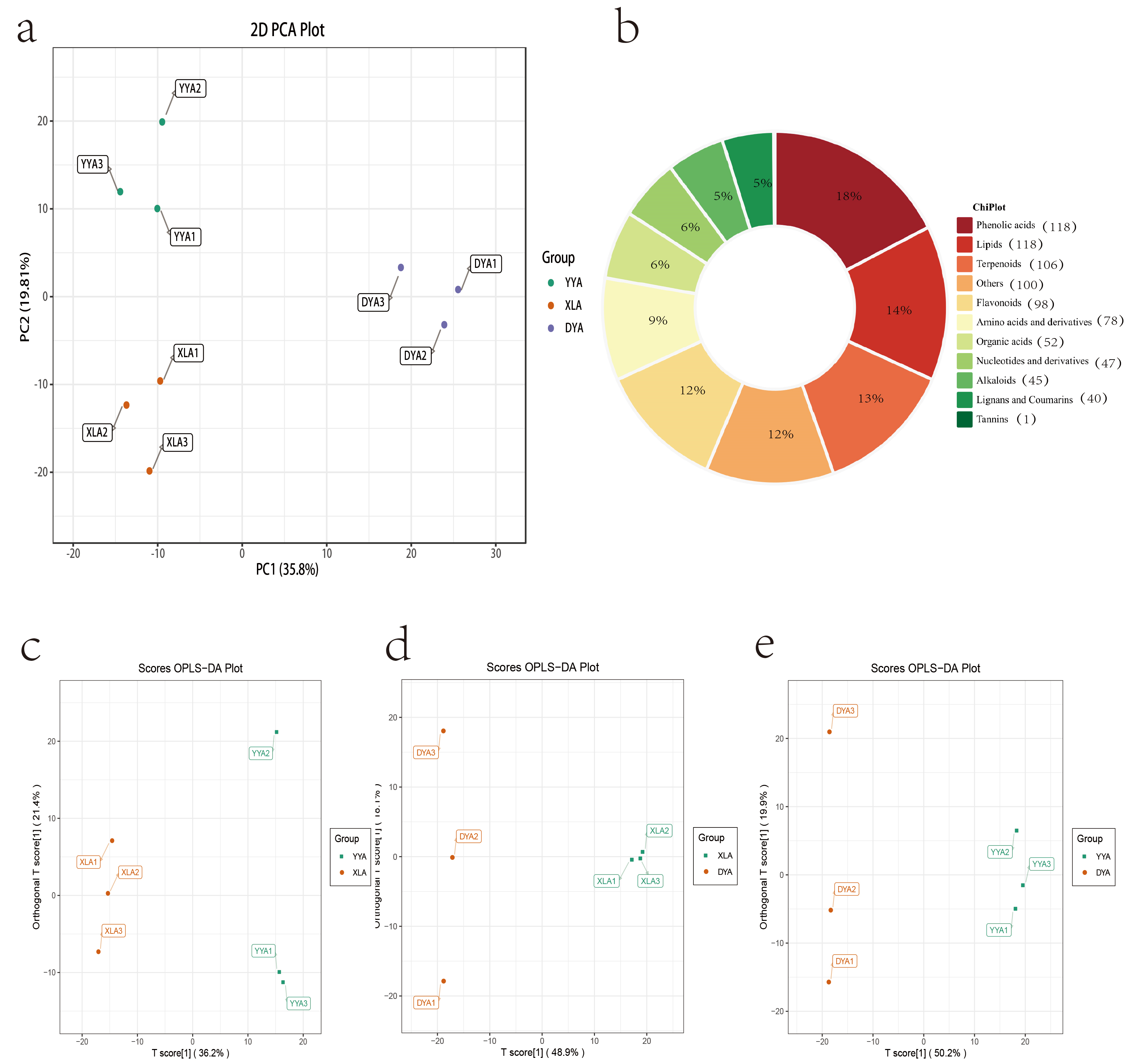

Supplement: Supplemental Information 1 [file peerj-11-16034-s001.png]

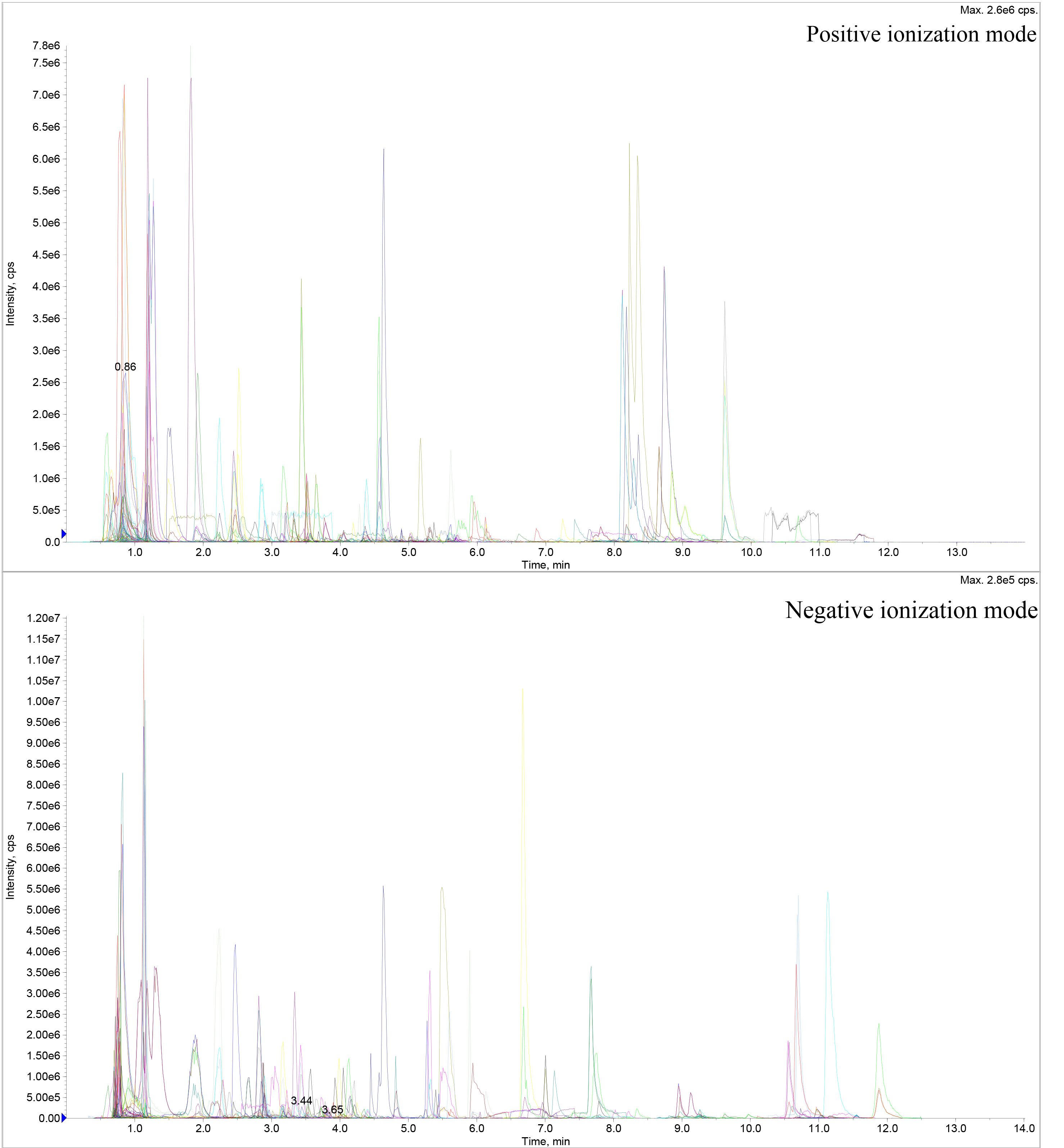

Supplement: Supplemental Information 2 [file peerj-11-16034-s002.png]

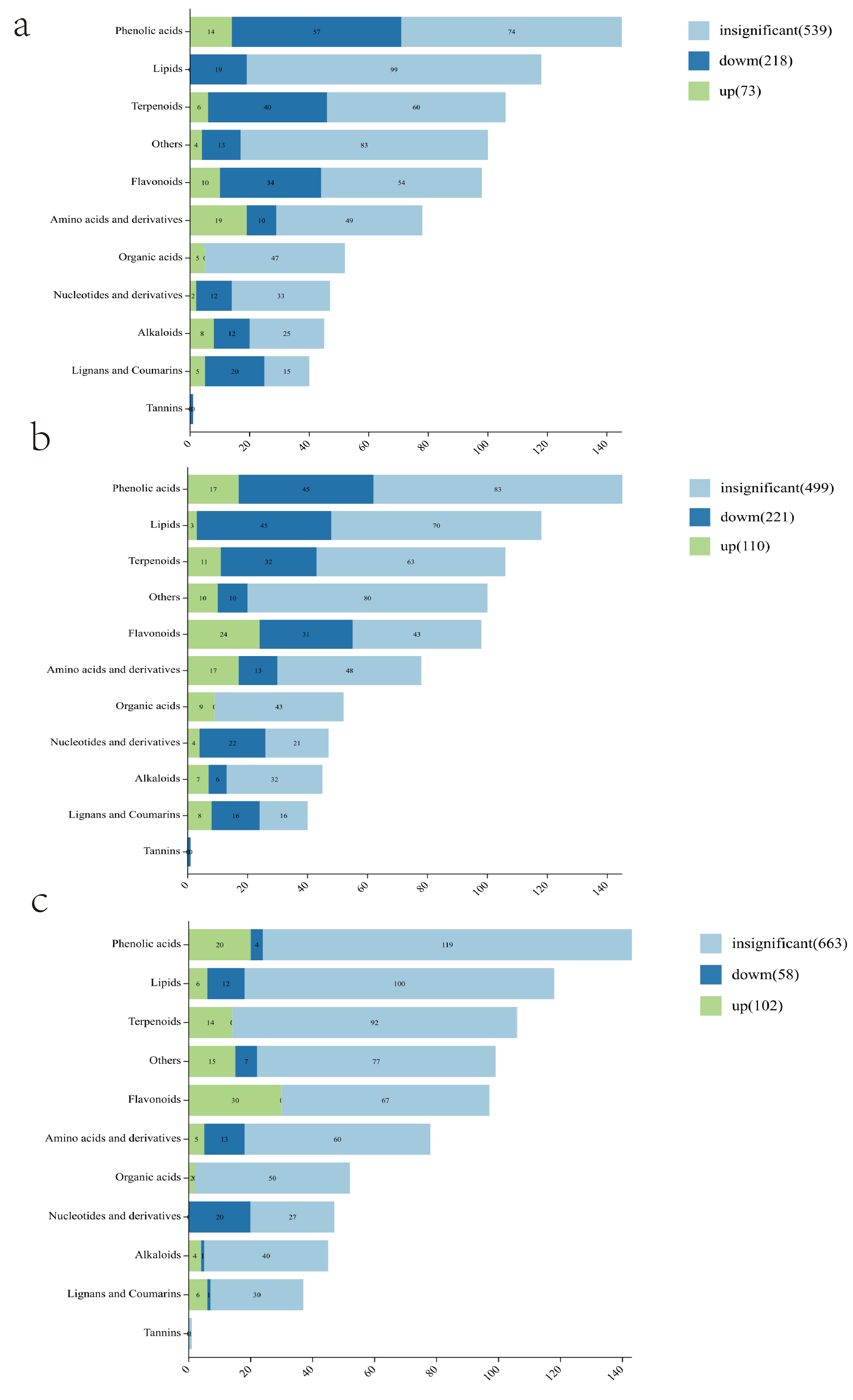

Supplement: Supplemental Information 3 [file peerj-11-16034-s003.png]

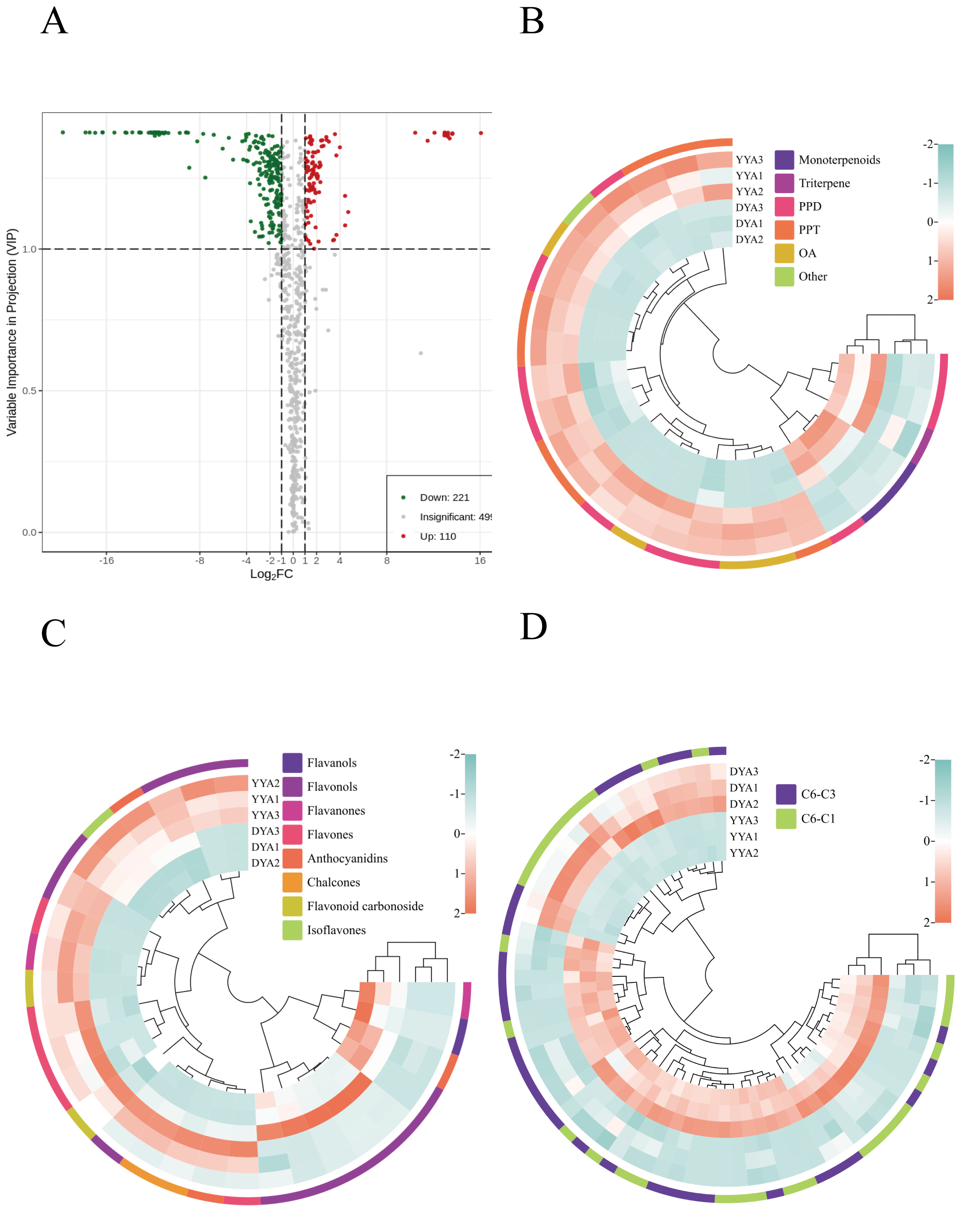

Supplement: Supplemental Information 4 [file peerj-11-16034-s004.png]

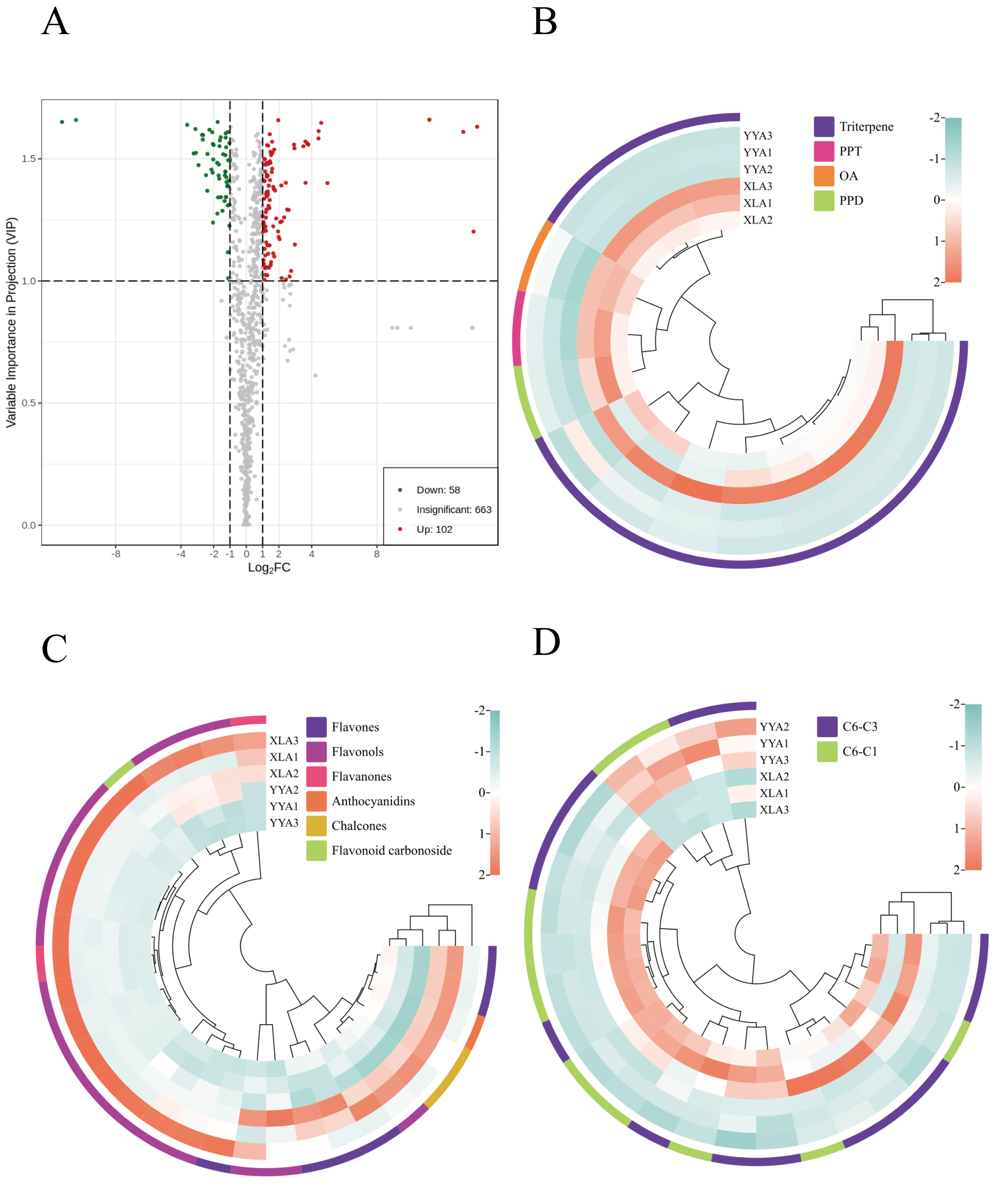

Supplement: Supplemental Information 5 [file peerj-11-16034-s005.png]

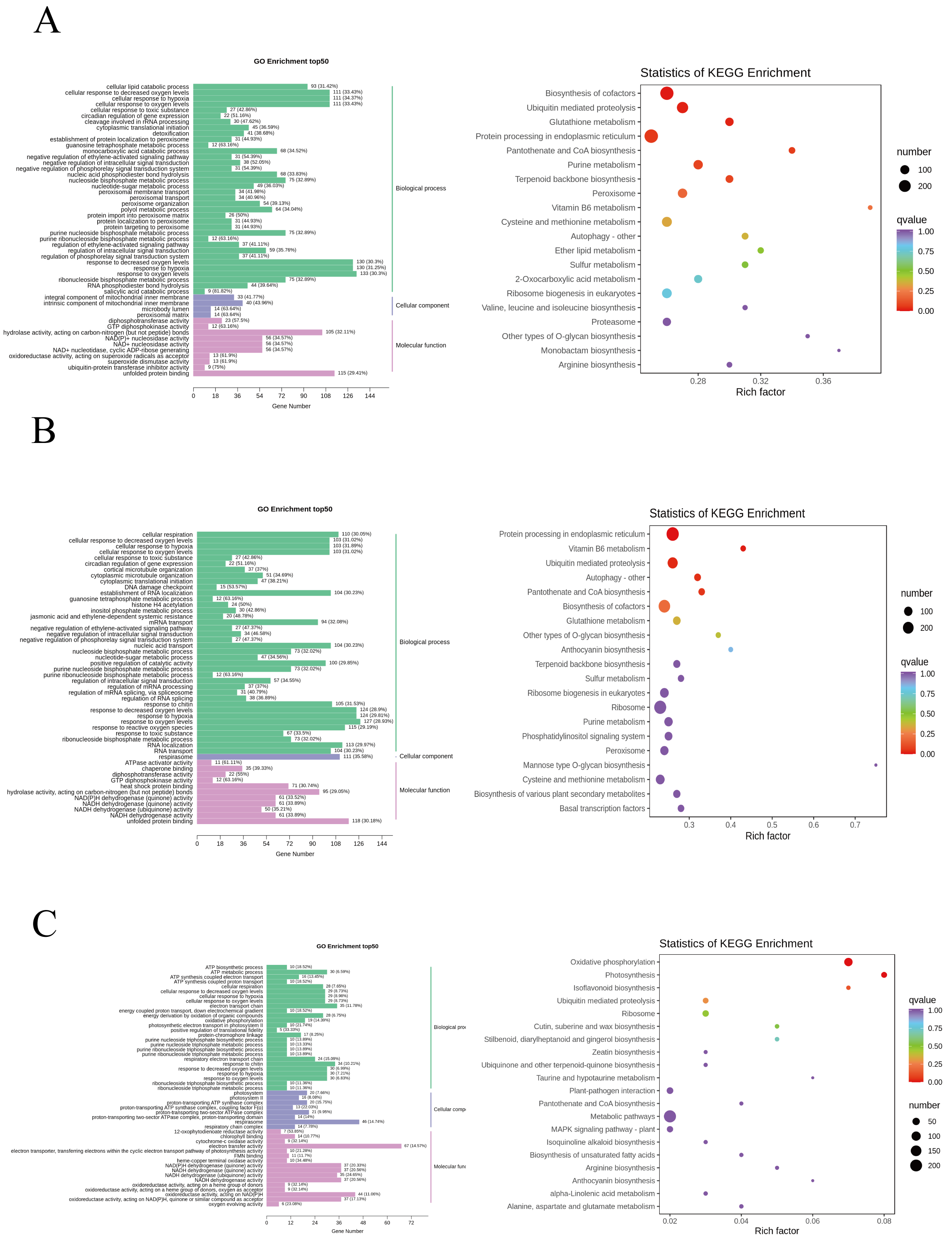

Supplement: Supplemental Information 6 [file peerj-11-16034-s006.png]
