# Supplementary material for: Metabolome and transcriptome analyses identify the characteristics and expression of related saponins of the three genealogical plants of bead ginseng
Source: PeerJ. 2023 Sep 1;11:e16034. doi: 10.7717/peerj.16034 (PMC10476608; doi:10.7717/peerj.16034)
Supplement: Supplementary file 2 [file peerj-11-16034-s007.odt]

Index

pmb0782

Q1 (Da)

8.61E+01

1.04E+02

1.04E+02

1.04E+02

1.04E+02

1.03E+02

1.03E+02

1.03E+02

1.06E+02

1.06E+02

1.10E+02

1.12E+02

1.12E+02

1.16E+02

1.15E+02

1.18E+02

1.18E+02

1.17E+02

1.17E+02

1.17E+02

1.20E+02

1.20E+02

1.19E+02

1.21E+02

1.20E+02

1.22E+02

1.21E+02

1.23E+02

1.24E+02

1.22E+02

1.24E+02

1.27E+02

1.28E+02

1.28E+02

1.30E+02

1.30E+02

1.30E+02

1.29E+02

1.32E+02

1.32E+02

1.30E+02

1.32E+02

1.32E+02

1.32E+02

1.32E+02

Q3 (Da)

6.92E+01

5.80E+01

5.90E+01

8.60E+01

6.01E+01

5.90E+01

5.90E+01

5.91E+01

6.00E+01

8.80E+01

6.50E+01

9.50E+01

7.00E+01

7.01E+01

7.10E+01

7.21E+01

5.90E+01

7.30E+01

7.30E+01

5.90E+01

7.40E+01

1.03E+02

5.90E+01

9.31E+01

6.20E+01

1.05E+02

9.20E+01

8.01E+01

7.80E+01

7.80E+01

7.80E+01

8.12E+01

8.20E+01

8.20E+01

8.41E+01

8.41E+01

8.41E+01

8.50E+01

8.61E+01

8.60E+01

8.80E+01

6.90E+01

8.62E+01

5.71E+01

8.60E+01

Molecular Weight (Da)

8.51E+01

1.03E+02

1.03E+02

1.03E+02

1.03E+02

1.04E+02

1.04E+02

1.04E+02

1.05E+02

1.05E+02

1.09E+02

1.11E+02

1.11E+02

1.15E+02

1.16E+02

1.17E+02

1.17E+02

1.18E+02

1.18E+02

1.18E+02

1.19E+02

1.19E+02

1.20E+02

1.20E+02

1.21E+02

1.21E+02

1.22E+02

1.22E+02

1.23E+02

1.23E+02

1.23E+02

1.26E+02

1.29E+02

1.29E+02

1.29E+02

1.29E+02

1.29E+02

1.30E+02

1.31E+02

1.31E+02

1.31E+02

1.31E+02

1.31E+02

1.31E+02

1.31E+02

Formula

C5H11N

pme3017

C4H9NO2

C4H9NO2

C4H9NO2

C5H13NO

C3H4O4

MWSmce58

pme3033

pmb0484

ML1017216

Lmbn000193

mws0576

pme0010

C3H4O4

C4H8O3

C3H7NO3

C4H11NO2

C6H7NO

pme2433

MWS2076

mws0255

MWS5173

pme0006

C4H5N3O

C4H5N3O

C5H9NO2

C4H4O4

mws0376

mws0256

mws0191

mws0192

mws0470

mws0147

mws0230

pmp001287

MWS5147

pma0134

C5H11NO2

C5H11NO2

C4H6O4

C4H6O4

C5H10O3

C4H9NO3

C8H9N

C4H8O2S

C4H8O4

pme0195

C3H7NO2S

C8H11N

mws0491

mws0628

mws0133

pme0490

C7H6O2

C6H6N2O

C6H5NO2

C6H5NO2

C6H5NO2

C5H6N2O2

C5H7NO3

C5H7NO3

C6H11NO2

C6H11NO2

C5H11N3O

C5H6O4

pme1216

Lmbp000668

pmb0812

mws0263

MWS0813

MWS0811

ML1018166

Zmdp000376

Zmgn000217

pma3649

C5H9NO3

C5H9NO3

C4H9N3O2

C6H13NO2

C6H13NO2

C6H13NO2

C6H13NO2

mws0216

ML1019792

pme0274

mws0227

pmp001198

mws1587

 

mws0258

pme2679

1.32E+02

1.32E+02

1.31E+02

1.31E+02

1.31E+02

1.31E+02

1.31E+02

1.33E+02

1.31E+02

1.33E+02

1.32E+02

1.32E+02

1.33E+02

1.33E+02

1.36E+02

1.36E+02

1.36E+02

1.35E+02

1.35E+02

1.35E+02

1.38E+02

1.38E+02

1.37E+02

1.37E+02

1.37E+02

1.37E+02

1.40E+02

1.42E+02

1.44E+02

1.44E+02

1.44E+02

1.44E+02

1.44E+02

1.45E+02

1.46E+02

1.46E+02

1.46E+02

1.45E+02

1.47E+02

1.47E+02

1.47E+02

1.48E+02

1.46E+02

1.48E+02

1.49E+02

1.47E+02

1.47E+02

8.60E+01

8.60E+01

8.71E+01

8.71E+01

8.70E+01

8.71E+01

7.00E+01

7.40E+01

8.50E+01

1.16E+02

8.80E+01

8.80E+01

7.10E+01

7.10E+01

9.11E+01

1.19E+02

9.11E+01

7.50E+01

9.20E+01

9.20E+01

7.70E+01

1.20E+02

9.30E+01

1.08E+02

9.30E+01

9.30E+01

7.90E+01

1.24E+02

5.81E+01

9.81E+01

8.41E+01

8.41E+01

8.41E+01

8.61E+01

1.18E+02

8.21E+01

8.70E+01

1.09E+02

8.40E+01

8.40E+01

8.72E+01

8.40E+01

1.02E+02

8.80E+01

6.50E+01

8.50E+01

8.71E+01

1.31E+02

1.31E+02

1.32E+02

1.32E+02

1.32E+02

1.32E+02

1.32E+02

1.32E+02

1.32E+02

1.32E+02

1.33E+02

1.33E+02

1.34E+02

1.34E+02

1.35E+02

1.35E+02

1.35E+02

1.36E+02

1.36E+02

1.36E+02

1.37E+02

1.37E+02

1.38E+02

1.38E+02

1.38E+02

1.38E+02

1.41E+02

1.41E+02

1.43E+02

1.43E+02

1.43E+02

1.43E+02

1.44E+02

1.44E+02

1.45E+02

1.45E+02

1.45E+02

1.46E+02

1.46E+02

1.46E+02

1.46E+02

1.47E+02

1.47E+02

1.47E+02

1.48E+02

1.48E+02

1.48E+02

C6H13NO2

C6H13NO2

C5H8O4

Lmmn00216

mws0473

C5H8O4

Lmbn001288

Lmmn00080

Lmgn000160

mws0001

C5H8O4

C5H8O4

C4H8N2O3

C4H8N2O3

C6H12O3

C5H12N2O2

C4H7NO4

C4H7NO4

C4H6O5

mws0972

pme2527

mws0219

MWS1882

Lmbn000198

mws0275

C4H6O5

Zmdp000972

pme0040

C4H9NO2S

C5H5N5

HJKP000649

mws0889

C8H9NO

C4H8O5

MWSmce46

MWS1848

Lmqp002784

mws1336

C8H8O2

C8H8O2

C7H7NO2

C7H7NO2

C7H6O3

Lmbn002862

Lmgn001670

Lmbn001981

Hmgn00165

mws0704

C7H6O3

C7H6O3

C7H6O3

C2H8NO4P

C6H11N3O

C7H13NO2

C7H13NO2

C7H13NO2

C8H17NO

C7H14NO2+

C7H16N2O

C9H7NO

pma2987

MWSmce15

Hmsp000364

Rfmb320

Hmcp000405

Rfmb318

MWS3020

Hmmp00212

Lmqp000427

mws0567

C6H11NO3

C5H11N3O2

C6H10O4

C5H10N2O3

C6H14N2O2

C7H16NO2

C5H9NO4

C5H9NO4

C5H9NO4

C8H4O3

Wmzn00022

pme0193

pme0026

pme1691

pme0014

Zmzn000113

mws1050

pmp001285

MWS3036

pmb2826

C5H8O5

C5H8O5

 

Zmyn000230

Lmbn000216

Zmyn000247

ML1017184

Lmgp000796

pme1210

1.47E+02

1.47E+02

1.47E+02

1.47E+02

1.50E+02

1.50E+02

1.50E+02

1.49E+02

1.49E+02

1.49E+02

1.52E+02

1.52E+02

1.51E+02

1.53E+02

1.51E+02

1.53E+02

1.53E+02

1.56E+02

1.58E+02

1.57E+02

1.57E+02

1.58E+02

1.59E+02

1.60E+02

1.62E+02

1.60E+02

1.61E+02

1.61E+02

1.63E+02

1.63E+02

1.64E+02

1.66E+02

1.65E+02

1.67E+02

1.65E+02

1.65E+02

1.65E+02

1.66E+02

1.68E+02

1.69E+02

1.67E+02

1.69E+02

1.70E+02

1.70E+02

1.70E+02

1.69E+02

1.71E+02

5.70E+01

5.70E+01

5.70E+01

8.50E+01

6.10E+01

6.10E+01

1.33E+02

7.50E+01

5.90E+01

5.90E+01

1.35E+02

1.35E+02

1.51E+02

6.50E+01

1.36E+02

8.31E+01

1.09E+02

1.10E+02

9.51E+01

1.57E+02

1.57E+02

1.16E+02

9.71E+01

1.16E+02

9.80E+01

9.80E+01

9.90E+01

7.10E+01

1.19E+02

1.19E+02

1.20E+02

1.20E+02

1.21E+02

1.21E+02

7.50E+01

5.90E+01

1.49E+02

8.00E+01

1.50E+02

1.23E+02

1.08E+02

6.50E+01

1.34E+02

1.24E+02

1.24E+02

7.90E+01

9.91E+01

1.48E+02

1.48E+02

1.48E+02

1.48E+02

1.49E+02

1.49E+02

1.49E+02

1.50E+02

1.50E+02

1.50E+02

1.51E+02

1.51E+02

1.52E+02

1.52E+02

1.52E+02

1.52E+02

1.54E+02

1.55E+02

1.57E+02

1.58E+02

1.58E+02

1.59E+02

1.60E+02

1.61E+02

1.61E+02

1.61E+02

1.62E+02

1.62E+02

1.64E+02

1.64E+02

1.65E+02

1.65E+02

1.66E+02

1.66E+02

1.66E+02

1.66E+02

1.66E+02

1.67E+02

1.67E+02

1.68E+02

1.68E+02

1.68E+02

1.69E+02

1.69E+02

1.69E+02

1.70E+02

1.72E+02

C5H8O5

C5H8O5

C5H8O5

C5H8O5

C8H7NO2

C5H11NO2S

C6H7N5

mws0847

Zmgn000173

MWSmce68

mws1499

C5H10O5

C5H10O5

C5H10O5

C5H5N5O

C5H5N5O

C5H4N4O2

C8H8O3

pme0183

pme1109

pme0256

MWSmce04

mws0458

C8H8O3

MWS1962

mws0639

C10H16O

C7H6O4

mws0254

C6H9N3O2

C8H15NO2

C10H22O

C10H22O

C7H13NO3

C7H12O4

C9H7NO2

C6H11NO4

C6H11NO4

C6H10O5

C6H10O5

C9H8O3

MWS1837

pmf0175

pmf0174

Lmbn001467

Lmbn002072

mws1417

mws1346

pme3382

pme2914

MWS0559

Lmmn00164

MWSmce24

pme3083

C9H8O3

C8H7NO3

C9H11NO2

C8H6O4

pme0021

pme0281

pmc0274

C6H6N4S

C5H10O6

C6H14O5

C10H14O2

C7H5NO4

C8H9NO3

C8H8O4

mws0344

MWSmce16

pmn001681

MWS3270

Zmjp000624

Lmmp00304

mws0028

C8H8O4

Hmbp00127

pme1383

C8H8O4

C8H11NO3

C7H11N3O2

C7H11N3O2

C3H7O6P

C9H16O3

MWS0933

pme0181

Zmzn000078

Lmbn003524

 

MA1003949

mws0154

1.73E+02

1.73E+02

1.73E+02

1.73E+02

1.73E+02

1.75E+02

1.73E+02

1.74E+02

1.76E+02

1.75E+02

1.75E+02

1.75E+02

1.75E+02

1.75E+02

1.75E+02

1.75E+02

1.77E+02

1.77E+02

1.78E+02

1.79E+02

1.79E+02

1.79E+02

1.79E+02

1.79E+02

1.79E+02

1.79E+02

1.79E+02

1.79E+02

1.81E+02

1.82E+02

1.82E+02

1.81E+02

1.81E+02

1.81E+02

1.81E+02

1.81E+02

1.84E+02

1.84E+02

1.85E+02

1.88E+02

1.88E+02

1.87E+02

1.87E+02

1.87E+02

1.89E+02

1.89E+02

1.89E+02

7.10E+01

9.30E+01

5.80E+01

1.11E+02

1.31E+02

1.16E+02

1.73E+02

8.80E+01

1.13E+02

5.90E+01

8.70E+01

8.50E+01

1.03E+02

1.15E+02

1.15E+02

1.15E+02

1.17E+02

1.45E+02

1.36E+02

1.35E+02

5.90E+01

5.90E+01

5.90E+01

5.90E+01

5.90E+01

1.46E+02

1.46E+02

9.20E+01

1.07E+02

1.36E+02

9.11E+01

1.07E+02

1.51E+02

7.10E+01

1.01E+02

1.01E+02

1.48E+02

1.25E+02

1.85E+02

1.18E+02

1.18E+02

1.23E+02

1.25E+02

1.69E+02

8.60E+01

1.26E+02

8.61E+01

1.74E+02

1.74E+02

1.74E+02

1.74E+02

1.74E+02

1.74E+02

1.74E+02

1.75E+02

1.75E+02

1.76E+02

1.76E+02

1.76E+02

1.76E+02

1.76E+02

1.76E+02

1.76E+02

1.78E+02

1.78E+02

1.79E+02

1.80E+02

1.80E+02

1.80E+02

1.80E+02

1.80E+02

1.80E+02

1.80E+02

1.80E+02

1.80E+02

1.80E+02

1.81E+02

1.81E+02

1.82E+02

1.82E+02

1.82E+02

1.82E+02

1.82E+02

1.83E+02

1.84E+02

1.86E+02

1.87E+02

1.87E+02

1.88E+02

1.88E+02

1.88E+02

1.88E+02

1.88E+02

1.88E+02

C6H6O6

C7H10O5

C6H10N2O4

C8H14O4

C7H14N2O3

C6H14N4O2

C10H22O2

C6H9NO5

C6H13N3O3

C6H8O6

MWS4471

mws0242

Zmyn000155

mws0260

pmf0173

pme2559

pme0008

MWSmce69

Hmfn000531

mws4175

C6H8O6

C6H8O6

Cmsn000131

pmb3101

C5H8N2O5

C7H12O5

C7H12O5

C7H12O5

C10H10O3

C10H10O3

C6H5N5O2

C9H8O4

Zmgn001448

Lmbn001754

Jmzn006005

mws1200

mws0981

mws2212

mws4170

C6H12O6

C6H12O6

C6H12O6

C6H12O6

C6H12O6

C10H12O3

C10H12O3

C10H12O3

C11H16O2

C9H11NO3

C9H11NO3

C9H10O4

C9H10O4

C6H14O6

C6H14O6

C6H14O6

C8H9NO4

5H15NO4P

C11H22O2

C11H9O2N

C11H9NO2

C11H8O3

C9H16O4

C9H16O4

C8H16N2O3

C8H16N2O3

C8H16N2O3

Hmln000297

pmf0139

pmf0138

mws1164

mws0093

Lmbn004847

MWS2070

Cmyp007180

mws0250

MWStz070

Hmcn006267

mws1350

mws0214

mws1155

pme2237

pme2596

pmb1754

mws0752

Hmgp00232

Smcp001137

pmn001492

mws0237

pmn001380

mws0736

pme0122

mws5041

 

Zmjp000182

Zmdp000292

MWSslk080

pme0075

1.89E+02

1.89E+02

1.87E+02

1.88E+02

1.90E+02

1.91E+02

1.91E+02

1.91E+02

1.93E+02

1.91E+02

1.93E+02

1.93E+02

1.93E+02

1.93E+02

1.93E+02

1.95E+02

1.95E+02

1.96E+02

1.95E+02

1.95E+02

1.98E+02

1.97E+02

1.99E+02

1.99E+02

2.03E+02

2.03E+02

2.03E+02

2.06E+02

2.07E+02

2.05E+02

2.05E+02

2.06E+02

2.07E+02

2.07E+02

2.07E+02

2.09E+02

2.09E+02

2.09E+02

2.13E+02

2.17E+02

2.15E+02

2.19E+02

2.20E+02

2.22E+02

2.23E+02

2.23E+02

2.23E+02

7.01E+01

7.01E+01

1.41E+02

1.28E+02

1.27E+02

1.17E+02

1.11E+02

1.11E+02

1.05E+02

8.50E+01

9.11E+01

7.30E+01

1.35E+02

1.34E+02

9.90E+01

8.31E+01

7.91E+01

9.11E+01

7.49E+01

1.36E+02

1.52E+02

1.23E+02

7.90E+01

1.99E+02

7.01E+01

8.30E+01

1.16E+02

7.60E+01

1.61E+02

1.89E+02

1.89E+02

5.80E+01

1.33E+02

1.33E+02

1.92E+02

8.50E+01

8.50E+01

5.90E+01

9.70E+01

1.58E+02

1.69E+02

1.60E+02

2.02E+02

8.41E+01

1.20E+02

1.20E+02

1.49E+02

1.88E+02

1.88E+02

1.88E+02

1.89E+02

1.89E+02

1.90E+02

1.92E+02

1.92E+02

1.92E+02

1.92E+02

1.92E+02

1.94E+02

1.94E+02

1.94E+02

1.94E+02

1.94E+02

1.94E+02

1.95E+02

1.96E+02

1.96E+02

1.97E+02

1.98E+02

2.00E+02

2.00E+02

2.02E+02

2.02E+02

2.04E+02

2.05E+02

2.06E+02

2.06E+02

2.06E+02

2.07E+02

2.08E+02

2.08E+02

2.08E+02

2.10E+02

2.10E+02

2.10E+02

2.14E+02

2.16E+02

2.16E+02

2.18E+02

2.19E+02

2.21E+02

2.22E+02

2.22E+02

2.22E+02

C7H16N4O2

C7H16N4O2

C10H20O3

C7H11NO5

C7H15N3O3

C12H14O2

C6H8O7

mws0193

mws1611

mws0281

Zmyn000453

Cmpp003619

mws0277

C6H8O7

C10H8O4

C7H12O6

MWSmce41

pme3705

C12H16O2

C6H10O7

Lmdn003756

mws0014

C10H10O4

C10H10O4

C7H14O6

mws2104

mws2218

C8H10N4O2

C12H18O2

C10H13NO3

C6H12O7

pmp001235

MWS1771

pme0534

MWS20182

pme3827

C10H12O4

C9H11NO4

C9H10O5

mws0027

Zmzn000079

pmb2640

C4H9O7P

C12H24O2

C8H18N4O2

C10H26N4

11H12N2O

C9H19NO4

C12H14O3

C14H22O

NK10251888

mws0018

mws0282

pme1261

MWSmce57

Lmln010063

Lmbn013410

Zmgn002106

pmb2620

C14H22O

C11H13NO3

C11H12O4

C11H12O4

C11H12O4

C6H10O8

MWSmce08

pme3443

Zmyn000108

Zmpn000199

Hmcn000192

pmb2507

C6H10O8

C7H14O7

C5H11O7P

C8H16N4O3

C12H24O3

12H14N2O

C9H17NO5

C8H15NO6

11H14N2O

11H14N2O

C13H18O3

pme0170

mws5045

mws0677

mws1337

mws2608

MWS4296

mws5042

Hmgp00595

 

Zmln002252

mws4085

2.23E+02

2.23E+02

2.07E+02

2.29E+02

2.27E+02

2.31E+02

2.35E+02

2.37E+02

2.37E+02

2.39E+02

2.39E+02

2.42E+02

2.43E+02

2.41E+02

2.41E+02

2.44E+02

2.44E+02

2.43E+02

2.45E+02

2.43E+02

2.45E+02

2.45E+02

2.47E+02

2.49E+02

2.51E+02

2.52E+02

2.53E+02

2.53E+02

2.53E+02

2.56E+02

2.55E+02

2.58E+02

2.57E+02

2.58E+02

2.59E+02

2.59E+02

2.59E+02

2.61E+02

2.61E+02

2.65E+02

2.63E+02

2.65E+02

1.33E+02

2.68E+02

2.68E+02

2.68E+02

2.67E+02

1.49E+02

1.93E+02

1.19E+02

7.01E+01

2.27E+02

7.21E+01

1.18E+02

1.35E+02

8.70E+01

2.23E+02

2.23E+02

1.26E+02

1.27E+02

2.41E+02

2.41E+02

1.12E+02

1.12E+02

1.10E+02

1.13E+02

1.81E+02

8.61E+01

2.03E+02

1.84E+02

1.28E+02

1.47E+02

1.36E+02

1.37E+02

2.35E+02

2.35E+02

1.24E+02

2.37E+02

1.04E+02

1.95E+02

7.90E+01

9.70E+01

9.70E+01

9.70E+01

8.61E+01

7.90E+01

1.03E+02

2.04E+02

1.77E+02

1.15E+02

1.52E+02

1.36E+02

1.36E+02

1.35E+02

2.24E+02

2.24E+02

2.24E+02

2.28E+02

2.28E+02

2.30E+02

2.34E+02

2.36E+02

2.38E+02

2.40E+02

2.40E+02

2.41E+02

2.42E+02

2.42E+02

2.42E+02

2.43E+02

2.43E+02

2.44E+02

2.44E+02

2.44E+02

2.44E+02

2.46E+02

2.46E+02

2.50E+02

2.50E+02

2.51E+02

2.52E+02

2.54E+02

2.54E+02

2.56E+02

2.56E+02

2.57E+02

2.58E+02

2.59E+02

2.60E+02

2.60E+02

2.60E+02

2.60E+02

2.62E+02

2.64E+02

2.64E+02

2.64E+02

2.66E+02

2.67E+02

2.67E+02

2.67E+02

2.68E+02

C10H8O6

C11H12O5

C12H16O4

11H20N2O

C14H28O2

11H22N2O

10H22N2O

C16H12O2

C8H14O8

pmp000253

Lmhp001461

mws0119

Lmhp001670

pmb0962

MWSHY020

Lmyn000160

MWS0552

pme2827

C15H28O2

C16H32O

MWS2910

pme0264

10H15N3O

10H14N2O

C15H30O2

C15H30O2

C9H13N3O5

C9H13N3O5

C9H12N2O6

C9H12N2O6

C13H24O4

12H24N2O

13H14N2O

10H18N2O

8H14N2O5

13H18N2O

10H13N5O

10H12N4O

C16H30O2

C16H30O2

11H14NO6

C16H32O2

C8H20NO6P

C14H26O4

C6H14NO8P

C6H13O9P

C6H13O9P

C6H13O9P

11H20N2O

C6H15O9P

C10H16O8

C15H20O4

14H20N2O

C17H30O2

10H13N5O

10H13N5O

10H13N5O

10H12N4O

MWS2430

mws0359

ML1018052

pme3732

mws0248

MWSmce29

MWS5231

Lmhp002031

pmb2591

Zmdp001647

pme2563

Lmhp002153

pme3961

pme2776

Lmcn009122

mws0361

pma3101

mws1488

mws0120

MWSslk133

Zmyn000110

MWS2442

mws1090

mws0866

Zmdp002216

Zmpn000095

Hmjp000461

Lmtn004049

Lmlp003161

Hmsn000210

pme1184

pme0230

Zmjp000966

mws1060

 

pme1119

pmp000087

mws0383

2.69E+02

2.69E+02

2.67E+02

2.71E+02

2.69E+02

2.69E+02

2.71E+02

2.71E+02

2.71E+02

2.71E+02

2.75E+02

2.74E+02

2.77E+02

2.75E+02

2.77E+02

2.79E+02

2.79E+02

2.79E+02

2.79E+02

2.79E+02

2.77E+02

2.79E+02

2.77E+02

2.77E+02

2.79E+02

2.79E+02

2.81E+02

2.81E+02

2.79E+02

2.82E+02

2.82E+02

2.82E+02

2.81E+02

2.84E+02

2.84E+02

2.84E+02

2.83E+02

2.83E+02

2.85E+02

2.85E+02

2.85E+02

2.87E+02

2.89E+02

2.93E+02

2.93E+02

2.93E+02

2.93E+02

1.37E+02

1.77E+02

2.67E+02

1.53E+02

1.51E+02

2.09E+02

1.51E+02

1.51E+02

1.08E+02

2.25E+02

2.13E+02

2.56E+02

1.20E+02

2.57E+02

9.31E+01

1.21E+02

1.49E+02

1.49E+02

1.49E+02

1.20E+02

5.90E+01

9.51E+01

2.77E+02

5.90E+01

1.49E+02

1.63E+02

1.66E+02

1.77E+02

2.79E+02

1.50E+02

1.36E+02

2.47E+02

2.81E+02

1.52E+02

1.52E+02

1.02E+02

1.51E+02

2.83E+02

1.23E+02

2.23E+02

2.39E+02

9.72E+01

9.71E+01

9.51E+01

1.64E+02

1.85E+02

1.13E+02

2.68E+02

2.68E+02

2.68E+02

2.70E+02

2.70E+02

2.70E+02

2.72E+02

2.72E+02

2.72E+02

2.72E+02

2.73E+02

2.73E+02

2.76E+02

2.76E+02

2.76E+02

2.78E+02

2.78E+02

2.78E+02

2.78E+02

2.78E+02

2.78E+02

2.78E+02

2.78E+02

2.78E+02

2.78E+02

2.80E+02

2.80E+02

2.80E+02

2.80E+02

2.81E+02

2.81E+02

2.81E+02

2.82E+02

2.83E+02

2.83E+02

2.83E+02

2.84E+02

2.84E+02

2.86E+02

2.86E+02

2.86E+02

2.86E+02

2.90E+02

2.92E+02

2.94E+02

2.94E+02

2.94E+02

10H12N4O

C13H16O6

C17H32O2

C15H10O5

C15H10O5

C18H38O

MWS20151

Lmyn006227

pmf0293

mws0914

C15H12O5

C15H12O5

C12H16O7

C16H32O3

C16H35NO2

C16H35NO2

14H16N2O

11H20N2O

C18H28O2

C16H22O4

C16H22O4

C16H22O4

C16H22O4

15H22N2O

C18H30O2

C18H30O2

C18H30O2

C18H30O2

C18H30O2

C13H12O7

13H16N2O

14H20N2O

C18H32O2

11H15N5O

11H15N5O

C18H35NO

C18H34O2

10H13N5O

10H13N5O

C18H37NO

10H12N4O

C18H36O2

C13H18O7

C16H30O4

C17H34O3

C18H38O2

10H18N4O

C19H32O2

14H18N2O

C18H30O3

C18H30O3

pme0376

MWSmce67

Lmmn00332

pmb1452

pmp001264

Lmrj002793

pme1712

pma0461

pmp001230

Lmlp012720

Lmxp011770

MWSmce34

mws5035

Lmbn005923

pmb0889

mws0367

MWSmce69

pmb1650

Lmhn002926

mws0629

Lmhp002501

mws1491

MWS4354

Hmhp00181

YC512118

mws2623

MWSmce33

pme1178

Zmzp005934

mws0668

mws1489

pmn001511

MWSslk132

Zmyn005252

mws1436

pmb2657

MWSmce68

Zmdn001564

Zmyn004732

Zmyn004676

 

pmb2787

Zmpn003368

pmb2792

2.93E+02

2.93E+02

2.93E+02

2.96E+02

2.95E+02

2.97E+02

2.95E+02

2.95E+02

2.95E+02

2.98E+02

2.98E+02

2.99E+02

2.97E+02

2.97E+02

2.99E+02

3.01E+02

3.01E+02

3.01E+02

3.01E+02

2.99E+02

2.99E+02

2.99E+02

2.99E+02

2.99E+02

2.99E+02

2.99E+02

2.99E+02

3.03E+02

3.01E+02

3.01E+02

3.01E+02

3.03E+02

3.04E+02

3.03E+02

3.06E+02

3.07E+02

3.07E+02

3.08E+02

3.09E+02

3.09E+02

3.11E+02

3.09E+02

3.09E+02

3.09E+02

3.12E+02

3.12E+02

3.12E+02

2.75E+02

1.95E+02

1.93E+02

1.04E+02

1.63E+02

8.11E+01

1.95E+02

1.71E+02

1.95E+02

1.36E+02

1.66E+02

1.31E+02

1.83E+02

1.83E+02

2.84E+02

2.86E+02

2.86E+02

2.86E+02

2.86E+02

1.37E+02

1.37E+02

1.37E+02

2.23E+02

2.53E+02

2.53E+02

2.53E+02

2.99E+02

2.57E+02

1.51E+02

1.23E+02

1.39E+02

5.71E+01

1.85E+02

2.59E+02

1.43E+02

1.45E+02

3.07E+02

1.46E+02

1.93E+02

2.09E+02

1.65E+02

2.27E+02

1.71E+02

3.09E+02

1.32E+02

1.80E+02

1.80E+02

2.94E+02

2.94E+02

2.94E+02

2.95E+02

2.96E+02

2.96E+02

2.96E+02

2.96E+02

2.96E+02

2.97E+02

2.97E+02

2.98E+02

2.98E+02

2.98E+02

3.00E+02

3.00E+02

3.00E+02

3.00E+02

3.00E+02

3.00E+02

3.00E+02

3.00E+02

3.00E+02

3.00E+02

3.00E+02

3.00E+02

3.00E+02

3.02E+02

3.02E+02

3.02E+02

3.02E+02

3.02E+02

3.03E+02

3.04E+02

3.07E+02

3.08E+02

3.08E+02

3.09E+02

3.10E+02

3.10E+02

3.10E+02

3.10E+02

3.10E+02

3.10E+02

3.11E+02

3.11E+02

3.11E+02

C18H30O3

C18H30O3

C18H30O3

8H14N3O7

C13H12O8

C18H32O3

C18H32O3

C18H32O3

C18H32O3

11H15N5O3

11H15N5O

C19H22O3

C18H34O3

C20H42O

Lmqp000329

pmn001687

Hmqp00541

Rfmb091

Lmbn005662

Lmbn005369

pme1474

pmb0197

Lmfp007216

Zmyn004714

pmf0297

Zmhn003257

MWSHY006

MWSHY000

MWS20189

Lmjp004941

Zmhn001926

Hmtn001302

pmb3142

C16H12O6

C16H12O6

C16H12O6

C16H12O6

C16H12O6

C13H16O8

C13H16O8

C13H16O8

C17H16O5

C18H36O3

C18H36O3

C18H36O3

C18H36O3

C15H10O7

C15H10O7

C13H18O8

C13H18O8

C17H34O4

12H21N3O

C20H32O2

10H17N3O6

11H20N2O

C20H36O2

C11H19NO9

C14H14O8

C18H30O4

14H18N2O

C18H30O4

C18H30O4

C20H38O2

C14H17NO7

12H17N5O

12H17N5O

pmc1990

MWS5235

Zmyn005384

MWS4295

Lmqn008288

Zmpp002571

pme3514

Hmqn00084

Zmhn000892

MWSmce54

mws1375

mws0369

pme1086

pmb2855

pmn001610

pmb2857

Lmhn003074

pmb2791

Zmdp001857

Zmzn003953

Zmyn004449

pmn001606

Lmap001823

pme3967

Lmcp002302

 

Lmhn001477

Lmqp002761

MWS80006

Lmbn005287

pmb2804

3.11E+02

3.13E+02

3.11E+02

3.11E+02

3.11E+02

3.11E+02

3.13E+02

3.13E+02

3.13E+02

3.13E+02

3.17E+02

3.17E+02

3.15E+02

3.15E+02

3.17E+02

3.15E+02

3.15E+02

3.15E+02

3.15E+02

3.18E+02

3.19E+02

3.19E+02

3.21E+02

3.22E+02

3.22E+02

3.23E+02

3.26E+02

3.25E+02

3.25E+02

3.25E+02

3.25E+02

3.25E+02

3.25E+02

3.25E+02

3.25E+02

3.27E+02

3.28E+02

3.27E+02

3.29E+02

3.28E+02

3.31E+02

3.31E+02

3.29E+02

3.31E+02

3.29E+02

3.29E+02

3.31E+02

1.79E+02

1.51E+02

2.93E+02

2.49E+02

1.71E+02

2.23E+02

1.07E+02

1.01E+02

2.51E+02

1.83E+02

3.02E+02

3.02E+02

1.53E+02

1.53E+02

1.53E+02

1.53E+02

1.53E+02

1.53E+02

1.35E+02

6.01E+01

1.63E+02

3.01E+02

1.37E+02

1.30E+02

1.30E+02

2.11E+02

6.21E+01

1.93E+02

1.93E+02

1.19E+02

1.63E+02

1.63E+02

1.19E+02

5.90E+01

1.45E+02

1.88E+02

3.10E+02

1.65E+02

1.67E+02

1.34E+02

3.16E+02

3.15E+02

1.67E+02

1.69E+02

1.67E+02

1.67E+02

3.16E+02

3.12E+02

3.12E+02

3.12E+02

3.12E+02

3.12E+02

3.12E+02

3.14E+02

3.14E+02

3.14E+02

3.14E+02

3.16E+02

3.16E+02

3.16E+02

3.16E+02

3.16E+02

3.16E+02

3.16E+02

3.16E+02

3.16E+02

3.17E+02

3.18E+02

3.20E+02

3.20E+02

3.21E+02

3.23E+02

3.24E+02

3.25E+02

3.26E+02

3.26E+02

3.26E+02

3.26E+02

3.26E+02

3.26E+02

3.26E+02

3.26E+02

3.26E+02

3.27E+02

3.28E+02

3.28E+02

3.29E+02

3.30E+02

3.30E+02

3.30E+02

3.30E+02

3.30E+02

3.30E+02

3.30E+02

C13H12O9

C19H20O4

C18H32O4

C18H32O4

C18H32O4

C18H32O4

C14H18O8

C14H18O8

C18H34O4

C18H34O4

C16H12O7

C16H12O7

C13H16O9

C13H16O9

C13H16O9

C14H20O8

C14H20O8

C14H20O8

C14H20O8

C18H39NO3

C19H26O4

C15H12O8

C20H16O4

11H19N3O6

C18H29NO4

9H13N2O9

C20H39NO2

C14H14O9

C18H14O6

C15H18O8

C15H18O8

C15H18O8

C15H18O8

C12H22O10

C16H22O7

C20H22O4

C15H21NO7

C15H20O8

C16H24O7

10H12N5O6

C17H14O7

C17H14O7

C14H18O9

C14H18O9

C14H18O9

C15H22O8

C19H22O5

Lmbn004685

Lmmn00217

Lmyn002403

Lmbn005487

Lmbn007891

pmp000003

Zmzp004111

pmn001367

pmb2871

Lmfp001509

Hmtn001120

pmn001553

Zmtn001661

mwn002172

pmb2221

Jmzp010766

mws0744

MWSslk214

mws0582

pme2074

pme3188

mws0983

Lmhn002140

Zmdn001863

Hmhn00306

pmn001419

Zmhn002301

Zmzn001549

Lmqn000351

Lmsn000363

MWSmce19

Smcp000882

Hmsn002272

pmp001044

mws0884

pmp000004

Lmzp004885

Smhn102346

Lmmp00308

Smhn002346

pmn001517

Zmhp002496

 

pmn001694

pmp001284

pme3184

3.29E+02

3.31E+02

3.32E+02

3.32E+02

3.33E+02

3.31E+02

3.31E+02

3.31E+02

3.31E+02

3.31E+02

3.31E+02

3.33E+02

3.33E+02

3.37E+02

3.37E+02

3.37E+02

3.37E+02

3.39E+02

3.39E+02

3.39E+02

3.41E+02

3.39E+02

3.39E+02

3.41E+02

3.41E+02

3.41E+02

3.41E+02

3.41E+02

3.41E+02

3.41E+02

3.41E+02

3.41E+02

3.41E+02

3.41E+02

3.41E+02

3.43E+02

3.43E+02

3.43E+02

3.43E+02

3.43E+02

3.43E+02

3.44E+02

3.45E+02

3.48E+02

3.49E+02

3.53E+02

3.53E+02

2.29E+02

3.13E+02

1.36E+02

1.52E+02

1.37E+02

1.69E+02

1.69E+02

1.69E+02

1.69E+02

1.53E+02

1.53E+02

1.67E+02

3.33E+02

1.63E+02

1.91E+02

1.91E+02

1.61E+02

7.90E+01

1.77E+02

1.77E+02

2.07E+02

1.59E+02

1.83E+02

1.79E+02

1.35E+02

1.61E+02

1.79E+02

8.90E+01

8.90E+01

1.13E+02

1.79E+02

1.19E+02

1.19E+02

8.90E+01

1.79E+02

1.37E+02

3.28E+02

1.81E+02

1.81E+02

8.90E+01

3.28E+02

1.50E+02

1.01E+02

1.36E+02

1.37E+02

2.61E+02

2.61E+02

3.30E+02

3.30E+02

3.31E+02

3.31E+02

3.32E+02

3.32E+02

3.32E+02

3.32E+02

3.32E+02

3.32E+02

3.32E+02

3.32E+02

3.34E+02

3.38E+02

3.38E+02

3.38E+02

3.38E+02

3.40E+02

3.40E+02

3.40E+02

3.40E+02

3.40E+02

3.40E+02

3.42E+02

3.42E+02

3.42E+02

3.42E+02

3.42E+02

3.42E+02

3.42E+02

3.42E+02

3.42E+02

3.42E+02

3.42E+02

3.42E+02

3.42E+02

3.44E+02

3.44E+02

3.44E+02

3.44E+02

3.44E+02

3.45E+02

3.46E+02

3.47E+02

3.48E+02

3.52E+02

3.52E+02

C18H34O5

C19H38O4

10H14N5O6

C14H21NO8

10H13N4O7

C13H16O10

C13H16O10

C13H16O10

C13H16O10

C14H20O9

C14H20O9

C18H20O6

C9H19O11P

C16H18O8

C16H18O8

C16H18O8

C16H18O8

C6H14O12P

C15H16O9

C15H16O9

C15H16O9

C16H20O8

C22H44O2

C15H18O9

C15H18O9

C15H18O9

C15H18O9

C12H22O11

C12H22O11

C12H22O11

C12H22O11

C12H22O11

C12H22O11

C12H22O11

C16H22O8

C20H22O5

C18H16O7

C15H20O9

C15H20O9

C12H24O11

C20H24O5

10H12N5O7

C16H26O8

10H14N5O7

10H13N4O8

C21H36O4

C21H36O4

pmb0789

pmc0066

Lmfn000604

pmn001518

Zmhn001703

Hmln000659

mwn002117

Lmmn00129

Lmjp002782

Lmqn000432

pma6460

pmb3074

pmn001421

pmb4777

Zmyn000083

Lmbn001162

mws1015

pma0149

Hmcn004128

pmf0400

pmn001420

Lman002731

mwn002620

Zmhn001793

MA1003964

Lmsn000381

mws1333

mws1080

mws0264

pme0519

mws5038

mws0906

Jmzp009876

MWSslk092

Lmmn00077

Zmln000899

MWS1983

Lmmn00468

mws0609

Cmrn002988

pmb0981

pmb0532

Lmhp011562

Lmhp011388

 

pme1816

mws2108

3.53E+02

3.53E+02

3.53E+02

3.53E+02

3.55E+02

3.55E+02

3.55E+02

3.55E+02

3.55E+02

3.55E+02

3.57E+02

3.59E+02

3.69E+02

3.67E+02

3.69E+02

3.69E+02

3.71E+02

3.73E+02

3.75E+02

3.75E+02

3.75E+02

3.77E+02

3.77E+02

3.83E+02

3.84E+02

3.85E+02

3.87E+02

3.87E+02

3.87E+02

3.87E+02

3.89E+02

3.91E+02

3.91E+02

3.99E+02

4.00E+02

4.03E+02

4.01E+02

4.01E+02

4.06E+02

4.07E+02

4.13E+02

4.19E+02

4.21E+02

4.25E+02

4.26E+02

4.26E+02

4.27E+02

1.91E+02

1.91E+02

1.91E+02

1.91E+02

1.35E+02

2.63E+02

2.63E+02

2.23E+02

1.93E+02

1.93E+02

2.65E+02

1.82E+02

1.77E+02

1.91E+02

1.77E+02

1.77E+02

1.91E+02

1.37E+02

1.77E+02

1.37E+02

1.35E+02

2.43E+02

3.29E+02

1.63E+02

2.52E+02

2.23E+02

1.51E+02

1.51E+02

2.25E+02

2.07E+02

1.91E+02

1.49E+02

5.90E+01

2.37E+02

1.36E+02

3.73E+02

2.69E+02

2.69E+02

1.51E+02

1.77E+02

1.36E+02

2.87E+02

2.41E+02

1.77E+02

2.85E+02

2.85E+02

2.95E+02

3.54E+02

3.54E+02

3.54E+02

3.54E+02

3.54E+02

3.54E+02

3.54E+02

3.56E+02

3.56E+02

3.56E+02

3.56E+02

3.60E+02

3.68E+02

3.68E+02

3.68E+02

3.68E+02

3.70E+02

3.72E+02

3.74E+02

3.74E+02

3.76E+02

3.76E+02

3.78E+02

3.82E+02

3.83E+02

3.86E+02

3.86E+02

3.86E+02

3.88E+02

3.88E+02

3.88E+02

3.90E+02

3.92E+02

3.98E+02

3.99E+02

4.02E+02

4.02E+02

4.02E+02

4.05E+02

4.06E+02

4.12E+02

4.18E+02

4.22E+02

4.24E+02

4.25E+02

4.25E+02

4.26E+02

C16H18O9

C16H18O9

C16H18O9

C16H18O9

C20H18O6

C21H38O4

C21H38O4

C15H16O10

C16H20O9

C20H20O6

C21H40O4

C15H20O10

C16H16O10

C17H20O9

C17H20O9

C17H20O9

C19H30O7

C17H24O9

C16H22O10

C20H22O7

C16H24O10

17H20N4O

C20H26O7

C24H30O4

14H17N5O

C17H22O10

C22H26O6

C22H26O6

C17H24O10

C18H28O9

C19H32O8

C24H38O4

C20H24O8

C19H26O9

15H21N5O

C21H22O8

C18H26O10

C18H26O10

C21H27NO7

C20H22O9

C19H24O10

C20H18O10

C12H23O14

24H28N2O

19H40NO7

19H40NO7

C17H14O13

mws0748

mws0178

pmd0209

Lmhp112042

Lmhp012042

Lmhn001580

Zmhn002422

Lmdn009099

pmb0296

pmb3107

Lmgp003270

mws0179

pmb0752

pma3724

Lmmp00438

Lmap001661

pmp000695

Hmlp002744

Lmzn001983

mws0232

HJN012

Hmap010529

pme3337

Zmhn002227

Lmzp006972

Smgp009046

Hmcn001415

Lmzn001582

Zmjp003597

Lmwp01119

Cmmn00523

Zmjp004455

pmc0281

mws0043

Hmln003529

Cmjn004337

Hmlp003142

Cmdp003077

Hmhp00258

Lmbp003668

mws2523

Lmhp005784

Lmhp008337

pmb0864

mad1424

 

pme2117

Cmbn003996

mws0895

4.26E+02

4.29E+02

4.31E+02

4.31E+02

4.31E+02

4.31E+02

4.31E+02

4.31E+02

4.35E+02

4.35E+02

4.35E+02

4.33E+02

4.35E+02

4.40E+02

4.40E+02

4.40E+02

4.41E+02

4.43E+02

4.41E+02

4.43E+02

4.48E+02

4.49E+02

4.49E+02

4.49E+02

4.49E+02

4.49E+02

4.49E+02

4.49E+02

4.49E+02

4.49E+02

4.51E+02

4.52E+02

4.52E+02

4.51E+02

4.54E+02

4.54E+02

5.21E+02

4.53E+02

4.53E+02

4.53E+02

4.53E+02

4.55E+02

4.53E+02

4.55E+02

4.55E+02

4.55E+02

4.58E+02

1.59E+02

1.93E+02

2.69E+02

2.85E+02

2.69E+02

2.69E+02

2.69E+02

1.49E+02

3.03E+02

3.03E+02

3.03E+02

2.71E+02

3.15E+02

1.84E+02

2.99E+02

2.99E+02

1.77E+02

1.23E+02

2.89E+02

1.47E+02

2.86E+02

3.03E+02

2.87E+02

2.87E+02

2.87E+02

2.87E+02

2.69E+02

2.87E+02

2.87E+02

2.87E+02

8.91E+01

3.11E+02

3.11E+02

3.91E+02

3.13E+02

3.13E+02

2.09E+02

4.53E+02

4.53E+02

4.53E+02

4.53E+02

9.70E+01

4.53E+02

4.55E+02

4.55E+02

4.55E+02

1.16E+02

4.27E+02

4.30E+02

4.32E+02

4.32E+02

4.32E+02

4.32E+02

4.32E+02

4.32E+02

4.34E+02

4.34E+02

4.34E+02

4.34E+02

4.36E+02

4.39E+02

4.39E+02

4.39E+02

4.40E+02

4.42E+02

4.42E+02

4.42E+02

4.47E+02

4.48E+02

4.48E+02

4.48E+02

4.48E+02

4.48E+02

4.50E+02

4.50E+02

4.50E+02

4.50E+02

4.50E+02

4.51E+02

4.51E+02

4.52E+02

4.53E+02

4.53E+02

5.20E+02

4.54E+02

4.54E+02

4.54E+02

4.54E+02

4.56E+02

4.54E+02

4.56E+02

4.56E+02

4.56E+02

4.57E+02

0H15N5O10

C19H26O11

C21H20O10

C21H20O10

C21H20O10

C19H28O11

C19H28O11

C19H28O11

C20H18O11

C20H18O11

C20H18O11

C21H22O10

C21H24O10

20H42NO7

20H42NO7

20H42NO7

24H28N2O

C22H18O10

C22H18O10

C20H26O11

C23H29NO8

C21H20O11

C21H20O11

C21H20O11

C21H20O11

C21H20O11

C21H22O11

C21H22O11

C21H22O11

C21H22O11

C31H46O2

21H42NO7

21H42NO7

C20H20O12

21H44NO7

21H44NO7

C22H32O14

C30H46O3

C30H46O3

C30H46O3

C30H46O3

17H21N4O9

C30H46O3

C30H48O3

C30H48O3

C30H48O3

16H27NO1

mws0919

pmb3006

Lmtn002324

Lmyn003028

Lmjn002534

Lmfp004055

Lmdp003509

mws2186

Cmzn005251

Lmfn004093

pmb0862

Lmhp008885

Lmhp009187

Lmhp005550

MWSHY002

Lmhn004960

pmp000090

Lmlp001118

mws0045

pme2459

Hmpp00327

Xmyp00565

MWSHY013

Jmgn004021

HJN086

pmb3023

Lmtn002796

MWSmce67

Lmhp008763

Lmhp009034

Li512113

pmb0876

pmd0160

pmp001059

MWSmce13

Lmqn012798

Lmzn206829

Lmzn006829

MWS5083

Lmzn006795

mws4053

pmn001700

Zmjn014062

pmb0464

 

mws0034

mws2220

4.57E+02

4.57E+02

4.60E+02

4.63E+02

4.63E+02

4.61E+02

4.63E+02

4.63E+02

4.65E+02

4.63E+02

4.65E+02

4.63E+02

4.63E+02

4.63E+02

4.63E+02

4.66E+02

4.65E+02

4.68E+02

4.68E+02

4.55E+02

4.69E+02

4.69E+02

4.71E+02

4.69E+02

4.71E+02

4.71E+02

4.71E+02

4.71E+02

4.71E+02

4.71E+02

4.71E+02

4.71E+02

4.71E+02

4.74E+02

4.74E+02

4.73E+02

4.76E+02

4.76E+02

4.75E+02

4.77E+02

4.78E+02

4.78E+02

4.77E+02

4.79E+02

4.79E+02

4.79E+02

4.77E+02

1.69E+02

1.69E+02

1.18E+02

3.01E+02

3.01E+02

3.15E+02

3.01E+02

3.01E+02

3.03E+02

3.00E+02

3.03E+02

3.01E+02

3.01E+02

3.00E+02

3.01E+02

3.25E+02

3.11E+02

3.27E+02

1.84E+02

4.55E+02

4.23E+02

4.69E+02

2.77E+02

4.23E+02

4.71E+02

4.71E+02

4.71E+02

4.71E+02

4.71E+02

4.71E+02

4.71E+02

4.71E+02

4.71E+02

3.27E+02

3.33E+02

1.49E+02

3.35E+02

3.35E+02

1.79E+02

3.15E+02

3.37E+02

3.37E+02

1.69E+02

3.17E+02

3.17E+02

3.17E+02

1.69E+02

4.58E+02

4.58E+02

4.61E+02

4.62E+02

4.62E+02

4.62E+02

4.62E+02

4.62E+02

4.64E+02

4.64E+02

4.64E+02

4.64E+02

4.64E+02

4.64E+02

4.64E+02

4.65E+02

4.66E+02

4.67E+02

4.67E+02

4.56E+02

4.70E+02

4.70E+02

4.72E+02

4.70E+02

4.72E+02

4.72E+02

4.72E+02

4.72E+02

4.72E+02

4.72E+02

4.72E+02

4.72E+02

4.72E+02

4.73E+02

4.73E+02

4.74E+02

4.75E+02

4.75E+02

4.76E+02

4.77E+02

4.77E+02

4.77E+02

4.78E+02

4.78E+02

4.78E+02

4.78E+02

4.78E+02

C22H18O11

C22H18O11

19H27NO1

C22H22O11

C22H22O11

C22H22O11

C22H22O11

C22H22O11

C21H20O12

C21H20O12

C21H20O12

C21H20O12

C21H20O12

C21H20O12

C22H24O11

22H44NO7

C22H26O11

22H46NO7

22H46NO7

C30H48O3

C30H46O4

C30H46O4

C23H20O11

C30H46O4

C30H48O4

C30H48O4

C30H48O4

C30H48O4

C30H48O4

C30H48O4

C30H48O4

C30H48O4

C30H48O4

20H23N7O

23H40NO7

C22H18O12

23H42NO7

23H42NO7

C21H32O12

C23H25O11

23H44NO7

23H44NO7

C21H18O13

C22H22O12

C22H22O12

C22H22O12

C22H22O12

pmb2654

Cmyp002064

MWSslk013

Lmpn006208

Lmjp003655

Hmgp00218

Lmdp003286

mws0091

pmp001309

mws1329

mws0856

mws0061

pme1598

Lmhp009464

Lman004071

Lmhp010162

pmd0130

HJN110

pmn001704

Hmjn008136

Lmhn003689

Lmsn012627

Zmjn012003

Lmzn106284

Lmzn006284

Smpn011792

Lmzn006169

Zmpn008194

pmn001706

pmn001705

mws1610

pmb1912

Lmhp008233

MWSmce62

Lmhp008801

Lmhp008589

Lmjn002774

pmb0545

pmb0874

pmb0881

Zmhn004909

Lmcp003788

Hmcp002207

Lmjp002906

Lmsn004162

 

Lmjp003044

Lmjp003295

Hmgp00203

Zmhn003046

pmb0856

4.79E+02

4.79E+02

4.79E+02

4.77E+02

4.80E+02

4.80E+02

4.80E+02

4.81E+02

4.81E+02

4.82E+02

4.82E+02

4.82E+02

4.85E+02

4.87E+02

4.87E+02

4.87E+02

4.87E+02

4.87E+02

4.92E+02

4.94E+02

4.94E+02

4.94E+02

4.95E+02

4.93E+02

4.95E+02

4.93E+02

4.96E+02

4.96E+02

5.01E+02

5.03E+02

5.27E+02

5.03E+02

5.03E+02

5.03E+02

5.06E+02

5.06E+02

5.06E+02

5.06E+02

5.06E+02

5.08E+02

5.07E+02

5.10E+02

5.10E+02

5.16E+02

5.17E+02

5.17E+02

5.18E+02

3.17E+02

3.17E+02

3.17E+02

3.37E+02

3.39E+02

3.39E+02

1.84E+02

3.19E+02

3.19E+02

3.41E+02

1.84E+02

1.84E+02

4.53E+02

3.41E+02

4.87E+02

4.69E+02

4.69E+02

4.87E+02

1.84E+02

3.33E+02

1.84E+02

1.84E+02

3.33E+02

1.69E+02

3.33E+02

3.31E+02

1.84E+02

1.84E+02

2.77E+02

3.23E+02

3.65E+02

3.83E+02

1.79E+02

1.79E+02

1.45E+02

3.65E+02

3.65E+02

1.84E+02

1.59E+02

1.84E+02

1.79E+02

1.84E+02

1.84E+02

1.84E+02

2.63E+02

2.63E+02

1.84E+02

4.78E+02

4.78E+02

4.78E+02

4.78E+02

4.79E+02

4.79E+02

4.79E+02

4.80E+02

4.80E+02

4.81E+02

4.81E+02

4.81E+02

4.86E+02

4.88E+02

4.88E+02

4.88E+02

4.88E+02

4.88E+02

4.91E+02

4.93E+02

4.93E+02

4.93E+02

4.94E+02

4.94E+02

4.94E+02

4.94E+02

4.95E+02

4.95E+02

5.02E+02

5.04E+02

5.04E+02

5.04E+02

5.04E+02

5.04E+02

5.05E+02

5.05E+02

5.05E+02

5.05E+02

5.07E+02

5.07E+02

5.08E+02

5.09E+02

5.09E+02

5.15E+02

5.16E+02

5.16E+02

5.17E+02

C22H22O12

C22H22O12

C22H22O12

C23H26O11

23H46NO7

23H46NO7

23H46NO7

C21H20O13

C22H24O12

23H48NO7

23H48NO7

23H48NO7

C31H50O4

C18H32O15

C30H48O5

C30H48O5

C30H48O5

C30H48O5

24H46NO7

20H31NO1

24H48NO7

24H48NO7

C22H22O13

C22H22O13

C22H22O13

C22H22O13

24H50NO7

24H50NO7

C24H22O12

C18H32O16

C18H32O16

C18H32O16

C18H32O16

C18H32O16

25H31NO1

25H48NO7

25H48NO7

25H48NO7

0H16N5O13

25H50NO7

C21H32O14

25H52NO7

25H52NO7

26H46NO7

C27H48O9

C27H48O9

26H48NO7

mws0289

pmb2260

Lmdp002969

Lmtp003948

pmb0883

pmb2319

Lmhp009129

Li512114

Lmmn00021

Smpn009230

Zmpn006881

Hmhn00781

Hmjn003948

pmb0863

pmb0790

pmp001270

Lmhp008833

HJAP005

Lmsn004321

Lmjp003231

Lmtn002997

pmb0855

pmd0132

Lmhn003668

pme0500

MWS0442

MWSmce48

pme2125

mws1589

pmp001141

Lmhp010514

Lmhp010757

Lmhp008718

MWSmce66

Lmhp009590

Hmcn000773

Lmhp010515

pmb2406

Hmqp00623

Lmhp010334

Lmhp010573

pmb0865

 

pmb0854

pmn001458

Hmbn00269

pmp001273

pmp001251

Li512111

5.18E+02

5.17E+02

5.17E+02

5.20E+02

5.20E+02

5.19E+02

9.46E+02

5.21E+02

5.19E+02

5.21E+02

5.22E+02

5.22E+02

5.21E+02

5.21E+02

5.21E+02

5.21E+02

5.21E+02

5.24E+02

5.24E+02

5.23E+02

5.25E+02

5.29E+02

5.29E+02

5.29E+02

5.36E+02

5.35E+02

5.35E+02

5.35E+02

5.35E+02

5.35E+02

5.37E+02

5.44E+02

5.48E+02

5.48E+02

5.47E+02

5.47E+02

5.47E+02

5.50E+02

5.51E+02

5.53E+02

5.61E+02

5.61E+02

5.65E+02

5.65E+02

5.65E+02

5.79E+02

5.81E+02

1.84E+02

1.75E+02

1.75E+02

1.84E+02

1.84E+02

3.15E+02

9.46E+02

1.31E+02

3.57E+02

1.31E+02

1.84E+02

1.84E+02

3.29E+02

3.59E+02

3.59E+02

3.59E+02

3.59E+02

1.84E+02

1.84E+02

3.61E+02

1.79E+02

3.53E+02

1.93E+02

1.93E+02

1.84E+02

3.23E+02

3.73E+02

3.73E+02

1.21E+02

3.73E+02

3.75E+02

1.84E+02

1.84E+02

1.84E+02

2.23E+02

2.23E+02

2.23E+02

1.84E+02

3.89E+02

1.63E+02

3.37E+02

3.23E+02

3.17E+02

3.17E+02

3.23E+02

4.17E+02

2.87E+02

5.17E+02

5.18E+02

5.18E+02

5.19E+02

5.19E+02

5.20E+02

9.47E+02

5.20E+02

5.20E+02

5.20E+02

5.21E+02

5.21E+02

5.22E+02

5.22E+02

5.22E+02

5.22E+02

5.22E+02

5.23E+02

5.23E+02

5.24E+02

5.26E+02

5.30E+02

5.30E+02

5.30E+02

5.35E+02

5.36E+02

5.36E+02

5.36E+02

5.36E+02

5.36E+02

5.38E+02

5.43E+02

5.47E+02

5.47E+02

5.48E+02

5.48E+02

5.48E+02

5.49E+02

5.52E+02

5.52E+02

5.62E+02

5.62E+02

5.64E+02

5.64E+02

5.66E+02

5.80E+02

5.81E+02

26H48NO7

C22H30O14

C22H30O14

26H50NO7

26H50NO7

C24H24O13

C48H82O18

C26H32O11

C26H32O11

C26H32O11

26H52NO7

26H52NO7

C23H22O14

C24H26O13

C26H34O11

C26H34O11

C26H34O11

26H54NO7

26H54NO7

C26H36O11

C25H34O12

C26H26O12

C23H30O14

C23H30O14

27H54NO7

4H22N2O16

C26H32O12

C26H32O12

C26H32O12

C26H32O12

C26H34O12

28H50NO7

28H54NO7

28H54NO7

C23H32O15

C23H32O15

C23H32O15

28H56NO7

C27H36O12

C27H36O12

C26H26O14

C24H34O15

C25H24O15

C25H24O15

5H24N2O17

C28H36O13

C26H29O15

Hmsn004596

Cmsp003083

pmn001378

Lmsp003655

Lmhp010190

pmp001281

pmb3041

pmn001710

Lmmn00227

Lmyn003971

HJN083

mws0126

pmd0136

Lmtn003096

Cmsn001914

Zmcn005156

pmb2554

pmb2833

Lmhp010908

Zmfn000481

Hmln002100

Zmcn003729

Lmtn004500

pmn001375

Lmdn001925

pmc0960

pmd0146

pmd0147

pmn001672

Lmsn003628

pmn001671

Lmhp011549

Lmmn00226

Zmjp003892

Lmhn003802

pmn001469

HJAP064

Zmhp005139

pmb2922

Zmhn001446

Lmjp001877

 

MWSmce45

Zmjp003291

pmp001106

Lmtp004044

Lmqn000780

Lmmp00309

Zmxp003107

Lmbp002592

pmn001583

Hmpp00261

pmp000686

pme0001

5.85E+02

5.95E+02

5.95E+02

5.97E+02

6.06E+02

6.11E+02

6.11E+02

6.11E+02

6.09E+02

6.11E+02

6.11E+02

6.09E+02

6.11E+02

6.11E+02

6.11E+02

6.11E+02

6.13E+02

6.13E+02

6.17E+02

6.19E+02

4.71E+02

6.17E+02

6.21E+02

6.25E+02

6.25E+02

6.25E+02

6.27E+02

6.27E+02

6.27E+02

6.27E+02

6.27E+02

6.21E+02

6.37E+02

6.33E+02

6.39E+02

6.39E+02

6.39E+02

6.39E+02

6.41E+02

6.41E+02

6.41E+02

6.49E+02

6.55E+02

6.39E+02

6.55E+02

6.64E+02

6.65E+02

5.67E+02

4.33E+02

5.77E+02

3.03E+02

3.85E+02

3.03E+02

2.87E+02

2.87E+02

3.00E+02

2.87E+02

1.63E+02

3.01E+02

3.03E+02

2.87E+02

2.87E+02

3.06E+02

2.89E+02

2.89E+02

1.53E+02

1.53E+02

4.71E+02

4.55E+02

4.17E+02

3.17E+02

3.17E+02

3.17E+02

3.03E+02

4.65E+02

3.03E+02

3.03E+02

4.65E+02

4.23E+02

4.75E+02

4.39E+02

4.23E+02

4.23E+02

4.23E+02

4.23E+02

3.17E+02

3.17E+02

3.17E+02

1.45E+02

2.07E+02

4.41E+02

4.39E+02

1.36E+02

1.61E+02

5.84E+02

5.94E+02

5.94E+02

5.96E+02

6.07E+02

6.10E+02

6.10E+02

6.10E+02

6.10E+02

6.10E+02

6.10E+02

6.10E+02

6.11E+02

6.11E+02

6.11E+02

6.12E+02

6.12E+02

6.12E+02

6.16E+02

6.18E+02

4.72E+02

6.18E+02

6.22E+02

6.24E+02

6.24E+02

6.25E+02

6.26E+02

6.26E+02

6.26E+02

6.26E+02

6.26E+02

6.20E+02

6.38E+02

6.32E+02

6.38E+02

6.38E+02

6.38E+02

6.38E+02

6.40E+02

6.40E+02

6.41E+02

6.50E+02

6.54E+02

6.38E+02

6.54E+02

6.63E+02

6.66E+02

32H44N2O

C27H30O15

C27H30O15

C26H28O16

7H27N3O17

C27H30O16

C27H30O16

C27H30O16

C27H30O16

C27H30O16

C28H34O15

C28H34O15

C30H27O14

C30H27O14

C27H31O16

0H32N6O12

C27H32O16

C27H32O16

C28H24O16

C28H26O16

C30H48O4

C36H58O8

C30H38O14

C28H32O16

C28H32O16

C31H29O14

C27H30O17

C27H30O17

C27H30O17

C27H30O17

C28H34O16

C36H60O8

C30H38O15

C36H56O9

C36H62O9

C36H62O9

C36H62O9

C36H62O9

C28H32O17

C28H32O17

C31H29O15

C24H42O20

C32H30O15

C36H62O9

C36H62O10

1H27N7O14

C31H38O16

Lmpp003662

Lmcp004436

Zmcp002924

mws4134

Cmxp003531

Zmgp003335

Lmyp003139

Lmfp003026

Hmbn00520

pmn001506

Rfmb26201

Hmlp004943

Lmhp003217

Lmpp003815

pmp001310

Zmhp003784

Zmhp002730

Lmtp003677

pmb0645

HJAP107

mwn004308

Lmxp06952

Zmjp006798

Zmjp007126

P4321

P4451

Lmhp002800

Hmgp00188

Lmpp003228

pmb2653

pmb0578

Lmzp004417

P4269

pmb0530

mws1153

 

mws4163

Lmqn000213

mws1593

6.65E+02

6.65E+02

6.65E+02

6.77E+02

6.79E+02

6.79E+02

6.79E+02

6.81E+02

6.85E+02

6.85E+02

6.95E+02

6.97E+02

6.97E+02

6.99E+02

6.55E+02

7.53E+02

7.53E+02

7.63E+02

7.67E+02

7.67E+02

7.67E+02

7.71E+02

7.71E+02

7.69E+02

7.99E+02

7.83E+02

7.83E+02

7.84E+02

7.83E+02

7.87E+02

7.87E+02

7.95E+02

8.01E+02

7.93E+02

7.99E+02

7.99E+02

7.99E+02

8.02E+02

8.09E+02

8.09E+02

8.17E+02

8.20E+02

8.30E+02

8.44E+02

8.42E+02

9.00E+02

9.18E+02

4.85E+02

3.41E+02

1.61E+02

6.77E+02

2.63E+02

2.63E+02

2.63E+02

5.19E+02

5.23E+02

5.23E+02

3.53E+02

5.35E+02

5.35E+02

5.37E+02

4.39E+02

4.23E+02

4.23E+02

7.63E+02

4.25E+02

4.25E+02

4.23E+02

2.95E+02

6.09E+02

4.75E+02

4.39E+02

4.23E+02

6.22E+02

4.37E+02

4.75E+02

1.43E+02

4.39E+02

4.39E+02

2.07E+02

6.31E+02

6.37E+02

6.37E+02

6.38E+02

4.39E+02

1.43E+02

4.39E+02

4.39E+02

4.23E+02

7.69E+02

4.39E+02

7.81E+02

4.23E+02

4.07E+02

6.66E+02

6.66E+02

6.66E+02

6.76E+02

6.78E+02

6.78E+02

6.78E+02

6.82E+02

6.86E+02

6.86E+02

6.96E+02

6.98E+02

6.98E+02

7.00E+02

6.54E+02

7.52E+02

7.54E+02

7.64E+02

7.66E+02

7.66E+02

7.66E+02

7.70E+02

7.72E+02

7.70E+02

7.80E+02

7.82E+02

7.84E+02

7.85E+02

7.84E+02

7.86E+02

7.86E+02

7.94E+02

8.00E+02

7.94E+02

8.00E+02

8.00E+02

8.00E+02

8.00E+02

8.08E+02

8.08E+02

8.16E+02

8.19E+02

8.31E+02

8.43E+02

8.43E+02

8.99E+02

9.17E+02

C24H42O21

C24H42O21

C24H42O21

C33H56O14

C33H58O14

C33H58O14

C33H58O14

C32H42O16

C31H42O17

C32H46O16

C31H36O18

C32H42O17

C32H42O17

C32H44O17

C36H62O10

C41H68O12

C41H70O12

C41H64O13

C42H70O12

C42H70O12

C42H70O12

C41H70O13

C36H36O19

C41H70O13

C42H68O13

C42H70O13

C42H72O13

7H33N9O15

C42H72O13

C41H70O14

C41H70O14

C42H66O14

C38H40O19

C42H66O14

C42H72O14

C42H72O14

C42H72O14

C42H72O14

C43H68O14

C43H68O14

C42H72O15

C42H74O15

C43H74O15

C44H74O15

C44H74O15

C47H78O16

C47H80O17

pmp001276

Lmhp009384

Lmhp009190

pmp001271

pmn001370

pmn001743

MWSmce49

Lmgn002253

pmn001369

Hmln101664

pmn001368

P4195

P3643

P4685

pmn001505

Cmrp007847

Cmrp008360

P4881

Lmxp06577

Zmcn004206

mws4038

Hmmp00606

P4501

mws4035

pme2746

mws4023

Lmzp004643

P4045

Lmzp006292

pmb0676

Lmzn004931

mws1553

HJN032

mws1665

Lmxp05341

Lmxp05257

Lmxp05320

P3859

P4308

HJN034

Lmxp06071

HJN033

Zmjp006771

Zmsp007698

 

Hmsn005090

P5367

9.16E+02

9.30E+02

9.32E+02

9.48E+02

9.46E+02

9.48E+02

9.46E+02

9.55E+02

9.56E+02

9.55E+02

9.58E+02

9.58E+02

9.55E+02

9.62E+02

9.62E+02

9.62E+02

9.62E+02

9.71E+02

9.88E+02

1.08E+03

1.09E+03

1.09E+03

1.09E+03

1.09E+03

1.11E+03

1.11E+03

1.11E+03

1.12E+03

1.12E+03

1.15E+03

1.15E+03

1.20E+03

1.21E+03

7.84E+02

4.07E+02

7.99E+02

4.23E+02

7.84E+02

3.25E+02

7.99E+02

7.93E+02

5.69E+02

7.94E+02

4.39E+02

4.39E+02

7.93E+02

9.32E+02

7.99E+02

7.99E+02

8.00E+02

8.09E+02

9.46E+02

1.08E+03

4.39E+02

7.31E+02

4.39E+02

4.39E+02

4.39E+02

1.11E+03

3.25E+02

4.39E+02

1.12E+03

1.11E+03

1.11E+03

4.11E+02

1.15E+03

9.17E+02

9.29E+02

9.33E+02

9.47E+02

9.47E+02

9.47E+02

9.47E+02

9.56E+02

9.56E+02

9.56E+02

9.56E+02

9.56E+02

9.56E+02

9.61E+02

9.63E+02

9.63E+02

9.63E+02

9.72E+02

9.89E+02

1.08E+03

1.09E+03

1.09E+03

1.09E+03

1.09E+03

1.11E+03

1.11E+03

1.11E+03

1.12E+03

1.12E+03

1.15E+03

1.15E+03

1.19E+03

1.21E+03

C47H80O17

C48H80O17

C47H80O18

C48H82O18

C48H82O18

C48H82O18

C48H82O18

C48H76O19

C48H76O19

C48H76O19

C48H76O19

C48H76O19

C48H76O19

C48H82O19

C48H82O19

C48H82O19

C48H82O19

C48H76O20

C50H84O19

C53H90O22

C53H84O23

C53H84O23

C53H84O23

C53H84O23

C54H90O23

C54H92O23

C54H92O23

C54H86O24

C54H92O24

C56H94O24

C56H94O24

C57H94O26

C58H98O26

mws1721

Lmxp04903

mws1666

Lmzp005427

Hmsn003200

Lmxn007617

Zmcn006328

mws1592

Zmcp005976

Zmcp005794

Lmzn004691

Lmxn05454

Lmzn004178

Hmsn002785

Hmsn003015

Lmxn006768

Lmzn005163

Hmsn004349

Zmcp005857

Cmsn001419

Zmcp005775

Zmcp105857

Zmjp006441

Hmsn004091

Lmxp06204

Zmcp002314

Hmsn003355

Lmzn004494

Cmsn001412

P4618

Lmzn004497

 

Table S1 Metabolome information of the widely-targeted m

Ionization model

[M+H]+

[M+H]+

[M+H]+

[M+H]+

[M+H]+

[M-H]-

Compounds

Piperidine

Class I

Class II

CAS

Level

3

3

3

1

3

3

3

3

3

3

3

1

3

1

2

1

3

1

1

3

3

1

3

3

3

3

3

1

3

3

3

3

3

3

2

2

1

3

3

3

3

1

1

1

1

YYA1

1531700

4637800

4167000

1444200

36636000

266890

258080

2716100

355200

34854

Alkaloids

Alkaloids 110-89-4

Aminoisobutyric ac Organic acids Organic acid 62-57-7

hyl 3-aminopropamnino acids and derivaticids and de 4138-35-6

,N-Dimethylglycminino acids and derivaticids and de 1118-68-9

Choline

Alkaloids

Alkaloids 62-49-7

Hydroxypyruvic aci Organic acids Organic acid1113-60-6

rtronate semialdehy Organic acids Organic acid2480-77-5

Hydroxybutyric ac Organic acids Organic acid 300-85-6

L-Serine mino acids and derivaticids and de 56-45-1

[M-H]-

[M-H]-

[M+H]+

[M+H]+

[M+H]+

[M+H]+

[M+H]+

[M+H]+

[M-H]-

Diethanolamine

2-Aminophenol

Cytosine

Alkaloids

Alkaloids

Alkaloids 111-42-2

Alkaloids 95-55-6

10976

cleotides and derivatiivdes and der 71-30-7

258140

288350

5659600

205470

22807000

1743100

15874000

16188000

251480

342000

759570

3901600

34442

Isocytosine cleotides and derivatiivdes and der 108-53-2

L-Proline mino acids and derivaticids and de 147-85-3

Fumaric acid

Organic acids Organic acid 110-17-8

[M+H]+

[M+H]+

[M-H]-

L-Valine mino acids and derivaticids and de 72-18-4

Betaine

Alkaloids Alkaloids 107-43-7

Organic acids Organic acid 110-15-6

Succinic acid

[M-H]-

Methylmalonic acid Organic acids Organic acid 516-05-2

Hydroxyisovaleric a Organic acids Organic acid 625-08-1

L-Threonine mino acids and derivaticids and de 72-19-5

[M-H]-

[M+H]+

[M+H]+

[M-H]-

lmethylene isomet

ethylthio)Propionic Organic acids Organic acid 646-01-5

D-(-)-Threose Others rides and A 95-43-2

L-Cysteine mino acids and derivaticids and de 52-90-4

Phenethylamine Alkaloids Alkaloids 64-04-0

Hydroxybenzaldehy Phenolic acids henolic acid 123-08-0

Alkaloids

Alkaloids

-

[M+H]+

[M-H]-

44021

[M+H]+

[M-H]-

79622

175930

1155700

77299

[M+H]+

[M+H]+

[M-H]-

Nicotinamide

tinic acid (Vitamin

2-Picolinic acid

Others

Others

Vitamin 98-92-0

Vitamin 59-67-6

Organic acids Organic acid 98-98-6

Organic acids Organic acid 55-22-1

74109

[M+H]+

[M+H]+

[M-H]-

Isonicotinic acid

idazole-4-Acetic A

84960

Alkaloids

Plumerane 645-65-8

119230

12623

5-Oxo-L-Prolinemino acids and derivaticids and de 98-79-3

5-Oxoproline mino acids and derivaticids and de 149-87-1

[M-H]-

16136

[M+H]+

[M+H]+

[M+H]+

[M-H]-

L-Pipecolic Acid

Cycloleucine mino acids and derivaticids and de 52-52-8

4-Guanidinobutana Others Others

ethylenesuccinic ac Organic acids Organic acid 97-65-4

5-Aminolevulinate Alkaloids Alkaloids 106-60-5

s-4-Hydroxy-L-pmroino acids and derivaticids and de 51-35-4

Creatine Organic acids Organic acid 57-00-1

Organic acids Organic acid3105-95-1

3348800

4938700

5481500

108220

36245

-

[M+H]+

[M+H]+

[M-H]-

6942900

13374

[M+H]+

[M+H]+

[M+H]+

[M+H]+

-Aminocaproic aci Organic acids Organic acid 60-32-2

L-Leucine mino acids and derivaticids and de 61-90-5

1216300

21511000

1766100

22072000

6-Deoxyfagomine eridine alka1l 97449-09-5

Alkaloids

L-Norleucine mino acids and derivaticids and de 327-57-1

 

[M+H]+

[M+H]+

[M-H]-

[M-H]-

[M-H]-

[M-H]-

[M-H]-

[M+H]+

[M-H]-

[M+H]+

[M-H]-

[M-H]-

[M-H]-

[M-H]-

[M+H]+

[M+H]+

[M+H]+

[M-H]-

[M-H]-

[M-H]-

[M+H]+

[M+H]+

[M-H]-

[M-H]-

[M-H]-

[M-H]-

[M-H]-

[M+H]+

[M+H]+

[M+H]+

[M+H]+

[M+H]+

[M]+

L-Isoleucine mino acids and derivaticids and de 73-32-5

L-Allo-isoleucinme ino acids and derivaticids and de 1509-34-8

onomethyl succina Organic acids Organic acid3878-55-5

1

1

1

1

1

1

3

3

3

3

1

1

1

1

3

2

1

1

3

3

3

3

3

3

3

3

3

3

3

1

3

3

3

3

2

3

1

1

1

1

3

1

1

3

1

3

3

22361000

1276900

265340

2938700

329250

320370

29853

Methylsuccinic ac

-2-methyl-3-oxobu Organic acids Organic acid71698-08-3

imethylmalonic ac Organic acids Organic acid 595-46-0

Organic acids Organic acid 498-21-5

Ureidopropionic Ac Organic acids Organic acid 462-88-4

L-Asparagine mino acids and derivaticids and de 70-47-3

Hydroxyhexanoic a Organic acids Organic acid1191-25-9

L-Ornithine mino acids and derivaticids and de 70-26-8

L-Aspartic Acidmino acids and derivaticids and de 56-84-8

Iminodiacetic acid Organic acids Organic acid 142-73-4

24342

53171

139910

5229100

548170

34889000

1236000

186100

671180

172500

282050

54116

hydro-L-Threonic

L-Malic acid

Others

rides and A

-

Organic acids Organic acid 97-67-6

-Methyl-L-cysteimnino acids and derivaticids and de 1187-84-4

Adenine cleotides and derivatiivdes and der 73-24-5

N-benzylformamide

D-Threonic Acid

Alkaloids

Others

Alkaloids 6343-54-0

rides and A 3909-12-4

Hydroxyacetophemnoino acids and derivaticids and de 99-93-4

Phenyl acetate

Salicylamide

Phenolic acids henolic acid 122-79-2

Alkaloids Alkaloids 65-45-2

57854

173490

92953

-Aminobenzoic aci Phenolic acids henolic acid 150-13-0

Hydroxybenzoic A

Salicylic acid

Organic acids Organic acid 99-06-9

Phenolic acids henolic acid 69-72-7

572330

47755

Dihydroxybenzalde Phenolic acids henolic acid1194-98-5

rotocatechualdehyd Phenolic acids henolic acid 139-85-5

147170

197960

109880

4738100

95496

hosphorylethanolam

Histidinol

Alkaloids

Alkaloids

Alkaloids

Alkaloids 1071-23-4

Alkaloids 501-28-0

rrole alkalo 471-87-4

Stachydrine

-Cyclopentylglycminino acids and derivaticids and de 2521-84-8

piperidine-2-carbmo ino acids and derivaticids and de 7730-87-2

1731400

245570

35543

ihydroisopelletierin

Alkaloids

eridine alkal

-

D-Proline betainme ino acids and derivaticids and de 1195-94-4

100480

92976

[M+H]+

[M+H]+

[M+H]+

[M+H]+

[M-H]-

[M+H]+

[M+H]+

[M+H]+

[M+H]+

[M-H]-

[M+H]+

[M+H]+

[M-H]-

[M-H]-

N-Acetylcadaverine

ole-3-carboxaldehy

Alkaloids

Alkaloids

Alkaloids 32343-73-0

Plumerane 487-89-8

34595

-Trans-4-Hydroxmy-ino acids and derivaticids and de 4252-82-8

131580

2624600

255350

3724400

3320900

261410

15817000

1317100

342930

2639900

53762

Guanidinobutyric a

Dimethylsuccinic

Organic acids Organic acid 463-00-3

Organic acids Organic acid 597-43-3

L-Glutamine mino acids and derivaticids and de 56-85-9

L-Lysine mino acids and derivaticids and de 56-87-1

Acetylcholine

Alkaloids

Alkaloids 60-31-1

L-Glutamic acidmino acids and derivaticids and de 56-86-0

hreo-3-Methylaspmarino acids and derivaticids and de 6061-13-8

O-Acetylserinemino acids and derivaticids and de 5147-00-2

Phthalic anhydride Phenolic acids henolic acid 85-44-9

Hydroxyglutaric ac Organic acids Organic acid 638-18-6

L-Citramalic acid

Organic acids Organic acid6236-09-5

86878

 

[M-H]-

[M-H]-

[M-H]-

[M-H]-

[M+H]+

[M+H]+

[M+H]+

[M-H]-

[M-H]-

[M-H]-

[M+H]+

[M+H]+

[M-H]-

[M+H]+

[M-H]-

[M+H]+

[M-H]-

[M+H]+

[M+H]+

[M-H]-

[M-H]-

[M-H]-

[M-H]-

[M-H]-

[M+H]+

[M-H]-

[M-H]-

[M-H]-

[M-H]-

[M-H]-

[M-H]-

[M+H]+

[M-H]-

[M+H]+

[M-H]-

[M-H]-

[M-H]-

[M-H]-

[M+H]+

[M+H]+

[M-H]-

[M+H]+

[M+H]+

[M+H]+

[M+H]+

[M-H]-

[M-H]-

dro-3-deoxy-L-arab

Others

rides and A

-

1

3

3

1

1

1

3

1

1

1

1

1

3

3

3

3

3

1

3

3

3

3

3

3

3

3

3

1

3

3

3

1

3

3

1

3

3

3

3

3

3

1

3

3

3

3

3

127290

133520

113950

63448

3-Methylmalic acid Organic acids Organic acid152204-30-3

Hydroxyglutaric Ac Organic acids Organic acid13095-48-2

Arabinono-1,4-lact

Hydroxymandelonit

Others

rides and A 2782-09-4

Phenolamine13093-65-7

Alkaloids

3702000

3393300

52286

L-Methionine mino acids and derivaticids and de 63-68-3

1-Methyladenine cleotides and derivatiivdes and der5142-22-3

D-Ribose

DL-Xylose

D-Arabinose

Others

Others

Others

rides and A 50-69-1

rides and A25990-60-7

rides and A10323-20-3

167790

175440

181210

30486

Isoguanine cleotides and derivatiivdes and der3373-53-3

Guanine

Xanthine

cleotides and derivatiivdes and der 73-40-5

cleotides and derivatiivdes and der 69-89-6

Phenolic acids henolic acid 621-59-0

Phenolic acids henolic acid 121-33-5

109020

18714

Isovanillin

Vanillin

258990

4159400

38399

cis-Citral

Others Others 5392-40-5

Organic acids Organic acid 303-38-8

Dihydroxybenzoic

198080

1364800

50379

L-Histidine mino acids and derivaticids and de 71-00-1

TranexamicAcid

2-Decanol

Organic acids Organic acid1197-18-8

Others

Others

rides and A 1120-06-5

rides and A 112-30-1

18470000

15340000

140820

24497

1-Decanol

etamidopentanoic

Organic acids Organic acid1072-10-2

-Propylsuccinic aci Organic acids Organic acid 618-57-5

dole-3-carboxylic a

L-2-Aminoadipic ac

Alkaloids

Alkaloids

Plumerane 771-50-6

Alkaloids 542-32-5

20930

60297

-Acetyl-L-threonminino acids and derivaticids and de17093-74-2

3-methylpentane-m1 ino acids and derivaticids and de 503-49-1

32019

125080

232530

780370

70959

-anhydro-β-D-gluc

Others

rides and A 498-07-7

Hydroxycinnamic a Phenolic acids henolic acid 583-17-5

Hydroxycinnamic A Phenolic acids henolic acid14755-02-3

rmylamino)benzoi Phenolic acids henolic acid3342-77-6

L-Phenylalaninemino acids and derivaticids and de 63-91-2

84003

8023400

121480

4401000

2561900

232560

90747

Terephthalic acid

Phenolic acids henolic acid 100-21-0

Methylmercaptopurcleotides and derivatiivdes and der 50-66-8

D-Xylonic acid

L-Fucitol

Others

Others

rides and A 526-91-0

rides and A13074-06-1

ethoxyphenyl)-1-p Phenolic acids henolic acid5349-60-0

Quinolinic Acid

Pyridoxal

Alkaloids

Others

Alkaloids 89-00-9

Vitamin 66-72-8

63851

27914

techuic Acid Meth Phenolic acids henolic acid2150-43-8

Vanillic acid Phenolic acids henolic acid 121-34-6

Gallacetophenone Phenolic acids henolic acid 528-21-2

Pyridoxine Others Vitamin 65-23-6

8666.3

131050

83385

120440

147270

369560

237090

16177

1-Methylhistidinme ino acids and derivaticids and de 332-80-9

-Methyl-L-Histidminino acids and derivaticids and de 368-16-1

droxyacetone phos rides and A1957-04-05

Others

9-Oxononanoic acid Organic acids Organic acid2553-17-5

 

[M-H]-

[M-H]-

[M-H]-

[M-H]-

[M-H]-

[M+H]+

[M-H]-

[M-H]-

[M+H]+

[M-H]-

[M-H]-

[M-H]-

[M-H]-

[M-H]-

[M-H]-

[M-H]-

[M-H]-

[M-H]-

[M-H]-

[M-H]-

[M-H]-

[M-H]-

[M-H]-

[M-H]-

[M-H]-

[M-H]-

[M-H]-

[M-H]-

[M+H]+

[M+H]+

[M+H]+

[M-H]-

[M-H]-

[M-H]-

[M-H]-

[M-H]-

[M+H]+

[M]+

ehydroascorbic aci

Shikimic acid

Others

Vitamin 490-83-5

3

3

3

3

1

1

3

3

1

1

1

2

3

3

3

3

3

3

3

3

1

1

1

1

1

3

3

3

3

1

1

3

3

3

3

3

3

3

3

1

1

3

3

3

3

3

3

109480

55112

Organic acids Organic acid 138-59-0

ha-Acetyl-L-Aspmaino acids and derivaticids and de 4033-40-3

Suberic Acid Organic acids Organic acid 505-48-6

176890

56702

α-Acetyl-L-ornitmhiino acids and derivaticids and de 6205-08-9

L-Arginine mino acids and derivaticids and de 74-79-3

13610000

6961500

85343

1,2-Decanediol

Others

rides and A 1119-86-4

Acetyl-L-AsparticmAino acids and derivaticids and de 997-55-7

L-Citrulline mino acids and derivaticids and de 372-75-8

25287

1596400

179060

720170

46724

bic Acid; Isoascorb

corbic acid (Vitam

Glucurono-6,3-lact

Others

Others

Others

Vitamin 89-65-6

Vitamin 50-81-7

rides and A32449-92-6

Dencichin mino acids and derivaticids and de 5302-45-4

Isopropylmalic Ac Organic acids Organic acid49601-06-1

293970

216730

188480

211370

20770

2-Propylmalic Acid Organic acids Organic acid

-

Isopropylmalic Ac Organic acids Organic acid 921-28-8

ylenedioxy cinnamLignans and Coumarin Lignans 58095-76-4

oxycinnamic Acid Organic acids Organic acid19367-38-5

Isoxanthopterin cleotides and derivatiivdes and der 529-69-1

16807

26178

Caffeic acid

D-Glucose

Phenolic acids henolic acid 331-39-5

84110

Others

Others

Others

Others

Others

rides and A 50-99-7

rides and A 87-89-8

rides and A 59-23-4

rides and A 3458-28-4

rides and A 57-48-7

4447600

4890100

3830700

4895400

4868500

58095

Inositol

D-Galactose

D-Mannose

D-Fructose

Coniferyl alcohol

Phenolic acids henolic acid 458-35-5

hoxyphenylpropion Phenolic acids henolic acid

pyl 4-hydroxybenz Phenolic acids henolic acid 94-13-3

Dihydroactinidiolid Others Others 17092-92-1

L-Tyrosine mino acids and derivaticids and de 60-18-4

xy-4-methoxypheny Alkaloids Phenolamine58469-06-0

-

17707

23234

7986.1

11451000

9580500

6342.7

60788

Dimethoxybenzoic Phenolic acids henolic acid1466-76-8

Hydroxy-3,5-Dime Phenolic acids henolic acid 134-96-3

D-Sorbitol

D-Mannitol

Others

Others

rides and A 50-70-4

rides and A 69-65-8

rides and A 608-66-2

Vitamin 82-82-6

Alkaloids 107-73-3

ree fatty acid112-37-8

53680

14986

Dulcitol

Others

41228

4-Pyridoxic acid

O-Phosphocholine

Undecylic Acid

mino-2-naphthoic a

naphthisoxazol A

Others

77392

Alkaloids

Lipids

286410

636200

5085100

5389800

54883

[M-H]-

[M+H]+

[M+H]+

[M-H]-

[M-H]-

[M-H]-

[M+H]+

[M+H]+

[M+H]+

Alkaloids

Others

Alkaloids

Others

-

-

Ayapin

Lignans and CoumarinCoumarins 494-56-4

Organic acids Organic acid 123-99-9

Azelaic acid

Eucommiol

763200

39208

Others

Others 55930-44-4

N-Glycyl-L-leucimneino acids and derivaticids and de 869-19-2

N6-Acetyl-L-lysimneino acids and derivaticids and de 692-04-6

-Glycyl-L-isoleucminino acids and derivaticids and de19461-38-2

100820

163920

76997

 

[M+H]+

[M+H]+

[M-H]-

[M-H]-

[M+H]+

[M+H]+

[M-H]-

[M-H]-

[M+H]+

[M-H]-

[M+H]+

[M-H]-

[M-H]-

[M-H]-

[M-H]-

[M+H]+

[M+H]+

[M+H]+

[M-H]-

[M-H]-

[M+H]+

[M-H]-

[M-H]-

[M-H]-

[M+H]+

[M+H]+

[M-H]-

[M+H]+

[M+H]+

[M-H]-

[M-H]-

[M-H]-

[M-H]-

[M-H]-

[M-H]-

[M-H]-

[M-H]-

[M-H]-

[M-H]-

[M+H]+

[M-H]-

[M+H]+

[M+H]+

[M+H]+

[M+H]+

[M+H]+

[M+H]+

Monomethyl-L-argmiino acids and derivaticids and de17035-90-4

rginine methyl esmteino acids and derivaticids and de

1

1

3

3

3

2

1

1

2

3

1

1

2

1

3

3

3

3

1

3

3

3

3

1

1

3

1

3

3

1

1

1

3

3

3

1

1

2

3

1

3

3

1

3

3

3

3

9671000

9636700

2513.9

795950

34989

-

Hydroxydecanoic a Organic acids Organic acid1679-53-4

Acetyl-L-glutamicmaino acids and derivaticids and de 1188-37-0

L-Homocitrullinme ino acids and derivaticids and de 1190-49-4

Z-Ligustilide

Citric Acid

Others

Others 4431-01-0

60347

Organic acids Organic acid 77-92-9

Organic acids Organic acid 320-77-4

33514000

17348000

245410

667770

253170

72183

Isocitric Acid

ydroxy-4-methylcLoignans and CoumarinCoumarins 529-84-0

Quinic Acid

Senkyunolide A

D-Glucoronic acid

Methyl caffeate

Ferulic acid

Organic acids Organic acid 77-95-2

Others

Others

Others 63038-10-8

rides and A 6556-12-3

Phenolic acids henolic acid3843-74-1

Phenolic acids henolic acid 537-98-4

358370

3397600

9430.5

9

D-Pinitol

Others

Alkaloids

Others

Others 10284-63-6

Alkaloids 1958-08-02

Caffeine

Cinidilide

Others

-

44738

Tyrosine methyl emsino acids and derivaticids and de 1080-06-4

Gluconic acid Others rides and A 526-95-4

26378

2093500

24574

Dihydroferulic Acid Phenolic acids henolic acid1135-23-5

oxy-L-phenylalanminino acids and derivaticids and de 59-92-7

55366

Syringic acid

Phenolic acids henolic acid 530-57-4

14629

Erythrose-4-phosph

canoic acid (Lauric

Others

Lipids

rides and A 585-18-2

ree fatty acid143-07-7

22107

125620

1381900

3345300

19303000

113610

22146

NG-Dimethyl-L-amrgino acids and derivaticids and de30315-93-6

Spermine Alkaloids Phenolamine 71-44-3

L-Tryptophan mino acids and derivaticids and de 73-22-3

D-Panthenol

Others

Others

rides and A 81-13-0

Others 24393-56-4

hyl p-methoxycinn

6-Di-tert-butylphen Phenolic acids henolic acid 128-39-2

-Di-Tert-Butylphe Phenolic acids henolic acid 96-76-4

Acetyl-L-phenylalmanino acids and derivaticids and de 2018-61-3

Dimethoxycinnamic Phenolic acids henolic acid14737-89-4

ulic acid methyl es Phenolic acids henolic acid2309-07-01

445560

463250

60450

8095.9

13103

Sinapinaldehyde

D-Saccharic acid

D-Galactaric acid

Sedoheptulose

Phenolic acids henolic acid4206-58-0

43035

Others

Others

Others

rides and A 87-73-0

rides and A 526-99-8

rides and A 3019-74-7

1504900

1432900

679610

32002

eoxyribose-1-phospcleotides and derivatiivdes and der17210-42-3

N-Acetyl-L-Arginminino acids and derivaticids and de 155-84-0

222380

2352.3

101390

1737600

484750

43453

ydroxydodecanoic

tyl-5-hydroxytrypt

D-Pantothenic Acid

cetyl-D-galactosam

Lipids

Alkaloids

Others

ree fatty acid505-95-3

Alkaloids 1210-83-9

Vitamin 79-83-4

Others

rides and A 1811-31-0

Glycylphenylalanimnino acids and derivaticids and de 3321-03-7

lycyl-L-phenylalmanino acids and derivaticids and de 721-66-4

97442

Dehydrovomifoliol

Terpenoids

onoterpenoi15764-81-5

266840

 

[M-H]-

[M-H]-

-Trihydroxy-6-meLtignans and CoumarinCoumarins

-

3

3

3

1

3

2

3

3

3

3

3

3

3

2

3

1

1

1

3

3

3

1

1

3

3

3

3

1

1

3

1

3

3

3

1

1

1

1

3

3

3

1

3

3

1

1

3

27804

18880

Sinapic acid

Phenolic acids henolic acid 530-59-6

Others Others 94596-27-7

[M-H2O+H]+

[M+H]+

[M-H]-

Senkyunolide H

9

L-Prolyl-L-Leucimneino acids and derivaticids and de52899-07-7

Myristic Acid Lipids ree fatty acid544-63-8

142580

19692000

109020

13436

[M+H]+

[M+H]+

[M+H]+

[M-H]-

L-Valyl-L-Leucinmeino acids and derivaticids and de 3989-97-7

Lysine-Butanoic Amino acids and derivaticids and de80407-71-2

6-Methylflavone

Flavonoids

Flavones 29976-75-8

65760

cic acid Dimethyl E Phenolic acids henolic acid

-

447370

398250

65079000

168600

494880

288410

231970

3562500

1889400

268080

160430

110130

84326

[M-H]-

entadecenoic Acid

Palmitaldehyde

Lipids

Lipids

ree fatty aci8d4743-29-3

ree fatty acid629-80-1

[M-H]-

[M+H]+

[M+H]+

[M-H]-

ethyl-2'-Deoxycyticleotides and derivatiivdes and der 838-07-3

Thymidine cleotides and derivatiivdes and der 50-89-5

-methylmyristic ac

Pentadecanoic Acid

Lipids

Lipids

ree fatty acid2485-71-4

ree fatty acid1002-84-2

[M-H]-

[M+H]+

[M+H]+

[M-H]-

Cytarabine cleotides and derivatiivdes and der 147-94-4

Cytidine

Uridine

cleotides and derivatiivdes and der 65-46-3

cleotides and derivatiivdes and der 58-96-8

[M+H]+

[M-H]-

D-Arabinofuranosycleotides and derivatiivdes and der3083-77-0

Tridecanedioic acid Organic acids Organic acid 505-52-2

L-Leucyl-L-Leucimnino acids and derivaticids and de 3303-31-9

Acetyl-L-Tryptopmhino acids and derivaticids and de 1218-34-4

[M+H]+

[M-H]-

1383900

55017

[M+H]+

[M-H]-

-Glutamyl-L-valimnino acids and derivaticids and de

γ-Glu-Cys mino acids and derivaticids and de 636-58-8

umaroylhydroxypu Alkaloids Phenolamine

-

12141

[M+H]+

[M+H]+

[M+H]+

[M-H]-

-

13826000

3234300

33360

2'-Deoxyadenosinecleotides and derivatiivdes and der 958-09-8

2'-Deoxyinosine cleotides and derivatiivdes and der 890-38-0

Z)-Hexadecenoic a

Palmitoleic Acid

tinate D-ribonucleo

Palmitic acid

Lipids

Lipids

Others

Lipids

Lipids

ree fatty acid2416-19-5

ree fatty acid373-49-9

Vitamin 17720-18-2

ree fatty acid 57-10-3

4338.4

5792.3

726350

74719

[M-H]-

[M+H]+

[M-H]-

[M+H]+

[M-H]-

Choline Alfoscerate

-Tetradecanedioic

ucosamine 1-phosp

Fructose 6-Phosph

Glucose-1-phosphat

Glucose 6-phospha

PC

28319-77-9

244580

12459

Organic acids Organic acid 821-38-5

[M-H]-

Others

Others

Others

Others

rides and A 2152-75-2

rides and A 643-13-0

rides and A 59-56-3

rides and A 56-73-5

11610

[M-H]-

2479900

2921800

3610600

174830

19277

[M-H]-

[M-H]-

[M+H]+

[M-H]-

γ-Glutamyl-L-leumciino acids and derivaticids and de 2566-39-4

orbitol-6-phosphat

ide-3-hydroxy-y-bu

Abscisic acid

Others

Others

rides and A20479-58-7

[M+H]+

[M-H]-

Others

-

219560

10062

Organic acids Organic acid21293-29-8

[M+H]+

[M-H]-

N-Feruloylputrescin

yl 7,10-hexadecadie

Alkaloids

Lipids

Phenolamine501-13-3

2946100

5512900

799910

48864000

47218000

150740

ree fatty acid

-

[M+H]+

[M+H]+

[M+H]+

[M-H]-

2'-Deoxyguanosinecleotides and derivatiivdes and der 961-07-9

Adenosine cleotides and derivatiivdes and der 58-61-7

Vidarabine

Others

Others 5536-17-4

rabinosyl)hypoxancleotides and derivatiivdes and der7013-16-3

 

[M+H]+

[M+H]+

[M-H]-

[M+H]+

[M-H]-

[M-H]-

[M-H]-

[M-H]-

[M-H]-

[M-H]-

[M+H]+

[M+H]+

[M+H]+

[M-H]-

[M+H]+

[M+H]+

[M+H]+

[M+H]+

[M+H]+

[M+H]+

[M-H]-

[M+H]+

[M-H]-

[M-H]-

[M+H]+

[M-H]-

[M+H]+

[M+H]+

[M-H]-

[M+H]+

[M+H]+

[M+H]+

[M-H]-

[M+H]+

[M+H]+

[M+H]+

[M-H]-

[M-H]-

[M-H]-

[M-H]-

[M-H]-

[M+H]+

[M-H]-

[M+H]+

[M-H]-

[M-H]-

[M-H]-

Inosine

cleotides and derivatiivdes and der 58-63-9

3

3

2

1

1

3

3

3

3

1

3

1

3

3

3

1

1

1

1

3

2

3

2

1

3

3

3

3

1

3

1

3

2

3

1

2

3

3

3

1

3

3

3

3

3

3

3

6450.9

61524

Feruloyl-sn-glycer Phenolic acids henolic acid

-

-Heptadecenoic Ac

Apigenin

Lipids

Flavonoids

Flavonoids

Lipids

ree fatty aci2d9743-97-3

Flavones 520-36-5

Flavones 548-83-4

ree fatty acid112-92-5

Flavanonols 548-82-3

Flavanones 480-41-1

Others 84380-01-8

97613

113040

27733

n (3,5,7-Trihydroxy

1-Octadecanol

Pinobanksin

88449

Flavonoids

Flavonoids

Others

6067

(5,7,4'-Trihydroxy

α-Arbutin

2936.5

47674

droxyhexadecanoic Organic acids Organic acid 764-67-0

2226600

139910

2811500

17427

Lauryldiethanolam

exadecylsphingosin

Others

Lipids

Others 1541-67-9

Sphingolipid

-

-

Cyclo(Phe-Glu)mino acids and derivaticids and de

L-Saccharopinemino acids and derivaticids and de 997-68-2

18613

Dehydrocrepenyni

Senkyunolide M

Dibutyl phthalate

Lipids

Others

ree fatty acid4154-44-3

Others 146986-60-9

33078

22351000

15789000

15925000

16012000

55801

Phenolic acids henolic acid 84-74-2

Diisobutyl phthalat Phenolic acids henolic acid 84-69-5

tyl isobutyl phthal Phenolic acids henolic acid17851-53-5

eucyl-L-phenylalmanino acids and derivaticids and de56217-82-4

Crepenynic acid

Z,11E,13Z-octadec

α-Linolenic Acid

γ-Linolenic Acid

-11E,13E,15Z-trie

Lipids

Lipids

Lipids

Lipids

Lipids

ree fatty acid2277-31-8

ree fatty acid544-72-9

ree fatty acid463-40-1

ree fatty acid506-26-3

ree fatty aci2d5575-00-2

13674

136550

7970100

15310

2542800

1301500

55573

Coumaroylmalic ac Phenolic acids henolic acid

partyl-L-Phenylamlaino acids and derivaticids and de13433-09-5

-

ruloylhydroxyputre

Linoleic acid

Alkaloids

Lipids

Phenolamine

-

10930000

28726000

80981

ree fatty acid 60-33-3

N6-methyladenosincleotides and derivatiivdes and der1867-73-8

-O-Methyladenosincleotides and derivatiivdes and der2140-79-6

457670

126130

4635900

628850

10821000

174540

90295

mide (9-Octadecena

ecanoic acid(Vacce

Lipids

Lipids

ree fatty acid301-02-0

ree fatty acid506-17-2

side; 2-Hydroxyadcleotides and derivatiivdes and der1818-71-9

Guanosine cleotides and derivatiivdes and der 118-00-3

Stearamide Alkaloids 124-26-5

Xanthosine cleotides and derivatiivdes and der 146-80-5

Stearic Acid Lipids ree fatty acid 57-11-4

5-Methylphenol-1-O Phenolic acids henolic acid

Alkaloids

1172600

24367

-

exadecanedioic ac

y-palmitic acid me

,18-Octadecanedio

rgininosuccinic ac

Methyl linolenate

Lipids

Lipids

Lipids

ree fatty acid505-54-4

ree fatty aci5d1883-36-4

ree fatty acid3155-43-9

366230

12150

13754

Organic acids Organic acid2387-71-5

Lipids ree fatty acid301-00-8

183840

10160

Glutamylphenylalamnino acids and derivaticids and de 7432-24-8

27860

Z,12Z,15Z-octadec

Hydroxylinolenic a

Others

Others

Others

Others

-

-

66047

52184

 

[M-H]-

[M-H]-

[M-H]-

[M+H]+

[M-H]-

[M+H]+

[M-H]-

[M-H]-

[M-H]-

[M+H]+

[M+H]+

[M+H]+

[M-H]-

[M-H]-

[M-H]-

[M+H]+

[M+H]+

[M+H]+

[M+H]+

[M-H]-

[M-H]-

[M-H]-

[M-H]-

[M-H]-

[M-H]-

[M-H]-

[M-H]-

[M+H]+

[M-H]-

[M-H]-

[M-H]-

[M+H]+

[M+H]+

[M-H]-

[M-H]-

[M-H]-

[M-H]-

[M-H]-

[M-H]-

[M-H]-

[M+H]+

[M-H]-

[M-H]-

[M-H]-

[M+H]+

[M+H]+

[M+H]+

E,12Z-octadecadie

9Z,11E,15Z-octade

y-6,9,11-octadecatr

Lipids

Lipids

Lipids

ree fatty aci5d4232-59-6

ree fatty aci8d7984-82-5

ree fatty aci7d4784-20-6

3

3

3

1

3

3

1

3

1

1

3

3

1

3

3

3

3

3

3

1

1

1

3

3

1

3

3

3

2

2

2

3

3

3

1

3

2

3

1

3

3

1

3

3

3

3

3

3562.5

10458

268030

321260

86205

oimidazole ribonucleotides and derivatiivdes and der25635-88-5

Cis-Coutaric acid

o-12Z-Octadecenoi

-10E,12Z-octadeca

0S)-(12Z)-9,10-Ep

Hydroxyoctadeca-

Phenolic acids henolic acid67920-37-0

Lipids

Lipids

Lipids

Lipids

ree fatty aci1d12543-32-5

ree fatty aci1d5514-85-9

ree fatty aci1d6833-56-0

ree fatty aci1d0219-69-9

24591

96618

30226

66331

y-5'-(methylthio)adcleotides and derivatiivdes and der2457-80-9

N7-Methylguanosincleotides and derivatiivdes and der20244-86-4

6013400

103010

1334.1

136140

161020

46157

Acerogenin A

Ricinoleic acid

1-Eicosanol

Phenolic acids henolic acid60503-28-8

Lipids

ree fatty acid141-22-0

ree fatty acid629-96-9

Lipids

hiyroxy-8-methox

4'-Trihydroxy-6-m

3'-Trihydroxy-4'-m

Tectorigenin

Flavonoids

Flavonoids

Flavonoids

Flavonoids

Flavonoids

Flavones

-

Flavones 1447-88-7

Flavanones 520-34-3

Isoflavones 548-77-6

Flavones 569-92-6

267880

256330

224640

218340

4263700

3926200

6174800

13484000

78194

citrin (7-Methylkae

O-Salicyl-D-gluco Phenolic acids henolic acid60517-74-0

ucosyloxybenzoic a Phenolic acids henolic acid

ylic acid-2-O-gluc Phenolic acids henolic acid10366-91-3

-

xy-5,7-dimethoxyf

ydroxyoctadecanoi

ydroxyoctadecanoic

-2-hydroxystearic a

droxyoctadecanoic

Robinetin

Flavonoids

Lipids

Flavanols

-

ree fatty acid106-14-9

ree fatty aci2d6633-48-7

ree fatty acid629-22-1

ree fatty aci4d5261-96-9

Flavonols 490-31-3

Flavonols 480-16-0

Lipids

72713

Lipids

84739

Lipids

579710

26890

Flavonoids

Flavonoids

Morin

5124.5

1036400

142280

343670

50902

Tachioside

Phenolic acids henolic aci1d09194-60-7

yl-3,4-dihydroxybe Phenolic acids henolic acid

-

1-Monomyristin

Nicotianamine

Lipids

Alkaloids

Lipids

ree fatty acid589-68-4

Alkaloids 34441-14-0

ree fatty acid506-32-1

Arachidonic Acid

18160

tathione reducedmfoino acids and derivaticids and de 70-18-8

Glutamine-O-glycmosino acids and derivaticids and de

Eicosadienoic acid Lipids ree fatty acid5598-38-9

439510

294800

332520

29412

-

utamic acid-O-glymcino acids and derivaticids and de

Feruloylmalic acid Phenolic acids henolic acid

-

-

1856500

166760

63956

-10E,12,15Z-octad ree fatty aci1d11004-08-1

γ-Glutamyltyrosinmeino acids and derivaticids and de 7432-23-7

Lipids

y-(9z,11e,15z)-octa

o-10(E),15(Z)-octa

Eicosenoic acid

Dhurrin

Lipids

Others

ree fatty aci6d7597-26-6

212330

4075.3

38124

Others

-

Lipids

ree fatty aci2d6764-41-0

Phenolamine499-20-7

Alkaloids

48203

methylamino)guancleotides and derivatiivdes and der2140-67-2

-Hydroxyethyl)adecleotides and derivatiivdes and der4338-48-1

95226

85780

 

[M-H]-

[M+H]+

[M-H]-

[M-H]-

[M-H]-

[M-H]-

[M-H]-

[M-H]-

[M-H]-

[M-H]-

[M+H]+

[M+H]+

[M-H]-

[M-H]-

[M+H]+

[M-H]-

[M-H]-

[M-H]-

[M-H]-

[M+H]+

[M+H]+

[M-H]-

[M+H]+

[M+H]+

[M-H]-

[M-H]-

[M+H]+

[M-H]-

[M-H]-

[M-H]-

[M-H]-

[M-H]-

[M-H]-

[M-H]-

[M-H]-

[M+H]+

[M+H]+

[M-H]-

[M+H]+

[M-H]-

[M+H]+

[M+H]+

[M-H]-

[M+H]+

[M-H]-

[M-H]-

[M+H]+

-L-tartaric acid (Ca Phenolic acids henolic acid67879-58-7

2

3

1

1

3

3

3

3

3

3

3

3

1

1

2

2

2

2

3

3

3

3

3

3

3

3

3

3

3

1

1

1

1

3

1

3

2

3

3

1

3

3

1

3

1

2

3

55763

416850

78400

Alnusonol

Phenolic acids henolic acid52330-12-8

y-9Z,11E-Octadec

(7S,8S)-Dihydroxy

oxy-9Z,11E-octade

Z,12Z)-5,8-Dihydr

Lipids

Lipids

Lipids

Lipids

ree fatty acid5502-91-0

ree fatty aci1d43288-65-7

ree fatty aci3d3964-75-9

211460

872750

134740

354840

92533

ree fatty acid

-

l salicylate-2-O-glu Phenolic acids henolic acid10019-60-0

ndelic acid-β-gluco Phenolic acids henolic acid

-

)-12,13-Dihydroxy

ydroxy ricinoleic ac

Lipids

Lipids

ree fatty aci2d63399-35-5

ree fatty acid

Flavones 520-11-6

366450

3849.5

35210

-

'-Tetrahydroxy-6-m Flavonoids

Selagin Flavonoids

echuic acid-4-O-g Phenolic acids henolic acid

Flavones

-

-

8296.8

7447400

7342400

155080

159270

373890

7616200

85205

Gentisoyl-D-gluco Phenolic acids henolic acid23445-11-6

O-Galloyl-rhamno Phenolic acids henolic acid

oxyethyl)-2-O-gluc Phenolic acids henolic acid

-

-

Cimidahurinine

Vanilloloside

Phenolic acids henolic aci1d42542-89-0

Phenolic acids henolic acid74950-96-2

henylethanol-β-D-g Phenolic acids henolic acid

Hydroxysphingani Lipids Sphingolipid 554-62-1

denudaquinol Lignans and Coumarin Lignans 189105-40-

-

1095900

5325.6

65341

omyricetin (Ampe

Corylin

Flavonoids

Flavonoids

Flavanonols27200-12-0

Isoflavones53947-92-5

20845

-(Methyl)glutathimonino acids and derivaticids and de 2922-56-7

asmonoyl-L-Isoleu Organic acids Organic acid120330-93-0

dine 5'-monophospcleotides and derivatiivdes and der 58-97-9

295120

1059

100390

19515

-Oleoylethanolami

Alkaloids

Alkaloids 111-58-0

tartaric acid (Ferta Phenolic acids henolic acid

oxyl)-benzofuranyl Phenolic acids henolic acid

nic acid-O-β-D-glu Phenolic acids henolic acid

E)-p-Cumaroyl]-D- Phenolic acids henolic acid

-

-

-

-

670500

638430

3582300

5815400

2867100

3534900

70634

maric acid-4-O-glu Phenolic acids henolic aci1d17405-48-8

ulic acid-1-O-gluco Phenolic acids henolic acid14364-05-7

Rutinose

Others

rides and A 90-74-4

pberryketone gluco Phenolic acids henolic acid38963-94-9

DehydrodiisoeugenLoignans and Coumarin Lignans 2680-81-1

19068000

9

aminoethyl-β-D-glu

Demethyl coniferin Phenolic acids henolic acid

Jasminoside C Terpenoids

clic 3',5'-Adenylic acleotides and derivatiivdes and der 60-92-4

Others

Others

-

129520

22268

-

onoterpeno2i14125-05-0

43650

108670

108760

307240

7925400

39186

-3',6-dimethoxyfla

rihydroxy-3',5'-dim

Flavonoids

Flavonoids

Flavones 18085-97-7

Flavones 520-32-1

etophenone-4-O-β- Phenolic acids henolic acid

Mudanoside A Phenolic acids henolic acid

-

-

-

-

-

etophenone-6-O-β- Phenolic acids henolic acid

ethoxyphenyl-1-O Phenolic acids henolic acid

magnolignan DLignans and Coumarin Lignans

7147200

19874000

128920

 

[M-H]-

[M+H]+

[M+H]+

[M+H]+

[M+H]+

[M-H]-

[M-H]-

[M-H]-

[M-H]-

[M-H]-

[M-H]-

[M+H]+

[M-H]-

[M-H]-

[M-H]-

[M-H]-

[M-H]-

[M-H]-

[M-H]-

[M-H]-

[M+H]+

[M-H]-

[M-H]-

[M-H]-

[M-H]-

[M-H]-

[M-H]-

[M-H]-

[M-H]-

[M-H]-

[M-H]-

[M-H]-

[M-H]-

[M-H]-

[M-H]-

[M+H]+

[M-H]-

[M-H]-

[M-H]-

[M-H]-

[M-H]-

[M-H]-

[M-H]-

[M+H]+

[M+H]+

[M+H]+

[M+H]+

hydroxy-11-Octade

Monopalmitin

Lipids

Lipids

ree fatty aci2d9907-57-1

Glycerol este 542-44-9

3

1

3

3

3

3

2

2

3

1

1

3

1

3

2

2

3

3

3

3

3

3

3

3

2

3

3

1

1

3

1

1

1

1

2

3

3

3

1

3

3

1

1

3

3

3

3

17151

98044

adenosine-5'-monocleotides and derivatiivdes and der 653-63-4

doxine-5'-O-gluco Others Vitamin

970640

3571300

2877500

27944

-

yinosine-5'-monophcleotides and derivatiivdes and der3393-18-8

O-Galloyl-D-gluco Phenolic acids henolic acid13186-19-1

O-Galloyl-D-gluco Phenolic acids henolic acid58511-73-2

344860

25993

Galloyl-D-hamame Phenolic acids henolic acid

O-Galloyl-D-gluco Phenolic acids henolic acid

nyl)ethanediol 1-O- Phenolic acids henolic acid

-

-

-

25818

3460300

3362400

42998

Koaburaside

Phenolic acids henolic acid41653-73-0

maroyl-7-deoxyreh

o-3-phospho)-1D-

Terpenoids

Others

onoterpenoi

rides and A

-

-

69627

p-Coumaroylquinic Phenolic acids henolic acid32451-86-8

p-Coumaroylquinic Phenolic acids henolic acid1899-30-5

p-Coumaroylquinic Phenolic acids henolic acid87099-71-6

484140

1069500

1931800

34818

methoxycoumarinL-ignans and CoumarinCoumarins

ucose 1,6-bisphosp Others rides and A10139-18-1

uletin-7-O-glucosLi ignans and CoumarinCoumarins

DihydroxyCoumarLinignans and CoumarinCoumarins 531-75-9

Sinapoyl malate Phenolic acids henolic acid92344-58-6

hoyxcinnamate glu Phenolic acids henolic acid

anoic acid (Beheni Lipids ree fatty acid112-85-6

(E)-Caffeoyl]-D-gl Phenolic acids henolic acid

Grevilloside F Phenolic acids henolic acid094004-50-

-

19927

-

6437.1

10111

27206

-

23879

21623

-

718600

62142

ic Acid-4-O-Glucu Phenolic acids henolic acid

O-Caffeoyl-D-gluco Phenolic acids henolic acid

-

-

19364

570950

7216700

7813600

3825700

1137300

5097300

13006000

6187000

723050

25989

Lactobiose

D-Maltose

Melibiose

Others

Others

Others

Others

Others

Others

Others

rides and A 63-42-3

rides and A 69-79-4

rides and A 585-99-9

rides and A 3687-64-7

rides and A 99-20-7

rides and A 57-50-1

rides and A13718-94-0

Galactinol

D-Trehalose

D-Sucrose

Isomaltulose

Coniferin

Phenolic acids henolic acid 531-29-3

magnovatins A Lignans and Coumarin Lignans

Usnic acid Phenolic acids henolic acid 125-46-2

-

4003.5

3142000

32725

hydrocaffeoylgluco Phenolic acids henolic acid

galdehyde-4-O-gluc Phenolic acids henolic acid

-

-

Maltitol

Others

rides and A 585-88-6

226810

9

Nectandrin B Lignans and Coumarin Lignans 74683-16-2

e 3',5'-cyclic monocleotides and derivatiivdes and der7665-99-8

486720

16186

Kankanoside A

Terpenoids

onoterpenoi

-

osine 5'-monophoscleotides and derivatiivdes and der 61-19-8

sine 5'-monophosphcleotides and derivatiivdes and der 131-99-7

439460

49985

α-Linolenoyl-glyce

α-Linolenoyl-glyce

Lipids

Lipids

Glycerol este

-

5901.5

6226.4

Glycerol este55268-58-1

 

[M-H]-

[M-H]-

[M-H]-

[M-H]-

[M+H]+

[M+H]+

[M+H]+

[M-H]-

[M-H]-

[M-H]-

[M+H]+

[M-H]-

[M+H]+

[M-H]-

[M+H]+

[M+H]+

[M+H]+

[M+H]+

[M+H]+

[M+H]+

[M-H]-

[M+H]+

[M-H]-

[M+H]+

[M+H]+

[M-H]-

[M+H]+

[M+H]+

[M-H]-

[M-H]-

[M+H]+

[M+H]+

[M-H]-

[M+H]+

[M+H]+

[M+H]+

[M-H]-

[M-H]-

[M+H]+

[M+H]+

[M+H]+

[M+H]+

[M-H]-

[M+H]+

[M+H]+

[M+H]+

[M+H]+

ic acid (5-O-Caffeo Phenolic acids henolic acid 906-33-2

nic acid (4-O-Caffe Phenolic acids henolic acid 905-99-7

-Caffeoylquinic aci Phenolic acids henolic acid1241-87-8

acid (3-O-Caffeoy Phenolic acids henolic acid 327-97-9

Sesamin Lignans and Coumarin Lignans 607-80-7

1

1

1

1

3

1

1

3

2

1

3

3

2

2

2

2

3

3

2

2

3

3

3

3

1

3

3

3

3

3

2

1

3

3

3

3

2

2

3

2

1

3

2

3

3

3

3

193070

8246300

9

12969000

41194

1-Linoleoylglycero

2-Linoleoylglycero

Lipids

Lipids

Glycerol este2277-28-3

Glycerol este3443-82-1

7480.5

14054

Sinapoyltartaric aci Phenolic acids henolic acid

O-Feruloyl-D-Gluc Phenolic acids henolic acid

-

-

11496

215090

6919.1

41810

Coniferyl ferulate Phenolic acids henolic acid63644-62-2

-Oleoyl-Sn-Glycero Lipids Glycerol est1e29784-87-8

Glucosyringic Acid Phenolic acids henolic acid

-

1725800

20289000

2096700

1524900

19556000

29890

oletin-7-O-glucurLoignans and CoumarinCoumarins132752-65-9

ogenic acid methyl Phenolic acids henolic acid29708-87-0

O-Feruloylquinic a Phenolic acids henolic acid1899-29-2

O-Feruloylquinic a Phenolic acids henolic acid

-

-

o-α-ionol-β-D-gluc

Grevilloside C

Swertiamarin

Others

Others

Phenolic acids henolic acid094004-47-

Terpenoids onoterpenoi17388-39-5

16060

59832

NortrachelogeninLignans and Coumarin Lignans 34444-37-6

acylglycerol-7-O-β Phenolic acids henolic acid

9

-

28772

boflavin (Vitamin B

oxy-3,3'-dimethoxy

Others

Others

Vitamin 83-88-5

37907

Others

-

-

39482

farnesiferol B Lignans and CoumarinCoumarins

21058

Succinyladenosinecleotides and derivatiivdes and der4542-23-8

960330

4117500

4498

O-Glucosyl-sinapa Phenolic acids henolic acid

-

imethylmatairesinLoignans and Coumarin Lignans 25488-59-9

oresinol dimethy eLtignans and Coumarin Lignans 29106-36-3

5000.2

90125

Majoroside

ucosyloxyjasmanic Phenolic acids henolic acid

Ampelopsisionosid Others Others

Others

Others 134140-02-6

-

-

61708

251760

23808000

1266600

41426

2-ethylhexyl)phtha Phenolic acids henolic acid 117-81-7

massoniresinol Lignans and Coumarin Lignans 96087-10-4

Trollioside

Ribosyladenosinecleotides and derivatiivdes and der

6,7,8,3',4'-Hexamet Flavonoids

nzyl β-primeverosi Phenolic acids henolic aci1d30622-31-0

Phenolic acids henolic acid

-

-

983820

2898.2

65452

Flavones 478-01-3

l-(2''-O-xylosyl)glu Phenolic acids henolic acid

-

-

75000

rachelogenin amid

Salviifoside A

Alkaloids

Alkaloids

5315.9

36293

Phenolic acids henolic acid175539-71-

hylpicraquassiosiLdignans and CoumarinCoumarins

-

-

541350

7560.2

103780

1145800

62443

mpferol-3-O-arabin

rehalose 6-phospha

maroylferuloylcada

soPE 14:0(2n isom

LysoPE 14:0

Flavonoids

Others

Flavones

rides and A 4484-88-2

Alkaloids

Lipids

Phenolamine

LPE

-

-

-

-

Lipids

LPE

68394

taroyl-hydroxycouLmignans and CoumarinCoumarins

1001700

 

[M-H]-

[M-H]-

[M-H]-

[M-H]-

[M-H]-

[M-H]-

[M-H]-

[M-H]-

[M+H]+

[M+H]+

[M+H]+

[M-H]-

[M-H]-

[M+H]+

[M+H]+

[M+H]+

[M+H]+

[M+H]+

[M-H]-

[M+H]+

[M+H]+

[M+H]+

[M+H]+

[M+H]+

[M+H]+

[M+H]+

[M-H]-

[M-H]-

[M-H]-

[M-H]-

[M+H]+

[M+H]+

[M+H]+

[M-H]-

[M+H]+

[M+H]+

[M+H]+

[M-H]-

[M-H]-

[M-H]-

[M-H]-

[M-H]-

[M-H]-

[M-H]-

[M-H]-

[M-H]-

[M+H]+

enosine 5'-diphosphcleotides and derivatiivdes and der 58-64-0

3

3

3

3

3

1

1

3

3

3

3

2

2

3

1

1

3

3

3

3

3

3

3

3

3

3

3

3

3

3

3

3

3

2

1

3

3

3

1

3

3

3

2

1

1

3

3

62058

115100

72818

9

Regaloside G

Phenolic acids henolic aci1d20601-64-1

n-7-O-Glucoside (G

-rhamnoside (Afze

-7-O-glucoside(Co

Flavonoids

Flavonoids

Flavonoids

Isoflavones 529-59-9

Flavonols 482-39-3

Flavones 578-74-5

93149

562140

704560

10373

132090

132350

72596

67977

30848

5921.9

587710

650620

225370

15894

9

-(2''-O-glucosyl)glu Phenolic acids henolic acid

enzyl-β-gentiobiosi Phenolic acids henolic acid56775-64-5

-

Darendoside A

Morin-3-O-xylosid

n-3-O-xyloside (Re

rcetin-3-O-α-L-ara

Phenolic acids henolic aci1d49596-95-2

Flavonoids

Flavonoids

Flavonoids

Flavonols

-

Flavonols 549-32-6

Flavonols 572-30-5

-O-Caffeoylarbutin Phenolic acids henolic aci1d36172-60-6

-4'-O-glucoside (T

LysoPC 12:0

Flavonoids

Lipids

Chalcones 4192-90-9

LPC 20559-18-6

soPE 15:0(2n isom

LysoPE 15:0

Lipids

LPE

LPE

-

-

Lipids

N'-Diferuloylputresc

Epicatechin gallate

O-Galloyltricetiflav

Regaloside D

Alkaloids

Flavonoids

Flavonoids

Phenolamine42369-86-8

Flavanols 1257-08-5

Flavanols

-

Phenolic acids henolic aci1d20601-66-3

108320

19699

7623.5

405250

57295

18787

31598

290650

9

xy-N-methyl-benzy

-3-O-rhamnoside(Q

7-O-glucoside (Cy

teolin-4'-O-glucosi

mpferol-4'-O-gluco

ol-3-O-glucoside (A

cosyl-2-Hydroxyna

dictyol-3'-O-gluco

odictyol-8-C-gluco

adendrin-7-O-gluc

oquinone (Vitamin

soPE 16:1(2n isom

LysoPE 16:1

Alkaloids

Flavonoids

Flavonoids

Flavonoids

Flavonoids

Flavonoids

Flavonoids

Flavonoids

Flavonoids

Flavonoids

Others

uinoline alka

-

Flavonols 522-12-3

Flavones 5373-11-05

Flavones 6920-38-3

Flavonols

-

Flavonols 480-10-4

Flavanones

Flavanones

-

-

Flavanones023271-51-

Flavanonols28189-90-4

Vitamin 84-80-0

35574

246070

6026.2

74676

153440

96348

12516000

1745700

88133

122520

125040

106270

111680

45812

102150

941140

780220

865630

552190

Lipids

LPE

LPE

-

-

-

Lipids

oyl-caffeoylquinic Phenolic acids henolic acid

LysoPE 16:0

soPE 16:0(2n isom

1-O-(2''-O-apiosyl)

Betulonic acid

Lipids

LPE 53862-35-4

Lipids

LPE

-

-

Terpenoids

Terpenoids

Terpenoids

Terpenoids

Terpenoids

onoterpenoi

Triterpene 4481-62-3

Triterpene

Triterpene17990-42-0

Triterpene

(13)-en-3-one-28-o

Oleanonic acid

-

3-Ketoursolic acid

-

Single Nucleotide(cleotides and derivatiivdes and der6184-17-4

Ursonic acid

Ursolic acid

Terpenoids

Terpenoids

Terpenoids

Terpenoids

Triterpene 6246-46-4

Triterpene 77-52-1

hydroxy-12(13)-en

ydammara-21-oic a

Triterpene

Triterpene

-

-

-

partic acid-O-diglmucino acids and derivaticids and de

 

[M-H]-

[M-H]-

[M-H]-

[M+H]+

[M+H]+

[M-H]-

[M+H]+

[M+H]+

[M+H]+

[M-H]-

[M+H]+

[M-H]-

[M-H]-

[M-H]-

[M-H]-

[M+H]+

[M-H]-

[M+H]+

[M+H]+

[M-H]-

[M-H]-

[M-H]-

[M-H]-

[M-H]-

[M-H]-

[M-H]-

[M-H]-

[M-H]-

[M-H]-

[M-H]-

[M-H]-

[M-H]-

[M-H]-

[M+H]+

[M+H]+

[M-H]-

[M+H]+

[M+H]+

[M-H]-

[M]+

gallocatechin-3-gal

locatechin 3-O-gal

Flavonoids

Flavonoids

Flavanols 989-51-5

Flavanols 5127-64-0

3

3

3

3

3

2

3

3

3

1

3

1

1

1

3

1

3

3

1

1

1

1

3

3

1

1

1

1

1

1

1

1

1

1

1

3

1

1

1

3

1

1

3

3

3

3

3

60663

79527

3573400

29233

33132

9

anilate-1-O-Sopho Phenolic acids henolic acid

-

Yuanhuanin

Tectoridin

Flavonoids

Flavonoids

Flavonoids

Flavonoids

Flavonoids

Flavonoids

Flavonoids

Flavonoids

Flavonoids

Flavonoids

Flavonoids

Flavonoids

Lipids

Flavones 83133-14-6

Isoflavones 611-40-5

ykaempferol-7-O-rh

hylKaempferol-3-g

pidulin-7-O-Gluco

Isohyperoside

Flavonols

Flavones

-

-

18796

20908

138310

34326

196020

24799

21076

56018

21024

45525

8868.5

41932

215580

965830

8215.1

123910

79443

8824.4

45109

178830

188830

103440

136660

26242

212900

50910

177780

2381000

100210

75952

46028

157470

1280500

28249

21545000

4262800

26261

36689

9

Flavones 17680-84-1

Flavonols 35589-21-0

Flavonols 482-35-9

3-O-glucoside (Iso

ykaempferol-7-O-

ercetin-7-O-glucos

4'-O-glucoside (Sp

n-3-O-galactoside (

peretin-5-O-glucos

soPE 17:1(2n isom

Grevilloside M

Flavonols

-

Flavonols 491-50-9

Flavonols 20229-56-5

Flavonols 482-36-0

Flavanones69651-80-5

LPE

-

Phenolic acids henolic acid407163-62-

LysoPE 17:0

Lipids

LPE

-

LysoPC 14:0

Lipids

LPC 20559-16-4

Triterpene 472-15-1

Betulinic acid

Terpenoids

Terpenoids

Terpenoids

xy 5(6),12(13)diene

Camaldulenic acid

Triterpene

-

Triterpene71850-15-2

maroylferuloyltarta Phenolic acids henolic acid

-

-

-

roxyurs-12,18-dien

Gypensapogenin F

Alphitolic acid

Terpenoids

Terpenoids

Terpenoids

Terpenoids

Terpenoids

Terpenoids

Terpenoids

Terpenoids

Terpenoids

Terpenoids

Alkaloids

Triterpene

Triterpene

Triterpene19533-92-7

-Hydroxyursolic ac

droxy-12-ursen-28

Pomolic acid

Triterpene

Triterpene

-

-

Triterpene13849-91-7

Triterpene 4547-24-4

Triterpene26707-60-8

Corosolic acid

Hydroxyoleanolic a

7,21-semiacetal-12

Maslinic acid

Triterpene

-

Triterpene 4373-41-5

Alkaloids 2800-34-2

rmyltetrahydrofolic

LysoPE 18:4

Lipids

LPE

-

Cichoric Acid

Phenolic acids henolic acid6537-80-0

LysoPE 18:3

Lipids

Lipids

LPE

LPE

-

-

-

-

-

-

-

-

soPE 18:3(2n isom

Darendoside B

Phenolic acids henolic acid

sinidin-3-O-glucos

soPE 18:2(2n isom

LysoPE 18:2

Flavonoids

Lipids

nthocyanidi

LPE

[M+H]+

[M+H]+

[M-H]-

[M+H]+

[M+H]+

[M+H]+

[M-H]-

Lipids

LPE

Digalloylshikimic

Isotamarixin

Phenolic acids henolic acid

Flavonoids

Flavonoids

Flavonoids

Flavonols

tin-7-O-glucoside (

mnetin-3-O-Gluco

Flavonols 6743-96-0

Flavonols 27875-34-9

9

2-O-p-Coumaroyl- Phenolic acids henolic acid94356-18-0

15878

 

[M+H]+

[M+H]+

[M+H]+

[M-H]-

amnetin-3-O-Gluc

xykaempferol-3-O-

epetin-7-O-glucosi

Flavonoids

Flavonoids

Flavonoids

Flavonols 5041-82-7

Flavonols 63422-27-5

Flavones 569-90-4

3

3

3

3

3

3

2

3

3

3

1

1

3

3

3

3

3

3

3

2

1

1

3

3

3

3

1

1

3

1

2

3

3

1

3

1

1

1

3

1

3

1

2

1

1

1

1

9

9

26076

feruloyl)-3,4-dihyd Phenolic acids henolic acid

-

-

11152

[M+H]+

[M+H]+

[M+H]+

[M+H]+

[M+H]+

[M+H]+

[M+H]+

[M+H]+

[M-H]-

soPE 18:1(2n isom

LysoPE 18:1

Lipids

Lipids

LPE

419540

517990

121680

12892

LPE 89576-29-4

LPC

Flavonols 15648-86-9

Flavones

LysoPC 15:1

Lipids

-

icetin-3-O-galacto

xy-4'-methoxyflava

LysoPE 18:0

Flavonoids

Flavonoids

Lipids

-

6490

LPE 69747-55-3

LPC 108273-89-8

46108

LysoPC 15:0

Lipids

2168900

345810

9890.7

4003400

34065

soPC 15:0(2n isom

solic Acid Methyl

Solatriose

Lipids

LPC

-

Terpenoids

Others

Triterpene 4518-70-1

rides and A 528-40-5

[M-H]-

[M-H]-

hydroxyolean-12-en

Tormentic acid

Terpenoids

Terpenoids

Terpenoids

Terpenoids

Lipids

Triterpene

Triterpene13850-16-3

Triterpene

Triterpene26532-66-1

-

[M-H]-

38853

[M-H]-

α-Dihydroxyursolic

Madasiatic acid

soPC 16:2(2n isom

doxine-5'-O-digluco

LysoPC 16:1

-

2636.6

34643

[M-H]-

[M+H]+

[M+H]+

[M+H]+

[M+H]+

[M+H]+

[M-H]-

LPC

-

-

23524

Others

Vitamin

133440

338850

136530

32658

Lipids

LPC 76790-27-7

LPC

Flavonols 39986-90-8

soPC 16:1(2n isom

ricitrin-3-O-glucos

Lipids

-

Flavonoids

oyl-3-O-galloyl-β- Phenolic acids henolic acid

-

-

-

17632

[M+H]+

[M-H]-

tuletin-3-O-glucosi

arnsetin-3-O-gluco

LysoPC 16:0

Flavonoids

Flavonoids

Lipids

Flavonols

Flavones

22211

35008

[M+H]+

[M+H]+

[M-H]-

LPC 17364-16-8

49032000

3490200

234010

749830

66945

soPC 16:0(2n isom

Lipids

LPC

-

-

maroylsinapoyltarta Phenolic acids henolic acid

[M-H]-

D-Melezitose

Maltotriose

Others

Others

Others

Others

Others

Alkaloids

Lipids

Lipids

Lipids

Alkaloids

Lipids

Others

Lipids

Lipids

Lipids

Lipids

Lipids

Lipids

rides and A 597-12-6

rides and A 1109-28-0

rides and A13382-86-0

rides and A 512-69-6

rides and A33401-87-5

[M+Na]+

[M-H]-

Manninotriose

Raffinose

28783

[M-H]-

1062200

400990

179720

96331

[M-H]-

D-Panose

[M+H]+

[M+H]+

[M+H]+

[M+H]+

[M-H]-

Cimicifugamide

soPE 20:2(2n isom

LysoPE 20:2

Phenolamine

LPE

-

-

-

-

LPE

75819

LysoPC 17:2

LPC

110350

59151

Adenosine 5'-Tripho

LysoPC 17:1

Alkaloids 56-65-5

[M+H]+

[M-H]-

LPC

Others

LPC

-

-

-

115690

24948

-O-Glucosylaucub

soPC 17:0(2n isom

LysoPC 17:0

[M+H]+

[M+H]+

[M+H]+

[M+H]+

[M+H]+

[M+H]+

130910

84734

LPC 50930-23-9

LysoPC 18:4

LPC

Glycerol este

Glycerol este

LPC

-

-

-

-

174230

9481.7

5039.9

907490

eoylglycerol-1-O-g

eoylglycerol-3-O-g

soPC 18:3(2n isom

 

[M+H]+

[M-H]-

[M-H]-

[M+H]+

[M+H]+

[M-H]-

[M-H]-

[M+H]+

[M-H]-

[M+H]+

[M+H]+

[M+H]+

[M-H]-

[M-H]-

[M-H]-

[M-H]-

[M-H]-

[M+H]+

[M+H]+

[M-H]-

[M-H]-

[M-H]-

[M-H]-

[M-H]-

[M+H]+

[M-H]-

[M-H]-

[M-H]-

[M-H]-

[M-H]-

[M-H]-

[M+H]+

[M+H]+

[M+H]+

[M-H]-

[M-H]-

[M-H]-

[M+H]+

[M-H]-

[M+H]+

[M-H]-

[M-H]-

[M+H]+

[M+H]+

[M-H]-

[M-H]-

[M]+

LysoPC 18:3

Sibiricose A5

Lipids

LPC

-

1

2

2

1

1

3

1

1

3

1

1

1

3

2

3

3

2

1

1

2

3

3

2

2

3

3

3

1

3

1

3

3

3

3

2

2

2

1

3

1

3

2

3

3

1

3

2

856830

131010

94944

Phenolic acids henolic aci1d07912-97-0

O-Feruloyl-D-sucro Phenolic acids henolic aci1d18230-77-6

LysoPC 18:2

Lipids

Lipids

LPC

LPC

-

-

-

30847000

17522000

9

soPC 18:2(2n isom

etin-3-O-(6''-acetyl

Gypenoside XVII

oniferyl alcohol-4-

Flavonoids

Terpenoids

Others

Flavonols

Triterpene80321-69-3

Others 107870-88-2

3046800

591760

87397

oresinol-4-O-glucLoignans and Coumarin Lignans 41607-20-9

feryl alcohol-gamm Phenolic acids henolic acid

-

-

-

-

635830

5310600

3117800

663140

458060

99782

soPC 18:1(2n isom

LysoPC 18:1

Lipids

Lipids

LPC

LPC

cin-7-O-saccharic a

Flavonoids

Flavones

arinic acid-3'-O-glu Phenolic acids henolic aci9d10028-78-3

iciresinol-9'-O-gluLcignans and Coumarin Lignans 63358-12-3

iferyl alcohol-4-OLignans and Coumarin Lignans

-

100500

495200

1341800

126220

4265600

74060

ciresinol-4'-O-glucLoignans and Coumarin Lignans 143663-00-7

LysoPC 18:0

Lipids

Lipids

LPC 19420-57-6

soPC 18:0(2n isom

LPC

-

-

-

-

-

-

-

lariciresinol 4-O-gLlignans and Coumarin Lignans

y-3-methoxy-8-OL-4ignans and Coumarin Lignans

feoylquinic Acid M Phenolic acids henolic acid

uloyl quinic acid g Phenolic acids henolic acid

loylquinic acid-O- Phenolic acids henolic acid

145480

15864

10764

LysoPC 19:1

Lipids

LPC

30132

-5'-Diphosphate-Dcleotides and derivatiivdes and der3616-06-06

chelogenin-4-O-gLluignans and Coumarin Lignans 33464-78-7

138610

262570

137400

9

ypinoresinol-4'-OL- ignans and Coumarin Lignans

Prupersin C Phenolic acids henolic acid

-

-

ypinoresinol-1-OL-Gignans and Coumarin Lignans 81495-71-8

Olivil-4'-O-glucosiLdignans and Coumarin Lignans 76880-93-8

38313

291370

63161

LysoPC 20:4

soPC 20:2(2n isom

LysoPC 20:2

Lipids

Lipids

Lipids

LPC

LPC

LPC

-

-

-

-

-

-

-

-

39306

531130

355650

471610

430830

48820

yl-α-D-(3-mustard

Phenolic acids henolic acid

-O-Sinapoylsucros Phenolic acids henolic acid

yl-α-D-(6-mustard

LysoPC 20:1

Phenolic acids henolic acid

Lipids LPC

isolariciresinol-9'-LOignans and Coumarin Lignans

Nudiposide Others

poylsinapoyltartaric Phenolic acids henolic acid

1025400

247740

21479

Others 62058-46-2

-

Glomeratose A

in-3-O-(6''-malony

n-3-O-(6''-malonyl

Phenolic acids henolic aci2d02471-84-9

511430

12300

Flavonoids

Flavonoids

Flavonols

Flavonols

-

-

3098.8

1097900

120870

108180

e 5'-diphospho-D-gcleotides and derivatiivdes and der 133-89-1

esinol-4'-O-glucoLpignans and Coumarin Lignans

ide [Cyanidin-3-O- Flavonoids nthocyanidi63535-17-1

-

 

[M+H]+

[M+H]+

[M+H]+

[M+H]+

[M-H]-

[M+H]+

[M+H]+

[M+H]+

[M-H]-

[M+H]+

[M+H]+

[M-H]-

[M]+

Lannaconitine

exin-2""-O-galacto

texin-2''-O-glucosi

3-O-apiosyl(1→2)g

iphospho-N-acetylg

-O-(4''-O-glucosyl)

olin-7,3'-di-O-gluc

pferol-3,7-di-O-glu

cetin-3-O-robinobi

olin-7-O-gentiobio

Forsythiaside J

Alkaloids

Flavonoids

Flavonoids

Flavonoids

Alkaloids

penoid alkal32854-75-4

noid carbon

noid carbon61360-94-9

Flavonols

3

3

3

3

3

3

1

1

1

1

3

3

3

2

3

1

3

3

3

3

1

1

3

3

3

3

1

1

1

1

3

1

3

1

2

2

1

1

3

3

3

3

3

1

2

3

3

161260

720890

47012

2812.5

17909

13462

2857800

2806300

8257

-

-

Alkaloids 528-04-1

Flavonols 59262-54-3

Flavones 52187-80-1

Flavonoids

Flavonoids

Flavonoids

Flavonoids

Flavonoids

Flavonols

-

Flavonols 52525-35-6

Flavones

-

-

2654400

105100

33420

19045

2649400

18046

494920

81768

72980

61269

27425

151930

382610

94982

9

Phenolic acids henolic acid

-neohesperidoside(

-O-(6''-O-p-couma

3-O-(6''-O-caffeoyl

3-O-(2''-O-glucosyl

Flavonoids

Flavonoids

Flavonoids

Flavonoids

Flavanones13241-33-3

nthocyanidi

nthocyanidi

-

-

[M]+

[M]+

nthocyanidi38820-68-7

[M-H]-

[M+H]+

[M+H]+

[M+H]+

[M+H]+

[M-H]-

[M-H]-

[M-H]-

[M+H]+

[M+H]+

[M]+

Oxiglutatione mino acids and derivaticids and de27025-41-8

nin-4'-O-gentiobio

in 3',4'-di-O-glucos

-3-O-(6''-galloyl)g

Maplexin H

Flavonoids

Flavonoids

Flavonoids

Tannins

Chalcones

Chalcones

-

-

Flavonols 53171-28-1

Tannin 357621-91-

erpene Sapo465-99-6

erpene Sapo3391-80-8

Hederagenin

Terpenoids

Terpenoids

nolic acid-3-O-gluc

inol-4'-O-(6''-acetLyignans and Coumarin Lignans

Nitensoside B Lignans and CoumarinCoumarins

-

-

-

-

-

-

oxyGenistein-O-rh

O-(6''-O-p-Coumar

Flavonoids

Flavonoids

Isoflavones

nthocyanidi

Flavonols

9

10695

551400

13522

540140

555400

27641

310520

50422

4097900

20815

13511

262970

271230

294380

335040

350450

452790

49223

399360

638280

493830

35336

[M+H]+

[M+H]+

[M+H]+

[M+H]+

[M+H]+

[M+H]+

[M-H]-

[M+H]+

[M+H]+

[M+H]+

[M+H]+

[M+H]+

[M+H]+

[M+H]+

[M]+

kaempferol-3,6-O-D Flavonoids

Rhodioflavonoside

ykaempferol 6,7-D

3-O-sophoroside (B

-6-C-glucoside-7-O

Ginsenoside Rh4

Flavonoids

Flavonoids

Flavonoids

Flavonoids

Terpenoids

Flavonols

Flavonols142674-16-6

Flavonols 18609-17-1

noid carbon

-

erpene Sap1o74721-08-5

-O-(4-O-β-D-gluco Phenolic acids henolic acid

-

-

-

-

-

-

-

-

-

-

-

-

-

cosyl chikusetsusa

Gypenoside LXXⅥ

Gypenoside Jh1

Terpenoids

Terpenoids

Terpenoids

Terpenoids

Terpenoids

Flavonoids

Flavonoids

Flavonoids

Others

erpene Sapo

erpene Sapo

erpene Sapo

erpene Sapo

erpene Sapo

Isoflavones

Flavonols

ginsenoside F1

ginsenoside Rh1

thoxyGenistein-4',

uletin-3-O-rutinos

3-O-(6''-O-caffeoyl

Melezitose O-rham

7-O-(6''-sinapoyl)g

Ginsenoside Rf1

ginsenoside Ki

nthocyanidi

rides and A

Flavones

[M-H]-

[M+H]+

[M+H]+

[M+H]+

[M+H]+

[M-H]-

Flavonoids

Terpenoids

Terpenoids

erpene Sapo

erpene Sapo

c acid adenine dinucleotides and derivatiivdes and der 53-84-9

2'-Acetylacteoside Phenolic acids henolic acid94492-24-7

 

[M-H]-

[M-H]-

[M-H]-

[M+H]+

[M+H]+

[M+H]+

[M+H]+

[M-H]-

[M-H]-

[M-H]-

[M-H]-

[M-H]-

[M-H]-

[M-H]-

[M+H]+

[M+H]+

[M+H]+

[M-H]-

[M+H]+

[M+H]+

[M+H]+

[M+H]+

[M-H]-

[M-H]-

[M+NH4]+

[M+H]+

[M-H]-

[M-H]-

[M-H]-

[M+H]+

[M+H]+

[M+H]+

[M+H]+

[M-H]-

[M-H]-

[M-H]-

[M-H]-

[M+H]+

[M+H]+

[M+H]+

[M+H]+

[M+H]+

[M-H]-

[M+H]+

[M-H]-

[M+H]+

[M+H]+

Nystose

Others

Others

Others

Lipids

Lipids

Lipids

Lipids

rides and A13133-07-8

rides and A 470-55-3

rides and A34612-38-9

1

1

3

1

1

1

1

3

3

2

2

3

3

3

1

3

3

3

1

2

1

1

3

3

2

1

1

1

3

2

1

1

3

1

1

1

1

1

1

1

3

3

1

1

1

2

1

146740

52618

Stachyose

D-Maltotetraose

oyl-rac-glycerol-di

ylglycerol-2,3-di-O

ylglycerol-1,3-di-O

oyl-sn-glycerol-dig

20502

ree fatty acid

Glycerol este

Glycerol este

ree fatty acid

-

-

-

-

175890

13550

31912

12274

inol-4,4'-O-di-O-gLlignans and Coumarin Lignans 63902-38-5

Specnuezhenide Phenolic acids henolic acid39011-92-2

olariciresinol diglLuignans and Coumarin Lignans 257930-74-8

17972

144050

121060

26266

affeoylquinic acid- Phenolic acids henolic acid

roxypineolin DiglLuignans and Coumarin Lignans

elogenin-4'-O-genLtignans and Coumarin Lignans

vil-4,4'-Di-O-glucLoignans and Coumarin Lignans

-

-

-

-

-

-

-

-

248140

684460

105330

1696200

215310

175990

9918300

21130

ikusetsusaponin L

notoginsenoside T5

ginsenoside Mc

Terpenoids

Terpenoids

Terpenoids

Terpenoids

Terpenoids

Terpenoids

Terpenoids

erpene Sapo

erpene Sapo

erpene Sapo

erpene Sapo

d-3-O-xylosyl(1→3

Ginsenoside Rg5

Ginsenoside Rg6

ginsenoside Pk1

erpene Sap1o86763-78-0

erpene Sap1o47419-93-0

25461

erpene Sapo

erpene Sapo

Flavonols

-

-

-

1049800

184980

29851

ikusetsusaponin LM Terpenoids

erol-3-caffeoyldigl

Notoginsenoside R2

Flavonoids

Terpenoids

erpene Sapo80418-25-3

339650

74978

d-3-O-glucosyl(1→ Terpenoids

erpene Sapo

-

hikusetsusaponin L

Ginsenoside F2

Terpenoids

Terpenoids

erpene Sapo69359-06-4

erpene Sapo62025-49-4

31422

577090

92063

n 5'-Adenosine Dipcleotides and derivatiivdes and der 146-14-5

Ginsenoside Rg2

Majoroside R2

Terpenoids

Terpenoids

Terpenoids

erpene Sapo52286-74-5

773100

80937

erpene Sapo

erpene Sapo

-

-

otoginsenoside Rw

14426000

173720

29112

hikusetsusaponin IV Terpenoids

erpene Sapo51415-02-2

glucoside-7-O-(6''-f

Zingibroside R1

Ginsenoside Rg1

Sanchirhinoside A4

Ginsenoside Rf

Flavonoids

Terpenoids

Terpenoids

Terpenoids

Terpenoids

Terpenoids

Terpenoids

Terpenoids

Terpenoids

Terpenoids

Terpenoids

Terpenoids

Terpenoids

Terpenoids

Terpenoids

noid carbon

-

erpene Sapo80930-74-1

erpene Sapo22427-39-0

8583700

1303900

982110

103410

28652000

204440

431040

1681100

70596

erpene Sapo

-

erpene Sapo52286-58-5

erpene Sapo69884-00-0

erpene Sapo58546-61-5

-pseudo ginsenosid

tsusaponin Iva met

tsusaponin IV meth

ginsenoside 26

erpene Sapo

erpene Sapo

erpene Sapo

erpene Sapo

erpene Sapo

-

-

-

-

-

quinquenoside L9*

Ginsenoside ST-3

Yesanchinoside D

Vina-Ginsenoside R

Phanoside

1196200

6809900

134290

2935.7

45465

erpene Sap7o84213-14-5

erpene Sapo

erpene Sapo80321-63-7

-

Notoginsenoside Fd

 

[M-H]-

[M+H]+

[M-H]-

[M+H]+

[M-H]-

[M+H]+

[M-H]-

[M-H]-

[M-H]-

[M-H]-

[M+H]+

[M+H]+

[M-H]-

[M+H]+

[M-H]-

[M-H]-

[M-H]-

[M-H]-

[M-H]-

[M-H]-

[M+H]+

[M-H]-

[M+H]+

[M+H]+

[M+H]+

[M-H]-

[M+H]+

[M+H]+

[M-H]-

[M-H]-

[M-H]-

[M+H]+

[M-H]-

Notoginsenoside Fe

quinquenoside L10

Notoginsenoside R1

Terpenoids

Terpenoids

Terpenoids

erpene Sapo88105-29-7

erpene Sapo

erpene Sapo80418-24-2

erpene Sapo

erpene Sapo52286-59-6

3

3

1

1

1

1

1

3

3

1

1

1

1

1

2

1

1

1

1

3

2

3

1

2

3

1

1

2

3

1

3

1

1

3896.7

493950

1475900

179540

853980

1928900

84967

-

ikusetsusaponin FK Terpenoids

-

Ginsenoside Re

Ginsenoside Rd

Notoginsenoside K

hydroxy-23-oxoole

Tarasaponin III

Terpenoids

Terpenoids

Terpenoids

Terpenoids

Terpenoids

Terpenoids

erpene Sapo

erpene Sapo

erpene Sapo

erpene Sapo

-

-

-

-

2080400

141750

46698000

1300100

1103200

2138800

2694300

18715

Ginsenoside Ro

erpene Sapo34367-04-9

erpene Sapo

erpene Sapo26020-29-1

Chikuselsusaponin V Terpenoids

-

Calenduloside H

Saponin V

Terpenoids

Terpenoids

erpene Sapo

erpene Sapo

-

-

ikusetsusaponin LM Terpenoids

Majoroside F1

Notoginsenoside N

Notoginsenoside M

ihydroxy-23-oxoo

Acetyl-ginsenoside

Notoginsenoside L

Armatoside

Terpenoids

Terpenoids

Terpenoids

Terpenoids

Terpenoids

Terpenoids

Terpenoids

Terpenoids

Terpenoids

Terpenoids

Terpenoids

Terpenoids

Terpenoids

Terpenoids

Terpenoids

Terpenoids

Terpenoids

Terpenoids

Terpenoids

erpene Sap1o14128-16-4

erpene Sapo

erpene Sapo

erpene Sapo

erpene Sapo

erpene Sapo

erpene Sapo

erpene Sapo

-

-

-

-

-

-

-

18938

79653

5197.8

428870

33413

84140

enoside Ro O-Pent

Stipuleanoside R2

Tarasaponin IV

6353

erpene Sapo96627-72-4

erpene Sap1o56980-31-3

80008

86685

y]-20-[(6-O-alpha-L

Notoginsenoside Rb

Yesanchinoside E

Elatoside D

erpene Sapo

erpene Sapo

erpene Sapo

erpene Sapo

-

-

-

-

141690

6325700

383160

64324

Notoginsenoside A

Quinquenoside R1

O-acetyl-O-gluco

lonyl ginsenoside R

Notoginsenoside Fc

erpene Sap1o93895-21-5

erpene Sapo85013-02-1

86672

132990

150840

186090

64065

erpene Sapo

erpene Sapo

-

-

erpene Sapo88122-52-5

 

metabolome.

YYA2

YYA3

XLA1

XLA2

XLA3

DYA1

DYA2

DYA3

mix01

2060000

3639300

3163600

4011100

1495000 3118600 1383100 1712600 5251600 5064500 4362500 2.55E+06

4418500 5226200 4173800 4822600 4789500 5501000 5469100 4.28E+06

3997000 4554900 3889100 4317700 4445100 5172800 5117600 3.88E+06

2102400

928280

1175900

902200

880770

1002400 1769800 1.47E+06

29394000 35538000 41574000 32905000 38396000 39164000 44663000 44411000 3.54E+07

149430

178450

1838100

450580

46828

316850

321400

307840

286000

323550

292750

260830

240440

359250

356980

455810

463190

373210 3.01E+05

322940 2.71E+05

2905000 2634900 2924000 2510500 3117900 3931400 3101300 2.94E+06

389480

32714

460490

37760

406840

40267

437220

28375

475320

30260

516440

30462

863330 4.78E+05

70138

19013

4.10E+04

2.52E+04

8420.1

15598

25201

43361

37741

19064

23174

523830

101330

6571900

318190

423930

215070

190370

200840

359540

113380

273630

180890

354640

199410

278080

291880

184070 3.20E+05

178940 1.89E+05

5943600 3357500 2550200 2374900 2823100 1560200 1805400 3.68E+06

308900 227620 202410 225560 206250 188700 206720 2.36E+05

28039000 25126000 44296000 21489000 23565000 54770000 51309000 55310000 3.70E+07

1445200 1645600 2413200 2331500 2510500 1490400 1547100 1925400 1.68E+06

17270000 17734000 23638000 17800000 18679000 8411300 13289000 14849000 1.57E+07

17587000 18682000 24394000 18546000 20186000 8838700 13150000 14741000 1.64E+07

251680

774960

1217100

4326300

23163

239680

441110

781910

220480

577730

141190

372280

238820

397270

147130

538950

116120

565480

144510 1.80E+05

787630 5.73E+05

1900300 1175200 1334900 1829100 2025800 2824500 1.37E+06

4355400 5293100 4814100 3763900 4522100 4320700 4032800 4.79E+06

53512

15817

70007

28205

59650

322820

67682

21788

46962

176230

908640

50871

43407

45530

260210

7028.8

3027.9

76724

13862

35984

11081

63673

165710

931110

59251

55018

67363

160300

11890

10006

60053

8562.9

118100

229780

36228

12224

64752

4.94E+04

4.73E+04

1.02E+05

37791

36916

139700

172930

172500

313600

1136700

100630

101120

108280

228270

7006.7

3349.8

109640

1006900

93844

208280 1.71E+05

1142400 1402900

1896500 1220300 1.14E+06

53261

47710

65492

159970

18089

25793

137810

116470

162410

122130

7497.4

9438.8

127680

121350

132450

193380

11431

128240 8.58E+04

135510 7.26E+04

135090 8.74E+04

117560 1.82E+05

64652

99166

180530

5116.7

3312.3

1638100

3372800

3526100

126640

68641

12529

10631

7.64E+03

9.36E+03

13620

4165500 6852500 2423300 4789000 5883000 4376500 4101400 4.39E+06

5607000 8122400 3377400 5443100 6442000 5101600 5161600 5.83E+06

6627500 8710100 3438500 5379800 6604600 4975900 5302300 6.00E+06

130610

40776

68256

60114

39183

60325

45363

77120

84808

124360 1.02E+05

153300 8.82E+04

106650

174660

146010

9971400

4710.8

1791500

7190800 13576000 6286100 7154500 25212000 24964000 22680000 1.36E+07

10728 14699 8446 7687.7 36226 56762 38010 1.60E+04

1309400 2820000 1105800 1308400 4537000 4432600 3667700 2.43E+06

26997000 21619000 36124000 19772000 24178000 67117000 64177000 59925000 3.67E+07

2340500 1715400 3329400 1518100 1865600 5479400 5436100 4498900 3.06E+06

28103000 22368000 36765000 20293000 23721000 67507000 64327000 61126000 3.72E+07

 

27721000 22675000 37206000 20657000 23984000 68824000 66795000 63426000 3.74E+07

1720700

301860

3328500

351190

341040

11747

1224100 2457600 1148400 1249200 4450300 4453100 3933200 2.42E+06

307630 448430 329940 528840 186340 246040 450330 3.24E+05

3020600 4630800 3316400 5617300 1790700 2472800 4679800 3.25E+06

312980

337330

30470

492700

507880

31202

31967

39115

46481

350780

365490

16108

32758

33654

30899

583990

647400

61917

88962

40536

39932

227090

205240

26871

269720

240290

21133

479700 3.80E+05

505820 3.77E+05

30384

26823

28034

2.63E+04

1.93E+04

2.73E+04

19725

24349

15029

10895

18443

17013

20568

46260

80589

144560

361730

204990

178500 1.28E+05

2887000

317940

4848300 7024500 4729800 5348500 3455700 3475300 4223300 4.38E+06

521460 763740 489800 602530 438030 366260 482710 4.68E+05

27870000 38521000 48783000 31666000 43844000 36688000 39645000 32489000 3.73E+07

1037700

317760

711590

300710

163350

78723

1317600 1624700 1085700 1546300 1318600 1423700 1217700 1.25E+06

140470

554860

149840

206070

117700

101930

157130

78639

227310

665410

232270

411600

78134

212560

314610

195070

246380

108880

96136

183350

571750

174450

421490

89889

793750

353060

742850

159520

105900

76370

738730

563080

663770

240240

110710

89770

487450 3.18E+05

747530 5.39E+05

488050 3.15E+05

347610 2.47E+05

38790

44640

6.49E+04

7.14E+04

59596

62885

78782

142690

55910

51812

48242

40338

268510

126220

289170

19095

192930

81902

374910 1.51E+05

166570 5.59E+04

340460 5.05E+05

20562

14578

12443

499750

25776

545610

35043

608300

112920

203730

226850

83184

550740

76948

657490

113240

190970

204310

52342

455120

60024

49256

6.11E+04

104580

141690

64588

176810

208160

101990

193460

189270

49427

133480

120290

80911

142360

139730

66425

187180 1.57E+05

158500 1.59E+05

57553

7.07E+04

7106700

32770

4354700 7980700 8192600 8738500 2889400 4321300 5133900 5.66E+06

99393

404170

113250

291670

92247

623970

355600

25345

155530

655970

490250

23659

238290 2.07E+05

1001200 1.73E+06

649500 4.44E+05

1608200

153550

17151

1721900 3032500 1835500 2362900

303060

38374

685500

39419

349970

38206

621330

46326

25995

2.61E+04

58050

133820

86087

284530

117910

51124

135340

134330

48628

250800

166350

66454

155730

73618

221090

84430

303770 1.72E+05

105180 1.08E+05

146600

18965

32154

27934

34115

25828 3.95E+04

229310 2.43E+05

199420

2295100

59314

141190

514340

389080

182370

149930

172070

3436800 2919800 4771400 4134400 5364900 7008900 4142200 3.68E+06

211960

140950

64692

113600

900700

811260

288690

59764

62651

81313

1.12E+05

3298700

2975000

371050

4447200 2867000 1857200

9180900 8336800 3452200 3.36E+06

8266800 6982500 2854000 2.96E+06

3683600 2267400

312630 353400

939110

356920

302680

395020

296490 3.50E+05

13913000 14935000 19801000 11835000 10504000 15272000 12774000 16151000 1.50E+07

588620

345320

2103100

65590

1064200 1391600

357510 453630

905140

210750

1041500 1426200 1037300 1176400 9.80E+05

309760 426910 606220 636290 3.74E+05

2483200 2399800 2189300 3045300 2383100 2387100 1606000 2.11E+06

51981

72286

54268

84893

40736

87875

36132

50446

49559 5.41E+04

228770 1.93E+05

128640

110910

172690

308530

255060

 

149660

147210

145430

63464

174620

138090

131240

59660

174410

137800

146500

70571

132570

122610

116440

45930

126620

104690

120840

47080

215500

184360

176610

45074

168170

182240

203810

40406

141780 1.66E+05

148440 1.45E+05

166020 1.35E+05

43102

5.33E+04

5191600

3930100

57032

3861000 5241800 3286800 3196500 9866000 7892700 7438500 5.35E+06

2954900 3771300 2542200 2619500 7660100 6476600 5986300 3.94E+06

38745

184980

179310

180240

33601

63309

193480

181240

194300

35453

32838

126630

281800

294850

36221

40116

150880

257870

274580

30580

128830

19423

118040

52086

86135

7.12E+04

69037

123120 9.39E+04

241010 1.88E+05

242190 2.13E+05

123720

141350

67070

114000

94918

143290

132970

72787

58300

52129 5.54E+04

141000 1.38E+05

26697 2.43E+04

226770 4.21E+05

53862

93964

164570

37987

112020

18800

189940

49078

164560

19102

236930

26560

19779

25835

265370

2950200

96069

295190

1002000

561320

887010

148370

258100

4156300 12145000 6341600 10113000 1624500 3739600 2888900 5.30E+06

29158

117350

250060

139200

539390

677310

33709

130540

506400

12749

28908

35036 4.91E+04

305650 3.99E+05

163060

619090

40443

183190

717130

567270

1398800 1027400

60485 56165

1122800 1198400 1240000 1056700 9.32E+05

41585 45570 50294 61121 4.77E+04

20502000 19842000 19869000 19054000 21684000 20568000 21720000 21717000 1.49E+07

18915000 17794000 17355000 17140000 19085000 19377000 18957000 19464000 1.35E+07

141930

12516

9135.7

71987

25189

101950

494000

440310

32674

71150

180730

13820

21285

56682

30109

105770

253820

530980

62854

79330

153690

21890

188130

19455

23192

62669

26275

133560

367720

870140

84525

124250

154910

16178

381180

11452

9429

392420

17676

240190 2.40E+05

30274 1.60E+04

8337.1 1.35E+04

25105

18831

7344.8

65725

110730

29691

76205

50053

26482

706390

51065

527120

40106

74446

81179

38041

5.58E+04

2.65E+04

33980

27917

201610

203220

1143900

152780

125050

134440

396630

1215200

160960

166730

408280

115070

1115800

75208

335540 1.75E+05

291240 2.67E+05

542480 6.90E+05

48843

68647

6.16E+04

9.37E+04

142410

12411000 7728000 18305000 11627000 13882000 18286000 19155000 25689000 1.41E+07

124560

6648400

1458700

148580

113130

52635

99581

42068

96808

93759

106010

100140

110950 9.08E+04

4354100 10586000 6456400 7907200 10294000 11196000 14813000 7.63E+06

2964200 3082100 2637200 2523800 2143300 2264900 2006700 2.24E+06

230370

78732

297360

85427

94408

31852

14379

336940

77205

91832

344140

752680

412250

18019

181020

59172

242590

87214

179060

103940

6936.6

17692

179540

100550

11202

156810 1.74E+05

82648

10916

30420

35184

95748

22181

73351

7.31E+04

6.85E+04

2.57E+04

1.95E+04

1.84E+05

5.80E+04

1.27E+05

52615

123160

40383

86449

51593

34375

44069

17615

17294

26002

32827

20402

33122

20024

94239

104850

120030

224270

177310

380310

307180

23380

247900

58501

308630

104760

126510

324940

750070

224500

16632

125690

16641

180950

42635

63987

450650

130230

307370

165770

23040

150010

170260

361880

171840

24409

26336

68393

218160

451760

191430

18275

184720

468530

263730

17756

555170 2.32E+05

1222000 5.56E+05

266090 2.18E+05

20741

1.39E+04

 

118550

22704

58986

43460

156900

34825

130680

44018

61606

49510

66005

37601

36905

36859

99945

33592

34474

72926

89804

23196

124710

50799

119340

24386

138460 1.23E+05

24008

79327

51113

2.29E+04

8.91E+04

4.10E+04

154540

43444

103010

59722

4419300 12215000 4817000 2690000 3480800 11998000 9841000 7498800 7.68E+06

4217900

76693

7075200 4244400 2863900 3846500 7304600 6163000 5122000 5.07E+06

82418

28128

295420

30213

317630

33234

688920

32376

28816

18237

314730

122360

105010

135070

25041

13661

36832

96062

335760

29049

139720

125760

112740

139240

186140 1.98E+05

106360 6.06E+04

17819

1120300

121730

1043200

42479

1619100 1068800

799920

81165

2058000 1726300 1645000 1.24E+06

145640

611770

52737

104590

318750

27082

60635

167570

23389

7946.9

35469

165030 6.74E+04

667700 3.05E+05

106430

24174

15651

32947

2.83E+04

158870

79366

316080

173770

176990

160350

26568

950970

407250

370510

389240

28187

527410

343110

294540

359470

21273

381600

588620

492550

560830

10528

753710

359420

325160

339910

25490

1763600 5.78E+05

641730 3.36E+05

569820 2.51E+05

629420 3.24E+05

73763

66386

20579

17195

9657

2.24E+04

1.33E+04

2.27E+04

9.33E+04

13639

19247

16284

13741

7555.5

26435

15412

15657

24811

22072

36215

20576

15499

85602

43111

56943

135360

154960

118030

139910

4012500

3819100

3139700

3453400

3333500

40034

5648000 6942300 11035000 11924000 1635300 3204400 8335900 6.51E+06

5502000 6801000 12161000 12653000 1783400 3518300 10118000 7.14E+06

3957100 6309100 10146000 9962700 1470300 3147200 7967600 5.69E+06

5816600 7723800 11883000 12584000 1609600 3530800 11119000 7.48E+06

5609600 7815200 12876000 12208000 1665300 3704200 10549000 7.44E+06

61917

15968

22714

6127.2

186930

60224

76795

9362.6

103890

32212

39212

8874.8

167820

55686

17425

9604.9

39111

13039

73144

11157

98786

50619

144380

10944

89600

35230

69788

6.40E+04

2.25E+04

5.29E+04

11640

11117

5234.1

4232.4 1.11E+04

19015000 9120900 13875000 11831000 12129000 39069000 33870000 28021000 1.96E+07

17380000 7528700 12018000 9904300 9403100 35433000 32560000 23582000 1.63E+07

7676.9

59730

7101.9

86331

55889

16191

54270

93095

298570

626480

15933

108790

69203

8913.4

100110

124220

26582

11541

181140

105880

18547

5829.9

78257

11635

134810

97549

6219.1 7.12E+03

57044 8.46E+04

101910 7.30E+04

36404

106120

33556

6205

22483

31245

32177

98812

2.63E+04

7.61E+04

19690

54043

74794

75218

96704

123200

230780

337790

588830

35240

165140

454520

597710

101520

472930

656640

157320

500870

641840

198580

243300

691440

273920 1.18E+05

400750 3.55E+05

585460 4.50E+05

414850

576210

2388200

2612800

48782

6030100 9334400 8514500 13499000 4220800 4506000 4081400 5.67E+06

5640800 9842700 9629200 14227000 4371900 4909600 4270800 6.22E+06

33918

597590

30847

47229

819730

44572

52206

149040

53738

41825

877020

47853

47785

196870

39468

54135

854650

51104

59834

171660

53220

66235

939750

57406

25691

205260

17422

65796

963330

52940

38126

179160

35216

56764 4.32E+04

779210 7.01E+05

587890

33187

48481

40315

72400

32954

3.80E+04

6.34E+04

1.32E+05

4.93E+04

192410

143910

159270

134380

153230

120480

 

4652600

4705700

5268.4

896010

19399

9558900 5239300 5117300 8505500 6249900 5829600 4307200 6.20E+06

9977500 5678300 5562900 8950100 6297800 5977700 4400800 6.18E+06

4640.4

639540

29882

22114

4652.3

425880

24943

18342

4495.7

365830

22090

15316

6660.7

453240

32508

25916

6585.2

296200

40667

15028

3831.8

423060

42374

16712

4529.1 3.15E+03

587310 5.57E+05

27362

2.48E+04

23196

7357.9 2.27E+04

41568000 33811000 39460000 27206000 21200000 29168000 37334000 34249000 3.17E+07

20230000 16457000 19651000 16213000 14087000 14344000 16905000 16869000 1.64E+07

292080

794250

139540

64945

251160

571760

163440

117260

416970

472310

643790

97780

303000

568140

80869

390150

466130

130980

127370

424280

39408

545690

67736

67135

6358

89924

630160

81984

149790

9

46575 1.31E+05

589250 5.75E+05

40711 1.10E+05

212180 8.54E+04

2.53E+05

112060

491710

104960

786060

404700

2983700

8301.3

26979

9

2916100 4826200 4125400 3777700

868560

19625

35734

13922

48804

2976800 1466900 2.93E+06

11070

9

11634

9

11981

9

14016

41846

24369

33557

18621

293920

13217

58881

9833.1 1.39E+04

19964 5.80E+04

9979.5 2.47E+04

33141 3.80E+04

23361

25836

21591

19894

23864

10586

29794

31714

1935700

18006

2046100 2132600 1858000 1773800 1401600 1862000 1773500 1.77E+06

18585

20087

25409

31375

20164

28597

129110

766990

18970

21238

25786

26408

132720

854250

18658

38173

3712

86472

13674

16583

134700

398510

22352

23835

19673

19489

145650

439670

12727

27353

11690

28886

1.17E+04

2.93E+04

1.74E+04

2.35E+04

28559

14774

10853

28372

20501

25657

26963

106680

763180

4759300

149030

1415600

136980

1295900

128000 1.32E+05

269930 7.62E+05

3006400 5243700 5141500 5626300 2258600 3067000 3969200 3.94E+06

10127000 21416000 31206000 24437000 38627000 15068000 15281000 15235000 2.06E+07

87953

24798

84436

25233

444610

476650

98960

6968.3

20507

56698

115770

37347

458580

456100

40319

9969.1

20100

46468

104560

59748

460530

438480

63227

8444.4

19928

55521

85086

16961

443540

476210

50278

10088

16415

59738

172770

12524

441930

466540

113930

2942

289380

37590

182460 1.28E+05

13377 2.66E+04

398370

430250

41883

478620

446070

100670

7711.7

24640

441420 4.56E+05

415720 4.20E+05

87898

3475

5.88E+04

7.87E+03

6442.3

15156

12144

51483

8803.5 1.67E+04

40009 4.57E+04

41602

81047

907730

790320

472390

71790

1602100 2407600 1511100 1676400 1198200 1837500 1967600 1.41E+06

1631900 2282600 1304800 1582700 1187800 1921900 1989700 1.39E+06

741970

47943

318710

4243

1074800

24732

654970

9759.6

43816

745670

12361

593390

20533

34714

5466.3

77577

821510

34414

21472

6529.5

98252

1063500 6.27E+05

35966

30103

3.42E+04

1.24E+05

89904

74973

71419

6670

4471.5

115560

4752.2

161770

7391.7

223390

6522.2 4.65E+03

79571 9.59E+04

82603

143270

1733700

407290

75875

1664500 1963200 1586300 1753400 1685700 1951500 3221400 1.81E+06

372370

56078

562640

27157

651210

31394

66460

246570

640100

23162

433810

14325

433300

5342.1

62365

517780 4.07E+05

17625

57294

2.58E+04

7.32E+04

206910

128930

233070

135650

195330

73377

76937

38419

240370

256050

384640

311780

260280

 

22250

35864

9

27781

18300

9

28312

40906

9

43867

46087

9

27704

48208

9

9

9

9

1.85E+04

2.36E+04

16082

881970

57228

16773

657820

75290

7457

320280 1.56E+05

90555 1.32E+05

373380

247700

71558

65092

104910

16952000 18495000 23229000 18985000 16589000 22881000 25397000 30370000 1.60E+07

78293

11276

44770

305000

499890

165310

26789

118280

37289

105520

23193

99107

19155

52944

53529

9

83726

66717

9

72456

48705

51267

8.36E+04

2.87E+04

8.68E+04

110440

448010

478710

181110

511790

387940

243850

511490

478450

131940

557360

513170

312260

557990

341270

528290

356630 3.84E+05

458750 1.80E+07

66890000 60772000 67269000 57590000 60502000 62965000 67112000 63537000 4.66E+07

179800

638940

215860

152350

7934400

8067000

792640

567280

83701

348700

839580

360880

280510

38716

149750

325280

285540

47151

134810

318060

218570

864320

583680

115340

86403

25488

180810

223790

172340

887620

495370

111640

71577

30147

94415

34530

115870

361650

328200

659730

403240

54214

24231

81938

8.71E+04

2.73E+05

284410

287360

267620

144560

20759

279910 2.90E+05

260070 2.59E+05

518290 2.27E+06

397560 1.73E+06

7175000 1110800

4194100

592500

337640

86662

583680

91896

82917

60229

2.55E+05

1.39E+05

84253

24069

58204

164170

105230

870770

24156

272620

89685

177560

86333

135050

25381

144990

39794

112230 1.46E+05

37650 8.58E+04

117780 1.05E+06

34837 3.02E+04

6917.4 6.59E+03

44194

113780

2157500

60416

1051800

32774

1153600 1294600

259180

42445

277000

46693

46050

5668.5

35416

15994

10650

5687.6

4807.6

9468.9

3397.3

9031600 13448000 16334000 14768000 16408000 5495300 5560500 3149900 9.59E+06

4813400

28862

6802200

23713

436650

41925

7370.8

7181.1

779800

67663

337800

16105

13898

440060

36626

7172.1

3064.4

903400

69030

211540

18179

9524.1

332960

26771

3852.4

3005.6

670410

69715

229060

16322

11981

186310

11335

7578.4

7236.6

762570

72923

333850

18533

14495

385040

20096

150320 1.73E+06

13548 3.00E+04

4325.9

1975.9

968910

62857

7629.9

6480.3

881970

80231

8004.4

7516.2

1061000

70698

5671.2 5.61E+03

6303.9 5.93E+03

665250 6.74E+05

83714

5.08E+04

308740

14962

264390

15084

320970

17774

446230 3.33E+05

16119

22676

1.45E+04

1.54E+04

13336

12088

13337

3335600

4126000

5208800

138200

32329

2678900 2134900 2540100 2350200 1320300 1252200 2394900 2.05E+06

3464300 2828100 3245600 3066700 1528000 1463000 3001800 2.42E+06

4083700 3120800 3998000 3719700 2051400 1936700 3676400 2.94E+06

216530

21539

104680

21027

255800

21256

176550

22112

126020

13153

192970

10737

100600 1.22E+05

25433 1.93E+04

324410 2.27E+05

12417 7.60E+03

408130 1.93E+06

172260

6286.5

2163400

4700800

1153000

206550

7455.3

296840

8660.7

342180

5776.2

402700

8459.9

256330

4565.1

274510

245760

4899.1

568210

2682000 2722500 1771900 3019500

6393300 7840700 5112700 7061500 6423900 6704100 5381300 6.12E+06

1359700 360580 423050 334090 285040 442970 252360 5.41E+05

109620000 86650000 19405000 22429000 17419000 6307000 11336000 11772000 4.28E+07

104030000 84507000 18588000 22925000 17315000 5931800 10698000 11444000 4.06E+07

158730

194730

166590

300180

142890

53607

78713

64661

1.29E+05

 

18772

26078

46711

9

17340

55919

188640

98297

19679

82335

3951.6

4397.6

73025

11014

116160

182880

110020

23425

18336

94309

155160

92101

17150

85234

4267.5

2634.5

163620

14742

76953

174520

92578

22161

86125

5547.2

3741.6

129200

956950

96941

7153

78187

147570

78336

22485

90655

2058.8

2601.5

154110

8946.6

36718

8953.2 1.89E+04

34670 4.85E+04

125050 1.76E+05

228050

115100

34167

17240

9

7.45E+04

2.08E+04

9

88066

5763.5

5149.1

33256

595410

90465

2567100

16113

16826

27312

85760

93033

105360 8.76E+04

5006.9

2971.2

125740

3826.5

2229.5

145400

25085

21449

99009

6.58E+03

4.55E+03

9.35E+04

1955800 1737100 1705500

101460 97773 106040

2952000 2988500

91157 99015

960470 1.58E+06

119510 5.64E+04

2774100 3131300 3006700 3090200 2624900 2710500 2612100 2.39E+06

16342

27302

32973

7823

36769

43068

7976.7

22720

24881

16390

24370

11214

16520

30942

14283

26371

79372

11290

23090

23837

1.25E+04

2.20E+04

4.71E+04

132740

18464000 21262000 19797000 17274000 22927000 17889000 17923000 11773000 1.80E+07

13004000 15285000 14401000 12199000 16303000 12696000 13335000 8965100 1.30E+07

12277000 13393000 13988000 11809000 15750000 12790000 12014000 8697500 1.19E+07

13130000 15082000 14759000 12478000 16444000 13043000 13477000 9181800 1.33E+07

23293

12042

69453

12534

53686

14683

45484

12693

142670

46166

15933

26094

14952

30322

16712

25559

13218

5.09E+04

1.39E+04

92486

151670

201950

172920

132160

209550

143210 1.54E+05

5508100

12936

7048200 7681400 6241900 7816000 7731200 10351000 7358000 7.15E+06

14770 15614 14661 13839 13635 19494 13894 1.32E+04

2389900 2299100 1996400 2732100 1904900 2085300 1359700 2.06E+06

2096600

774610

32738

1173000 1257300 1182100 1118300

84921 104270 86038 87381

736110

101080

787400

102050

504710 9.11E+05

90788 7.24E+04

7207200 11776000 13336000 11394000 13874000 3734600 4370500 3519700 8.70E+06

24989000 33260000 31521000 25015000 30103000 25601000 35846000 37566000 3.74E+07

207460

982100

172800

3821600

3132200

144190

794570

184590

74287

97802

61901

82647

249690

60947

77661

28422

28209

56493

55830

41793

44700

98956

8.23E+04

2.70E+05

1.28E+05

188680

253620

179140

149350

4947400 4553100 4675700 4902400 4671300 5267200 5924600 5.32E+06

1382900

263170

226920

187810

43466

754930

157790

58952

100120 121470 5.91E+05

1783600 1917200 7.83E+06

27592000 22069000 3783700 2892000 2863400

97302

50594

170900

84690

255620

133080

285960

110160

255640

156150

137440

35822

89295

50315

1.50E+05

7.69E+04

1953400

8441.2

118740

8844.8

17425

1922700 1162300 1899100 1815000 1965400 2008900 1971900 5.48E+04

44595

427880

9947.1

30016

177020

9002.9

27153

99226

55554

9

83266

227950

10469

19636

138260

10813

9

9

9

1.73E+04

163350

13915

32215

134020

12909

29121

195410

122060

97803

11272

38971

78199

6242.6

43565

25338

63024

153550

13070

82772

87784

10677

39626

70166

92361

111230 1.85E+05

9939.7 9.10E+03

37238

25734

62591

3.74E+04

220390

4163.3

33632

165650

8134.7

18927

128850

8207.4

18246

192620 1.34E+05

8009.7 1.04E+04

40989

113590 7.48E+04

56558 6.51E+04

3.20E+04

31476

207940

150060

126970

105340

27397

 

9

3002.3

6484.7

149510

380040

177200

34165

13473

17780

15598

11391

135550

208810

554260

22134

130710

37260

96067

6247.9

70379

9

5529.5

36658

4325.5 5.04E+03

10650 1.97E+04

5246.6

156430

255400

44622

8886

13476

174620

428020

314070

24302

116710

24283

83338

235590

382340

101900

17749

171930

292730

354570

19801

232610

339630

350840

46168

264140 1.83E+05

646010 3.23E+05

69760

29731

1.62E+05

1.60E+04

65850

20669

47290

111360

51593

161970

38419

225230

32257

175140

34863

124950 1.29E+05

32811

88351

3.69E+04

7.75E+04

76617

109750

154200

126350

11938000 4982700 2993900 3281500 3466000 2762300 3160800 6438700 4.63E+06

350330

1829.7

58021

233740

2043.2

171300

191110

27579

59735

4610.1

47044

14352

57982

4106.9

785470

913090

37928

15888

8438.5

14786

40173

6222.7

33455

44363 9.69E+04

3784.9 3.32E+03

94419 2.44E+05

117970 2.87E+05

1313800

1528300

65106

719460

805190

25140

77011

26779

48840

3150.3

19427

122230

310090

256840

252520

265130

163120

430150

456620

394840

396840

17030

45238

38093

29188

42166

5.63E+04

2.29E+05

2.11E+05

2.09E+05

2.48E+05

151610

153280

107450

140220

300770

316920

286700

298280

179020

166890

127560

148460

198170

196640

174560

191330

14321

8686.7

18205

1997200

1957700

4751800

8669600 1101900 9451600 2266900 2893000 2131200 2775700 4.06E+06

8245600 1159300 9397000 2195100 2957700 1934300 2699700 4.00E+06

8559600 7088800 8537500 7305500 2129200 1862600 1531700 5.10E+06

14605000 12258000 15354000 10929000 12219000 13756000 14364000 12238000 9.17E+06

17844

42120

16084

73871

96581

222050

25594

1930.4

14268

62253

72455

290700

20205

7893.6

38868

60502

80811

173760

14892

13409

9135.3

50678

63038

219980

15989

29478

52799

98853

31069

106450

135030

189660

15766

9615.3 1.12E+04

64394

82606

6.62E+04

7.72E+04

53562

122000

266300

24597

229680

25391

285270 3.59E+05

18788

16849

2.84E+04

2.26E+04

14032

3743.7

529490

71988

4047.7

597380

73108

555920

83851

1577600 1606700 1425600 1600000

349950 9.83E+05

40385 1.17E+05

337650 2.04E+05

194840

293140

41237

205840

343950

36124

191380

318140

49793

196200

301580

59886

348380

36707

292960

13639

282280

22068

36268

11479

3.58E+04

1.79E+04

7911.2

531760

118890

206670

14931

12675

11076

9045.5

547700

151420

423950

23201

10494

8997.9

947220

228780

447850

8089.5

15301

397140

330690

436000

28614

586590

145160

366970

17139

725330

258740

367540

23482

503420

218310

611050

17805

971760 5.82E+05

121590 1.82E+05

394800 4.25E+05

18325

2.26E+04

1170700

105930

58498

2059700 2520500 3438200 3754900 1773000 3313800 1216200 2.23E+06

102030

55951

263610

56388

250250

7031

361340

34325

198990

7356.5

26897

88035

56672

48563

176940

38161

169580

8374.8

20536

63058

43322

34574

89098

238620

271370

3533.2

14201

77781

30520

21826

115270

218610

293030

2505.6

18125

66326

47052

38340

173980 1.17E+05

115940 8.63E+04

309880 1.92E+05

3694.8 3.96E+03

183400

2848.6

46669

188730

6567.8

36093

43782

48869

57122

40455

20262

43257

34024

29140

2.96E+04

6.20E+04

9.30E+04

6.70E+04

35098

45204

244030

240530

164500

176360

 

44516

300820

25654

63786

516440

49356

400750

120210

91446

4230.5

9

43536

542980

88733

43792

631760

58698

100300

468420

81110

81315

481460

39765

97911

9

9

9

4.78E+04

49237

11818

31785

129900

23967

56746

108310 3.23E+05

24522

60582

5.00E+04

1.15E+05

257320

160790

161740

1493900 2855000 2077800 2224500 1278700 2494000 3371900 1.64E+06

37833

481220

126580

394970

4989.4

10090

117260

222180

69102

242880

4486.7

20248

14742

48885

472160

122150

417180

3239.2

9024.8

15553

41329

376390

95102

173360

6518.8

11696

33982

35961

227910

66236

34575

5880.2

30630

2381.8

42482

177320

61168

63774

12252

29525

573.53

85367 6.08E+04

111370 3.59E+05

40488

72413

9.19E+04

1.76E+05

5505.8 5.11E+03

2.09E+04

8847.8 1.34E+04

9

28696

5905.8

5142200 10089000 6578000 20956000 15316000 43689000 30638000 16796000 1.61E+07

5265700

107570

165770

341440

4932600

98809

9442000 6220700 20004000 14497000 42495000 30522000 16323000 1.54E+07

146730

229080

473650

163700

209270

335940

179600

262760

616120

174220

142070

420930

178740

92332

185700

49854

140860 1.56E+05

53161 1.32E+05

106990 2.85E+05

200340

206890

9735800 6927500 20560000 16471000 46822000 32408000 17113000 1.71E+07

111010

1004400

4962.5

69316

116730

987380

7969.1

41997

29027

295040

2087.2

54240

24750

230260

185080

35941

184170

4335.7

23633

11152

186350

7651

18565

148320

3343.2

45526

22655

235800

10844

39047

18714

10797

9.76E+04

929620

2343.8

40766

1023300 1411000

433460 8.57E+05

3049.5 7.36E+03

18094

54110

28058

73162

1207.2

52599

15867

4642.4

87889

40043

95125

2491.5

58567

15940

15319

10744

4.51E+04

2.28E+04

13212

18940

167000

1056.9

252800

12642

359980

1646.7

216220

18543

235550 3.08E+05

12655

70097

29710

4.99E+03

8.07E+04

1.64E+04

32072

13984

617980

697220

3786900

6576600

3133400

4027500

31152

1494800 1007900 5363400 4351500 1916400 2397400

1423100 918030 5092500 4892700 1968800 2695200

631180 1.93E+06

672040 2.02E+06

3515300 1990100 2866300 2560800 2036400 1204100 1005300 2.45E+06

5512500 3253200 4740600 3918800 3268700 1808200 1719400 3.84E+06

2845400 1667100 2241100 1923400 1581500 783520 1.95E+06

3425300 2068200 2888700 2242900 1886800 1047000 1056000 2.36E+06

87325 71064 39440 84325 73441 65989 79182 6.54E+04

890150

3123800 16823000 16284000 7144700 15916000 2895400 3913900 9218500 1.09E+07

9

9

9

9

9

47251

92025

112300

70154

180190 3.74E+04

40236 1.19E+05

4661.1 2.76E+04

110720

14601

136050

16347

35334

254510

30510

88737

47614

22487

52392

34997

73771

190530

195610

23153

21044

35032

39527

105320

161230

29924

95798

13157

95994

238140

120680

8846.2

2486.5

63594

8659.9

9234.7

2254.1

106960

302990

31065

52378

16979

25535

409530

6956.8

15349

9.12E+04

7716.6 7.53E+04

18592 1.77E+05

217830

6528900

28731

9585300 15270000 13602000 12759000 8366900 10910000 7940700 9.19E+06

50156 65074 91369 49989 9422.2 14129 25880 5.97E+04

7518200 13640000 11269000 11287000 7269900 10558000 7040500 8.20E+06

4682900

16631000 23834000 39912000 32689000 33117000 20173000 25252000 20546000 2.44E+07

6931.4 38745 74164 47692 114450 88085 132160 8582.4 7.17E+04

 

13839

98440

27911

96979

16237

95746

10761

100930

1444900

14697

104770

831930

19395

93683

30511

90330

21432

1.73E+04

116310 7.97E+04

801470 8.73E+05

925320

3215600

1994500

35279

897970

807840

1355300

964680

3198900 2596700 5212900 3284100 5447200 3566900 2981700 3.24E+06

3463800 1689400 2004000 1450200 9848500 6360000 4343700 3.13E+06

42999

433050

29682

44184

32404

465530

31849

32792

72252

732900

69944

82221

54267

509910

49815

38644

9

9

9

2.86E+04

252120

28027

396250

21652

9

377470

9847.7

9

282250 3.98E+05

5209.7 2.75E+04

28515

9

3.32E+04

2874300

2867200

19447

4608200 4809600 6789300 5535200 1894800 2311200 1313900 3.47E+06

4955800 4530000 6510900 6586300 2060700 2256200 1352100 3.34E+06

34239

65124

43799

68717

45638

39844

203290

34120

59329

15785

78515

242190

405890

834390

87184

19175

16883

8847.2

103140

9

25757

69637

350780

534800

1128000

99375

23146

8863.5

9535.1

165580

9

12500

31390

50499

132330 6.71E+04

243150 3.84E+05

1033300 1.22E+06

721560 1.14E+06

464650

588060

1353100

34006

595990

346240

233600

1536300 3071400 1451300 2373800

2012800 1141700

745470

73041

15638

16234

16996

32066

33066

16510

1059600

54714

22004

871870

826690

64371

17378

13307

22849

24304

31141

11083

952130

57929

32463

646730

43738

29663

10428

8834.8

22993

13040

14221

791280

57700

39830

641010

61305

30037

15306

16038

34427

41810

14343

740570

25539

11507

546830

41337

26663

14467

6.49E+04

2.00E+04

1.30E+04

17164

11188

15103

7752.6 1.30E+04

17001

93987

9

4.01E+04

1.75E+04

21712

21167

10185

403590

56210

9

6969.9

249100

35120

9

8003.9 1.04E+04

402900 6.40E+05

651970

55147

25187

9

4.99E+04

1.44E+04

18625

558120

347550

260070

262890 4.77E+05

12304000 7543300 8766600 6431800 8347000 8938800 7609600 9264400 8.68E+06

13414000 7727300 9378500 9445900 7843200 5707800 5001100 6441800 6.74E+06

7490800

4702500

7378100

4456700 5014800 4605200 4188300 4360400 4710400 5182000 5.60E+06

1996500 353810 574640 489370 254930 291880 325000 1.39E+06

4962300 4228200 7398100 4573300 3913900 4564000 6049300 4.68E+06

14905000 12269000 12754000 17309000 15110000 14940000 13859000 19339000 1.53E+07

12493000 10985000 6475800 13652000 8118400 6122000 7115500 8940400 8.27E+06

648460

14565

1267.8

2604100

27553

369710

9

754990

21883

4706.7

834860

36547

29226

1071600 1023400

453330

9

346080

9

332940 6.80E+05

37060

10936

16168

13322

9

1.10E+04

10401

257600

17042

305330

13293

156760

85429

221900

19949

3237.8

4891.5

6242.4

204290

16971

271910

14467

218670

107090

204710

25700

5102.5

5786

3491.8 8.31E+03

210790 2.62E+06

3729000 3900100 4434100 4459300

55617

216730

9

29045

195440

9

75866

244880

9

31823

282440

9

11047

1.79E+04

272930 2.31E+05

9735.1 5.59E+03

240370 5.20E+05

1328500

9261.8

464760

37337

5886.5

6859.7

743430

23542

564960

43897

8250.2

6277

573170

25869

431430

40944

8420.7

6493.5

390060

24547

588680

42021

3655.5

3149.3

514040

27738

450780

31390

3901.1

6471.9

33468

451280 3.61E+05

43148 3.09E+04

46215

6800.1 6.78E+03

5049.7 3.92E+03

 

288520 5.41E+05

7348700 11555000 14796000 4610700 7757500 15081000 4774800 4383500 7.80E+06

72622 94618 48067 2.26E+04

11171000 17902000 23404000 7210200 12638000 21412000 7070900 6713000 1.16E+07

373420

207190

229710

204350

222930

99784

93177

9

9

9

9

9

190190

16861

51558

10605

24132

13918

260010

4564.8

63379

38854

8289.6

14731

6289.4

358150

8806.7

24887

48982

2243.1

9979.1

7290.6

377670

29456

57811

57404

7430.9

11401

7774.4

358300

15426

95719

13199

916.41

7152

9172.8

3388.8

9343.8

162960

280590

1307.5

10966

15363

6150

4.81E+04

8.74E+03

1.54E+04

24543

13772

11738

150120

306620

356.7

127540 6.20E+04

159390 2.23E+05

2693.6 4.83E+03

205010

5208.2

172920

1941000

8799.3

29032

7.06E+04

2089700 2824100 3594400 2223300 2744700 3575600 1632300 2.38E+06

16811000 23672000 14787000 11061000 10270000 9985500 14124000 6426400 1.42E+07

1453400

1427900

2200000 5016000 2364100 3287100 1281100 1447100 1017200 1.93E+06

2233400 1349900 1035200 787390 234850 454270 134430 1.00E+06

16862000 23116000 14682000 10239000 10251000 9649600 12344000 5991600 1.26E+07

28110

12322

84906

9

33166

21700

37970

9

11391

29980

110780

9

60196

27037

91063

9

25381

32730

98777

9

121830

33279

63260

112370

2419.2

22102

49940

14225

804680

947490

9

264780

38483

53418

84975

2784.8

24739

26101

12904

472430

959000

9

68450

15271

67351

66077

5.21E+04

1.61E+04

74836

2.81E+04

19695

31288

33285

12904

706870

4518200

4307.5

4338.7

81431

21789

232940

27919

45595

33350

24839

952680

58411

30393

36904

3060.1

558240

30268

18020

27167

16475

801510

35338

46816

35748

7347.1

615590

9967.9 2.09E+04

16759

27622

2.61E+04

3.02E+04

6646.7 9.28E+03

302640 6.10E+05

403690 4.03E+06

3871700 5220200 9311900 7228600

6925.9

6033

16544

14171

13430

17923

136710

29852

259860

7051.8

7742.2

114670

90748

9

9

5.02E+03

5.59E+03

9

9

90353

48782

349510

97208

36649

266360

547990

19748

226730

541770

8111.8 6.91E+04

149800 9.24E+04

296520 2.99E+05

126960

135050

217290

10872000 17328000 21787000 13091000 14633000 34723000 11571000 11761000 5.89E+05

559640

50229

1617900 3739100 1377800 1531700

9

9

9

1.03E+06

6.31E+04

64707

1047000

3113.5

83625

24037

534140

3127.3

47278

50843

16948

33864

354400

16704

108110

101950

43699

170510

515590

6755.2

623890

390000

1778.5

9

228600

487120

12283

368070

470720

2288.5

9

88647

769790

1951.6

59300

1481900 1130600

256340 7.65E+05

3836.1 5.38E+03

513530 1.68E+05

381790 2.35E+05

624.49 6.72E+03

2171.7

125410

165480

10663

9653.2

87847

65830

11793

38006

397570

87365

99513

42905

88404

1836.3

37446

5404.2

52652

55027

9

2.83E+04

455430

42131

624370

5655.9

103530

623610

15070

2407000 2231400 1342500 8.21E+05

16286

75209

6011.5

72024

34343

82101

8.65E+04

7.79E+04

120560

1075300

26717

105060

1438100 1207500 1374900 1561500 1482100 1554600 1499300 1.03E+06

84202

96610

48019

50757

33813

41146

35706

38365

9061.6

10196

18100

15242

17103

21242

3.31E+04

2.72E+04

32030

882330

1152100

768540

1806000 1195300 1383800 1116300

604620 9.94E+05

 

101290

109730

62262

9

63219

179860

52417

9

83809

370710

50611

9

79715

317800

26833

9

76615

331350

44160

13265

55418

591480

643310

9

30087

254030

10054

72224

47378

12576

13945

49502

172640

195160

124610

9

33761

156330

31029

76332

38170

5629.1

2368

48800

96004

63182

67578

89376

15425

19181

17380

6.17E+04

2.15E+05

3.94E+04

3.27E+04

8.24E+04

4.62E+05

5.54E+05

1.83E+04

133960

477070

751320

13068

453540

468770

361860

49794

21216

6085.6

277320

307570

159820

9586.4

9

111530

501880

890690

13552

141480

166930

117610

110910

43182

8607

104820

491600

646940

9

83883

985250

1503200

9

36789

95049

113040

80712

9

278980

266450

210160

101500

25053

5718.2

358890

372910

449250

8893.2

9

417910

394930

299280

145640

35741

10918

366400

357340

400110

8245

812360

781190

551520

119850

28446

10872

254190

280450

474920

17845

29086

351770

15842

50333

397640

251830

204930

193530

286590

26714

39083

277490

4484.3

145590

36891

114830

845040 8.05E+05

940080 8.64E+05

573620 6.29E+05

9

5.65E+04

3.77E+04

52489

3866.2

89394

96381

29718

26180

41490

734240

47371

18688

9

53964

6578.3

191030

186500

4014.6

110040

229580

182830

42105

15180

9

15720

6296.5 6.27E+03

176500 3.32E+05

183820 3.59E+05

7985.4 1.85E+05

743380

748880

280800

9011.9

9

18449

18771

84077

22247

63934

83580

3.06E+04

3.38E+04

2.31E+05

3.03E+04

8.51E+04

2.89E+05

9

68047

16768

31026

74793

77324

51211

69050

191550

9

94507

34446

8344

309320

20646

16579

1346900

104640

91878

85045

175760

20649

21873

167390

2784.7

75478

93497

62391

153660

37133

31726

800320

90020

70910

69110

233820

16478

45898

259280

11464

77471

59116

396490

332430

39553

38521

30245

220330

9

123790

59780

92367

19071

64632

9

79802

78144

81204

33725

134270

9

195330 2.27E+05

128680 1.68E+05

153450 1.84E+05

14758

33230

9

1.46E+05

3.10E+04

3.46E+04

1.67E+05

5.13E+03

28914

189680

2524.8

39091

42873

66980

37882

238840

2595.9

62789

176080

71713

19098

3113.8

87046

7398.5

105120

29481

2151.6

105890

28534

69864

25513

9

143880 1.04E+05

79684

54269

5.55E+04

9.17E+04

7066600 13934000 7117500 7101000 5921800 2188400 3880200 6037400 7.71E+06

999790

45284

1978900

88418

991230

46220

29788

34063

21824

28694

51302

31641

536220

582040

536330

370510

591540

62640

34073

31382

26826

25055

14851

34169

798560

619870

718950

500150

573810

44368

242500

289230

234770

230940

224620

226960

22273

409440

342820

9542.4

8206.2

5967.1

6003.6

16204

625190 8.87E+05

120860

6265.2

6080.1

5730.1

6288.4

33872

104940

289770

258750

272900

275840

3.24E+04

246380

988390

993100

975730

11257

14945

854160

730430

711820

824660

28077

12054

17124

10450

15145

11120

14250

39036

51237

7012.7

334160

286080

316820

1027500

9018.1

581360

500030

594130

564640

682560

195540

5328.9

820850

710150

856890

132030

7019

1700100 1395500

1343100 1130700

1280400 1374100

764640

686200

681810

333730

242820

153450 4.91E+05

 

69615

77738

9318200

29184

24744

9

30504

63283

31394

31119

34695

55506

50438

71016

482460

612140

1253900

1649800

66955

91795

2.16E+05

1.96E+05

4064300 3961600 2871500 3538900 5561400 4780700 6194000 4.20E+06

28155

34115

9

47500

40404

52177

49878

9

27878

30408

9

9

9

2.16E+04

3.00E+04

3.92E+04

3.10E+04

9

9

9

58644

9

9

9

150760

9

131720

9

29906

7991.8

477320

179300

477800

97335

117640

249810

111900

38394

6742

54966

21591

143190

45748

161850

29672

42107

75143

19906

50397

14137

45741

264470

549790

4207.7

34245

75658

2443.8

14586

84495

78224

61823

73525

15212

81449

15918

58364

39544

34028

29249

293820

136370

375780

84172

74676

210760

107850

21900

26384

24378

145740

783730

11632

502160

78416

11472

303680

761830

741630

84536

106170

341710

737550

399180

707840

22236

28158

27227

7649.8

221490

67497

204960

34681

69758

72259

41177

12847

209360

13374

52294

9

4193.5 2.30E+04

794540 1.16E+06

335800 5.44E+05

850490 1.43E+06

163370 3.40E+05

216100 4.01E+05

411170 8.24E+05

202580 3.48E+05

266030

104500

326720

48968

3042500

1498200

3360500

878920

973580

2008500

1017900

12943

127240

28125

195610

36270

47481

57047

39946

31925

182460

28719

72613

755810

5882.4

18755

279230

5068.8

90071

123140

146850

36324

44723

67006

109450

64694

120450

88389

119200

75557

32913

19468

81540

11184

71704

827490

2277

3.56E+04

4.79E+04

2.44E+04

1.66E+05

1030100

8222.5

16868

27560

17634

137250

345330

4648.2

32897

49322

2659.5

9390.1

86343

102650

207610

210290

21054

95660

25635

78134

1775300

54069

105320

31906

76075

1277800

18331

31713

19642

162300

604510

3965.2

277220

128620

4935.6

497420

901880

1086300

129080

131030

496050

945910

718310

949380

174430

1629500 1217700

17000

5193800

87261

27242

138460

53847

29110

24532

48390

86798

83323

71083

48450

73034

44306

60123

817640

69923

681370

26575

76945

37667

478080

838430 1.98E+05

14892

2431.8

71427

249930

255610

53931

59740

81149

230230

81917

172450

7016

675310

2953600

2716400

86323

139510

447970

476700

70680

74959

94378

662440

2702600

872810

2712700

161340

401070

152400

417240

2513600 1898900 2742400 2099800

1108100 1421900 1.76E+06

149810

94540

95521

66129

45407

186400

58856

146760

44679

77910

63949

53647

158300

66241

753610

37748

46284 8.29E+04

530140 2.52E+05

23419 3.52E+04

123290 1.01E+05

47831

185870

120970

100810

1771500 2247500 1438100 1844000 2318500 2150200 1864300 1.74E+06

12445 18096 26139 31882 104370 122480 56132 4.12E+04

11760000 26252000 15773000 14319000 15624000 8599900 10679000 12145000 1.47E+07

2924900

15712

19223

9

5037100 3804300 1994200 2060200 1152000 1982000 2029300 2.92E+06

16372

22787

9

47045

24089

9

24483

96306

9

44253

78946

9

26850

13992

34691

22288

32972

9

2.42E+04

3.21E+04

16007

48580

44408

9

30994

397140

443050

44707

179880 9.47E+04

190280 7.65E+04

9

9

14520

27383

27061

34543

26062

12700

9

4.11E+04

 

9

9

9

9

392390

394070

323140

9670.8

161260

211600

61555

38180

42702

31871

22896

109170

128550

38145

27123

24756

9263.5

346080

62021

45814

25427

31922

31870

33544

162050

244820

68898

30539

28903

20326

654160

101020

3136.5

148390 8.48E+04

162990 7.98E+04

134980 5.90E+04

9

9

9

22542

33999

9457.2

214780

277310

63217

49215

47382

35017

18088

4741.8

196170

330250

124120

57856

90070

21489

1101300

213720

5464

13526

17034

444100

590400

204660

23646

14523

54728

17321

11249

224570

334540

66963

37699

24628

33360

10609

1.01E+04

166550 2.72E+05

259730 3.17E+05

58351

9.97E+04

159570

137880

27193

117350 8.16E+04

109710 8.56E+04

28364

2.53E+04

2289200 1064200 1147700

873330

130700

33679

590050 1.19E+06

459060

5976.6

187500

5920.2

117860

20651

81620

1.89E+05

12977

2473.4

1556300

19816

16996

211.31

18065

37502

101630

132200

89803

37573

20277

38846

57035

3555600 2856400 1424400 2324500 2424100 2098500 1727100 2.15E+06

15788

16033

691.32

11392

35339

158380

434960

199980

28780

13940

27063

102080

16857

13629

12744

13960

9066.6

71207

227900

136740

29084

23721

17625

46584

24939

46721

4496.6

15877

13013

111830

174370

185830

51412

65470

57322

108820

14883

83481

26416

22668

9225.9

62665

135840

161940

31936

30169

23936

41766

9298.3

78560

427.64

13166

3859.7

19022

50123

63437

9

9

27010

13595

110.21

19442

31459

31456

4542.8

29310

21925

118.56

9

5607.9

13062

75653

85911

9

6979.5 1.86E+04

9057.8 6.20E+04

139160 2.12E+05

87572

1.54E+05

2.43E+04

2.78E+04

2.91E+04

9

25372

9

24385

9

26842

9

26566

11789

4818.7 5.80E+04

28255000 45328000 23373000 23724000 21527000 8269000 14834000 21851000 2.69E+07

2187800

178200

771130

74552

3537400 1624200 1004300 1184500

488150

11399

1087300

37253

968990 1.70E+06

59137 1.95E+05

252060

524760

32695

313730

341890

258700

1617800 1664900 1131500 2903700 1949700 1092300 9.97E+05

74252

13378

102380

24232

92284

20995

54184

77154

10977

49767 4.91E+04

9775.3 3.23E+04

115980

1128900

946590

194780

49874

55507

6681.5

1020600 1914600 1616800 1656200 3529700 2034800 1530200 1.77E+06

525820

155640

109600

107170

129370

57344

441450

128190

66911

79074

60774

79069

54445

66686

59688

43014

123390

6997.8

4111.9

766240

517680

300400

67157

60031

66047

28124

73531

42922

52083

35785

51581

10617

10101

650990

450740

162750

45569

45015

34185

44855

33457

57441

49144

22042

57217

10175

4998.1

562640

1787300

129470

24890

29589

32243

22530

40018

30376

31850

22318

68028

3122.4

3207.1

203190

980760

129600

58787

53664

34101

25947

54805

37703

64268

30496

90088

5612

494960 5.69E+05

68282

57706

42489

41139

58953

45074

46188

26238

18913

49681

1.86E+05

6.13E+04

6.34E+04

7.80E+04

3.85E+04

7.16E+04

4.00E+04

5.51E+04

4.27E+04

1.20E+05

47176

80928

49841

87124

130510

26895

63306

63751

117020

99787

49339

131950

19400

212620

13176

7269.4 9.09E+03

5385 1.37E+04

443950 6.68E+05

10520

4823.9

840950

5141.9

337750

726290

 

688770

67347

32172

867530

105140

83040

739130

62931

67910

572250

140570

107240

473280

137780

110750

203510

186340

189940

321240

221700

127830

386480 6.27E+05

70667

67751

1.00E+05

8.07E+04

24733000 28203000 18043000 13816000 13593000 5670800 10806000 11272000 1.82E+07

11974000 25058000 13166000 11342000 13383000 7302400 9951200 9325200 1.44E+07

9

9

9

9

16368

6089.9

7188.1

65203

607090

9

8.88E+03

2332400

5.82E+05

3492100

598030

78866

3325900 2748900 5858700 2191100 1514200 1742300

1006700 1303400 1319000 1159400

9

25748

9

9

33512

9

88519

175600

499910

123940

126690 1.48E+05

633860

3315800

2297400

669440

373000

68698

980370

1375400 1253500 1101600

9

5.75E+05

7823600 3741400 3791900 3742600 2097000 2841200 4187200 4.02E+06

3604900 1651400 1200400 1648400

1129500 1654400 2425000 1062000

578380

141430

259410

355960

402250

266650

220990

15875

929860

35965

1175800 1.78E+06

246890 9.87E+05

124820 6.17E+05

168210 1.47E+05

168710 1.72E+05

122850 7.66E+05

599660 6.19E+05

575670

103910

84674

376220

88725

2066500

264060

296030

3018600

613110

29976

655850

119860

101390

951580

632470

33482

111720

308390

324950

140420

379600

32021

87170

82135

474250

640770

63270

636920

1382400

125380

619200

758250

68890

36877

4.20E+04

4472300

60334

4833500 3788000 6370900 4176300 3165800 3383700 3633000 3.76E+06

49909

479730

23642

24263

30253

147170

445330

147620

9

38489

210030

12417

15944

38087

138430

184130

179670

9

59583

220630

16737

11342

45399

69895

1199100

393660

9

47535

117790

6415.3

8021.4

36170

116360

595120

214970

9

61910

53300

9

58456

58008

9

35677

14185

9

4.93E+04

1.93E+05

9.71E+03

6.51E+03

2.65E+04

396610

14184

9706

9

9

9

14032

36662

85842

42317

143850

43633

113680

67892

64728

15925

145110

765490

784010

767480

9093.3

76652

199640

67693

947630

35070

51757

443540

44260

33307

90192

28531

111080

45031

139950

59092

106140

29361

249320

516910

552790

612950

13328

74555

207890

140800

1050900

38359

29507

425900

47338

41849

107560

249290

193850

9

146410 1.16E+05

2963.8 3.55E+05

106670 1.62E+05

29524

72174

80586

1.44E+04

6.36E+04

2.33E+05

55861

53088

303690

46942

56780

530240

534390

512010

555050

67772

51684

340080

37933

21098

279580

233050

214680

246200

21610

128590

516980

59407

14860

290880

838120

876780

946810

27128

66886

372010

67392

14331

208450

863840

858330

883770

21759

299030

49920

106480 1.08E+05

18905 2.60E+04

25733

385040

261810

281100

259170

52609

207080 3.01E+05

176360 5.16E+05

165710 4.85E+05

174560 4.91E+05

21810

55753

2.31E+04

8.45E+05

943390

260770

16451

1464700 1408300 1465300 1251900

319570

22145

836720

7335

284440

19053

414270

13352

10946

242810

24172

329550

13651

111920 2.20E+05

45246 3.26E+04

456670

10947

971990

13492

691220

110440

96938

391810 7.07E+05

591580 8.25E+04

526860 7.25E+04

1093000 8.02E+05

9205.2

860590

161060

31182

11004

9192.5

401380

254090

18983

1068400 1053000

798060

187680

111340

290300

389960

172470

207430

35291 1.46E+05

1182500 1443100 8853900 1.12E+06

 

174040

781550

44838

3272.8

17543

21513

836840

848370

5305.5

871450

22129

29324

23306

668090

21622

475230

22965

26988

50909

27553

230240

146130

91845

9

175090

815910

36743

2763.8

18427

5485

235810

401390

30476

5458.8

24731

20201

188680

877620

47397

8422

222150

695870

52692

3765.5

19021

24397

186940

583140

22419

24978

8430.7

30639

53692

34614

13558

55577

9

158930

469350

27100

17549

11211

29438

142470

122610

8376.8

147010

9

160420 1.74E+05

270940 5.91E+05

20819

71544

18187

3.46E+04

9.45E+03

1.77E+04

17480

63706

416340 9.35E+04

1148900 3.10E+06

946090 3.24E+06

120960 3.60E+04

1100100 2.65E+06

4761400 8703100 6608100 3694700

3754300 8105600 6143300 3939400

7072.8

5892.5

19441

4122

4786700 8291100 6381700 3838600

148520

48986

14074

266000

44767

23367

184150

54236

70223

115060

81785

21410

36328

96899

1.22E+05

6.09E+04

118040

21639

38189

37696

466300

9

200780

21932

120630

51432

501480

9

406680 8.99E+04

1074100 3.08E+06

4207300 7315100 5456900 3452100

17445

473300

109700

114440

74198

18738

86039

267980

167770

9

29436

375030

225020

196780

85859

42951

264680

155810

157300

128040

21298

31650

285390

101040

112610

90442

25931

261130

763230

115300

14041

9

32292 3.27E+04

487680 3.65E+05

31798

17169

9.18E+04

7.52E+04

9

9

3045.2

9

2947.8

9

5485.8 5.80E+04

46200

9

1.17E+04

130680

155410

351930

88605

87519

89438

529040

149400

9

43091

515700

44460

9

65618

464540

63462

52325

41158

41595

435490

129960

244080

241820

219140

359840

7.28E+04

7.39E+04

5.13E+04

6.37E+04

11248

13279

12905

565200

28235

603170

635160

15495

271510

56222

3864400

11909

9645.8

233980

217290

524450

514910

524900

250970

28118

341230

813110

262620

20932

9

8029.5

15577

9

8626

9541.2

506870

16007

470670

436760

20998

349560

68094

8122.8

9

11520

29387

9

1180300 1162100 7443500

35065 35044 174360

33802

9

319520 1.18E+06

9

2.68E+04

1074100 1215500 6850400

1014600 1081200 5979900

22615

28485

9

24615

31378

9

366340 1.14E+06

326740 1.18E+06

28358

766820

61030

44480

137360

67070

190760

588840

87572

9

3.66E+04

307600

5903.7

28712

19842

35767

318610

25274

4.23E+04

4050300 2809300 3905700 4105000 6011600 4736500 5206400 4390000

17205

22468

26720

28179

381380

409460

313300

328420

253370

395740

30632

420970

629100

484850

9

32109

19065

416010

461240

555010

537770

534020

200100

43049

560770

439190

271300

9

34082

22366

250450

263660

763810

706280

707890

347850

31913

396080

723380

374120

9

13089

9950.6

247560

239650

23659

31706

26092

358030

9

30549

13410

332340

355910

28799

44673

42150

230010

9

16485

15590

20360

15189

261490

248530

258450

277770

261270

480640

44912

261010

215860

302970

272720

255070 4.07E+05

260750 3.10E+05

224830 3.18E+05

158600 4.03E+05

9

2.27E+04

428390

393730

488270

505330

10948

333580

9

416010

9

265960

4030.3

228630

394730

27100

355260

9

424410 4.17E+05

9

3.15E+04

 

283670

118770

115660

455900

40236

168980

61378

40666

163160

11214

46446

15457

37655

76110

102900

37275

376610

256670

67913

34894

132210

13536

27747

19829

15186

60269

69911

28289

466100

622930

182400

102480

273400

20394

30156

19475

30451

54692

56533

9

335500

134960

37077

170440

13319

49481

14112

35309

70486

67487

9

530270

117370

47356

103580

3894.5

7741.9

4603.8

9

422740

115920

33389

106950

6242.4

11734

5782.1

9

219400 2.32E+05

58331

30287

9.70E+04

3.20E+04

245590 3.36E+05

9028.4 1.97E+04

170340

57524

15606

10292

9

5.18E+04

2.70E+04

1.50E+04

7.76E+04

9.32E+04

2.62E+04

3.23E+05

20062

57440

162530

171520

9

146680

149130

9

96392

69060

9

83658

52839

227810

759750

119330

2256000

523900

125350

625190

527290

72614

237970

9

187690

536970

9

56385

1111300 1308300 1886200 1658500

155520 97862 212980 121300

2154500 2098400 1758400 1841600

144650 8.37E+05

9

1.11E+05

1316900

669350

9

9

9

492140

237250

262780

217600

452710

250540

346920

268270

113100

317480

180490

494540

861260

47631

56892

1415700

9

161440

623650

216430

6986600 10721000 10527000 18706000 10474000 580410

1595500 9603600

23257

22181

703820

96587

27564

103370

49535

27332

643260

51773

681920

58716

25919

25675

17923

14693

47156

40719

38426

39285

64896

78015

773620

9

26124

26542

754210

9

30503

26581

762510

253420

30708

1094600 1238800 1200900

1066000

203420

2.30E+04

623410

59334

585020

16167

376690

69347

477490

75366

37985

576290

66617

922670

80266

496720

52930

9

9

9

239970

59781

460250

63841

356280

50472

534890

71635

200920

208240

69111

533570

75752

170560

186960

8481.2

807530

85318

113710

332020

79958

1162400

69331

163060

182380

24643

1202700

72558

147400

332130

65469

971280

57749

79379

202870

16346

23637

51833

44833

59388

511020

114300

701620

114050

510530

95564

464690

6.37E+04

858880

61989

1338700 1680600

103170 101610

1343700

99932

12031000 19771000 11363000 13634000 9995200

10940000

212510

5.07E+04

188740

24699

231630

45509

197020

47640

260630

51982

106070

53020

8652900

1513300

902950

70716

8368800 7181600 8146200 9614400 10031000 9615900 9827200 9496800

1289900 1190700 1110600 1748800

213080

125630

183450

235280

549210

834760

741060

172840

20014000

111620

196950

842620

50578

944530

101990

1203600 1043300 1091900

142430 105600 124330

139860

463230

305190

414990

23090000 31999000 25776000 30896000 21288000 171960

145140

82555

172630

295080

1067800

97003

197660

374540

133540

182370

123860

239910

82225

9

9

9

9

9

9

9

9

9

135470

9

2031100 1226100 1325000 1074300

9

9

76675

86594

50622

99883

1237900

7030500

159350

881.23

1163100

958070

1228300 1047200

982690

9

1634600 2217500 1150800

9608700 8938300 8279400 7576400

9

9

5470500

111530

3126.7

39012

132390

2769.5

45755

268410

1392.2

82546

81870

2070.5

48483

132580

1908.7

44364

30512

5555.1

9

45000

8720.7

9

130030

10561

9

8630.9

 

610.45

586420

1294300

206950

647340

1586400

69953

3553.2

512080

8988.7

266010

7244.4

661660

3064

9

9

9

2800.3

543990

1135700

201680

853840

1218800

42755

419950

258380

412690

296430

343790

382830

299270

145970

478200

264570

1609900 1302500 1711400 1139100

161910

734210

171370

582100

174170

714530

152460

640600

1170600 1222900 1069300

1800700 1101100 2485400 1185000

97386 172830 46004 92043

643680

9

984380

9

487550

9

2186000

144910

2123000 1960700 2159600 2051800 2113300 2010500 1842900 2046900

149930 132000 139260 147100 133690 127870 122630 142730

46106000 44042000 42593000 46958000 45443000 47221000 42610000 42015000 44948000

1321800

1320500

2162700

2738600

14093

1242300 1211100 1178200 1360000 1027300 1199800

951030

1200600

1375900 1055500 1082800 1222400 1166000 1193000 1106900 1266800

2042600 1926300 2062800 1961700 2038900 2017600 1869500 1994300

2648500 2266500 2794300 2355400

317040

9

431790

9

511370

9

2485500

23600

32448

23519

161790

4157.2

121480

48646

90275

6641.3

84887

91109

157010

50046

19844

94765

3114.2

612360

136990

72335

2722.8

72540

72548

32201

43659

18130

276470

5653.6

362470

76690

96070

8653.2

85423

95342

139980

48462

18933

273390

7708.8

183510

92001

76328

7383.4

80484

90189

109050

18063

1779.8

19112

9

7624.8

18247

9

2374.2

30653

9

11252

103500

4614.2

212550

21716

112400

5755.3

372870

41884

140710

7606.4

105640

9

428390

9398.4

84023

9

151420

3569.1

87479

9

69855

86209

9736.7

63638

5835.2

75819

108880

99541

498410

88312

80523

447710

78599

81340

359080

75840

89099

164740

6693500

414790

52725

289010

7620000 8837400 9092700 10099000 12205000 15522000 10458000 9976600

707900

55041

392220

54886

720430

49216

723430

63356

1183700

49460

47084

68657

71971

139080

68349

994150

61231

39965

75215

96593

211070

39639

789210

55225

37795

95088

93401

206650

57655

741210

51012

68354

169940

159800

168410

190930

86744

105000

113980

120640

268520

86791

300010

172080

175390

307230

120740

226550

137150

130250

176540

78306

97259

120130

138020

210390

31941

111660

123400

311210

96806

 

mix02 cpd_ID kegg_map

mix03

2.67E+06 2.67E+06 C01746 ko00960

4.39E+06 4.51E+06 C03665

4.11E+06 4.27E+06 --

--

--

1.45E+06 1.45E+06 C01026 00260,ko011

3.60E+07 3.89E+07 C00114 00564,ko011

2.87E+05 3.13E+05 C00168 0,ko01100,k

2.92E+05 2.88E+05 C01146 3,ko00630,k

2.98E+06 2.79E+06 C01089 2,ko00650,k

4.77E+05 4.34E+05 C00065 0,ko00920,k

3.26E+04 3.00E+04 C06772 ko00564

2.45E+04 2.54E+04 C01987 00380,ko011

3.27E+05 2.91E+05 C00380 00240,ko011

2.02E+05 2.27E+05

--

--

3.80E+06 3.06E+06 C00148 0,ko01100,k

2.58E+05 2.44E+05 C00122 00360,ko006

3.68E+07 3.32E+07 C00183 00966,ko009

1.95E+06 1.92E+06 C00719 0,ko01100,k

1.70E+07 1.67E+07 C00042 0,ko00630,k

1.71E+07 1.76E+07 C02170 00280,ko006

2.07E+05 2.88E+05 C20827 00280,ko011

5.91E+05 4.23E+05 C00188 0,ko00970,k

1.43E+06 1.52E+06

3.17E+06 4.28E+06 C08276 0,ko00920,k

4.32E+04 3.70E+04 C06463 --

--

--

4.91E+04 2.62E+04 C00097 00730,ko007

8.09E+04 8.70E+04 C05332 00360,ko011

2.09E+05 2.38E+05 C00633 ko01100

1.14E+06 1.20E+06 C00153 00760,ko011

8.32E+04 1.08E+05 C00253 00960,ko011

7.45E+04 8.51E+04 C10164 00380,ko011

8.37E+04 8.96E+04 C07446

--

1.81E+05 1.42E+05 C02835 00340,ko011

9.85E+03 1.02E+04 C01879 00480,ko011

1.62E+04 7.82E+03 C02237 ko01100

4.17E+06 3.99E+06 C00408 00960,ko011

5.82E+06 4.65E+06 C03969

5.92E+06 4.88E+06 --

--

--

9.94E+04 8.35E+04 C00490 00660,ko011

9.44E+04 6.33E+04 C00430 0,ko01100,k

1.29E+07 1.39E+07 C01157 0,ko01100,k

1.52E+04 1.40E+04 C00300 0,ko00330,k

2.41E+06 2.33E+06 C02378 ko01100

3.84E+07 3.85E+07 C00123 0,ko01100,k

2.97E+06 3.18E+06

--

--

--

3.94E+07 3.86E+07 C01933

 

4.02E+07 3.99E+07 C00407 6,ko00970,k

2.38E+06 2.55E+06 C21096

--

--

--

2.95E+05 3.19E+05

3.43E+06 3.42E+06

--

--

3.78E+05 3.87E+05 C06010 00770,ko011

3.76E+05 3.68E+05 -- --

3.31E+04 3.09E+04 C02642 00410,ko007

2.79E+04 2.06E+04 C00152 00970,ko011

3.25E+04 3.15E+04 C06103

--

1.22E+05 1.28E+05 C00077 0,ko01100,k

4.35E+06 4.62E+06 C00049 0,ko00710,k

5.11E+05 5.27E+05 C19911

--

3.77E+07 3.70E+07 C03064 00053,ko011

1.30E+06 1.32E+06 C00149 0,ko00710,k

3.26E+05 3.32E+05 C22040 ko02010

5.69E+05 5.77E+05 C00147 0,ko00908,k

3.01E+05 3.25E+05 C15561

2.63E+05 2.81E+05 C21649

--

--

7.30E+04 7.76E+04 C10700 ko01100

9.17E+04 6.43E+04 C00548

1.54E+05 1.30E+05 --

--

--

6.70E+04 5.72E+04 C00568 00790,ko011

4.96E+05 4.91E+05 C00587 ko00400

7.55E+04 7.36E+04 C00805 01100,ko011

1.68E+05 1.33E+05 C05585 00350,ko011

1.59E+05 2.02E+05 C16700 ko00950

7.47E+04 6.88E+04 C00346 4,ko00600,k

6.01E+06 6.39E+06 C00860 01100,ko011

1.87E+05 1.55E+05 C10172

--

--

--

--

--

--

--

--

1.66E+06 1.54E+06

4.63E+05 4.68E+05

3.78E+04 2.89E+04

--

--

--

2.12E+05 1.64E+05 C21514

1.13E+05 1.35E+05 --

4.59E+04 4.40E+04 C08493

2.41E+05 2.47E+05 --

3.89E+06 3.82E+06 C01035 00330,ko011

1.14E+05 1.37E+05 -- --

3.26E+06 3.15E+06 C00064 0,ko00750,k

3.11E+06 2.85E+06 C00047 00970,ko011

3.14E+05 2.98E+05 C01996 ko00564

1.65E+07 1.31E+07 C00025 0,ko00660,k

1.06E+06 1.11E+06 C03618 00660,ko011

4.40E+05 3.89E+05 C00979 01100,ko011

2.23E+06 2.40E+06

6.46E+04 4.65E+04

--

--

--

--

1.49E+05 1.58E+05 C02614 00660,ko011

 

1.70E+05 1.74E+05 C00684 00053,ko011

1.54E+05 1.51E+05 C06032 0,ko01100,k

1.46E+05 1.42E+05 C03196 00310,ko011

6.02E+04 5.07E+04 C00652

5.49E+06 5.27E+06 C00650

--

--

4.37E+06 4.20E+06 C00073 0,ko01100,k

7.11E+04 5.78E+04 C02216 --

1.10E+05 1.46E+05 C00121 0,ko01100,k

2.17E+05 2.20E+05

1.92E+05 1.94E+05 C00216

5.93E+04 3.70E+04 --

--

--

--

--

1.40E+05 1.41E+05 C00242 00230,ko011

2.94E+04 2.28E+04 C00385 00232,ko011

5.02E+05 4.59E+05

--

--

5.31E+06 5.56E+06 C00755 8,ko01100,k

6.24E+04 5.82E+04 C09847 ko01110

3.74E+05 3.76E+05 C00196 ko01110

1.09E+06 1.07E+06 C00135 0,ko01100,k

3.88E+04 2.95E+04

1.53E+07 1.58E+07

--

--

--

--

--

1.38E+07 1.40E+07 C01633

2.09E+05 2.13E+05 C03087 00310,ko011

1.20E+04 1.87E+04 C16657

1.88E+04 1.95E+04 C19837

--

--

6.14E+04 6.92E+04 C00956 1,ko01100,k

2.77E+04 2.93E+04

2.25E+05 2.62E+05 C03761

2.99E+05 3.56E+05 --

--

--

--

--

6.89E+05 7.20E+05 C01772 00940,ko011

7.59E+04 6.59E+04 C12621 00360,ko011

9.76E+04 1.36E+05 C05653 00380,ko011

1.41E+07 1.48E+07 C00079 6,ko00970,k

1.01E+05 1.04E+05 C06337 ko01100

7.99E+06 8.26E+06 C16614

--

2.29E+06 2.27E+06 C00502 00040,ko011

1.91E+05 2.28E+05

6.05E+04 5.39E+04

--

--

--

--

5.78E+04 5.65E+04 C03722 00410,ko007

2.39E+04 3.45E+04 C00250 00750,ko011

1.95E+04 2.80E+04

1.74E+05 2.08E+05 C06672

5.91E+04 3.24E+04 --

--

--

--

--

1.26E+05 1.32E+05 C00314 00750,ko011

2.31E+05 2.38E+05 -- --

5.58E+05 5.75E+05 C01152 00340,ko011

2.29E+05 2.38E+05 C00111 00564,ko006

1.81E+04 1.45E+04 C16322 00592,ko011

 

1.41E+05 1.13E+05 C05422 3,ko00480,k

2.70E+04 2.36E+04 C00493 01100,ko011

9.12E+04 9.42E+04

--

--

--

3.89E+04 3.82E+04 C08278

7.32E+06 7.35E+06 C00437 0,ko01110,k

5.18E+06 5.37E+06 C00062 2,ko00970,k

1.61E+05 1.98E+05

--

--

5.73E+04 6.01E+04 C01042 00250,ko011

1.33E+06 1.37E+06 C00327 01100,ko011

6.66E+04 9.08E+04

--

--

3.15E+05 2.62E+05 C00072 00480,ko011

1.52E+04 2.10E+04 C02670 00053,ko011

6.39E+05 6.09E+05 C04209

--

3.46E+05 3.20E+05 C02504 01100,ko011

2.51E+05 3.20E+05 C05994 ko00620

3.43E+05 3.17E+05 C04411 0,ko01110,k

2.13E+04 2.46E+04

1.36E+04 1.58E+04

--

--

--

--

--

3.02E+04 2.33E+04 C03975

9.45E+04 1.27E+05 C01197 0,ko01100,k

6.70E+06 6.77E+06 C00031 0,ko00520,k

7.66E+06 7.72E+06 C00137 2,ko01100,k

6.39E+06 6.02E+06 C00124 00052,ko011

7.63E+06 6.62E+06 C00159 2,ko00520,k

7.26E+06 6.80E+06 C05003

--

7.73E+04 8.89E+04 C00590 00998,ko011

2.92E+04 3.26E+04

5.22E+04 5.10E+04

9.63E+03 7.08E+03

--

--

--

--

--

--

2.02E+07 1.99E+07 C00082 0,ko00950,k

1.70E+07 1.77E+07

8.22E+03 8.23E+03

1.06E+05 5.88E+04

--

--

--

--

--

--

8.53E+04 7.69E+04 C00794 00052,ko011

2.17E+04 2.33E+04 C00392 1,ko01100,k

7.19E+04 7.78E+04 C01697 00052,ko011

1.25E+05 1.30E+05 C00847 00750,ko011

3.47E+05 4.32E+05 C00588 00564,ko011

4.53E+05 4.83E+05 C17715

--

--

--

--

--

--

--

5.95E+06 6.56E+06

6.02E+06 6.65E+06

--

--

5.11E+04 3.85E+04 C18078

7.48E+05 7.68E+05 C08261

4.02E+04 3.84E+04 C17878

7.72E+04 8.27E+04 C02155

1.67E+05 1.40E+05 C02727 00310,ko011

5.28E+04 6.34E+04 -- --

 

6.27E+06 6.45E+06 C03884

6.41E+06 6.72E+06 --

2.49E+03 5.59E+03 C02774

--

--

--

5.63E+05 5.23E+05 C00624 0,ko01110,k

4.87E+04 4.13E+04 C02427

2.23E+04 1.89E+04 C16987

--

--

3.25E+07 3.26E+07 C00158 01100,ko011

1.54E+07 1.65E+07 C00311 0,ko01110,k

2.86E+05 2.60E+05

5.55E+05 5.73E+05 C00296 00400,ko011

1.03E+05 1.23E+05 C17853 --

1.10E+05 1.00E+05 C00191 3,ko00520,k

2.37E+05 3.31E+05 C10477 --

3.09E+06 3.39E+06 C01494 0,ko01100,k

1.36E+04 1.74E+04 C03844 --

5.94E+04 7.71E+04 C07481 2,ko01100,k

--

--

2.89E+04 3.13E+04

--

--

--

3.41E+04 3.61E+04 C03404

1.88E+06 1.80E+06 C00257 01100,ko011

1.21E+04 2.55E+04 -- --

3.19E+04 3.03E+04 C00355 0,ko00965,k

1.60E+04 2.85E+04 C10833 --

1.97E+04 2.30E+04 C00279 0,ko00998,k

1.29E+05 1.36E+05 C02679 1,ko01100,k

7.58E+05 7.99E+05 C03626

--

4.08E+06 4.58E+06 C00750 0,ko00480,k

2.18E+07 2.25E+07 C00078 6,ko00970,k

1.07E+05 1.33E+05 C05944 ko00770

2.24E+04 3.37E+04

4.19E+05 4.39E+05

4.35E+05 4.11E+05

--

--

--

--

--

--

6.44E+04 6.70E+04 C03519 00360,ko011

7.98E+03 8.58E+03

1.75E+04 1.47E+04

--

--

--

--

5.66E+04 5.73E+04 C05610 0,ko01100,k

1.54E+06 1.60E+06 C00818 00053,ko011

1.54E+06 1.51E+06 C00879 00053,ko011

7.15E+05 7.63E+05 C02076 00710,ko011

4.13E+04 2.25E+04 C00672 0,ko00240,k

1.16E+05 9.01E+04

--

--

--

2.99E+03 2.61E+03 C08317

1.67E+05 1.35E+05 C00978 00380,ko011

1.72E+06 1.84E+06 C00864 00770,ko011

4.53E+05 5.21E+05 C01132 00052,ko011

3.59E+04 4.74E+04

8.19E+04 9.16E+04

--

--

--

--

--

247580

247320

C02533

 

2.96E+04 3.39E+04 C22007

--

3.53E+04 2.82E+04 C00482 0,ko01100,k

2.67E+05 2.02E+05

1.35E+05 1.49E+05

--

--

--

--

1.61E+07 1.57E+07 C06424 00061,ko011

8.95E+04 9.56E+04

2.61E+04 2.69E+04

8.51E+04 9.38E+04

4.10E+05 4.14E+05

1.77E+07 3.35E+07

--

--

--

--

--

--

--

--

--

--

5.01E+07 5.45E+07 C00517 ko00071

7.96E+04 8.66E+04 C03592 --

2.77E+05 3.05E+05 C00214 00240,ko011

2.81E+05 2.91E+05

--

--

--

--

2.19E+05 2.54E+05 C16537

2.47E+06 2.65E+06 C02961

1.62E+06 1.58E+06 C00475 0,ko01100,k

2.09E+05 2.59E+05 C00299 0,ko01100,k

1.46E+05 1.35E+05 C16908

--

--

--

--

--

1.53E+05 1.48E+05

5.56E+04 6.71E+04

1.07E+06 1.28E+06

3.57E+04 4.40E+04

--

--

--

--

9.81E+03 7.75E+03 C00669 00480,ko011

1.02E+07 1.04E+07 -- --

1.64E+06 1.75E+06 C00559 0,ko01100,k

1.96E+04 3.16E+04 C05512 0,ko01100,k

4.41E+03 6.67E+03

--

--

5.40E+03 2.76E+03 C08362 ko00061

6.98E+05 7.64E+05 C05841 00760,ko011

5.33E+04 5.50E+04 C00249 1,ko00073,k

3.30E+05 2.77E+05 C00670 00564,ko005

1.61E+04 1.92E+04

--

--

1.70E+04 1.30E+04 C06156 00520,ko011

2.14E+06 2.19E+06 C00085 00710,ko011

2.72E+06 2.74E+06 C00103 0,ko00520,k

3.39E+06 3.28E+06 C00092 2,ko00998,k

1.60E+05 1.74E+05

2.19E+04 1.91E+04 C01096 00051,ko011

2.25E+05 2.76E+05 -- --

--

--

7.00E+03 7.32E+03 C06082 01100,ko011

1.63E+06 1.89E+06 C10497 00330,ko011

6.25E+06 6.11E+06

--

--

5.61E+05 5.28E+05 C00330 0,ko01100,k

4.29E+07 4.81E+07 C00212 0,ko01100,k

4.20E+07 4.48E+07

1.48E+05 1.37E+05

--

--

--

--

 

1.12E+04 1.43E+04 C00294 0,ko01100,k

7.02E+04 7.01E+04

1.93E+05 1.68E+05

--

--

--

--

7.46E+04 9.29E+04 C01477 3,ko00944,k

2.23E+04 2.07E+04 C10044 ko00941

9.17E+04 9.06E+04

--

--

4.29E+03 3.88E+03 C09826 ko00941

4.97E+03 3.23E+03 C00509 00943,ko011

9.94E+04 1.03E+05

1.62E+06 1.73E+06

6.47E+04 7.41E+04

2.81E+06 3.07E+06

1.25E+04 1.30E+04

--

--

--

--

--

--

--

--

--

--

1.89E+04 2.04E+04 C00449 0,ko01100,k

5.14E+04 5.96E+04

1.86E+07 1.86E+07

--

--

--

--

--

--

--

--

1.32E+07 1.32E+07 C14214

1.25E+07 1.23E+07 C15205

1.38E+07 1.32E+07

4.41E+04 4.62E+04

--

--

1.40E+04 1.89E+04 C07289 ko00591

1.73E+05 1.58E+05 C08364 --

7.36E+06 7.54E+06 C06427 01040,ko011

1.35E+04 1.82E+04 C06426 1,ko01040,k

1.98E+06 2.13E+06

8.51E+05 9.99E+05

6.28E+04 7.55E+04

8.57E+06 9.19E+06

--

--

--

--

--

--

--

--

3.71E+07 3.88E+07 C01595 1,ko01040,k

8.12E+04 8.69E+04

2.34E+05 2.63E+05

--

--

--

--

--

--

--

1.19E+05 1.45E+05 C19670

4.99E+06 5.18E+06 C21944

5.96E+05 5.01E+05 C08432

7.84E+06 8.08E+06 C00387 0,ko01100,k

1.43E+05 1.46E+05 C13846 --

7.71E+04 8.67E+04 C01762 2,ko01100,k

5.53E+04 5.25E+04 C01530 00061,ko010

1.82E+04 2.25E+04

--

--

1.93E+05 1.91E+05 C19615 00073,ko011

8.18E+03 8.73E+03 C11849

4.07E+04 3.69E+04 --

--

--

1.46E+05 1.51E+05 C03406 0,ko01100,k

9.53E+03 1.01E+04

2.84E+04 2.48E+04

--

--

--

--

7.82E+04 9.23E+04 C16342 ko00592

7.20E+04 7.74E+04 C16346 ko00592

 

6.92E+03 5.50E+03 C14766 ko00591

1.99E+04 2.31E+04 C16316 ko00592

1.70E+05 1.87E+05

3.35E+05 3.82E+05

2.01E+05 2.50E+05

1.73E+04 1.31E+04

--

--

--

--

--

--

--

--

1.41E+05 1.43E+05 C14767 ko00591

3.46E+04 3.46E+04 C14825 00591,ko011

8.16E+04 8.68E+04 C14762 ko00591

4.64E+06 5.29E+06 C00170 0,ko00908,k

1.01E+05 7.12E+04 C20674

3.31E+03 5.71E+03 --

2.51E+05 2.87E+05 C08365

--

--

--

--

--

--

--

--

--

--

--

--

--

--

--

--

--

--

--

--

--

--

--

2.95E+05 3.51E+05

5.28E+04 5.76E+04

--

--

2.32E+05 2.35E+05 C10058

2.16E+05 2.56E+05 C10038

2.12E+05 2.01E+05 C10534

2.28E+05 2.26E+05 C17059

3.84E+06 3.95E+06

3.87E+06 4.12E+06

5.11E+06 4.71E+06

1.19E+07 1.04E+07

9.00E+03 1.01E+04

--

--

--

--

--

6.97E+04 6.71E+04 C03042

8.85E+04 7.98E+04

3.52E+05 5.41E+05

--

--

2.98E+04 2.37E+04 C10177

2.01E+04 2.23E+04 C10105

9.84E+05 1.08E+06

1.37E+05 1.24E+05

2.10E+05 2.37E+05

--

--

--

3.42E+04 4.26E+04 C05324

1.29E+04 9.13E+03 C00219 00591,ko010

5.19E+05 5.14E+05 C00051 00480,ko011

2.12E+05 2.17E+05

--

--

4.26E+05 4.45E+05 C16525 ko01040

2.10E+04 3.23E+04

2.24E+06 2.29E+06

--

--

--

--

1.15E+05 1.23E+05 C16321 00592,ko011

9.69E+04 1.03E+05 -- --

1.84E+05 1.96E+05 C04785 2,ko01100,k

3.61E+03 5.60E+03 C21923 00592,ko011

4.01E+04 3.46E+04 C16526 ko01040

5.32E+04 5.25E+04 C05143 0,ko01100,k

7.73E+04 9.02E+04

7.38E+04 7.72E+04

--

--

--

--

 

3.81E+04 4.74E+04

3.24E+05 3.31E+05

--

--

--

--

4.73E+04 5.95E+04 C14827 00591,ko011

1.24E+05 1.21E+05 C07354 ko00591

1.71E+06 1.79E+06 C04717 00591,ko011

6.34E+04 5.94E+04 C20186 ko00591

3.41E+05 2.76E+05

9.53E+04 7.40E+04

--

--

--

--

1.82E+05 1.99E+05 C14829 ko00591

6.82E+03 5.66E+03

1.57E+04 1.80E+04

1.46E+04 8.80E+03

1.72E+07 1.78E+07

1.68E+07 1.75E+07

1.48E+05 1.38E+05

1.36E+05 1.38E+05

3.46E+05 3.19E+05

1.73E+07 1.80E+07

1.14E+05 1.23E+05

--

--

--

--

--

--

--

--

--

--

--

--

--

--

--

--

--

--

--

--

8.63E+05 9.41E+05 C12144 00600,ko011

6.71E+03 6.95E+03 -- --

4.25E+04 3.33E+04 C02906 00941,ko011

2.32E+04 2.40E+04

--

--

--

2.27E+05 2.03E+05 C11347

4.76E+03 4.45E+03 C18699 ko04075

1.02E+05 8.49E+04 C00105 00240,ko011

1.80E+04 1.83E+04 C20792

--

--

--

--

--

--

--

--

--

--

--

--

--

2.03E+06 2.26E+06

2.08E+06 1.97E+06

2.39E+06 2.51E+06

3.77E+06 4.06E+06

--

--

--

--

1.81E+06 1.83E+06 C06739

2.23E+06 2.39E+06 C04415

4.47E+04 8.24E+04 C08247

1.17E+07 1.21E+07

--

3.42E+04 4.23E+04 C10650

1.28E+05 1.19E+05

2.92E+04 3.42E+04

--

--

--

37584

38603

9.70E+04 1.06E+05 C00575 00230,ko011

7.05E+04 7.58E+04

--

--

--

--

--

--

--

--

1.70E+05 2.03E+05 C10193

1.04E+07 9.82E+06

5.04E+04 5.60E+04

8.97E+06 8.49E+06

2.45E+07 2.56E+07

7.70E+04 7.66E+04

--

--

--

--

--

 

1.80E+04 1.70E+04 C14835 ko00591

8.29E+04 8.41E+04 -- --

9.85E+05 8.93E+05 C00360 00230,ko011

3.29E+06 3.28E+06 C03996 --

3.21E+06 2.76E+06 C06196 ko00230

2.97E+04 2.74E+04

--

--

--

--

--

--

--

--

--

--

4.01E+05 4.23E+05 C01158

2.86E+04 3.00E+04

3.38E+04 3.43E+04

3.93E+06 4.02E+06

4.09E+06 4.04E+06

--

--

--

--

--

--

32361

31534

9.87E+04 6.71E+04

3.37E+05 3.77E+05 C10441

1.33E+06 1.24E+06 C12208 00941,ko009

1.19E+06 1.28E+06

5.99E+04 7.48E+04

--

--

--

--

2.30E+04 2.40E+04 C01231 00500,ko011

1.13E+04 1.01E+04

--

--

--

1.42E+04 1.21E+04 C09264

6.43E+04 4.74E+04 C02887 ko00940

7.72E+03 1.15E+04 -- --

6.99E+03 5.19E+03 C08281 00073,ko010

7.05E+05 6.86E+05

4.65E+04 4.23E+04

2.31E+04 1.56E+04

5.07E+05 5.61E+05

--

--

--

--

--

--

--

--

7.92E+06 7.91E+06 C00243 2,ko01100,k

9.24E+06 1.19E+07 C01971 --

5.09E+06 5.25E+06 C05402 2,ko01100,k

1.71E+06 1.46E+06 C01235 00052,ko011

6.86E+06 5.48E+06 C01083 01100,ko011

1.70E+07 1.48E+07 C00089 0,ko01100,k

7.76E+06 9.50E+06 C01742

--

6.22E+05 6.83E+05 C00761 ko00940

1.33E+04 1.36E+04

--

--

--

--

--

--

--

9.23E+03 9.61E+03 C10101

2.51E+06 2.48E+06

2.69E+04 4.52E+04

2.44E+05 2.52E+05

4.98E+03 6.13E+03

--

--

--

--

5.28E+05 4.89E+05 C00942 00230,ko011

31075 35726 -- --

3.77E+05 3.89E+05 C00020 00908,ko011

3.39E+04 3.37E+04 C00130 0,ko01100,k

7.17E+03 5.06E+03

5.20E+03 7.27E+03

--

--

--

--

 

5.01E+05 5.39E+05 C17147

--

--

--

8.23E+06 8.37E+06

2.12E+04 3.31E+04

--

--

1.34E+07 1.32E+07 C00852 00941,ko009

5.26E+04 7.36E+04 C10882 ko01110

6.71E+03 7.22E+03

1.44E+04 1.55E+04

7.05E+04 5.76E+04

--

--

--

--

--

--

--

--

--

--

--

--

--

--

--

--

--

--

--

2.46E+05 2.85E+05 C17759

8.83E+03 6.43E+03 C17855

6.70E+04 8.62E+04

2.62E+06 2.46E+06

1.40E+07 1.27E+07

2.02E+06 2.23E+06

--

--

--

--

9.69E+05 1.13E+06 C02572

1.34E+07 1.31E+07

4.98E+04 6.45E+04

2.64E+04 2.13E+04

--

--

--

75729

69590

C09800

3.64E+04 3.12E+04 C10725

1.92E+04 2.37E+04 --

2.56E+04 2.98E+04 C00255 01100,ko011

3.14E+04 2.89E+04

1.18E+04 1.35E+04

6.46E+05 6.83E+05

--

--

--

--

--

--

--

--

--

--

--

--

--

--

--

--

--

--

--

--

--

--

--

4.72E+06 4.84E+06 C02919

8.35E+03 4.51E+03 --

5.42E+03 5.15E+03 C10561

8.63E+04 8.25E+04

1.01E+05 1.40E+05

3.08E+05 3.21E+05

--

--

--

6.58E+05 3.78E+05 C03690

1.23E+06 1.12E+06

8.73E+04 9.58E+04

7.15E+05 7.87E+05

--

--

--

5.13E+03 6.37E+03 C10112

2.67E+05 2.62E+05

2.05E+05 2.61E+05

7.21E+03 8.41E+03

1.79E+04 2.91E+04

9.91E+05 9.34E+05

1.10E+05 7.39E+04

--

--

--

--

--

--

9.54E+04 9.48E+04 C00689 0,ko01100,k

1.43E+06 1.31E+06

4.34E+04 3.91E+04

3.78E+04 3.52E+04

1.04E+06 1.12E+06

--

--

--

--

--

--

--

--

 

7.72E+04 5.97E+04 C00008 00230,ko009

1.81E+05 1.73E+05 -- --

5.13E+04 4.14E+04 C09126 ko00943

3.76E+04 2.80E+04 C16911 ko00944

6.80E+04 8.53E+04 C04608 ko00944

5.46E+05 5.33E+05 C17564

--

--

--

--

--

--

--

--

--

--

--

--

--

--

--

--

5.91E+05 5.82E+05

1.79E+04 1.41E+04

8.20E+05 8.95E+05

8.53E+05 9.16E+05

5.33E+05 6.52E+05

7.74E+04 6.55E+04

4.19E+04 4.36E+04

8.76E+03 8.08E+03

3.11E+05 3.85E+05

3.20E+05 4.01E+05

2.21E+05 3.04E+05

4.20E+04 5.33E+04

4.49E+04 4.34E+04

2.68E+05 2.10E+05

3.24E+04 2.13E+04

--

--

--

--

--

--

--

--

--

--

--

--

--

--

--

5.89E+04 1.06E+05 C01750 ko00944

4.65E+05 3.78E+05 C03951 ko00944

2.29E+05 2.50E+05

1.70E+05 2.50E+05

--

--

--

--

2.08E+05 2.66E+05 C12249 00944,ko011

1.76E+05 1.61E+05

2.64E+04 3.09E+04

1.86E+04 1.79E+04

1.71E+05 1.57E+05

5.64E+03 3.42E+03

8.40E+04 9.81E+04

8.28E+04 8.07E+04

1.23E+05 1.35E+05

7.62E+06 8.11E+06

8.14E+05 1.03E+06

--

--

--

--

--

--

--

--

--

--

--

--

--

--

--

--

--

--

--

--

--

--

--

--

--

--

--

--

--

--

98026

219360

214640

225560

239760

115890

125890

162400

96456

118960

3.39E+04 3.18E+04 C00061 00740,ko011

213340

1144400

958320

122840

703230

641440

595440

--

--

--

--

--

--

C08988

--

--

--

1092800

4.43E+05 3.91E+05

 

2.31E+05 2.83E+05 C09731

--

--

--

--

--

--

--

--

--

2.93E+05 3.51E+05

4.82E+06 4.92E+06

2.23E+04 1.32E+04

--

--

--

2.38E+04 2.93E+04 C10533

4.13E+04 4.15E+04

3.61E+04 3.75E+04

--

--

3.34E+04 2.57E+04 C17762

1.20E+06 1.22E+06 --

5.93E+05 5.63E+05 C05623 4,ko01100,k

1.33E+06 1.47E+06 --

3.11E+05 2.84E+05 C12639

3.62E+05 4.20E+05 --

8.00E+05 8.28E+05 C10073

--

--

--

--

--

--

--

--

--

--

--

--

--

--

--

--

--

--

--

--

--

--

--

3.70E+05 3.89E+05

3.17E+04 3.54E+04

6.19E+04 6.84E+04

2.49E+04 3.19E+04

1.77E+05 1.79E+05

--

--

--

--

--

1069000

8351.6

764390

8042

C08619

--

501220

698610

--

2.38E+05 2.13E+05

--

9862.8

148790

449720

474220

70550

10088

165330

528840

561300

100720

99862

--

--

C16912

--

--

112100

147060

426320

151200

413870

--

174260

501250

185690

535250

--

--

--

C16939

1.73E+06 1.88E+06 C00234 00970,ko011

7.08E+04 8.09E+04

--

--

--

--

--

--

--

--

--

--

--

--

--

--

2.73E+05 3.33E+05 C10437

3.98E+04 3.70E+04

1.42E+05 1.35E+05

1.76E+06 1.73E+06

3.99E+04 4.79E+04

1.45E+07 1.63E+07

3.05E+06 3.59E+06

2.43E+04 2.78E+04

2.91E+04 2.12E+04

7.60E+04 8.13E+04

8.18E+04 9.30E+04

2.18E+04 2.29E+04

--

--

--

--

--

--

--

--

--

--

--

 

5.76E+04 7.01E+04

6.70E+04 7.24E+04

7.41E+04 6.11E+04

8.07E+03 1.95E+04

2.63E+05 2.60E+05

3.17E+05 3.25E+05

7.94E+04 1.00E+05

9.08E+04 1.13E+05

1.15E+05 1.17E+05

2.87E+04 3.52E+04

1.23E+06 1.24E+06

2.14E+05 2.39E+05

--

--

--

--

--

--

--

--

--

--

--

--

--

--

--

--

--

--

--

--

--

--

--

--

--

--

--

--

--

--

--

--

--

--

--

--

--

--

--

--

--

--

--

--

--

--

--

--

--

--

--

--

--

--

--

--

--

--

--

12287

12568

2.45E+06 2.74E+06

24891

36259

3109.1

28360

34732

37808

3740.3

34352

1.78E+04 1.28E+04

6.84E+04 7.18E+04

2.09E+05 2.59E+05

1.59E+05 1.60E+05

3.26E+04 2.39E+04

3.51E+04 2.35E+04

2.43E+04 2.57E+04

4.85E+04 4.40E+04

2.77E+07 2.82E+07

1.72E+06 1.86E+06

2.11E+05 1.92E+05

1.38E+06 1.70E+06 C08243

7.10E+04 3.89E+04 C01835 ko02010

3.26E+04 3.56E+04 C05404 00052,ko011

2.15E+06 1.82E+06 C00492 2,ko01100,k

6.67E+05 5.84E+05 C00713

--

--

--

--

--

--

--

--

--

--

--

--

--

--

1.67E+05 1.46E+05

6.04E+04 6.78E+04

4.68E+04 7.29E+04

8.28E+04 8.51E+04

4.49E+04 4.47E+04

6.06E+04 8.31E+04

4.36E+04 5.28E+04

7.77E+04 7.82E+04

5.81E+04 4.23E+04

1.06E+05 1.58E+05

1.23E+04 1.44E+04

1.35E+04 1.07E+04

6.24E+05 6.20E+05

--

--

--

--

--

--

--

--

--

--

--

--

--

 

6.56E+05 6.07E+05

8.38E+04 9.96E+04

9.63E+04 9.85E+04

1.77E+07 1.99E+07

1.49E+07 1.54E+07

1.27E+04 1.11E+04

--

--

--

--

--

--

--

--

--

--

--

--

--

--

--

--

--

--

--

--

--

--

--

--

--

--

--

--

--

--

--

2572700 2943400 C20736

7.03E+05 6.22E+05 --

1.76E+05 1.69E+05 C17529

6.98E+05 7.17E+05

4.35E+06 4.25E+06

1.84E+06 1.79E+06

8.67E+05 1.01E+06

6.46E+05 6.41E+05

1.87E+05 1.85E+05

1.67E+05 2.13E+05

8.11E+05 8.45E+05

7.54E+05 6.52E+05

5.96E+04 4.90E+04

3.97E+06 3.89E+06

5.58E+04 3.98E+04

1.62E+05 2.03E+05

1.01E+04 1.57E+04

1.06E+04 1.14E+04

3.87E+04 3.42E+04

--

--

--

--

--

--

--

--

--

--

--

--

--

--

--

--

1.26E+05 1.05E+05 C00190 0,ko00908,k

2.81E+05 4.01E+05

1.91E+05 1.83E+05

1.66E+04 1.48E+04

8.25E+04 8.98E+04

2.74E+05 3.04E+05

6.54E+04 5.54E+04

2.43E+04 2.30E+04

3.07E+05 3.35E+05

5.01E+05 4.98E+05

5.07E+05 5.40E+05

5.22E+05 5.78E+05

3.58E+04 3.04E+04

9.29E+05 8.66E+05

1.91E+05 2.29E+05

3.84E+04 4.27E+04

6.80E+05 6.71E+05

9.44E+04 6.92E+04

7.23E+04 1.02E+05

--

--

--

--

--

--

--

--

--

--

--

--

--

--

--

--

--

--

--

--

--

--

--

--

--

--

--

--

--

--

--

--

--

--

--

--

8.00E+05 8.59E+05 C00029 00500,ko005

1.60E+05 1.66E+05 -- --

1.20E+06 1.33E+06 C20490 ko00942

 

1.78E+05 2.12E+05 C08694

5.49E+05 6.03E+05 --

--

--

4.34E+04 2.32E+04 C04024 ko00944

1.12E+04 1.50E+04 -- --

1.81E+04 1.26E+04 C00043 00520,ko011

9.78E+04 1.08E+05 C17563

--

--

--

--

--

--

2.74E+06 3.79E+06

2.98E+06 3.08E+06

3.00E+04 3.59E+04

3.28E+06 3.65E+06

1.03E+05 1.03E+05

--

--

--

--

--

5.94E+04 7.59E+04 C09806 ko00941

7.63E+04 1.12E+05 C16370 ko00942

3.11E+06 2.95E+06

--

--

2.82E+04 1.86E+04 C16306 ko00942

3.75E+05 4.24E+05 C00127 00480,ko011

9.64E+04 8.69E+04

8.45E+04 8.64E+04

6.52E+04 5.36E+04

2.35E+04 2.06E+04

--

--

--

--

--

--

--

--

--

--

--

--

--

--

--

--

--

--

--

--

--

--

--

--

--

--

134880

403700

131750

452140

1.17E+05 9.75E+04

7.07E+04 7.05E+04

6.17E+04 5.91E+04

5.19E+04 6.59E+04

1.19E+06 1.47E+06

3.45E+04 3.28E+04

1.18E+06 1.33E+06

1.16E+06 1.33E+06 C12667 ko00944

3.69E+04 4.20E+04

295810 299630

--

--

--

--

--

--

--

--

--

--

--

--

--

--

--

--

--

--

--

--

--

--

--

--

--

--

--

--

--

--

3.41E+04 6.63E+04

4185300 4609800

22292

17378

16842

9699

297950

340040

319960

270800

3.14E+05 3.12E+05

3.13E+05 3.23E+05

2.89E+05 3.33E+05

3.60E+05 3.59E+05

3.52E+04 2.02E+04

416230

360920

393970

528730

3.63E+05 4.26E+05 C00003 00730,ko007

4.12E+04 4.35E+04 -- --

 

4.03E+05 3.22E+05

1.08E+05 1.30E+05 C01613 00052,ko011

4.25E+04 5.84E+04 C02052

--

--

--

--

--

--

--

--

--

--

--

--

--

--

--

--

--

--

--

--

--

--

--

--

--

--

--

3.11E+05 3.85E+05

1.93E+04 2.55E+04

5.86E+04 4.42E+04

1.99E+04 1.92E+04

1.74E+04 2.24E+04

1.10E+05 1.22E+05

9.67E+04 7.91E+04

2.24E+04 2.20E+04

2.89E+05 3.22E+05

9.58E+05 9.81E+05

9.45E+04 1.18E+05

1245000 1562300

--

--

--

--

--

--

--

--

--

--

--

--

380950

359240

314790

227720

--

--

9315000 10489000

--

39917

37209

33663

39532

--

--

765260

308630

1077700

245770

--

--

2.78E+04 2.84E+04

--

537710

68281

50172

502420

366270

80788

--

--

51320

--

435000

C20779

7.86E+04 7.07E+04 C00016 0,ko01100,k

938980

75444

1185500

86105

--

--

--

--

--

--

--

--

--

--

--

--

--

--

--

--

--

--

--

--

--

--

--

--

--

8565200 10604000

218430 188280

2.63E+04 3.26E+04

8960400 9371500

977290

980230

167260

1167500 C08946

845980

201390

--

C08945

21062000 21648000

--

--

--

--

--

--

--

--

--

--

95989

158680

830750

63458

105740

190180

934270

35733

1175500 1259800

5387700 5147300

117840

2550.5

32761

166850

3470.7

28903

 

2269.6

392720

2659.9

423610

--

--

--

--

--

--

--

--

--

--

--

--

--

--

--

--

--

--

--

--

--

--

--

--

--

--

--

--

--

--

--

--

--

--

--

1149000 1200300 C08961

184170

736220

226050

962660

--

C08944

1221500 1255500 C20725

51744

2019700 2056000

147590 132130

47655

--

--

--

46496000 45476000 C17543

977790 1223200

--

--

--

--

--

--

--

--

--

--

--

--

--

--

--

--

--

--

--

--

--

--

--

1245800 1247800

1903200 1832300

1831100 2167600

23135

12225

149110

4412.6

223620

43406

96252

3404.4

80466

89287

244610

28346

11233

111540

4718.2

262180

45739

75314

4062

80036

101370

293190

9431900 10752000

641590

61083

687570

55022

89381

95954

107380

112230

223340

47547

111480

126550

347170

44397
