# Supplementary material for: Metabolome and transcriptome analyses identify the characteristics and expression of related saponins of the three genealogical plants of bead ginseng
Source: PeerJ. 2023 Sep 1;11:e16034. doi: 10.7717/peerj.16034 (PMC10476608; doi:10.7717/peerj.16034)
Supplement: Supplementary file 3 [file peerj-11-16034-s008.odt]

Index

Formula

Compounds

Piperidine

N,N-Dimethylglycine

2-Aminophenol

L-Proline

Class I

Class II

CAS

Level YA_vs_XL

pmb0782 C5H11N

pme3033 C4H9NO2

MWS2076 C6H7NO

pme0006 C5H9NO2

mws0256C5H11NO2

Alkaloids Alkaloids 110-89-4

cids and decids and de 1118-68-9

Alkaloids Alkaloids 95-55-6

cids and decids and de 147-85-3

cids and decids and de 72-18-4

3

1

3

1

1

1

3

3

3

3

3

1

1

1

1

1

1

3

3

3

1

3

3

3

3

3

1

3

3

3

1

1

3

1

1

3

1

1

3

3

3

3

3

3

FALSE

down

up

down

FALSE

FALSE

FALSE

FALSE

FALSE

FALSE

FALSE

FALSE

FALSE

FALSE

FALSE

FALSE

FALSE

up

L-Valine

pmp001287 C8H9N -Benzylmethylene isomethylami Alkaloids Alkaloids

-

pme0195 C3H7NO2S

MWS0813C5H7NO3

pma3649 C5H9NO3

L-Cysteine

5-Oxoproline

5-Aminolevulinate

cids and decids and de 52-90-4

cids and decids and de 149-87-1

Alkaloids Alkaloids 106-60-5

mws0216 C5H9NO3 Trans-4-Hydroxy-L-proline cids and decids and de 51-35-4

ML1019792C4H9N3O2

pme0274 C6H13NO2

mws0227C6H13NO2

pmp001198C6H13NO2

mws1587C6H13NO2

mws0258C6H13NO2

pme2679 C6H13NO2

mws0001C4H8N2O3

pme2527C5H12N2O2

Zmdp00097C24H9NO2S

HJKP000649C8H9NO

Lmqp002784C7H7NO2

mws1336 C7H7NO2

Lmgn001670C7H6O3

pma2987 C6H11N3O

MWSmce15C7H13NO2

Hmsp00036C47H13NO2

Creatine

6-Aminocaproic acid

L-Leucine

6-Deoxyfagomine

L-Norleucine

L-Isoleucine

L-Allo-isoleucine

L-Asparagine

Organic aciOdrganic acid 57-00-1

Organic aciOdrganic acid 60-32-2

cids and decids and de 61-90-5

Alkaloidseridine alka1l 97449-09-5

cids and decids and de 327-57-1

cids and decids and de 73-32-5

cids and decids and de 1509-34-8

cids and decids and de 70-47-3

cids and decids and de 70-26-8

cids and decids and de 1187-84-4

Alkaloids Alkaloids 6343-54-0

Alkaloids Alkaloids 65-45-2

henolic acidhenolic acid 150-13-0

henolic acidhenolic acid 69-72-7

Alkaloids Alkaloids 501-28-0

Alkaloids rrole alkalo 471-87-4

cids and decids and de 2521-84-8

L-Ornithine

down

FALSE

FALSE

down

down

up

FALSE

up

FALSE

up

S-Methyl-L-cysteine

N-benzylformamide

Salicylamide

4-Aminobenzoic acid

Salicylic acid

Histidinol

Stachydrine

L-Cyclopentylglycine

Rfmb320 C7H13NO2Methylpiperidine-2-carboxylic acids and decids and de 7730-87-2

Rfmb318C7H14NO2+ D-Proline betaine cids and decids and de 1195-94-4

Lmqp00042C76H11NO3Methyl-Trans-4-Hydroxy-L-Prolcids and decids and de 4252-82-8

up

up

pme0193C5H10N2O3

pme0026C6H14N2O2

pmb2826 C5H8O5

L-Glutamine

L-Lysine

L-Citramalic acid

cids and decids and de 56-85-9

cids and decids and de 56-87-1

Organic aciOdrganic acid6236-09-5

AlkaloidsPhenolamine13093-65-7

cids and decids and de 63-68-3

ides and derides and der5142-22-3

Others rides and A 50-69-1

ides and derides and der 73-40-5

henolic acidhenolic acid 621-59-0

henolic acidhenolic acid 121-33-5

down

down

FALSE

FALSE

FALSE

FALSE

FALSE

FALSE

up

Lmgp000796C8H7NO2 4-Hydroxymandelonitrile

pme1210C5H11NO2S

mws0847 C6H7N5

Zmgn000173C5H10O5

pme1109 C5H5N5O

MWSmce04 C8H8O3

mws0458 C8H8O3

MWS1962 C10H16O

L-Methionine

1-Methyladenine

D-Ribose

Guanine

Isovanillin

Vanillin

up

up

cis-Citral

Others

Others 5392-40-5

mws0639 C7H6O4 2,3-Dihydroxybenzoic Acid Organic aciOdrganic acid 303-38-8

Lmbn00146C77H13NO3 5-Acetamidopentanoic Acid Organic aciOdrganic acid1072-10-2

up

FALSE

FALSE

mws1417 C9H7NO2

Indole-3-carboxylic acid

Alkaloids Plumerane 771-50-6

 

pme2914 C6H10O5droxy-3-methylpentane-1,5-dioiccids and decids and de 503-49-1

3

3

1

3

3

3

1

3

3

1

3

3

1

1

1

2

3

3

3

3

3

1

1

1

1

1

3

3

3

1

1

3

3

3

3

1

1

3

3

2

2

1

2

1

3

3

FALSE

up

MWSmce24 C9H8O3

pme0021 C9H11NO2

pmc0274 C6H6N4S

MWS3270C7H5NO4

mws0028 C8H8O4

Hmbp00127 C8H8O4

pme1383 C8H11NO3

MWS4471C6H10N2O4N-Alpha-Acetyl-L-Asparagine cids and decids and de 4033-40-3

Zmyn00015C57H14N2O3 N-α-Acetyl-L-ornithine

pmf0173 C10H22O2 1,2-Decanediol

3-Hydroxycinnamic Acid henolic acidhenolic acid14755-02-3

L-Phenylalanine cids and decids and de 63-91-2

6-Methylmercaptopurine ides and derides and der 50-66-8

FALSE

FALSE

FALSE

up

FALSE

down

down

down

up

Quinolinic Acid

Vanillic acid

Gallacetophenone

Pyridoxine

Alkaloids Alkaloids 89-00-9

henolic acidhenolic acid 121-34-6

henolic acidhenolic acid 528-21-2

Others

Vitamin 65-23-6

cids and decids and de 6205-08-9

Others rides and A 1119-86-4

pme2559 C6H9NO5 N-Acetyl-L-Aspartic Acid cids and decids and de 997-55-7

pme0008C6H13N3O3 L-Citrulline cids and decids and de 372-75-8

MWSmce69 C6H8O6Erythorbic Acid; Isoascorbic Aci Others

Hmfn000531C6H8O6 L-Ascorbic acid (Vitamin C) Others

FALSE

FALSE

down

down

down

up

FALSE

FALSE

FALSE

up

Vitamin 89-65-6

Vitamin 50-81-7

mws4175 C6H8O6

Cmsn00013C15H8N2O5

pmb3101 C7H12O5

Zmgn001448C7H12O5

Lmbn001754C7H12O5

mws2212 C9H8O4

mws4170 C6H12O6

Hmln000297C6H12O6

pmf0139 C6H12O6

pmf0138 C6H12O6

mws1164 C6H12O6

mws0093 C10H12O3

D-Glucurono-6,3-lactone

Dencichin

2-Isopropylmalic Acid Organic aciOdrganic acid49601-06-1

2-Propylmalic Acid Organic aciOdrganic acid

3-Isopropylmalic Acid Organic aciOdrganic acid 921-28-8

Others rides and A32449-92-6

cids and decids and de 5302-45-4

-

Caffeic acid

D-Glucose

Inositol

henolic acidhenolic acid 331-39-5

Others rides and A 50-99-7

Others rides and A 87-89-8

Others rides and A 59-23-4

Others rides and A 3458-28-4

Others rides and A 57-48-7

henolic acidhenolic acid 458-35-5

up

up

up

up

up

up

D-Galactose

D-Mannose

D-Fructose

Coniferyl alcohol

Lmbn004847C10H12O34-Methoxyphenylpropionic acidhenolic acidhenolic acid

MWS2070C10H12O3 Propyl 4-hydroxybenzoate henolic acidhenolic acid 94-13-3

mws0250C9H11NO3 L-Tyrosine cids and decids and de 60-18-4

MWStz070C9H11NO3Hydroxy-4-methoxyphenyl)acetaAlkaloidsPhenolamine58469-06-0

-

up

FALSE

FALSE

FALSE

up

FALSE

FALSE

up

mws0214 C6H14O6

mws1155 C6H14O6

pme2237 C6H14O6

pme2596 C8H9NO4

Hmgp00232C11H9O2N 3-amino-2-naphthoic acid

Smcp00113C7 11H9NO2

mws0736C8H16N2O3

mws5041C8H16N2O3 L-Glycyl-L-isoleucine

mws1611 C12H14O2 Z-Ligustilide

Cmpp003619C10H8O46,7-Dihydroxy-4-methylcoumarinns and CoumCoumarins 529-84-0

D-Sorbitol

D-Mannitol

Dulcitol

Others rides and A 50-70-4

Others rides and A 69-65-8

Others rides and A 608-66-2

4-Pyridoxic acid

Others

Alkaloids Alkaloids

Others Others

Vitamin 82-82-6

-

-

up

up

naphthisoxazol A

N-Glycyl-L-leucine

cids and decids and de 869-19-2

cids and decids and de19461-38-2

down

down

FALSE

FALSE

FALSE

FALSE

FALSE

FALSE

FALSE

Others

Others 4431-01-0

MWSmce41C12H16O2

Lmdn003756C10H10O4

mws0014 C10H10O4

mws2218C8H10N4O2

pmp001235C12H18O2

Senkyunolide A

Methyl caffeate

Ferulic acid

Caffeine

Others

Others 63038-10-8

henolic acidhenolic acid3843-74-1

henolic acidhenolic acid 537-98-4

Alkaloids Alkaloids 1958-08-02

Cinidilide

Others

Others

-

 

NK1025188C88H18N4O2 NG,NG-Dimethyl-L-arginine cids and decids and de30315-93-6

1

1

3

3

3

1

3

3

3

3

3

1

3

3

3

3

1

1

1

3

3

3

1

3

3

3

3

1

3

1

1

3

3

3

3

3

1

3

1

2

3

3

3

3

3

3

FALSE

FALSE

FALSE

FALSE

down

down

down

FALSE

FALSE

FALSE

FALSE

down

FALSE

up

mws0282 11H12N2O

pme1261 C9H19NO4

L-Tryptophan

D-Panthenol

cids and decids and de 73-22-3

Others rides and A 81-13-0

pmb2620 C11H12O4 3,4-Dimethoxycinnamic acid henolic acidhenolic acid14737-89-4

pmb2507 C5H11O7P 2-Deoxyribose-1-phosphate ides and derides and der17210-42-3

pme0170C8H16N4O3

MWS429611H14N2O

N-Acetyl-L-Arginine

Glycylphenylalanine

cids and decids and de 155-84-0

cids and decids and de 3321-03-7

mws5042 11H14N2O L-Glycyl-L-phenylalanine cids and decids and de 721-66-4

Zmln002252C10H8O6n (5,7,8-Trihydroxy-6-methoxycnos and CoumCoumarins

-

mws4085 C11H12O5

pmp000253C12H16O4

Lmhp0014611H20N2O

pmb0962 10H22N2O L-Lysine-Butanoic Acid

MWSHY020C16H12O2 6-Methylflavone

Sinapic acid

Senkyunolide H

L-Prolyl-L-Leucine

henolic acidhenolic acid 530-59-6

Others Others 94596-27-7

cids and decids and de52899-07-7

cids and decids and de80407-71-2

Flavonoids Flavones 29976-75-8

MWS291010H15N3O 5-Methyl-2'-Deoxycytidine ides and derides and der 838-07-3

down

down

down

down

down

down

up

FALSE

FALSE

down

FALSE

down

FALSE

FALSE

down

down

down

FALSE

up

pme0264 10H14N2O

ML1018052C9H13N3O5

pme3732C9H13N3O5

mws0248C9H12N2O6

Thymidine

Cytarabine

Cytidine

ides and derides and der 50-89-5

ides and derides and der 147-94-4

ides and derides and der 65-46-3

ides and derides and der 58-96-8

Uridine

MWSmce29C9H12N2O16-beta-D-Arabinofuranosyluraciildes and derides and der3083-77-0

MWS5231C13H24O4

Lmhp0020312H24N2O

pmb2591 13H14N2O N-Acetyl-L-Tryptophan

pme2563 8H14N2O5 γ-Glu-Cys

Tridecanedioic acid

L-Leucyl-L-Leucine

Organic aciOdrganic acid 505-52-2

cids and decids and de 3303-31-9

cids and decids and de 1218-34-4

cids and decids and de 636-58-8

Lmhp00215313H18N2ON-p-Coumaroylhydroxyputrescin AlkaloidsPhenolamine

-

pme3961 10H13N5O

pme2776 10H12N4O

Lmlp00316114H20N2O

pme1184 10H13N5O

pme0230 10H13N5O

Zmjp00096610H13N5O

2'-Deoxyadenosine

2'-Deoxyinosine

N-Feruloylputrescine

2'-Deoxyguanosine

Adenosine

ides and derides and der 958-09-8

ides and derides and der 890-38-0

AlkaloidsPhenolamine501-13-3

ides and derides and der 961-07-9

ides and derides and der 58-61-7

Vidarabine

Others

Others 5536-17-4

mws1060 10H12N4O 9-(Arabinosyl)hypoxanthine ides and derides and der7013-16-3

pmp000087C13H16O6

MWSmce67C12H16O7

2-Feruloyl-sn-glycerol

α-Arbutin

henolic acidhenolic acid

-

Others Others 84380-01-8

up

Lmhp0025014H20N2O N-Feruloylhydroxyputrescine AlkaloidsPhenolamine

-

FALSE

down

down

down

down

FALSE

FALSE

FALSE

FALSE

up

MWS435411H15N5O

Hmhp0018111H15N5O

N6-methyladenosine

2'-O-Methyladenosine

ides and derides and der1867-73-8

ides and derides and der2140-79-6

MWSmce3310H13N5OCrotonoside; 2-Hydroxyadenosinides and derides and der1818-71-9

pme1178 10H13N5O

Zmzp00593C418H37NO

mws0668 10H12N4O

Guanosine

Stearamide

Xanthosine

ides and derides and der 118-00-3

Alkaloids Alkaloids 124-26-5

ides and derides and der 146-80-5

pmn001511C13H18O7droxy-5-Methylphenol-1-O-Gluchenolic acidhenolic acid

mws1436 C18H38O2 1,18-Octadecanediol Lipids ree fatty acid3155-43-9

Zmyn004732C18H30O3roxy-9Z,12Z,15Z-octadecatrieno Others

Zmyn004676C18H30O3 17-Hydroxylinolenic acid Others

-

Others

Others

-

-

up

pmb2787 C18H30O3Oxo-10E,12Z-octadecadienoic ac Lipids ree fatty aci5d4232-59-6

up

 

Zmpn003368C18H30O3droxy-9Z,11E,15Z-octadecatrien Lipids ree fatty aci8d7984-82-5

pmn001687C13H12O8 Cis-Coutaric acid henolic acidhenolic acid67920-37-0

pme1474 11H15N5O'3-Deoxy-5'-(methylthio)adenosinides and derides and der2457-80-9

3

3

1

3

3

1

3

1

2

2

2

3

3

3

3

3

3

3

2

3

1

1

3

3

3

3

3

1

1

2

2

2

3

3

3

3

3

3

3

3

3

1

1

1

1

1

up

up

down

pmb0197 11H15N5O

Lmfp007216C19H22O3

Zmyn004714C18H34O3

pmf0297 C20H42O

N7-Methylguanosine

Acerogenin A

ides and derides and der20244-86-4

henolic acidhenolic acid60503-28-8

Lipids ree fatty acid141-22-0

Lipids ree fatty acid629-96-9

down

up

up

up

Ricinoleic acid

1-Eicosanol

pmb3142 C13H16O8 Salicylic acid-2-O-glucoside henolic acidhenolic acid10366-91-3

FALSE

up

FALSE

FALSE

FALSE

up

FALSE

FALSE

FALSE

down

pme3514 C15H10O7

Hmqn00084C13H18O8

Morin

Tachioside

Flavonoids Flavonols 480-16-0

henolic acidhenolic aci1d09194-60-7

Zmhn000892C13H18O8Glucosyl-3,4-dihydroxybenzyl alhenolic acidhenolic acid

mws1375 12H21N3O Nicotianamine Alkaloids Alkaloids 34441-14-0

pmb2791 C18H30O4peroxy-10E,12,15Z-octadecatrie Lipids ree fatty aci1d11004-08-1

Zmdp00185714H18N2O γ-Glutamyltyrosine cids and decids and de 7432-23-7

Zmyn004449C18H30O4y-12-oxo-10(E),15(Z)-octadecadi Others Others

pmn001606C20H38O2 Eicosenoic acid Lipids ree fatty aci2d6764-41-0

-

-

pme3967 12H17N5O 2-(Dimethylamino)guanosine ides and derides and der2140-67-2

Lmcp00230212H17N5ON6-(2-Hydroxyethyl)adenosineides and derides and der4338-48-1

Lmhn001477C13H12O9affeoyl-L-tartaric acid (Caftaric ahenolic acidhenolic acid67879-58-7

down

FALSE

FALSE

FALSE

FALSE

up

FALSE

FALSE

FALSE

FALSE

FALSE

FALSE

FALSE

FALSE

FALSE

FALSE

FALSE

up

Lmqp002761C19H20O4

Alnusonol

henolic acidhenolic acid52330-12-8

MWS80006C18H32O4operoxy-9Z,11E-Octadecadieno Lipids ree fatty acid5502-91-0

Lmbn005287C18H32OZ4,12Z)-(7S,8S)-Dihydroxyoctade Lipids ree fatty aci1d43288-65-7

pmb2804 C18H32O4droperoxy-9Z,11E-octadecadien Lipids ree fatty aci3d3964-75-9

Lmmn00217C14H18O8Methyl salicylate-2-O-glucosidehenolic acidhenolic acid10019-60-0

Lmyn002403C14H18O8 Mandelic acid-β-glucoside henolic acidhenolic acid

-

Lmbn005487C18H34O4E; (9Z)-12,13-Dihydroxyoctadec Lipids ree fatty aci2d63399-35-5

Zmzp004111C16H12O7

pmn001367C13H16O9rotocatechuic acid-4-O-glucosidhenolic acidhenolic acid

Selagin

Flavonoids Flavones

-

-

pmb2871 C13H16O9 1-O-Gentisoyl-D-glucoside henolic acidhenolic acid23445-11-6

Hmtn001120C14H20O8-Hydroxyethyl)-2-O-glucosylphhenolic acidhenolic acid

-

pmn001553C14H20O8

Zmtn001661C14H20O8

Cimidahurinine

Vanilloloside

henolic acidhenolic aci1d42542-89-0

henolic acidhenolic acid74950-96-2

mwn002172C14H20O8droxyphenylethanol-β-D-glucopyhenolic acidhenolic acid

-

pmb2221C18H39NO3 4-Hydroxysphinganine

Jmzp010766C19H26O4 denudaquinol

Lipids Sphingolipid 554-62-1

ns and CoumLignans 189105-40-

mws0744 C15H12O8Dihydromyricetin (Ampelopsin)FlavonoidsFlavanonols27200-12-0

MWSslk214C20H16O4 Corylin FlavonoidsIsoflavones53947-92-5

pme2074C18H29NO4 (-)-Jasmonoyl-L-Isoleucine Organic aciOdrganic acid120330-93-0

pme3188 9H13N2O9 Uridine 5'-monophosphate ides and derides and der 58-97-9

FALSE

FALSE

FALSE

down

Lmhn002140C14H14O9eruloyltartaric acid (Fertaric acidhenolic acidhenolic acid

Zmdn001863C18H14O6Dihydroxyl)-benzofuranyl-ferulihcenolic acidhenolic acid

Hmhn00306C15H18O8propionic acid-O-β-D-glucopyrahenolic acidhenolic acid

pmn001419C15H18O81-O-[(E)-p-Cumaroyl]-D-glucosehenolic acidhenolic acid

Zmhn002301C15H18O8p-Coumaric acid-4-O-glucosidehenolic acidhenolic aci1d17405-48-8

Zmzn001549C15H18O8 Ferulic acid-1-O-glucoside henolic acidhenolic acid14364-05-7

Lmsn000363C16H22O7 Raspberryketone glucoside henolic acidhenolic acid38963-94-9

-

-

-

-

up

up

FALSE

FALSE

FALSE

FALSE

FALSE

 

MWSmce19C20H22O4

mws088410H12N5O6 Cyclic 3',5'-Adenylic acid ides and derides and der 60-92-4

Lmmp00308C14H18O9 Mudanoside A henolic acidhenolic acid

pmc0066 10H13N4O7-Deoxyinosine-5'-monophosphaides and derides and der3393-18-8

Lmfn00060C413H16O10 6-O-Galloyl-D-glucose henolic acidhenolic acid13186-19-1

Zmhn00170C313H16O10 5-O-Galloyl-D-hamamelose henolic acidhenolic acid

Hmln00065C913H16O10 3-O-Galloyl-D-glucose henolic acidhenolic acid

mwn002117C14H20O9xyphenyl)ethanediol 1-O-β-D-glhenolic acidhenolic acid

Lmmn00129C14H20O9 Koaburaside

Lmjp002782C18H20O6p-Coumaroyl-7-deoxyrehmaglutTerpenoidsonoterpenoi

pmb3074 C16H18O8 5-O-p-Coumaroylquinic acid henolic acidhenolic acid1899-30-5

pmb4777 C16H18O8oxy-7-methoxycoumarin-β-rhamns and CoumCoumarins

mws1015 C15H16O9 (6,7-DihydroxyCoumarin-6-gluns and CoumCoumarins 531-75-9

pma0149 C15H16O9 Sinapoyl malate henolic acidhenolic acid92344-58-6

Hmcn00412C8 16H20O8P-Methoyxcinnamate glucosidehenolic acidhenolic acid

pmf0400 C22H44O2Docosanoic acid (Behenic acid) Lipids ree fatty acid112-85-6

Dehydrodiisoeugenol

ns and CoumLignans 2680-81-1

3

1

3

3

3

2

3

1

1

3

2

3

3

3

3

3

3

3

3

1

2

3

3

3

1

3

1

1

1

1

3

1

1

3

1

3

2

2

2

3

2

3

3

3

3

3

FALSE

down

-

FALSE

FALSE

FALSE

FALSE

FALSE

FALSE

FALSE

FALSE

up

FALSE

FALSE

FALSE

FALSE

FALSE

FALSE

FALSE

FALSE

down

-

-

-

henolic acidhenolic acid41653-73-0

-

-

-

pmn001420C15H18O9 1-O-[(E)-Caffeoyl]-D-glucose henolic acidhenolic acid

mwn002620C15H18O9Vanillic Acid-4-O-Glucuronidehenolic acidhenolic acid

Zmhn001793C15H18O9 6-O-Caffeoyl-D-glucose henolic acidhenolic acid

-

-

-

mws1080C12H22O11

mws0906 C16H22O8

Jmzp009876C20H22O5

MWSslk092C18H16O7

Lmmn00077C15H20O9

Zmln000899C15H20O9 Syringaldehyde-4-O-glucosidehenolic acidhenolic acid

Lmmn00468C20H24O5 Nectandrin B

mws060910H12N5O7anosine 3',5'-cyclic monophosphides and derides and der7665-99-8

Cmrn002988C16H26O8 Kankanoside A Terpenoidsonoterpenoi

pme1816 C16H18O9orogenic acid (5-O-Caffeoylquinhenolic acidhenolic acid 906-33-2

mws0748 C16H18O9

pmd0209 C20H18O6

Lmhp112042C21H38O4

Lmhp012042C21H38O4

Lmhn00158C015H16O10

Lmdn009099C20H20O6

pmb0296 C21H40O4

mws0179 C17H20O9 Chlorogenic acid methyl ester henolic acidhenolic acid29708-87-0

pmb0752 C17H20O9 3-O-Feruloylquinic acid henolic acidhenolic acid1899-29-2

pma3724 C17H20O9 1-O-Feruloylquinic acid henolic acidhenolic acid

Lmmp00438C19H30O7 3-Oxo-α-ionol-β-D-glucoside Others Others

Hmlp002744C20H22O7 Nortrachelogenin

Lmzn00198C316H24O1e0o-guaiacylglycerol-7-O-β-D-gluhenolic acidhenolic acid

Hmap01052C9 24H30O4

Zmhn00222C717H22O10

Lmzp006972C22H26O6

Galactinol

Coniferin

magnovatins A

Usnic acid

Others rides and A 3687-64-7

henolic acidhenolic acid 531-29-3

FALSE

FALSE

up

ns and CoumLignans

-

henolic acidhenolic acid 125-46-2

Dihydrocaffeoylglucose henolic acidhenolic acid

-

-

FALSE

FALSE

FALSE

FALSE

FALSE

FALSE

FALSE

FALSE

FALSE

FALSE

FALSE

up

FALSE

FALSE

FALSE

FALSE

FALSE

FALSE

FALSE

down

ns and CoumLignans 74683-16-2

-

1-Caffeoylquinic acid

Sesamin

henolic acidhenolic acid1241-87-8

ns and CoumLignans 607-80-7

Lipids Glycerol este2277-28-3

Lipids Glycerol este3443-82-1

1-Linoleoylglycerol

2-Linoleoylglycerol

Sinapoyltartaric acid

Coniferyl ferulate

1-Oleoyl-Sn-Glycerol

henolic acidhenolic acid

-

henolic acidhenolic acid63644-62-2

Lipids Glycerol est1e29784-87-8

-

-

ns and CoumLignans 34444-37-6

-

-

-

farnesiferol B

4-O-Glucosyl-sinapate

Dimethylmatairesinol

ns and CoumCoumarins

henolic acidhenolic acid

FALSE

up

ns and CoumLignans 25488-59-9

Smgp009046C22H26O6 pinoresinol dimethy ether ns and CoumLignans 29106-36-3

up

 

Hmcn00141C517H24O10

Majoroside

Others

Others 134140-02-6

3

3

2

3

3

3

3

2

2

3

2

1

3

3

3

3

3

1

1

3

3

2

1

1

3

3

3

3

3

3

3

3

3

3

3

3

3

1

3

3

3

3

3

3

2

3

FALSE

FALSE

FALSE

FALSE

FALSE

FALSE

FALSE

FALSE

FALSE

up

Lmzn001582C18H28O9 5'-Glucosyloxyjasmanic acid henolic acidhenolic acid

-

-

Zmjp003597C19H32O8

Cmmn00523C20H24O8

Zmjp004455C19H26O9

pmc0281 15H21N5O

Ampelopsisionoside

massoniresinol

Trollioside

Others

Others

ns and CoumLignans 96087-10-4

henolic acidhenolic acid

ides and derides and der

-

-

Ribosyladenosine

mws0043 C21H22O8tin (5,6,7,8,3',4'-HexamethoxyflFlavonoids Flavones 478-01-3

Hmln00352C918H26O10 Benzyl β-primeveroside henolic acidhenolic aci1d30622-31-0

Cmjn00433C718H26O10Benzyl-(2''-O-xylosyl)glucosidehenolic acidhenolic acid

-

-

Hmlp00314C221H27NO7

Cmdp00307C7 20H22O9

Trachelogenin amide

Salviifoside A

Alkaloids Alkaloids

henolic acidhenolic acid175539-71-

FALSE

FALSE

FALSE

FALSE

FALSE

up

FALSE

FALSE

FALSE

down

Hmhp00258C19H24O10 Methylpicraquassioside A ns and CoumCoumarins

-

-

-

Lmhp00833719H40NO7 LysoPE 14:0(2n isomer)

pmb0864 19H40NO7 LysoPE 14:0

Lipids

Lipids

LPE

LPE

pme21170H15N5O10 Adenosine 5'-diphosphate ides and derides and der 58-64-0

Cmbn00399C619H26O11 Regaloside G henolic acidhenolic aci1d20601-64-1

mws0919C21H20O10ol-3-O-rhamnoside (Afzelin)(KaFlavonoids Flavonols 482-39-3

Lmtn00232C419H28O1B1 enzyl-(2''-O-glucosyl)glucosidehenolic acidhenolic acid

-

Lmyn00302C819H28O11 Benzyl-β-gentiobioside

henolic acidhenolic acid56775-64-5

henolic acidhenolic aci1d49596-95-2

Flavonoids Flavonols

Lmjn00253C419H28O11

Lmfp00405C520H18O11

Cmzn00525C121H22O10

Darendoside A

Morin-3-O-xyloside

6-O-Caffeoylarbutin

-

up

henolic acidhenolic aci1d36172-60-6

FALSE

FALSE

FALSE

FALSE

FALSE

FALSE

up

Lmhp00888520H42NO7 LysoPE 15:0(2n isomer)

Lmhp00918720H42NO7 LysoPE 15:0

Lipids

Lipids

LPE

LPE

-

-

Lmhp00555024H28N2O N,N'-Diferuloylputrescine AlkaloidsPhenolamine42369-86-8

MWSHY002C22H18O10 Epicatechin gallate Flavonoids Flavanols 1257-08-5

Lmhn00496C022H18O10 7-O-Galloyltricetiflavan Flavonoids Flavanols

pmp000090C20H26O11 Regaloside D henolic acidhenolic aci1d20601-66-3

-

mws0045C21H20O11ercetin-3-O-rhamnoside(QuercitrFlavonoids Flavonols 522-12-3

pme2459 C21H20O1u1teolin-7-O-glucoside (CynarosidFlavonoids Flavones 5373-11-05

up

up

Hmpp00327C21H20O11 Luteolin-4'-O-glucoside

Xmyp00565C21H20O11 Kaempferol-4'-O-glucoside Flavonoids Flavonols

MWSHY013C21H20O1e1mpferol-3-O-glucoside (AstragaFlavonoids Flavonols 480-10-4

Flavonoids Flavones 6920-38-3

up

up

up

-

Jmgn00402C121H22O11C-Glucosyl-2-HydroxynaringenFlavonoidsFlavanones

HJN086 C21H22O11 Eriodictyol-3'-O-glucoside FlavonoidsFlavanones

-

-

FALSE

up

pmb3023C21H22O11 Eriodictyol-8-C-glucoside FlavonoidsFlavanones023271-51-

Lmtn00279C621H22O11 Aromadendrin-7-O-glucoside FlavonoidsFlavanonols28189-90-4

FALSE

FALSE

FALSE

down

FALSE

FALSE

FALSE

FALSE

FALSE

FALSE

FALSE

pmb0876 21H44NO7

pmd0160 21H44NO7 LysoPE 16:0(2n isomer)

pmb0464 16H27NO1L-Aspartic acid-O-diglucoside cids and decids and de

LysoPE 16:0

Lipids

Lipids

LPE 53862-35-4

LPE

-

-

mws0034C22H18O11 Epigallocatechin-3-gallate Flavonoids Flavanols 989-51-5

mws2220C22H18O11 Gallocatechin 3-O-gallate Flavonoids Flavanols 5127-64-0

Cmyp00206C422H22O11

MWSslk01C322H22O11

Yuanhuanin

Tectoridin

Flavonoids Flavones 83133-14-6

FlavonoidsIsoflavones 611-40-5

Lmpn00620C822H22OM11ethoxykaempferol-7-O-rhamnosFlavonoids Flavonols

Lmjp00365C522H22O11C-MethylKaempferol-3-glucosidFlavonoids Flavones

-

-

 

mws0091C21H20O1e2rcetin-3-O-glucoside (IsoquercitFlavonoids Flavonols 482-35-9

mws0856C21H20O1e2rcetin-4'-O-glucoside (SpiraeosiFlavonoids Flavonols 20229-56-5

pme1598C22H24O11 Hesperetin-5-O-glucoside FlavonoidsFlavanones69651-80-5

1

1

3

1

3

1

1

3

1

1

1

1

1

1

1

1

1

1

3

3

1

3

3

3

3

3

3

3

3

3

3

2

3

1

1

3

3

3

2

1

1

3

3

3

3

1

up

up

up

Lmhp00946422H44NO7 LysoPE 17:1(2n isomer)

Lipids

henolic acidhenolic acid407163-62-

Lipids LPC 20559-16-4

Terpenoids Triterpene71850-15-2

LPE

-

FALSE

up

FALSE

up

Lman00407C122H26O11

pmd0130 22H46NO7

Hmjn008136C30H46O4

Grevilloside M

LysoPC 14:0

Camaldulenic acid

Lmsn012627C30H46O4-Dihydroxyurs-12,18-dien-28-oiTerpenoids Triterpene

-

-

up

up

up

up

Zmjn012003C30H48O4

Lmzn106284C30H48O4

Lmzn006284C30H48O4

Lmzn006169C30H48O4

Zmpn008194C30H48O4

Gypensapogenin F

Alphitolic acid

2α-Hydroxyursolic acid

Pomolic acid

Terpenoids Triterpene

Terpenoids Triterpene19533-92-7

Terpenoids Triterpene

Terpenoids Triterpene13849-91-7

Terpenoids Triterpene 4547-24-4

-

FALSE

up

Corosolic acid

pmn001706C30H48O4 2-Hydroxyoleanolic acid Terpenoids Triterpene26707-60-8

pmn001705C30H48O4roxy-17,21-semiacetal-12(13)oleTerpenoids Triterpene

up

up

-

mws1610 C30H48O4

Hmbn00520C30H48O4

Maslinic acid

Hederagenin

Terpenoids Triterpene 4373-41-5

Terpenoidserpene Sapo465-99-6

up

FALSE

FALSE

FALSE

FALSE

FALSE

FALSE

up

pmb1912 20H23N7O10-Formyltetrahydrofolic Acid Alkaloids Alkaloids 2800-34-2

MWSmce62C22H18O12 Cichoric Acid henolic acidhenolic acid6537-80-0

pmb0545C23H25O11 Rosinidin-3-O-glucoside Flavonoidsnthocyanidi

pmb0881 23H44NO7 LysoPE 18:2 Lipids LPE

Zmhn00490C921H18O13 3,4-Digalloylshikimic acid henolic acidhenolic acid

Lmcp00378C822H22O12 Isotamarixin Flavonoids Flavonols

-

-

-

-

Hmcp00220C722H22O12hamnetin-7-O-glucoside (BrassiFlavonoids Flavonols 6743-96-0

Lmjp00290C622H22O12 Rhamnetin-3-O-Glucoside Flavonoids Flavonols 27875-34-9

Lmsn00416C222H22O1G2alloyl-2-O-p-Coumaroyl-β-D-ghl enolic acidhenolic acid94356-18-0

Lmjp00304C422H22O12 Isorhamnetin-3-O-Glucoside Flavonoids Flavonols 5041-82-7

Lmjp00329C522H22O1M2 ethoxykaempferol-3-O-glucosiFlavonoids Flavonols 63422-27-5

FALSE

up

FALSE

FALSE

up

Hmgp00203C22H22O12 Nepetin-7-O-glucoside

pmb0856 23H46NO7 LysoPE 18:1(2n isomer)

Flavonoids Flavones 569-90-4

FALSE

FALSE

FALSE

down

up

Lipids

Lipids

Lipids

LPE

LPE 89576-29-4

LPC

-

mws0289 23H46NO7

pmb2260 23H46NO7

LysoPE 18:1

LysoPC 15:1

-

Lmdp00296C921H20O13 Myricetin-3-O-galactoside Flavonoids Flavonols 15648-86-9

pmb2319 23H48NO7

Lmhp00912923H48NO7 LysoPC 15:0(2n isomer)

Li512114 C31H50O4 Corosolic Acid Methyl Ester Terpenoids Triterpene 4518-70-1

LysoPC 15:0

Lipids

Lipids

LPC 108273-89-8

LPC

FALSE

down

up

-

Hmhn00781C30H48O5 2α,3α-Dihydroxyursolic acid Terpenoids Triterpene

-

-

-

up

pmb0863 24H46NO7 LysoPC 16:2(2n isomer)

pmb0790 20H31NO1 Pyridoxine-5'-O-diglucoside

Lipids

Others

Lipids

Lipids

LPC

Vitamin

LPC 76790-27-7

LPC

down

FALSE

FALSE

FALSE

FALSE

up

FALSE

FALSE

FALSE

pmp00127024H48NO7

LysoPC 16:1

Lmhp00883324H48NO7 LysoPC 16:1(2n isomer)

HJAP005C22H22O13 Laricitrin-3-O-glucoside

-

Flavonoids Flavonols 39986-90-8

Lmsn00432C122H22O1O3-Caffeoyl-3-O-galloyl-β-D-gluchenolic acidhenolic acid

Lmjp00323C122H22O13 Patuletin-3-O-glucoside Flavonoids Flavonols

Lmtn00299C722H22O13 Mearnsetin-3-O-glucoside Flavonoids Flavones

pmb0855 24H50NO7 LysoPC 16:0 Lipids

-

-

-

LPC 17364-16-8

 

pmd0132 24H50NO7 LysoPC 16:0(2n isomer)

Lipids

LPC

-

-

1

3

1

3

3

1

1

1

2

1

1

1

1

1

1

3

3

1

3

1

1

3

2

3

3

2

1

1

3

2

2

3

1

3

1

3

3

3

1

3

3

3

3

3

2

3

down

FALSE

up

Lmhn00366C824H22O1p2-Coumaroylsinapoyltartaric acidhenolic acidhenolic acid

pme0500C18H32O16

MWSmce48C18H32O16

pme2125C18H32O16

Lmhp00871825H48NO7

Lmhp00959025H50NO7

Lmhp01051525H52NO7 LysoPC 17:0(2n isomer)

pmb2406 25H52NO7

Hmqp0062326H46NO7

Lmhp010334C27H48O9-Linoleoylglycerol-1-O-glucosid Lipids Glycerol este

pmb0865 26H48NO7 LysoPC 18:3(2n isomer)

pmb0854 26H48NO7

pmp00127326H50NO7

pmp00125126H50NO7 LysoPC 18:2(2n isomer)

D-Melezitose

Manninotriose

Raffinose

LysoPC 17:2

LysoPC 17:1

Others rides and A 597-12-6

Others rides and A13382-86-0

Others rides and A 512-69-6

down

FALSE

FALSE

down

FALSE

down

Lipids

Lipids

Lipids

Lipids

Lipids

LPC

LPC

LPC

-

-

-

LysoPC 17:0

LysoPC 18:4

LPC 50930-23-9

LPC

-

-

-

-

-

-

-

-

down

FALSE

FALSE

FALSE

FALSE

FALSE

FALSE

FALSE

FALSE

up

Lipids

Lipids

Lipids

Lipids

LPC

LPC

LPC

LPC

LysoPC 18:3

LysoPC 18:2

Li512111C24H24O13rhamnetin-3-O-(6''-acetylglucosiFlavonoids Flavonols

pmp001059C22H32O14nipin-1-O-(2''-O-apiosyl)glucosiTerpenoidsonoterpenoi

Cmsp00308C326H32O11drodiconiferyl alcohol-4-O-gluc Others

Others 107870-88-2

pmn001378C26H32O11 Pinoresinol-4-O-glucoside ns and CoumLignans 41607-20-9

Lmsp00365C526H32O11diconiferyl alcohol-gamma'-O-ghenolic acidhenolic acid

pmp00128126H52NO7 LysoPC 18:1 Lipids LPC

pmb3041C23H22O14 Tricin-7-O-saccharic acid Flavonoids Flavones

-

-

-

FALSE

down

up

pmn001710C24H26O13Rosmarinic acid-3'-O-glucosidehenolic acidhenolic aci9d10028-78-3

Lmmn00227C26H34O11Isolariciresinol-9'-O-glucoside ns and CoumLignans 63358-12-3

FALSE

FALSE

FALSE

up

Lmyn00397C126H34O1r1odiconiferyl alcohol-4-O-β-D-gnl s and CoumLignans

-

HJN083 C26H34O11 Lariciresinol-4'-O-glucoside ns and CoumLignans 143663-00-7

mws0126 26H54NO7

pmd0136 26H54NO7 LysoPC 18:0(2n isomer)

Zmcn00515C626H26O1O2-Dicaffeoylquinic Acid Methylhenolic acidhenolic acid

pmb2554C23H30O14-O-Feruloyl quinic acid glucosidhenolic acidhenolic acid

pmb2833C23H30O1O4 -Feruloylquinic acid-O-glucosihenolic acidhenolic acid

Hmln00210C026H32O12Nortrachelogenin-4-O-glucosidens and CoumLignans 33464-78-7

Zmcn00372C926H32O1H2ydroxypinoresinol-4'-O-Glucosns and CoumLignans

Lmtn00450C026H32O12 Prupersin C henolic acidhenolic acid

pmn001375C26H32O1H2ydroxypinoresinol-1-O-Glucosnis and CoumLignans 81495-71-8

Lmdn00192C526H34O12 Olivil-4'-O-glucoside ns and CoumLignans 76880-93-8

pmd0146 28H54NO7 LysoPC 20:2(2n isomer)

pmd0147 28H54NO7

Lmhp01154928H56NO7

LysoPC 18:0

Lipids

Lipids

LPC 19420-57-6

FALSE

down

LPC

-

-

-

-

FALSE

FALSE

FALSE

FALSE

FALSE

FALSE

FALSE

FALSE

down

FALSE

down

FALSE

FALSE

up

-

-

Lipids

Lipids

Lipids

LPC

LPC

LPC

-

-

-

-

-

-

-

-

LysoPC 20:2

LysoPC 20:1

Lmmn00226C27H36O12ethoxyisolariciresinol-9'-O-glucnos and CoumLignans

Lmhn00380C226H26O14 Sinapoylsinapoyltartaric acid henolic acidhenolic acid

HJAP064C25H24O1h5amnetin-3-O-(6''-malonylglucosFlavonoids Flavonols

Zmhp00513C925H24O1m5arixetin-3-O-(6''-malonyl)glucoFlavonoids Flavonols

Zmhn00144C628H36O1y3ringaresinol-4'-O-glucopyranosns and CoumLignans

FALSE

FALSE

FALSE

FALSE

Lmjp00187C726H29O15bubioside [Cyanidin-3-O-(2''-O-Flavonoidsnthocyanidi63535-17-1

Lmtp00404C426H28O16rcetin-3-O-apiosyl(1→2)galactoFlavonoids Flavonols

-

 

Lmmp00309C27H30O16cetin-3-O-(4''-O-glucosyl)rhamnFlavonoids Flavonols 59262-54-3

Zmxp00310C727H30O16 Luteolin-7,3'-di-O-glucoside Flavonoids Flavones 52187-80-1

3

1

1

1

3

3

2

3

3

3

3

3

3

1

1

1

1

3

3

3

3

3

3

2

1

3

1

1

1

1

3

3

2

2

3

3

3

3

3

3

2

1

3

3

1

1

up

up

Lmbp00259C227H30O16Kaempferol-3,7-di-O-glucoside Flavonoids Flavonols

Hmpp00261C27H30O16 Luteolin-7-O-gentiobioside Flavonoids Flavones

-

-

-

up

up

pmp000686C28H34O15

Forsythiaside J

henolic acidhenolic acid

FALSE

FALSE

up

FALSE

up

pme0001C28H34O15in-7-O-neohesperidoside(NeoheFlavonoidsFlavanones13241-33-3

Lmcp00443C630H27O14anidin-3-O-(6''-O-caffeoyl)glucosFlavonoidsnthocyanidi

Zmcp00292C427H31O16nidin-3-O-(2''-O-glucosyl)glucoFlavonoidsnthocyanidi38820-68-7

Cmxp00353C127H32O16 Okanin-4'-O-gentiobioside Flavonoids Chalcones

Zmgp00333C527H32O16 okanin 3',4'-di-O-glucoside* Flavonoids Chalcones

-

-

-

up

Lmyp00313C928H24O16ercetin-3-O-(6''-galloyl)galactosFlavonoids Flavonols 53171-28-1

FALSE

FALSE

up

Lmfp00302C628H26O16

Hmlp00494C328H32O16

Maplexin H

Nitensoside B

Tannins Tannin 357621-91-

ns and CoumCoumarins

-

-

-

pmp001310C27H30O1y7droxykaempferol-3,6-O-DiglucoFlavonoids Flavonols

Zmhp00378C427H30O17 Rhodioflavonoside Flavonoids Flavonols

up

up

Zmhp00273C027H30O1H7ydroxykaempferol 6,7-DiglucosFlavonoids Flavonols142674-16-6

Lmtp00367C727H30O17rcetin-3-O-sophoroside (BaimasFlavonoids Flavonols 18609-17-1

up

up

pmb0645 C28H34O1p6eretin-6-C-glucoside-7-O-glucoFlavonoidsnoid carbon

mwn00430C830H38O15β-D-[6-O-(4-O-β-D-glucopyranohenolic acidhenolic acid

Lmhp00280C028H32O17y,5-methoxyGenistein-4',7-O-diFlavonoidsIsoflavones

-

-

-

-

-

-

-

-

up

FALSE

FALSE

FALSE

FALSE

FALSE

FALSE

FALSE

down

up

Hmgp00188C28H32O17 Patuletin-3-O-rutinoside

Flavonoids Flavonols

Lmpp0032C2831H29O15unidin-3-O-(6''-O-caffeoyl)glucoFlavonoidsnthocyanidi

pmb0578 C32H30O1u5teolin-7-O-(6''-sinapoyl)glucosiFlavonoids Flavones

P4269 C36H62O10

P4195 C36H62O10

mws1153C31H38O16

mws4163C24H42O21

ginsenoside Ki

chikusetsusaponin L9a

2'-Acetylacteoside

Nystose

Terpenoidserpene Sapo

Terpenoidserpene Sapo

henolic acidhenolic acid94492-24-7

Others rides and A13133-07-8

Lmhp00938C433H58O14inoleoylglycerol-2,3-di-O-glucos Lipids Glycerol este

Lmhp00919C033H58O14inoleoylglycerol-1,3-di-O-glucos Lipids Glycerol este

pmp001271C33H58O14Linoleoyl-sn-glycerol-diglucosid Lipids ree fatty acid

pmn001370C32H42O1P6inoresinol-4,4'-O-di-O-glucosidns and CoumLignans 63902-38-5

pmn001743C31H42O17 Specnuezhenide henolic acidhenolic acid39011-92-2

MWSmce49C32H46O16Secoisolariciresinol diglucosidens and CoumLignans 257930-74-8

Lmgn00225C331H36O18ngoylcaffeoylquinic acid-D-gluchenolic acidhenolic acid

pmn001369C32H42O171-Hydroxypineolin Diglucosidens and CoumLignans

Hmln10166C432H42O1o7rtrachelogenin-4'-O-gentiobiosins and CoumLignans

pmn001368C32H44O17 Olivil-4,4'-Di-O-glucoside ns and CoumLignans

-

-

-

FALSE

FALSE

FALSE

FALSE

FALSE

FALSE

down

FALSE

FALSE

FALSE

FALSE

FALSE

FALSE

FALSE

up

-

-

-

-

-

-

-

P3643 C41H68O12

P4685 C41H70O12

notoginsenoside T5

ginsenoside Mc

Terpenoidserpene Sapo

Terpenoidserpene Sapo

pmn001505C41H64O1o3lic acid-3-O-xylosyl(1→3)glucuTerpenoidserpene Sapo

Cmrp00836C042H70O12 Ginsenoside Rg6

Lmxp06577C41H70O13 Chikusetsusaponin LM1 Terpenoidserpene Sapo

mws4038C41H70O13 Notoginsenoside R2 Terpenoidserpene Sapo80418-25-3

Zmcn00420C636H36O1k9aempferol-3-caffeoyldiglucosideFlavonoids Flavonols

Terpenoidserpene Sap1o47419-93-0

-

FALSE

FALSE

FALSE

FALSE

-

P4501 C42H70O13 Chikusetsusaponin LT8

mws4035C42H72O13 Ginsenoside F2

Terpenoidserpene Sapo69359-06-4

Terpenoidserpene Sapo62025-49-4

 

P4045 C41H70O14

notoginsenoside Rw2

Terpenoidserpene Sapo

-

-

1

3

1

1

1

1

1

1

3

3

1

1

2

1

3

3

1

1

1

1

1

2

1

1

1

3

3

3

3

FALSE

FALSE

FALSE

FALSE

FALSE

FALSE

FALSE

FALSE

FALSE

FALSE

FALSE

FALSE

FALSE

FALSE

FALSE

FALSE

FALSE

FALSE

FALSE

FALSE

FALSE

up

pmb0676 C38H40O19l-8-C-glucoside-7-O-(6''-feruloyFlavonoidsnoid carbon

mws1553C42H72O14

HJN032 C42H72O14

mws1665C42H72O14

Ginsenoside Rg1

Sanchirhinoside A4

Ginsenoside Rf

Terpenoidserpene Sapo22427-39-0

Terpenoidserpene Sapo

Terpenoidserpene Sapo52286-58-5

-

Lmxp05341C42H72O14(24R)-pseudo ginsenoside F11 Terpenoidserpene Sapo69884-00-0

Lmxp05257C43H68O14hikusetsusaponin Iva methyl esteTerpenoidserpene Sapo58546-61-5

Lmxp05320C43H68O1C4hikusetsusaponin IV methyl esteTerpenoidserpene Sapo

-

-

-

-

P3859 C42H72O15

P4308 C42H74O15

ginsenoside 26

quinquenoside L9*

Yesanchinoside D

Vina-Ginsenoside R1

Phanoside

Notoginsenoside Fd

Notoginsenoside Fe

quinquenoside L10

Notoginsenoside R1

Ginsenoside Rd

Terpenoidserpene Sapo

Terpenoidserpene Sapo

Terpenoidserpene Sapo

Terpenoidserpene Sap7o84213-14-5

Terpenoidserpene Sapo

Terpenoidserpene Sapo80321-63-7

Terpenoidserpene Sapo88105-29-7

Terpenoidserpene Sapo

Terpenoidserpene Sapo80418-24-2

Terpenoidserpene Sapo

Terpenoids Triterpene80321-69-3

Lmxp06071C44H74O15

HJN033 C44H74O15

Zmjp00677C147H78O16

Zmsp00769C847H80O17

Hmsn00509C047H80O17

P5367 C48H80O17

mws1721C47H80O18

Lmzp00542C748H82O18

Hmsn00459C648H82O18

Hmsn00320C048H82O18

-

-

-

Gypenoside XVII

Notoginsenoside K

Terpenoidserpene Sapo

-

-

Lmxn05454C48H82O19 Chikusetsusaponin LM4 Terpenoidserpene Sapo

Lmzn00417C848H82O19

Hmsn00278C548H82O19

Hmsn00301C548H82O19

Lmxn00676C848H76O20β,6α-trihydroxy-23-oxoolean-12Terpenoidserpene Sapo

Hmsn00434C953H90O22 Notoginsenoside L Terpenoidserpene Sapo

Cmsn00141C953H84O23 Ginsenoside Ro O-Pentoside Terpenoidserpene Sapo

Zmjp00644C154H90O23syl)oxy]-20-[(6-O-alpha-L-rhamTerpenoidserpene Sapo

Hmsn00335C554H92O24

Majoroside F1

Notoginsenoside N

Notoginsenoside M

Terpenoidserpene Sap1o14128-16-4

Terpenoidserpene Sapo

Terpenoidserpene Sapo

-

-

-

-

-

-

FALSE

FALSE

FALSE

up

FALSE

FALSE

FALSE

Notoginsenoside A

Terpenoidserpene Sap1o93895-21-5

 

Table S2

YA_vs_DYLA_vs_DY YYA1

YYA2

YYA3

XLA1

XLA2

XLA3

DYA1

DYA2

up

up

1.53E+06 2.06E+06 1.50E+06 3.12E+06 1.38E+06 1.71E+06 5.25E+06 5.06E+06

FALSE FALSE 1.44E+06 4.01E+06 2.10E+06 9.28E+05 1.18E+06 9.02E+05 8.81E+05 1.00E+06

FALSE FALSE 1.10E+04 8.42E+03 1.56E+04 2.52E+04 4.34E+04 3.77E+04 1.91E+04 2.32E+04

down

up

up

down

FALSE

up

FALSE 5.66E+06 6.57E+06 5.94E+06 3.36E+06 2.55E+06 2.37E+06 2.82E+06 1.56E+06

FALSE 2.28E+07 2.80E+07 2.51E+07 4.43E+07 2.15E+07 2.36E+07 5.48E+07 5.13E+07

FALSE 7.60E+05 1.22E+06 7.82E+05 1.90E+06 1.18E+06 1.33E+06 1.83E+06 2.03E+06

down 4.40E+04 3.78E+04 1.58E+04 2.82E+04 2.18E+04 1.39E+04 1.11E+04 8.56E+03

up

up

up

up

up

up

up

up

up

up

1.61E+04 3.31E+03 2.58E+04 9.44E+03 3.03E+03 3.35E+03 1.00E+04 1.36E+04

3.62E+04 6.86E+04 4.08E+04 1.07E+05 3.92E+04 4.54E+04 1.75E+05 1.46E+05

6.94E+06 9.97E+06 7.19E+06 1.36E+07 6.29E+06 7.15E+06 2.52E+07 2.50E+07

1.34E+04 4.71E+03 1.07E+04 1.47E+04 8.45E+03 7.69E+03 3.62E+04 5.68E+04

1.22E+06 1.79E+06 1.31E+06 2.82E+06 1.11E+06 1.31E+06 4.54E+06 4.43E+06

2.15E+07 2.70E+07 2.16E+07 3.61E+07 1.98E+07 2.42E+07 6.71E+07 6.42E+07

1.77E+06 2.34E+06 1.72E+06 3.33E+06 1.52E+06 1.87E+06 5.48E+06 5.44E+06

2.21E+07 2.81E+07 2.24E+07 3.68E+07 2.03E+07 2.37E+07 6.75E+07 6.43E+07

2.24E+07 2.77E+07 2.27E+07 3.72E+07 2.07E+07 2.40E+07 6.88E+07 6.68E+07

1.28E+06 1.72E+06 1.22E+06 2.46E+06 1.15E+06 1.25E+06 4.45E+06 4.45E+06

up

up

up

up

up

up

up

up

FALSE

up

down 2.43E+04 1.97E+04 2.43E+04 3.20E+04 3.28E+04 8.90E+04 1.50E+04 1.09E+04

up

up

up

up

up

1.40E+05 8.06E+04 1.45E+05 4.65E+04 3.09E+04 3.99E+04 3.62E+05 2.05E+05

1.86E+05 3.18E+05 1.40E+05 2.27E+05 2.13E+05 1.83E+05 7.94E+05 7.39E+05

1.73E+05 3.01E+05 1.50E+05 2.32E+05 1.95E+05 1.74E+05 7.43E+05 6.64E+05

1.73E+05 1.43E+05 1.57E+05 5.18E+04 4.82E+04 4.03E+04 2.69E+05 1.93E+05

9.30E+04 5.59E+04 7.86E+04 2.06E+04 1.46E+04 1.24E+04 1.26E+05 8.19E+04

up

up

FALSE

FALSE

FALSE

FALSE

down 4.78E+04 2.58E+04 3.50E+04 1.13E+05 7.69E+04 1.13E+05 1.91E+04 6.00E+04

down 4.74E+06 7.11E+06 4.35E+06 7.98E+06 8.19E+06 8.74E+06 2.89E+06 4.32E+06

FALSE FALSE 9.55E+04 3.28E+04 9.94E+04 4.04E+05 1.13E+05 2.92E+05 9.22E+04 1.56E+05

down

up

up

down 1.73E+06 1.61E+06 1.72E+06 3.03E+06 1.84E+06 2.36E+06 6.24E+05 6.56E+05

FALSE 2.46E+05 1.54E+05 3.03E+05 6.86E+05 3.50E+05 6.21E+05 3.56E+05 4.90E+05

FALSE 1.00E+05 5.81E+04 1.34E+05 2.85E+05 1.35E+05 2.51E+05 1.56E+05 2.21E+05

FALSE FALSE 1.32E+05 1.99E+05 1.41E+05 5.14E+05 3.89E+05 1.82E+05 1.50E+05 1.72E+05

FALSE

FALSE

up

FALSE

FALSE

up

up

up

up

up

up

up

3.72E+06 3.30E+06 4.45E+06 2.87E+06 1.86E+06 9.01E+05 9.18E+06 8.34E+06

3.32E+06 2.98E+06 3.68E+06 2.27E+06 9.39E+05 8.11E+05 8.27E+06 6.98E+06

8.69E+04 1.29E+05 1.11E+05 1.73E+05 8.49E+04 8.79E+04 3.09E+05 2.55E+05

3.70E+06 5.19E+06 3.86E+06 5.24E+06 3.29E+06 3.20E+06 9.87E+06 7.89E+06

3.39E+06 3.93E+06 2.95E+06 3.77E+06 2.54E+06 2.62E+06 7.66E+06 6.48E+06

5.23E+04 5.70E+04 3.87E+04 6.33E+04 3.28E+04 4.01E+04 1.29E+05 1.18E+05

FALSE

up

FALSE

FALSE

FALSE

up

down 1.68E+05 6.90E+04 1.85E+05 1.93E+05 1.27E+05 1.51E+05 1.94E+04 5.21E+04

FALSE 1.09E+05 5.39E+04 9.40E+04 1.65E+05 1.12E+05 1.90E+05 1.65E+05 2.37E+05

down 2.59E+05 2.65E+05 2.95E+05 1.00E+06 5.61E+05 8.87E+05 1.48E+05 2.58E+05

down 4.16E+06 2.95E+06 4.16E+06 1.21E+07 6.34E+06 1.01E+07 1.62E+06 3.74E+06

down 3.84E+04 9.61E+04 2.92E+04 1.17E+05 1.39E+05 1.31E+05 1.27E+04 2.89E+04

FALSE 1.98E+05 1.63E+05 1.83E+05 2.50E+05 5.39E+05 5.06E+05 7.17E+05 5.67E+05

up

down

up 1.41E+05 1.42E+05 1.81E+05 1.54E+05 1.88E+05 1.55E+05 3.81E+05 3.92E+05

down 2.09E+04 9.14E+03 2.13E+04 2.51E+04 2.32E+04 1.88E+04 9.43E+03 7.34E+03

 

up

up

1.25E+05 1.02E+05 1.06E+05 2.02E+05 1.34E+05 1.34E+05 7.06E+05 4.08E+05

FALSE

up

down 7.10E+04 3.27E+04 6.29E+04 1.53E+05 8.45E+04 1.61E+05 4.01E+04 7.52E+04

FALSE 8.02E+06 1.24E+07 7.73E+06 1.83E+07 1.16E+07 1.39E+07 1.83E+07 1.92E+07

FALSE 4.40E+06 6.65E+06 4.35E+06 1.06E+07 6.46E+06 7.91E+06 1.03E+07 1.12E+07

down 6.39E+04 5.26E+04 5.26E+04 9.44E+04 1.23E+05 8.64E+04 6.94E+03 1.12E+04

down 1.31E+05 9.42E+04 1.05E+05 3.37E+05 2.48E+05 3.09E+05 1.26E+05 1.81E+05

down 8.34E+04 6.40E+04 1.20E+05 7.72E+04 5.85E+04 1.05E+05 1.66E+04 4.26E+04

down 1.20E+05 4.51E+05 2.24E+05 9.18E+04 1.50E+05 1.27E+05 2.63E+04 6.84E+04

up

down

FALSE

down

down

FALSE

FALSE

FALSE

up

up

up

1.77E+05 5.90E+04 1.55E+05 6.16E+04 3.69E+04 3.45E+04 1.25E+05 1.03E+05

1.36E+07 4.42E+06 1.22E+07 4.82E+06 2.69E+06 3.48E+06 1.20E+07 9.84E+06

down 8.53E+04 7.67E+04 8.24E+04 2.95E+05 3.18E+05 3.36E+05 1.40E+05 1.13E+05

up

up

2.53E+04 1.78E+04 2.81E+04 3.02E+04 3.32E+04 2.90E+04 1.26E+05 1.39E+05

1.60E+06 1.12E+06 1.62E+06 1.07E+06 6.89E+05 8.00E+05 2.06E+06 1.73E+06

FALSE

FALSE FALSE 1.79E+05 1.22E+05 1.46E+05 1.05E+05 3.24E+04 8.12E+04 6.06E+04 7.95E+03

FALSE FALSE 7.20E+05 1.04E+06 6.12E+05 3.19E+05 2.88E+04 1.06E+05 1.68E+05 3.55E+04

FALSE FALSE 4.67E+04 4.25E+04 5.27E+04 2.71E+04 1.82E+04 2.42E+04 2.34E+04 1.57E+04

up

up

up

up

FALSE 2.94E+05 1.59E+05 3.16E+05 9.51E+05 3.15E+05 5.27E+05 3.82E+05 7.54E+05

FALSE 2.17E+05 7.94E+04 1.74E+05 4.07E+05 1.22E+05 3.43E+05 5.89E+05 3.59E+05

FALSE 1.88E+05 7.38E+04 1.77E+05 3.71E+05 1.05E+05 2.95E+05 4.93E+05 3.25E+05

FALSE 2.11E+05 6.64E+04 1.60E+05 3.89E+05 1.35E+05 3.59E+05 5.61E+05 3.40E+05

FALSE FALSE 8.41E+04 4.31E+04 5.69E+04 1.35E+05 9.61E+04 1.55E+05 1.18E+05 1.40E+05

FALSE down 4.45E+06 4.01E+06 5.65E+06 6.94E+06 1.10E+07 1.19E+07 1.64E+06 3.20E+06

FALSE FALSE 4.89E+06 3.82E+06 5.50E+06 6.80E+06 1.22E+07 1.27E+07 1.78E+06 3.52E+06

FALSE FALSE 3.83E+06 3.14E+06 3.96E+06 6.31E+06 1.01E+07 9.96E+06 1.47E+06 3.15E+06

FALSE FALSE 4.90E+06 3.45E+06 5.82E+06 7.72E+06 1.19E+07 1.26E+07 1.61E+06 3.53E+06

FALSE FALSE 4.87E+06 3.33E+06 5.61E+06 7.82E+06 1.29E+07 1.22E+07 1.67E+06 3.70E+06

FALSE

down 5.81E+04 4.00E+04 6.19E+04 1.87E+05 1.04E+05 1.68E+05 3.91E+04 9.88E+04

FALSE FALSE 1.77E+04 1.16E+04 1.60E+04 6.02E+04 3.22E+04 5.57E+04 1.30E+04 5.06E+04

up

up

up

FALSE 2.32E+04 1.11E+04 2.27E+04 7.68E+04 3.92E+04 1.74E+04 7.31E+04 1.44E+05

up

up

1.15E+07 1.90E+07 9.12E+06 1.39E+07 1.18E+07 1.21E+07 3.91E+07 3.39E+07

9.58E+06 1.74E+07 7.53E+06 1.20E+07 9.90E+06 9.40E+06 3.54E+07 3.26E+07

up

up

up

up

FALSE 5.37E+04 3.64E+04 5.59E+04 6.92E+04 1.24E+05 1.06E+05 1.06E+05 9.75E+04

FALSE 1.50E+04 6.21E+03 1.62E+04 2.25E+04 2.66E+04 1.85E+04 3.36E+04 3.12E+04

FALSE 4.12E+04 1.97E+04 5.43E+04 5.40E+04 7.48E+04 7.52E+04 9.67E+04 1.23E+05

FALSE 7.74E+04 3.52E+04 9.31E+04 1.65E+05 1.02E+05 1.57E+05 1.99E+05 2.31E+05

down 5.09E+06 2.39E+06 6.03E+06 9.33E+06 8.51E+06 1.35E+07 4.22E+06 4.51E+06

down 5.39E+06 2.61E+06 5.64E+06 9.84E+06 9.63E+06 1.42E+07 4.37E+06 4.91E+06

FALSE 1.01E+05 1.92E+05 1.34E+05 5.22E+04 4.78E+04 5.98E+04 2.57E+04 3.81E+04

FALSE 7.70E+04 1.59E+05 1.20E+05 5.37E+04 3.95E+04 5.32E+04 1.74E+04 3.52E+04

FALSE 6.03E+04 2.32E+04 2.21E+04 1.83E+04 1.53E+04 2.59E+04 1.50E+04 1.67E+04

down 2.45E+05 2.92E+05 2.51E+05 4.72E+05 3.03E+05 3.90E+05 3.94E+04 8.99E+04

FALSE 2.53E+05 1.40E+05 1.63E+05 9.78E+04 8.09E+04 1.31E+05 6.77E+04 8.20E+04

down 3.58E+05 4.05E+05 4.17E+05 4.92E+05 7.86E+05 4.24E+05 6.36E+03 9.00E+00

down 3.40E+06 2.98E+06 2.92E+06 4.83E+06 4.13E+06 3.78E+06 8.69E+05 2.98E+06

FALSE

FALSE

down

down

down

down

down

down

FALSE

up

up

9.00E+00 2.70E+04 9.00E+00 9.00E+00 9.00E+00 4.18E+04 3.57E+04 2.94E+05

down

FALSE 4.47E+04 2.34E+04 2.58E+04 1.99E+04 1.06E+04 2.44E+04 1.39E+04 1.32E+04

 

down

FALSE

up

down 1.38E+06 7.63E+05 1.42E+06 7.67E+05 8.54E+05 1.30E+06 3.99E+05 4.40E+05

down 1.93E+07 1.01E+07 2.14E+07 3.12E+07 2.44E+07 3.86E+07 1.51E+07 1.53E+07

up

1.14E+05 8.80E+04 8.44E+04 1.16E+05 1.05E+05 8.51E+04 1.73E+05 2.89E+05

FALSE

down 8.10E+03 6.44E+03 6.97E+03 9.97E+03 8.44E+03 1.01E+04 2.94E+03 7.71E+03

FALSE FALSE 3.20E+04 7.18E+04 4.79E+04 2.47E+04 9.76E+03 1.24E+04 2.05E+04 3.44E+04

down

down

down

down

FALSE

up

down 2.22E+05 8.99E+04 3.19E+05 7.50E+04 4.38E+04 7.14E+04 3.47E+04 2.15E+04

down 4.35E+04 7.59E+04 5.61E+04 2.72E+04 3.14E+04 2.32E+04 1.43E+04 5.34E+03

FALSE 9.74E+04 1.29E+05 1.36E+05 7.34E+04 6.65E+04 7.69E+04 3.84E+04 6.24E+04

down 2.78E+04 2.23E+04 2.78E+04 2.83E+04 4.39E+04 2.77E+04 9.00E+00 9.00E+00

down 1.89E+04 3.59E+04 1.83E+04 4.09E+04 4.61E+04 4.82E+04 1.61E+04 1.68E+04

up

FALSE 1.43E+05 3.73E+05 2.48E+05 7.16E+04 6.51E+04 1.05E+05 5.72E+04 7.53E+04

up 1.34E+04 1.13E+04 2.68E+04 3.73E+04 2.32E+04 1.92E+04 5.35E+04 6.67E+04

9.00E+00 9.00E+00 9.00E+00 9.00E+00 9.00E+00 9.00E+00 8.82E+05 6.58E+05

down

up

down

down

down

down

down

down

down

down 6.58E+04 4.48E+04 1.10E+05 1.81E+05 2.44E+05 1.32E+05 9.00E+00 9.00E+00

FALSE 1.69E+05 1.80E+05 3.49E+05 3.87E+04 4.72E+04 2.55E+04 3.01E+04 3.45E+04

FALSE 4.95E+05 6.39E+05 8.40E+05 1.50E+05 1.35E+05 1.81E+05 9.44E+04 1.16E+05

FALSE 3.56E+06 7.93E+06 7.18E+06 1.11E+06 8.64E+05 8.88E+05 2.68E+05 6.60E+05

FALSE 1.89E+06 8.07E+06 4.19E+06 5.84E+05 5.84E+05 4.95E+05 1.45E+05 4.03E+05

down 2.68E+05 7.93E+05 5.93E+05 9.19E+04 1.15E+05 1.12E+05 2.08E+04 5.42E+04

FALSE 1.60E+05 5.67E+05 3.38E+05 8.43E+04 8.64E+04 7.16E+04 2.41E+04 5.82E+04

FALSE FALSE 1.10E+05 8.37E+04 8.67E+04 1.64E+05 2.73E+05 1.78E+05 1.35E+05 1.45E+05

down

down

down 8.43E+04 4.42E+04 1.14E+05 1.05E+05 8.97E+04 8.63E+04 2.54E+04 3.98E+04

down 1.38E+06 1.05E+06 2.16E+06 8.71E+05 1.15E+06 1.29E+06 2.59E+05 2.77E+05

FALSE FALSE 1.21E+04 1.60E+04 1.07E+04 5.69E+03 5.67E+03 4.81E+03 9.47E+03 3.40E+03

down

down

FALSE

down

down

down

down

down

down 1.38E+07 9.03E+06 1.34E+07 1.63E+07 1.48E+07 1.64E+07 5.50E+06 5.56E+06

FALSE 3.23E+06 4.81E+06 6.80E+06 4.37E+05 4.40E+05 3.33E+05 1.86E+05 3.85E+05

down 3.34E+04 2.89E+04 2.37E+04 4.19E+04 3.66E+04 2.68E+04 1.13E+04 2.01E+04

down 2.95E+06 2.16E+06 2.68E+06 2.72E+06 1.77E+06 3.02E+06 2.75E+05 5.68E+05

FALSE 8.00E+05 1.15E+06 1.36E+06 3.61E+05 4.23E+05 3.34E+05 2.85E+05 4.43E+05

down 4.89E+07 1.10E+08 8.67E+07 1.94E+07 2.24E+07 1.74E+07 6.31E+06 1.13E+07

down 4.72E+07 1.04E+08 8.45E+07 1.86E+07 2.29E+07 1.73E+07 5.93E+06 1.07E+07

down 1.51E+05 1.59E+05 1.95E+05 1.67E+05 3.00E+05 1.43E+05 5.36E+04 7.87E+04

FALSE FALSE 6.15E+04 2.61E+04 5.59E+04 1.16E+05 9.43E+04 7.70E+04 7.82E+04 3.67E+04

up

FALSE 4.77E+04 3.33E+04 7.30E+04 1.26E+05 1.64E+05 1.29E+05 1.54E+05 1.45E+05

down 1.09E+07 7.21E+06 1.18E+07 1.33E+07 1.14E+07 1.39E+07 3.73E+06 4.37E+06

FALSE 8.10E+04 2.07E+05 1.44E+05 7.43E+04 6.19E+04 6.09E+04 2.84E+04 5.65E+04

down 4.58E+05 9.82E+05 7.95E+05 9.78E+04 8.26E+04 7.77E+04 2.82E+04 5.58E+04

down 6.29E+05 3.13E+06 1.38E+06 2.63E+05 2.27E+05 1.88E+05 4.35E+04 1.00E+05

down 1.08E+07 2.76E+07 2.21E+07 3.78E+06 2.89E+06 2.86E+06 7.55E+05 1.78E+06

down 1.75E+05 9.73E+04 1.71E+05 2.56E+05 2.86E+05 2.56E+05 1.58E+05 1.37E+05

down 9.03E+04 5.06E+04 8.47E+04 1.33E+05 1.10E+05 1.56E+05 5.90E+04 3.58E+04

down 2.44E+04 8.44E+03 4.46E+04 9.00E+00 8.33E+04 1.96E+04 9.00E+00 9.00E+00

FALSE 1.38E+04 1.74E+04 3.00E+04 3.22E+04 3.72E+04 2.57E+04 3.90E+04 8.28E+04

down 6.60E+04 3.15E+04 9.92E+04 1.95E+05 2.08E+05 1.27E+05 2.53E+04 7.02E+04

down

down

down

down

down

FALSE

FALSE

down

up

FALSE

FALSE FALSE 5.22E+04 2.74E+04 5.56E+04 1.22E+05 1.50E+05 1.05E+05 6.30E+04 9.24E+04

FALSE FALSE 3.56E+03 9.00E+00 3.00E+03 1.35E+04 1.56E+04 6.25E+03 9.00E+00 5.53E+03

 

up

FALSE 1.05E+04 5.25E+03 6.48E+03 1.78E+04 1.14E+04 7.04E+04 1.35E+04 3.67E+04

FALSE FALSE 8.62E+04 4.46E+04 1.77E+05 1.02E+05 5.54E+05 3.55E+05 3.14E+05 3.51E+05

FALSE FALSE 6.01E+06 1.19E+07 4.98E+06 2.99E+06 3.28E+06 3.47E+06 2.76E+06 3.16E+06

down

up

FALSE

FALSE

down

FALSE 1.03E+05 3.50E+05 2.34E+05 5.97E+04 4.70E+04 5.80E+04 1.59E+04 4.02E+04

FALSE 1.33E+03 1.83E+03 2.04E+03 4.61E+03 1.44E+04 4.11E+03 8.44E+03 6.22E+03

down 1.36E+05 5.80E+04 1.71E+05 1.31E+06 7.19E+05 7.85E+05 1.48E+04 3.35E+04

down 1.61E+05 7.70E+04 1.91E+05 1.53E+06 8.05E+05 9.13E+05 2.68E+04 4.88E+04

down 6.17E+06 4.75E+06 8.56E+06 7.09E+06 8.54E+06 7.31E+06 2.13E+06 1.86E+06

FALSE FALSE 5.12E+03 1.40E+04 1.93E+03 7.89E+03 1.34E+04 2.95E+04 3.74E+03 4.05E+03

down

down

FALSE

FALSE

up

down 1.04E+06 5.56E+05 1.58E+06 1.61E+06 1.43E+06 1.60E+06 5.29E+05 5.97E+05

down 1.42E+05 8.39E+04 1.95E+05 2.06E+05 1.91E+05 1.96E+05 7.20E+04 7.31E+04

down 5.09E+04 3.67E+04 4.12E+04 3.61E+04 4.98E+04 5.99E+04 1.36E+04 2.21E+04

down 1.67E+05 1.06E+05 1.02E+05 2.64E+05 3.61E+05 1.77E+05 8.91E+04 1.15E+05

up

6.40E+04 5.85E+04 5.60E+04 5.64E+04 3.43E+04 3.82E+04 2.39E+05 2.19E+05

FALSE

down

down

down

down

down

down

down

up

down

down

down

FALSE

up

down 4.08E+03 2.85E+03 6.57E+03 7.03E+03 7.36E+03 8.37E+03 3.53E+03 2.51E+03

FALSE 3.81E+04 4.67E+04 3.61E+04 4.38E+04 2.69E+04 2.05E+04 1.42E+04 1.81E+04

FALSE 9.52E+04 2.44E+05 1.65E+05 5.71E+04 5.67E+04 4.33E+04 3.05E+04 4.71E+04

FALSE 8.58E+04 2.41E+05 1.76E+05 4.05E+04 4.86E+04 3.46E+04 2.18E+04 3.83E+04

down 5.58E+04 4.45E+04 4.35E+04 4.38E+04 1.00E+05 8.13E+04 9.00E+00 9.00E+00

down 4.17E+05 3.01E+05 5.43E+05 6.32E+05 4.68E+05 4.81E+05 4.92E+04 1.30E+05

down 7.84E+04 2.57E+04 8.87E+04 5.87E+04 8.11E+04 3.98E+04 1.18E+04 2.40E+04

down 2.11E+05 6.38E+04 2.57E+05 1.61E+05 1.62E+05 9.79E+04 3.18E+04 5.67E+04

FALSE 8.73E+05 5.16E+05 1.49E+06 2.86E+06 2.08E+06 2.22E+06 1.28E+06 2.49E+06

down 3.55E+05 4.01E+05 4.81E+05 2.22E+05 4.72E+05 3.76E+05 2.28E+05 1.77E+05

FALSE 9.25E+04 1.20E+05 1.27E+05 6.91E+04 1.22E+05 9.51E+04 6.62E+04 6.12E+04

down 3.66E+05 9.14E+04 3.95E+05 2.43E+05 4.17E+05 1.73E+05 3.46E+04 6.38E+04

down 8.30E+03 2.87E+04 5.91E+03 1.47E+04 1.56E+04 3.40E+04 2.38E+03 5.74E+02

FALSE 7.45E+06 5.14E+06 1.01E+07 6.58E+06 2.10E+07 1.53E+07 4.37E+07 3.06E+07

FALSE 7.34E+06 5.27E+06 9.44E+06 6.22E+06 2.00E+07 1.45E+07 4.25E+07 3.05E+07

down 1.59E+05 1.66E+05 2.29E+05 2.09E+05 2.63E+05 1.42E+05 9.23E+04 4.99E+04

down 3.74E+05 3.41E+05 4.74E+05 3.36E+05 6.16E+05 4.21E+05 2.00E+05 2.07E+05

FALSE 7.62E+06 4.93E+06 9.74E+06 6.93E+06 2.06E+07 1.65E+07 4.68E+07 3.24E+07

down 8.52E+04 9.88E+04 1.11E+05 1.17E+05 2.30E+05 1.85E+05 3.59E+04 1.86E+04

down 1.10E+06 9.30E+05 1.00E+06 9.87E+05 1.02E+06 1.41E+06 1.84E+05 1.48E+05

down 5.33E+03 2.34E+03 4.96E+03 7.97E+03 1.81E+04 4.64E+03 4.34E+03 3.34E+03

down 6.53E+04 4.08E+04 6.93E+04 4.20E+04 5.41E+04 8.79E+04 2.36E+04 4.55E+04

down 2.08E+04 1.32E+04 1.89E+04 2.90E+04 2.81E+04 4.00E+04 1.12E+04 2.27E+04

up

down

down

up

down

down

FALSE

down

FALSE

up

up

1.06E+03 1.06E+03 1.65E+03 2.09E+03 1.21E+03 2.49E+03 7.65E+03 1.08E+04

down

FALSE 1.00E+05 2.53E+05 2.16E+05 5.42E+04 5.26E+04 5.86E+04 3.21E+04 3.90E+04

FALSE FALSE 6.71E+05 6.18E+05 1.49E+06 1.01E+06 5.36E+06 4.35E+06 1.92E+06 2.40E+06

FALSE FALSE 6.38E+05 6.97E+05 1.42E+06 9.18E+05 5.09E+06 4.89E+06 1.97E+06 2.70E+06

down

down

down

down

FALSE

FALSE 3.58E+06 3.79E+06 3.52E+06 1.99E+06 2.87E+06 2.56E+06 2.04E+06 1.20E+06

FALSE 5.82E+06 6.58E+06 5.51E+06 3.25E+06 4.74E+06 3.92E+06 3.27E+06 1.81E+06

FALSE 2.87E+06 3.13E+06 2.85E+06 1.67E+06 2.24E+06 1.92E+06 1.58E+06 8.90E+05

FALSE 3.53E+06 4.03E+06 3.43E+06 2.07E+06 2.89E+06 2.24E+06 1.89E+06 1.05E+06

down 1.91E+07 3.12E+06 1.68E+07 1.63E+07 7.14E+06 1.59E+07 2.90E+06 3.91E+06

 

up

down

down

up

up

9.00E+00 9.00E+00 9.00E+00 9.00E+00 9.00E+00 9.00E+00 4.73E+04 1.12E+05

down 1.09E+05 4.10E+05 2.55E+05 3.50E+04 3.50E+04 1.32E+04 2.49E+03 2.25E+03

down 3.92E+04 2.87E+04 5.02E+04 6.51E+04 9.14E+04 5.00E+04 9.42E+03 1.41E+04

up

2.88E+06 1.99E+06 3.46E+06 1.69E+06 2.00E+06 1.45E+06 9.85E+06 6.36E+06

down

down

down

FALSE

FALSE

FALSE

FALSE

up

down 2.79E+04 3.53E+04 4.30E+04 3.24E+04 7.23E+04 5.43E+04 9.00E+00 9.00E+00

down 2.60E+04 2.80E+04 2.97E+04 3.18E+04 6.99E+04 4.98E+04 2.17E+04 9.85E+03

down 2.58E+04 2.85E+04 4.42E+04 3.28E+04 8.22E+04 3.86E+04 9.00E+00 9.00E+00

down 3.46E+06 2.87E+06 4.61E+06 4.81E+06 6.79E+06 5.54E+06 1.89E+06 2.31E+06

down 3.36E+06 2.87E+06 4.96E+06 4.53E+06 6.51E+06 6.59E+06 2.06E+06 2.26E+06

down 4.30E+04 1.94E+04 3.42E+04 4.38E+04 4.56E+04 3.41E+04 1.58E+04 2.58E+04

down 1.07E+06 5.88E+05 1.54E+06 3.07E+06 1.45E+06 2.37E+06 4.06E+05 5.35E+05

FALSE 3.48E+04 3.40E+04 4.37E+04 6.13E+04 7.30E+04 6.44E+04 8.72E+04 9.94E+04

down 1.01E+04 1.51E+04 8.83E+03 1.60E+04 1.70E+04 2.28E+04 8.85E+03 9.54E+03

FALSE

up

up

2.72E+04 1.70E+04 2.30E+04 3.44E+04 3.21E+04 2.43E+04 1.03E+05 1.66E+05

down

down

down

down

down

down

FALSE

down

FALSE

down

down

up

down 2.39E+04 2.17E+04 1.30E+04 4.18E+04 3.31E+04 3.11E+04 9.00E+00 9.00E+00

FALSE 2.16E+04 2.12E+04 1.42E+04 1.43E+04 1.65E+04 1.11E+04 1.02E+04 6.97E+03

down 7.19E+05 6.52E+05 7.91E+05 7.41E+05 1.06E+06 9.52E+05 4.04E+05 2.49E+05

down 1.94E+04 1.86E+04 3.98E+04 1.15E+04 2.20E+04 3.25E+04 9.00E+00 9.00E+00

down 5.71E+05 5.58E+05 6.41E+05 5.47E+05 8.72E+05 6.47E+05 3.48E+05 2.60E+05

FALSE 1.14E+06 4.70E+06 2.00E+06 3.54E+05 5.75E+05 4.89E+05 2.55E+05 2.92E+05

down 7.23E+05 6.48E+05 7.55E+05 8.35E+05 1.07E+06 1.02E+06 4.53E+05 3.46E+05

down 2.60E+04 1.46E+04 2.19E+04 3.65E+04 3.71E+04 1.62E+04 9.00E+00 9.00E+00

down 4.00E+03 1.27E+03 4.71E+03 2.92E+04 1.09E+04 1.33E+04 1.04E+04 6.24E+03

down 3.14E+06 2.60E+06 3.73E+06 3.90E+06 4.43E+06 4.46E+06 2.58E+05 2.04E+05

down 3.27E+04 2.76E+04 5.56E+04 2.90E+04 7.59E+04 3.18E+04 1.70E+04 1.70E+04

up

down 4.87E+05 1.33E+06 7.43E+05 5.73E+05 3.90E+05 5.14E+05 1.57E+05 2.19E+05

up 1.62E+04 9.26E+03 2.35E+04 2.59E+04 2.45E+04 2.77E+04 8.54E+04 1.07E+05

FALSE 1.93E+05 2.89E+05 3.73E+05 2.07E+05 2.30E+05 2.04E+05 2.23E+05 9.98E+04

up 9.00E+00 9.00E+00 9.00E+00 9.00E+00 9.00E+00 9.00E+00 7.26E+04 9.46E+04

9.00E+00 9.00E+00 9.00E+00 9.00E+00 9.00E+00 9.00E+00 1.33E+04 1.45E+04

down

up

down

up

down

down

down

up

down 4.12E+04 1.90E+05 5.16E+04 3.89E+04 4.90E+04 5.74E+04 1.32E+04 9.17E+03

FALSE 7.48E+03 1.69E+04 1.06E+04 8.29E+03 2.24E+03 7.43E+03 9.16E+02 3.39E+03

FALSE 1.41E+04 2.45E+04 2.41E+04 1.47E+04 9.98E+03 1.14E+04 7.15E+03 9.34E+03

up

1.15E+04 1.17E+04 1.39E+04 6.29E+03 7.29E+03 7.77E+03 1.50E+05 1.63E+05

down

down

FALSE

down

down

up

down 6.92E+03 5.21E+03 4.56E+03 8.81E+03 2.95E+04 1.54E+04 3.57E+02 1.31E+03

down 4.18E+04 1.73E+05 6.34E+04 2.49E+04 5.78E+04 9.57E+04 8.80E+03 1.10E+04

down 2.10E+06 1.45E+06 2.20E+06 5.02E+06 2.36E+06 3.29E+06 1.28E+06 1.45E+06

down 1.52E+06 1.43E+06 2.23E+06 1.35E+06 1.04E+06 7.87E+05 2.35E+05 4.54E+05

FALSE 1.96E+07 1.69E+07 2.31E+07 1.47E+07 1.02E+07 1.03E+07 9.65E+06 1.23E+07

up

up

2.99E+04 2.81E+04 3.32E+04 1.14E+04 6.02E+04 2.54E+04 1.22E+05 2.65E+05

9.00E+00 9.00E+00 9.00E+00 9.00E+00 9.00E+00 9.00E+00 1.12E+05 8.50E+04

up

down

down 2.88E+04 1.97E+04 2.79E+04 5.84E+04 3.03E+04 3.53E+04 2.42E+03 2.78E+03

FALSE FALSE 2.11E+04 1.29E+04 2.48E+04 3.06E+03 1.65E+04 7.35E+03 1.42E+04 1.29E+04

down

down

down

down 4.12E+06 4.52E+06 3.87E+06 5.22E+06 9.31E+06 7.23E+06 9.47E+05 9.59E+05

down 4.50E+03 4.31E+03 6.93E+03 1.65E+04 1.34E+04 7.05E+03 9.00E+00 9.00E+00

down 5.00E+03 4.34E+03 6.03E+03 1.42E+04 1.79E+04 7.74E+03 9.00E+00 9.00E+00

 

down

up

FALSE

down

up

down 9.01E+04 8.14E+04 9.04E+04 9.72E+04 1.37E+05 1.15E+05 3.66E+04 1.97E+04

up

up

6.17E+04 2.18E+04 4.88E+04 1.27E+05 2.99E+04 9.07E+04 2.66E+05 2.27E+05

2.52E+05 2.33E+05 3.50E+05 1.35E+05 2.60E+05 2.17E+05 5.48E+05 5.42E+05

down 1.27E+06 5.60E+05 1.62E+06 3.74E+06 1.38E+06 1.53E+06 9.00E+00 9.00E+00

up 4.14E+04 5.02E+04 6.47E+04 2.40E+04 1.02E+05 4.37E+04 1.71E+05 2.29E+05

down

up

down 9.84E+05 7.70E+05 1.05E+06 5.34E+05 1.48E+06 1.13E+06 5.16E+05 4.87E+05

FALSE 2.90E+03 1.95E+03 3.11E+03 3.13E+03 2.17E+03 9.65E+03 6.76E+03 1.23E+04

up

up

up

up

6.55E+04 5.93E+04 8.36E+04 4.73E+04 1.25E+05 8.78E+04 6.24E+05 3.68E+05

7.50E+04 4.29E+04 8.84E+04 5.08E+04 1.65E+05 6.58E+04 3.90E+05 4.71E+05

FALSE

down

up

down 5.32E+03 1.84E+03 5.40E+03 1.69E+04 1.07E+04 1.18E+04 1.78E+03 2.29E+03

down 3.63E+04 3.74E+04 5.27E+04 3.39E+04 5.50E+04 3.80E+04 9.00E+00 9.00E+00

up

5.41E+05 4.55E+05 6.24E+05 3.54E+05 6.24E+05 3.98E+05 2.41E+06 2.23E+06

down

down

down

FALSE

up

down 6.24E+04 2.67E+04 8.42E+04 4.80E+04 3.38E+04 3.57E+04 9.06E+03 1.81E+04

down 6.84E+04 3.20E+04 9.66E+04 5.08E+04 4.11E+04 3.84E+04 1.02E+04 1.52E+04

down 6.21E+04 1.01E+05 6.32E+04 8.38E+04 7.97E+04 7.66E+04 3.01E+04 3.38E+04

down 1.15E+05 1.10E+05 1.80E+05 3.71E+05 3.18E+05 3.31E+05 2.54E+05 1.56E+05

up

9.00E+00 9.00E+00 9.00E+00 9.00E+00 9.00E+00 1.33E+04 7.22E+04 7.63E+04

down

down

up

down 5.62E+05 4.77E+05 5.02E+05 4.92E+05 9.85E+05 5.91E+05 1.26E+04 5.63E+03

down 7.05E+05 7.51E+05 8.91E+05 6.47E+05 1.50E+06 6.43E+05 1.39E+04 2.37E+03

up

1.04E+04 1.31E+04 1.36E+04 9.00E+00 9.00E+00 9.00E+00 4.95E+04 3.68E+04

FALSE FALSE 1.32E+05 4.54E+05 1.41E+05 2.79E+05 4.18E+05 8.12E+05 1.73E+05 9.50E+04

down

down

down

down

up

down 6.80E+04 4.98E+04 1.11E+05 1.02E+05 1.46E+05 1.20E+05 9.00E+00 9.00E+00

down 5.88E+05 2.77E+05 7.43E+05 3.59E+05 3.66E+05 2.54E+05 8.94E+04 1.91E+05

down 6.51E+05 3.08E+05 7.49E+05 3.73E+05 3.57E+05 2.80E+05 9.64E+04 1.87E+05

down 2.25E+05 1.60E+05 2.81E+05 4.49E+05 4.00E+05 4.75E+05 2.97E+04 4.01E+03

FALSE 1.59E+04 9.59E+03 9.01E+03 8.89E+03 8.25E+03 1.78E+04 2.62E+04 1.10E+05

up

up

9.00E+00 9.00E+00 9.00E+00 9.00E+00 9.00E+00 2.91E+04 4.15E+04 2.30E+05

FALSE FALSE 1.08E+05 6.80E+04 9.45E+04 3.09E+05 1.54E+05 3.52E+05 7.34E+05 1.83E+05

FALSE FALSE 7.62E+03 3.10E+04 8.34E+03 1.66E+04 3.17E+04 5.03E+04 1.87E+04 1.52E+04

down

up

up

up

down

up

down 4.05E+05 7.48E+04 3.32E+05 1.35E+06 8.00E+05 3.98E+05 9.00E+00 9.00E+00

FALSE 5.73E+04 7.73E+04 3.96E+04 1.05E+05 9.00E+04 2.52E+05 1.24E+05 7.98E+04

FALSE 1.88E+04 5.12E+04 3.85E+04 9.19E+04 7.09E+04 2.05E+05 5.98E+04 7.81E+04

FALSE 3.16E+04 6.91E+04 3.02E+04 8.50E+04 6.91E+04 1.94E+05 9.24E+04 8.12E+04

down 2.91E+05 1.92E+05 2.20E+05 1.76E+05 2.34E+05 2.87E+05 1.91E+04 3.37E+04

up

9.00E+00 9.00E+00 9.00E+00 2.06E+04 1.65E+04 2.67E+04 6.46E+04 1.34E+05

down

down

down

down

down

up

down 3.56E+04 2.89E+04 3.79E+04 2.19E+04 4.59E+04 3.91E+04 9.00E+00 9.00E+00

down 2.46E+05 1.90E+05 2.39E+05 1.67E+05 2.59E+05 2.77E+05 1.91E+04 2.95E+04

FALSE 1.25E+07 7.07E+06 1.39E+07 7.12E+06 7.10E+06 5.92E+06 2.19E+06 3.88E+06

FALSE 1.75E+06 1.00E+06 1.98E+06 9.91E+05 5.92E+05 5.74E+05 2.43E+05 4.09E+05

down 5.52E+05 1.03E+06 5.65E+05 3.71E+05 5.00E+05 3.34E+05 2.43E+05 1.32E+05

up

up

6.07E+04 6.96E+04 3.05E+04 3.14E+04 3.47E+04 5.04E+04 4.82E+05 1.25E+06

7.95E+04 7.77E+04 6.33E+04 3.11E+04 5.55E+04 7.10E+04 6.12E+05 1.65E+06

up

down

down

up

down 2.92E+04 2.92E+04 2.82E+04 4.75E+04 5.22E+04 2.79E+04 9.00E+00 9.00E+00

down 3.31E+04 2.47E+04 3.41E+04 4.04E+04 4.99E+04 3.04E+04 9.00E+00 9.00E+00

up

9.00E+00 9.00E+00 9.00E+00 9.00E+00 9.00E+00 9.00E+00 1.51E+05 1.32E+05

down

down 1.88E+04 2.99E+04 5.50E+04 3.95E+04 3.40E+04 2.22E+04 9.00E+00 9.00E+00

 

FALSE FALSE 3.43E+04 1.79E+05 4.57E+04 1.05E+05 1.36E+05 1.50E+06 6.75E+04 2.81E+04

FALSE FALSE 2.11E+04 1.18E+05 4.21E+04 8.84E+04 7.47E+04 9.74E+05 6.98E+04 4.75E+04

FALSE FALSE 2.10E+04 1.12E+05 1.99E+04 7.56E+04 1.08E+05 1.02E+06 4.12E+04 3.99E+04

down

up

FALSE 4.55E+04 3.84E+04 5.04E+04 3.29E+04 2.19E+04 1.29E+04 1.28E+04 3.19E+04

up 8.87E+03 6.74E+03 1.41E+04 1.69E+04 2.64E+04 2.76E+04 2.09E+05 1.82E+05

down

FALSE

down 2.16E+05 1.37E+05 2.64E+05 1.62E+05 1.46E+05 1.74E+05 5.23E+04 7.26E+04

down 1.24E+05 3.29E+04 3.42E+04 2.77E+05 5.02E+05 5.19E+06 1.38E+05 1.88E+04

FALSE FALSE 8.82E+03 2.66E+03 2.44E+03 4.94E+03 1.15E+04 1.49E+04 2.91E+04 5.07E+03

FALSE

FALSE

FALSE

down

up

FALSE

up

FALSE

down

FALSE

up

down 4.51E+04 9.39E+03 1.46E+04 4.97E+05 3.04E+05 6.75E+05 2.45E+04 9.01E+04

down 1.79E+05 8.63E+04 8.45E+04 9.02E+05 7.62E+05 2.95E+06 4.84E+04 1.23E+05

down 1.89E+05 1.03E+05 7.82E+04 1.09E+06 7.42E+05 2.72E+06 8.68E+04 1.47E+05

FALSE 1.37E+05 2.10E+05 7.35E+04 1.31E+05 1.06E+05 7.50E+04 7.11E+04 4.47E+04

down 2.62E+04 2.11E+04 1.52E+04 4.96E+05 3.42E+05 6.62E+05 4.85E+04 6.70E+04

down 2.13E+05 9.57E+04 8.14E+04 9.46E+05 7.38E+05 2.70E+06 7.30E+04 1.09E+05

down 5.09E+04 2.56E+04 1.59E+04 7.18E+05 3.99E+05 8.73E+05 4.43E+04 6.47E+04

down 1.78E+05 7.81E+04 5.84E+04 9.49E+05 7.08E+05 2.71E+06 6.01E+04 1.20E+05

down 1.52E+05 2.30E+05 8.60E+04 1.55E+05 8.75E+04 2.61E+05 8.94E+04 4.31E+04

down 2.38E+06 1.78E+06 2.51E+06 1.90E+06 2.74E+06 2.10E+06 8.18E+05 1.11E+06

up

up

7.60E+04 1.05E+05 9.45E+04 6.61E+04 1.47E+05 6.39E+04 6.81E+05 7.54E+05

2.82E+04 1.83E+04 1.24E+04 1.81E+04 2.61E+04 3.19E+04 1.04E+05 1.22E+05

up

down

FALSE

FALSE

up

up

FALSE

up

up

up

down

down

down

FALSE 4.26E+06 2.92E+06 5.04E+06 3.80E+06 1.99E+06 2.06E+06 1.15E+06 1.98E+06

down 2.63E+04 1.57E+04 1.64E+04 4.70E+04 2.45E+04 4.43E+04 9.00E+00 2.69E+04

down 3.67E+04 1.92E+04 2.28E+04 2.41E+04 9.63E+04 7.89E+04 1.60E+04 1.40E+04

FALSE 9.00E+00 9.00E+00 9.00E+00 9.00E+00 9.00E+00 3.97E+05 4.86E+04 3.47E+04

FALSE 9.00E+00 9.00E+00 9.00E+00 1.45E+04 2.71E+04 4.43E+05 4.44E+04 2.23E+04

down 1.59E+04 2.61E+04 1.27E+04 2.74E+04 3.45E+04 4.47E+04 9.00E+00 3.30E+04

FALSE 9.00E+00 9.00E+00 9.00E+00 9.00E+00 9.00E+00 3.92E+05 3.82E+04 2.54E+04

FALSE 9.00E+00 9.00E+00 9.00E+00 9.00E+00 2.25E+04 3.94E+05 4.27E+04 3.19E+04

FALSE 2.61E+04 1.81E+04 1.35E+04 1.73E+04 3.40E+04 3.23E+05 3.19E+04 3.19E+04

FALSE 4.20E+05 1.96E+05 4.44E+05 2.25E+05 2.15E+05 1.61E+05 1.09E+05 1.62E+05

FALSE 5.18E+05 3.30E+05 5.90E+05 3.35E+05 2.77E+05 2.12E+05 1.29E+05 2.45E+05

FALSE 1.22E+05 1.24E+05 2.05E+05 6.70E+04 6.32E+04 6.16E+04 3.81E+04 6.89E+04

FALSE FALSE 1.29E+04 5.79E+04 2.36E+04 3.77E+04 4.92E+04 1.60E+05 2.71E+04 3.05E+04

down

down

FALSE 2.17E+06 1.10E+06 2.29E+06 1.06E+06 1.15E+06 8.73E+05 3.46E+05 6.54E+05

FALSE 3.46E+05 2.14E+05 4.59E+05 1.88E+05 1.18E+05 1.31E+05 6.20E+04 1.01E+05

FALSE FALSE 9.89E+03 5.46E+03 5.98E+03 5.92E+03 2.07E+04 3.37E+04 4.58E+04 3.14E+03

FALSE

down

down

down

FALSE

down

down 2.64E+03 2.11E+02 6.91E+02 1.27E+04 4.50E+03 2.64E+04 4.28E+02 1.19E+02

FALSE 2.35E+04 3.75E+04 3.53E+04 9.07E+03 1.30E+04 9.23E+03 3.86E+03 5.61E+03

down 1.33E+05 1.02E+05 1.58E+05 7.12E+04 1.12E+05 6.27E+04 1.90E+04 1.31E+04

down 3.39E+05 1.32E+05 4.35E+05 2.28E+05 1.74E+05 1.36E+05 5.01E+04 7.57E+04

down 1.37E+05 8.98E+04 2.00E+05 1.37E+05 1.86E+05 1.62E+05 6.34E+04 8.59E+04

down 3.27E+04 3.76E+04 2.88E+04 2.91E+04 5.14E+04 3.19E+04 9.00E+00 9.00E+00

FALSE FALSE 1.76E+04 2.03E+04 1.39E+04 2.37E+04 6.55E+04 3.02E+04 2.44E+04 2.68E+04

down

down

down

down 2.22E+04 3.88E+04 2.71E+04 1.76E+04 5.73E+04 2.39E+04 9.00E+00 9.00E+00

down 3.50E+04 5.70E+04 1.02E+05 4.66E+04 1.09E+05 4.18E+04 2.66E+04 1.18E+04

FALSE 4.90E+07 2.83E+07 4.53E+07 2.34E+07 2.37E+07 2.15E+07 8.27E+06 1.48E+07

 

down

down

up

down

up

down

down

down

down

down

down

down

down

down

down

up

FALSE 3.49E+06 2.19E+06 3.54E+06 1.62E+06 1.00E+06 1.18E+06 4.88E+05 1.09E+06

down 2.34E+05 1.78E+05 2.52E+05 3.14E+05 3.42E+05 2.59E+05 1.14E+04 3.73E+04

FALSE 7.50E+05 7.71E+05 5.25E+05 1.62E+06 1.66E+06 1.13E+06 2.90E+06 1.95E+06

down 2.88E+04 1.16E+05 5.55E+04 1.34E+04 2.42E+04 2.10E+04 6.68E+03 1.10E+04

FALSE 1.06E+06 1.13E+06 1.02E+06 1.91E+06 1.62E+06 1.66E+06 3.53E+06 2.03E+06

FALSE 1.10E+05 8.09E+04 1.29E+05 6.08E+04 6.60E+04 3.42E+04 3.22E+04 3.41E+04

FALSE 1.16E+05 8.71E+04 1.31E+05 5.44E+04 7.35E+04 3.35E+04 4.00E+04 5.48E+04

FALSE 1.31E+05 6.38E+04 1.17E+05 5.97E+04 5.21E+04 4.91E+04 3.19E+04 6.43E+04

FALSE 8.47E+04 4.93E+04 9.98E+04 4.30E+04 3.58E+04 2.20E+04 2.23E+04 3.05E+04

FALSE 1.74E+05 1.32E+05 2.13E+05 1.23E+05 5.16E+04 5.72E+04 6.80E+04 9.01E+04

FALSE 9.48E+03 1.94E+04 1.32E+04 7.00E+03 1.06E+04 1.02E+04 3.12E+03 5.61E+03

down 9.07E+05 7.26E+05 8.41E+05 7.66E+05 6.51E+05 5.63E+05 2.03E+05 3.38E+05

FALSE 8.57E+05 6.89E+05 8.68E+05 7.39E+05 5.72E+05 4.73E+05 2.04E+05 3.21E+05

FALSE 3.08E+07 2.47E+07 2.82E+07 1.80E+07 1.38E+07 1.36E+07 5.67E+06 1.08E+07

FALSE 1.75E+07 1.20E+07 2.51E+07 1.32E+07 1.13E+07 1.34E+07 7.30E+06 9.95E+06

FALSE 9.00E+00 9.00E+00 9.00E+00 9.00E+00 9.00E+00 1.64E+04 6.09E+03 7.19E+03

up

up

8.81E+04 4.53E+04 8.84E+04 4.62E+04 6.26E+04 4.44E+04 2.89E+05 3.43E+05

down

FALSE

down

down

down

down

up

down 5.92E+05 5.98E+05 1.01E+06 1.30E+06 1.32E+06 1.16E+06 9.00E+00 9.00E+00

down 8.74E+04 7.89E+04 8.85E+04 1.76E+05 5.00E+05 1.24E+05 2.57E+04 3.35E+04

down 6.36E+05 6.34E+05 9.80E+05 1.38E+06 1.25E+06 1.10E+06 9.00E+00 9.00E+00

FALSE 3.12E+06 2.30E+06 3.60E+06 1.65E+06 1.20E+06 1.65E+06 5.78E+05 9.30E+05

down 6.63E+05 6.69E+05 1.13E+06 1.65E+06 2.43E+06 1.06E+06 1.41E+05 3.60E+04

down 4.58E+05 3.73E+05 5.76E+05 3.76E+05 2.07E+06 6.56E+05 2.59E+05 1.12E+05

FALSE 9.98E+04 6.87E+04 1.04E+05 8.87E+04 2.64E+05 1.20E+05 3.56E+05 3.08E+05

FALSE 1.01E+05 8.72E+04 8.47E+04 8.21E+04 2.96E+05 1.01E+05 4.02E+05 3.25E+05

down 4.95E+05 4.74E+05 6.37E+05 6.19E+05 3.02E+06 9.52E+05 2.67E+05 1.40E+05

FALSE 1.34E+06 6.41E+05 1.38E+06 7.58E+05 6.13E+05 6.32E+05 2.21E+05 3.80E+05

FALSE 1.26E+05 6.33E+04 1.25E+05 6.89E+04 3.00E+04 3.35E+04 1.59E+04 3.20E+04

down 1.45E+05 3.97E+05 4.80E+05 2.10E+05 2.21E+05 1.18E+05 5.33E+04 5.80E+04

down 1.59E+04 1.42E+04 2.36E+04 1.24E+04 1.67E+04 6.42E+03 9.00E+00 9.00E+00

down 1.08E+04 9.71E+03 2.43E+04 1.59E+04 1.13E+04 8.02E+03 9.00E+00 9.00E+00

down 2.63E+05 2.49E+05 4.45E+05 1.84E+05 1.20E+06 5.95E+05 4.23E+04 2.85E+04

down 1.37E+05 1.94E+05 1.48E+05 1.80E+05 3.94E+05 2.15E+05 1.44E+05 1.11E+05

up

down

down

down

down

down

down

down

FALSE

up

up

9.00E+00 9.00E+00 9.00E+00 9.00E+00 9.00E+00 9.00E+00 4.36E+04 4.50E+04

up

down

FALSE 3.83E+04 5.59E+04 5.31E+04 5.17E+04 1.29E+05 6.69E+04 1.14E+05 1.40E+05

down 2.91E+05 2.99E+05 3.04E+05 3.40E+05 5.17E+05 3.72E+05 6.79E+04 5.91E+04

FALSE FALSE 3.93E+04 2.57E+04 5.68E+04 2.11E+04 1.49E+04 1.43E+04 1.59E+04 2.94E+04

down

down

down

up

FALSE 5.31E+05 3.85E+05 5.30E+05 2.80E+05 2.91E+05 2.08E+05 1.45E+05 2.49E+05

FALSE 4.88E+04 5.26E+04 6.78E+04 2.16E+04 2.71E+04 2.18E+04 9.09E+03 1.33E+04

down 1.03E+06 9.43E+05 1.46E+06 1.41E+06 1.47E+06 1.25E+06 7.67E+04 7.46E+04

up

2.15E+04 1.65E+04 2.21E+04 1.91E+04 2.42E+04 1.37E+04 6.77E+04 1.41E+05

up

up

down

up

up

FALSE 1.23E+04 1.09E+04 7.34E+03 1.34E+04 1.35E+04 1.10E+05 3.51E+04 3.84E+04

FALSE 3.10E+03 9.21E+03 1.10E+04 1.09E+04 9.19E+03 9.69E+04 5.18E+04 2.95E+04

down 1.21E+05 1.61E+05 2.90E+05 1.72E+05 2.54E+05 1.88E+05 4.43E+04 4.73E+04

up

up

1.08E+05 3.12E+04 3.90E+05 2.07E+05 1.90E+04 1.11E+05 1.18E+06 1.44E+06

2.81E+03 3.27E+03 2.76E+03 5.46E+03 8.42E+03 3.77E+03 2.50E+04 1.75E+04

 

FALSE FALSE 1.35E+04 2.15E+04 5.49E+03 2.02E+04 6.37E+04 2.44E+04 3.06E+04 2.94E+04

down

down

down

down

up

down 2.86E+06 8.37E+05 4.76E+06 8.70E+06 6.61E+06 3.69E+06 5.37E+04 1.42E+05

down 2.81E+06 8.48E+05 3.75E+06 8.11E+06 6.14E+06 3.94E+06 3.46E+04 1.23E+05

down 2.65E+06 8.71E+05 4.79E+06 8.29E+06 6.38E+06 3.84E+06 5.56E+04 1.47E+05

down 1.05E+05 2.21E+04 1.49E+05 2.66E+05 1.84E+05 1.15E+05 9.00E+00 9.00E+00

up

3.34E+04 2.93E+04 4.90E+04 4.48E+04 5.42E+04 8.18E+04 1.18E+05 2.01E+05

down

up

down

down

down

down

down 2.65E+06 6.68E+05 4.21E+06 7.32E+06 5.46E+06 3.45E+06 3.82E+04 1.21E+05

FALSE 1.80E+04 2.16E+04 1.74E+04 2.94E+04 4.30E+04 3.17E+04 3.77E+04 5.14E+04

down 8.18E+04 2.30E+04 1.10E+05 2.25E+05 1.56E+05 1.01E+05 9.00E+00 9.00E+00

down 7.30E+04 2.70E+04 1.14E+05 1.97E+05 1.57E+05 1.13E+05 9.00E+00 9.00E+00

down 6.13E+04 5.09E+04 7.42E+04 8.59E+04 1.28E+05 9.04E+04 3.05E+03 2.95E+03

down 2.74E+04 2.76E+04 1.87E+04 4.62E+04 2.13E+04 2.59E+04 9.00E+00 9.00E+00

FALSE FALSE 9.00E+00 9.00E+00 9.00E+00 1.12E+04 2.44E+05 1.40E+04 9.00E+00 9.00E+00

down

down

down

down

down

FALSE

down

down

down

down

down

down

down 5.51E+05 5.65E+05 5.07E+05 1.18E+06 1.16E+06 7.44E+06 3.38E+04 2.94E+04

down 1.35E+04 2.82E+04 1.60E+04 3.51E+04 3.50E+04 1.74E+05 9.00E+00 9.00E+00

down 5.40E+05 6.03E+05 4.71E+05 1.07E+06 1.22E+06 6.85E+06 2.26E+04 2.46E+04

down 5.55E+05 6.35E+05 4.37E+05 1.01E+06 1.08E+06 5.98E+06 2.85E+04 3.14E+04

down 2.76E+04 1.55E+04 2.10E+04 2.84E+04 4.45E+04 1.91E+05 9.00E+00 9.00E+00

down 5.04E+04 5.62E+04 6.81E+04 6.10E+04 6.71E+04 8.76E+04 2.87E+04 3.58E+04

down 2.94E+05 5.24E+05 2.58E+05 3.13E+05 5.55E+05 7.64E+05 2.37E+04 2.88E+04

down 3.35E+05 5.15E+05 2.78E+05 3.28E+05 5.38E+05 7.06E+05 3.17E+04 4.47E+04

down 3.50E+05 5.25E+05 2.61E+05 2.53E+05 5.34E+05 7.08E+05 2.61E+04 4.22E+04

down 4.92E+04 2.81E+04 4.49E+04 3.06E+04 4.30E+04 3.19E+04 9.00E+00 9.00E+00

down 6.38E+05 8.13E+05 4.88E+05 6.29E+05 4.39E+05 7.23E+05 9.00E+00 9.00E+00

down 1.70E+06 2.26E+06 2.15E+06 2.10E+06 1.76E+06 1.84E+06 9.00E+00 9.00E+00

FALSE FALSE 3.53E+04 2.09E+04 1.09E+04 9.00E+00 9.00E+00 9.00E+00 2.71E+04 9.00E+00

FALSE FALSE 1.47E+05 2.84E+05 1.69E+05 2.57E+05 6.23E+05 3.36E+05 5.30E+05 4.23E+05

down

down

down

down

FALSE

FALSE

down

down

down

down

down

up

down

up

down

up

down

up

down 1.36E+04 4.02E+04 1.12E+04 1.35E+04 2.04E+04 1.33E+04 3.89E+03 6.24E+03

down 3.19E+04 1.70E+05 4.64E+04 2.77E+04 3.02E+04 4.95E+04 7.74E+03 1.17E+04

down 1.23E+04 5.75E+04 1.55E+04 1.98E+04 1.95E+04 1.41E+04 4.60E+03 5.78E+03

down 1.80E+04 2.01E+04 3.77E+04 1.52E+04 3.05E+04 3.53E+04 9.00E+00 9.00E+00

up

up

1.44E+05 5.74E+04 7.61E+04 6.03E+04 5.47E+04 7.05E+04 1.63E+05 1.47E+05

1.21E+05 8.37E+04 1.03E+05 6.99E+04 5.65E+04 6.75E+04 1.72E+05 1.49E+05

FALSE 2.63E+04 5.28E+04 3.73E+04 2.83E+04 9.00E+00 9.00E+00 9.00E+00 9.00E+00

down 2.48E+05 2.28E+05 3.77E+05 4.66E+05 6.25E+05 5.27E+05 7.26E+04 1.88E+05

down 6.84E+05 7.60E+05 1.11E+06 1.31E+06 1.89E+06 1.66E+06 2.38E+05 5.37E+05

down 1.05E+05 1.19E+05 1.56E+05 9.79E+04 2.13E+05 1.21E+05 9.00E+00 9.00E+00

down 2.15E+05 5.24E+05 4.92E+05 2.63E+05 4.53E+05 3.47E+05 1.13E+05 1.80E+05

FALSE 1.76E+05 1.25E+05 2.37E+05 2.18E+05 2.51E+05 2.68E+05 3.17E+05 4.95E+05

down 9.92E+06 6.99E+06 1.07E+07 1.05E+07 1.87E+07 1.05E+07 5.80E+05 8.61E+05

FALSE 2.55E+04 2.22E+04 2.57E+04 1.47E+04 4.07E+04 3.93E+04 7.80E+04 5.69E+04

down 1.85E+05 9.66E+04 2.53E+05 5.85E+05 3.77E+05 4.97E+05 9.00E+00 9.00E+00

FALSE 3.40E+05 1.03E+05 2.40E+05 4.60E+05 4.77E+05 3.56E+05 5.35E+05 8.08E+05

down 2.99E+04 2.76E+04 3.07E+04 1.62E+04 6.93E+04 5.29E+04 9.00E+00 9.00E+00

up

3.14E+04 2.73E+04 2.36E+04 5.18E+04 3.80E+04 4.48E+04 2.01E+05 1.14E+05

FALSE

down 5.77E+05 6.43E+05 5.11E+05 7.02E+05 5.76E+05 5.11E+05 2.08E+05 3.32E+05

 

down

down

down

down

up

down 1.44E+07 1.20E+07 1.98E+07 1.14E+07 1.36E+07 1.00E+07 1.71E+05 1.63E+05

down 2.91E+04 2.47E+04 4.55E+04 4.76E+04 5.20E+04 5.30E+04 8.48E+03 2.46E+04

down 1.30E+06 1.51E+06 1.29E+06 1.19E+06 1.11E+06 1.75E+06 2.13E+05 2.35E+05

down 9.82E+05 9.03E+05 9.45E+05 1.20E+06 1.04E+06 1.09E+06 1.26E+05 1.40E+05

up

1.03E+05 7.07E+04 1.02E+05 1.42E+05 1.06E+05 1.24E+05 1.83E+05 3.05E+05

down

down

down

down

down

down

down

up

down 2.87E+07 2.31E+07 3.20E+07 2.58E+07 3.09E+07 2.13E+07 1.72E+05 1.45E+05

down 2.04E+05 1.73E+05 1.98E+05 1.34E+05 1.24E+05 8.22E+04 9.00E+00 9.00E+00

down 4.31E+05 2.95E+05 3.75E+05 1.82E+05 2.40E+05 1.35E+05 9.00E+00 9.00E+00

down 1.68E+06 1.07E+06 2.03E+06 1.23E+06 1.33E+06 1.07E+06 9.00E+00 9.00E+00

down 7.06E+04 9.70E+04 7.67E+04 8.66E+04 5.06E+04 9.99E+04 9.00E+00 9.00E+00

down 6.81E+06 7.03E+06 9.61E+06 8.94E+06 8.28E+06 7.58E+06 9.00E+00 9.00E+00

FALSE 1.34E+05 1.59E+05 1.32E+05 2.68E+05 8.19E+04 1.33E+05 3.05E+04 4.50E+04

up

2.94E+03 8.81E+02 2.77E+03 1.39E+03 2.07E+03 1.91E+03 5.56E+03 8.72E+03

down

down

down

down

down

down

down

down

down

down

down

down

down

down

up

down 4.55E+04 8.63E+03 4.58E+04 8.25E+04 4.85E+04 4.44E+04 9.00E+00 9.00E+00

down 3.90E+03 6.10E+02 3.55E+03 8.99E+03 7.24E+03 3.06E+03 9.00E+00 9.00E+00

FALSE 4.94E+05 5.86E+05 5.12E+05 2.66E+05 6.62E+05 4.20E+05 2.58E+05 3.44E+05

down 1.48E+06 1.29E+06 1.61E+06 1.30E+06 1.71E+06 1.14E+06 4.13E+05 3.83E+05

down 1.93E+06 1.59E+06 1.80E+06 1.10E+06 2.49E+06 1.19E+06 6.44E+05 9.84E+05

down 3.05E+06 3.49E+06 3.33E+06 2.75E+06 5.86E+06 2.19E+06 1.51E+06 1.74E+06

down 8.50E+04 7.00E+04 9.74E+04 1.73E+05 4.60E+04 9.20E+04 9.00E+00 9.00E+00

down 2.69E+06 2.74E+06 2.65E+06 2.27E+06 2.79E+06 2.36E+06 3.17E+05 4.32E+05

down 1.87E+04 1.41E+04 3.24E+04 5.00E+04 4.37E+04 4.85E+04 9.00E+00 9.00E+00

down 1.89E+04 1.81E+04 2.35E+04 1.98E+04 1.81E+04 1.89E+04 1.78E+03 7.62E+03

down 7.97E+04 1.04E+05 1.62E+05 9.48E+04 2.76E+05 2.73E+05 1.91E+04 1.82E+04

down 5.20E+03 4.61E+03 4.16E+03 3.11E+03 5.65E+03 7.71E+03 9.00E+00 9.00E+00

down 3.34E+04 2.17E+04 4.86E+04 1.37E+05 7.67E+04 9.20E+04 7.61E+03 9.40E+03

down 6.35E+03 9.74E+03 6.64E+03 2.72E+03 8.65E+03 7.38E+03 9.00E+00 9.00E+00

up

1.42E+05 1.65E+05 1.57E+05 3.22E+04 1.40E+05 1.09E+05 4.98E+05 4.48E+05

down

down 8.67E+04 6.84E+04 1.70E+05 1.05E+05 3.00E+05 2.27E+05 4.71E+04 4.00E+04

 

DYA3

cpd_ID kegg_map

4.36E+06 C01746 ko00960

1.77E+06 C01026 00260,ko011

1.90E+04 C01987 00380,ko011

1.81E+06 C00148 0,ko01100,k

5.53E+07 C00183 00966,ko009

2.82E+06

--

--

1.22E+04 C00097 00730,ko007

1.06E+04 C02237 ko01100

1.53E+05 C00430 0,ko01100,k

2.27E+07 C01157 0,ko01100,k

3.80E+04 C00300 0,ko00330,k

3.67E+06 C02378 ko01100

5.99E+07 C00123 0,ko01100,k

4.50E+06

6.11E+07 C01933

--

--

--

6.34E+07 C00407 6,ko00970,k

3.93E+06 C21096 --

2.68E+04 C00152 00970,ko011

1.79E+05 C00077 0,ko01100,k

4.87E+05 C22040 ko02010

4.88E+05 C15561

3.75E+05 --

--

--

1.67E+05 C00568 00790,ko011

4.93E+04 C00805 01100,ko011

5.13E+06 C00860 01100,ko011

2.38E+05 C10172

--

--

--

--

--

1.00E+06

6.50E+05

--

--

3.04E+05 C21514

2.29E+05 --

3.45E+06 C00064 0,ko00750,k

2.85E+06 C00047 00970,ko011

2.29E+05 C02614 00660,ko011

7.44E+06 C00650

5.99E+06 C00073 0,ko01100,k

8.61E+04 C02216 --

--

1.23E+05 C00121 0,ko01100,k

1.41E+05 C00242 00230,ko011

2.27E+05

--

--

2.89E+06 C00755 8,ko01100,k

3.50E+04 C09847 ko01110

3.06E+05 C00196 ko01110

2.40E+05 C03087 00310,ko011

8.34E+03 C19837

--

 

3.36E+05 C03761

--

4.88E+04 C12621 00360,ko011

2.57E+07 C00079 6,ko00970,k

1.48E+07 C16614

--

1.09E+04 C03722 00410,ko007

9.57E+04 C06672

2.22E+04 --

--

--

7.34E+04 C00314 00750,ko011

7.93E+04 -- --

7.50E+06 C00437 0,ko01110,k

1.86E+05 -- --

1.06E+05 C01042 00250,ko011

1.65E+06 C00327 01100,ko011

1.65E+05

--

--

6.68E+05 C00072 00480,ko011

3.29E+04 C02670 00053,ko011

1.76E+06 C04209

--

6.42E+05 C02504 01100,ko011

5.70E+05 C05994 ko00620

6.29E+05 C04411 0,ko01110,k

8.56E+04 C01197 0,ko01100,k

8.34E+06 C00031 0,ko00520,k

1.01E+07 C00137 2,ko01100,k

7.97E+06 C00124 00052,ko011

1.11E+07 C00159 2,ko00520,k

1.05E+07 C05003

--

8.96E+04 C00590 00998,ko011

3.52E+04

6.98E+04

--

--

--

--

2.80E+07 C00082 0,ko00950,k

2.36E+07 -- --

1.02E+05 C00794 00052,ko011

3.22E+04 C00392 1,ko01100,k

9.88E+04 C01697 00052,ko011

2.74E+05 C00847 00750,ko011

4.08E+06

4.27E+06

4.03E+04 C02155

3.30E+04 --

7.36E+03 C16987

4.66E+04 --

--

--

--

--

--

--

--

--

--

--

4.07E+04 C17853

9.00E+00 C10477

1.47E+06 C01494 0,ko01100,k

2.00E+04 C07481 2,ko01100,k

9.98E+03

--

--

 

2.70E+05 C03626

--

1.52E+07 C00078 6,ko00970,k

1.82E+05 C05944 ko00770

3.48E+03

--

--

3.60E+04 C00672 0,ko00240,k

3.01E+04

1.76E+04

5.73E+04

--

--

--

--

--

--

--

9.00E+00 C22007

7.46E+03 C00482 0,ko01100,k

3.20E+05

9.06E+04

4.87E+04

5.13E+04

--

--

--

--

--

--

--

--

--

2.42E+04 C03592

8.19E+04 C00214 00240,ko011

5.18E+05 C02961 --

3.98E+05 C00475 0,ko01100,k

8.29E+04 C00299 0,ko01100,k

6.02E+04 C16908

--

--

--

--

1.12E+05

3.77E+04

1.18E+05

--

--

--

6.92E+03 C00669 00480,ko011

3.15E+06 -- --

1.50E+05 C00559 0,ko01100,k

1.35E+04 C05512 0,ko01100,k

4.08E+05 C10497 00330,ko011

2.52E+05 C00330 0,ko01100,k

1.18E+07 C00212 0,ko01100,k

1.14E+07

6.47E+04

3.47E+04

9.90E+04

3.52E+06

4.18E+04

4.47E+04

--

--

--

--

--

--

--

--

--

--

--

--

--

--

--

1.21E+05 C08432

1.92E+06 C00387 0,ko01100,k

8.93E+04 C13846 --

5.03E+04 C01762 2,ko01100,k

9.00E+00

6.26E+04

--

--

--

--

1.14E+05 C16342 ko00592

5.66E+04 C16346 ko00592

4.33E+03 C14766 ko00591

 

1.07E+04 C16316 ko00592

6.98E+04 -- --

6.44E+06 C00170 0,ko00908,k

4.44E+04 C20674

3.78E+03 --

9.44E+04 C08365

--

--

--

--

--

--

--

--

--

1.18E+05

1.53E+06

--

--

1.68E+04 C10105

3.50E+05

4.04E+04

--

--

3.63E+04 C05324

1.74E+05 C16321 00592,ko011

1.16E+05 -- --

3.69E+03 C21923 00592,ko011

2.03E+04 C16526 ko01040

3.40E+04

2.91E+04

9.00E+00

1.08E+05

--

--

--

--

--

--

--

--

2.45E+04 C14827 00591,ko011

6.06E+04 C07354 ko00591

3.37E+06 C04717 00591,ko011

1.11E+05

4.05E+04

--

--

--

--

7.24E+04 C14829 ko00591

8.85E+03

1.68E+07

1.63E+07

5.32E+04

1.07E+05

1.71E+07

1.08E+04

--

--

--

--

--

--

--

--

--

--

--

--

--

--

4.33E+05 C12144 00600,ko011

3.05E+03 -- --

1.53E+04 C02906 00941,ko011

1.07E+04 -- --

1.27E+04 C18699 ko04075

7.01E+04 C00105 00240,ko011

6.31E+05

6.72E+05

1.01E+06

1.72E+06

7.84E+05 C06739

1.06E+06 C04415

--

--

--

--

--

--

--

--

--

--

--

9.22E+06

--

 

1.80E+05 C10650

1.70E+04 C00575 00230,ko011

2.59E+04 -- --

4.34E+06 C06196 ko00230

--

9.00E+00

5.21E+03

9.00E+00

1.31E+06

1.35E+06

1.25E+04

--

--

--

--

--

--

--

--

--

--

--

--

1.03E+06 C12208 00941,ko009

4.13E+04

7.75E+03 C09264

--

--

--

9.40E+04 C02887 ko00940

9.00E+00 -- --

8.00E+03 C08281 00073,ko010

4.03E+05

9.00E+00

2.63E+05

--

--

--

--

--

--

3.25E+05 C01235 00052,ko011

3.33E+05 C00761 ko00940

9.00E+00

3.49E+03 C10101

2.11E+05

1.10E+04

9.74E+03

--

--

--

--

--

--

--

--

--

2.40E+05 C00942 00230,ko011

3.35E+04

9.32E+04 C17147

4.81E+04 --

--

--

--

--

1.54E+04 C10882 ko01110

6.15E+03

1.38E+04

1.28E+05

2.69E+03 C17855

2.90E+04

1.02E+06

1.34E+05 C02572

5.99E+06

6.85E+04

6.61E+04 C10725

9.97E+03

6.65E+03

--

--

--

--

--

--

--

--

--

--

--

--

--

--

--

--

--

--

--

--

--

--

--

--

4.04E+05 C02919

9.00E+00 --

9.00E+00 C10561

 

8.11E+03

1.50E+05

2.97E+05

9.00E+00

8.86E+04

2.56E+05

--

--

--

--

--

--

--

--

--

--

--

--

--

--

--

--

--

--

--

--

3.84E+03 C10112

5.14E+05

3.82E+05

6.24E+02

9.00E+00

1.34E+06

1.71E+04

2.12E+04

--

--

--

--

--

--

--

4.88E+04 C00008 00230,ko009

9.60E+04 -- --

6.76E+04 C16911 ko00944

1.54E+04 C17564

--

--

--

--

--

--

--

--

--

--

--

1.92E+04

1.74E+04

8.45E+05

9.00E+00

1.77E+05

1.84E+05

7.99E+03

1.84E+04

1.88E+04

8.41E+04

--

--

--

--

--

--

--

--

--

--

6.39E+04 C01750 ko00944

8.36E+04 C03951 ko00944

1.95E+05

1.29E+05

--

--

--

--

1.53E+05 C12249 00944,ko011

1.48E+04

3.32E+04

9.00E+00

2.55E+04

6.04E+06

6.25E+05

1.53E+05

6.70E+04 C09731

9.18E+04

9.00E+00

--

--

--

--

--

--

--

--

--

--

--

--

--

--

--

--

--

--

--

--

--

--

9.00E+00 C10533

5.86E+04

9.00E+00

--

--

 

3.36E+05 C05623 4,ko01100,k

2.16E+05

2.03E+05

1.95E+04

8.15E+04

7.17E+04

3.77E+04

2.43E+03

7.14E+04

--

--

--

--

--

--

--

--

--

--

--

--

--

--

--

--

--

--

--

--

--

--

--

--

2.50E+05 C16912

2.56E+05

5.97E+04

8.11E+04

2.30E+05

8.19E+04

--

--

--

--

--

1.72E+05 C16939

6.56E+04 --

1.42E+06 C00234 00970,ko011

5.30E+05 C10437

--

--

--

--

--

--

--

--

--

--

--

--

--

--

--

--

--

--

--

--

--

--

--

--

--

--

--

--

5.61E+04

2.03E+06

9.00E+00

3.10E+04

1.80E+05

1.90E+05

9.00E+00

1.48E+05

1.63E+05

1.35E+05

1.67E+05

2.60E+05

5.84E+04

1.17E+05

5.90E+05

8.16E+04

2.47E+03

1.10E+02

6.98E+03

9.06E+03

1.39E+05

8.76E+04

9.00E+00

2.54E+04

9.00E+00

4.82E+03

2.19E+07

--

--

--

--

--

--

--

--

--

--

--

--

--

--

--

--

--

--

--

--

--

--

--

--

--

--

--

 

9.69E+05

5.91E+04

1.09E+06 C08243

--

--

--

--

--

9.78E+03 C05404 00052,ko011

1.53E+06 C00492 2,ko01100,k

4.11E+04

4.51E+04

2.62E+04

1.89E+04

4.97E+04

7.27E+03

4.44E+05

3.86E+05

1.13E+07

9.33E+06

6.52E+04

1.21E+05

9.00E+00

--

--

--

--

--

--

--

--

--

--

--

--

--

--

--

--

--

--

--

--

--

--

--

--

--

--

--

--

--

--

--

--

--

--

--

--

--

--

--

--

--

--

--

--

--

--

--

--

--

--

--

--

1.27E+05 C17529

9.00E+00

1.18E+06

2.47E+05

1.25E+05

1.68E+05

1.69E+05

1.23E+05

6.00E+05

3.69E+04

1.42E+04

9.00E+00

9.00E+00

2.96E+03

1.07E+05

2.95E+04

7.22E+04

8.06E+04

1.89E+04

2.07E+05

2.18E+04

5.58E+04

4.52E+04

5.92E+05

5.27E+05

3.53E+04

--

--

--

--

--

--

--

--

--

--

--

--

--

--

--

--

--

--

--

--

--

--

--

--

--

8.85E+06 C20490 ko00942

7.15E+04 -- --

 

4.16E+05 C17563

--

--

--

--

--

1.15E+06

9.46E+05

1.10E+06

3.63E+04

--

--

--

--

9.69E+04 C09806 ko00941

1.07E+06 -- --

3.23E+04 C16306 ko00942

3.18E+04

1.72E+04

5.49E+03

9.00E+00

5.23E+04

3.20E+05

9.00E+00

3.66E+05

--

--

--

--

--

--

--

--

--

--

--

--

--

--

--

--

3.27E+05 C12667 ko00944

9.00E+00

2.53E+04

2.55E+05

2.61E+05

2.25E+05

9.00E+00

4.03E+03

9.00E+00

9.00E+00

2.19E+05

9.03E+03

1.56E+04

1.03E+04

9.00E+00

9.64E+04

6.91E+04

9.00E+00

5.64E+04

1.45E+05

9.00E+00

1.61E+05

6.24E+05

1.60E+06

2.65E+04

9.00E+00

1.20E+06

9.00E+00

1.47E+05

--

--

--

--

--

--

--

--

--

--

--

--

--

--

--

--

--

--

--

--

--

--

--

--

--

--

--

--

--

--

--

--

--

--

--

--

--

--

--

--

--

--

--

--

--

--

--

--

--

--

--

--

--

--

--

--

--

3.32E+05 C20779

 

7.94E+04

1.63E+04

--

--

--

--

--

--

--

--

--

--

--

--

--

--

--

--

--

--

--

--

--

--

--

--

--

--

--

--

--

--

--

5.49E+05 C08946

4.63E+05 --

4.15E+05 C08945

8.26E+04

9.00E+00

9.00E+00

9.00E+00

9.00E+00

9.00E+00

1.30E+05

1.06E+04

9.00E+00

9.00E+00

1.46E+05

--

--

--

--

--

--

--

--

--

--

--

4.78E+05 C08961

4.88E+05 C20725

6.07E+05 C20736

9.00E+00

5.11E+05

9.00E+00

2.37E+03

3.07E+04

9.00E+00

3.57E+03

9.00E+00

3.59E+05

3.78E+04

--

--

--

--

--

--

--

--

--

--
