# Supplementary material for: Metabolome and transcriptome analyses identify the characteristics and expression of related saponins of the three genealogical plants of bead ginseng
Source: PeerJ. 2023 Sep 1;11:e16034. doi: 10.7717/peerj.16034 (PMC10476608; doi:10.7717/peerj.16034)
Supplement: Supplementary file 4 [file peerj-11-16034-s009.odt]

Table S3 KEGG enrichment outcome of differential genes of XL vs

Cluster_fGenome_frP-value Correctedcount

KEGG_pathko_id

GeneID KEGG OrthUp

266 EVM002015K00002+K0

177 EVM004927K02927+K0

83 EVM003108K00031+K0

276 EVM006390K01230+K0

46 EVM002974K00053+K0

111 EVM004707K00088+K0

68 EVM001634K00021+K0

111 EVM003108K00031+K0

22 EVM005399K00275+K0

130 EVM002492K00003+K0

49 EVM000409K00914+K0

41 EVM001494K00993+K0

45 EVM004849K00380+K0

76 EVM002664K00030+K0

121 EVM003255K01164+K0

17 EVM001092K00133+K0

20 EVM002937K00728+K0

32 EVM005693K00261+K0

27 EVM001931K00052+K0

80 EVM003151K02725+K0

259 EVM001260K03283+K0

3 EVM002937K00728+K0

71 EVM005340K00025+K0

29 EVM002636K00939+K0

55 EVM005364K00224+K0

34 EVM006373K00432+K0

61 EVM004857K00789+K0

66 EVM002492K00003+K0

44 novel.580K02202+K0

272 EVM002492K00003+K0

224 EVM005019K00688+K0

42 EVM003533K00232+K0

116 EVM002519K00006+K0

6 EVM004892K03644+K0

11 EVM005871K12338+K1

25 EVM003671K00278+K0

84 EVM005340K00025+K0

13 EVM002492K00003+K0

10 EVM004566K00128+K0

85 EVM005818K01866+K0

11 EVM001931K00052+K0

43 EVM003676K00140+K0

1389 EVM000873K00001+K0

125 EVM004208K00012+K0

116 EVM000873K00001+K0

Biosyntheko01240 266 out o1021 out 6.23E-05 0.008965

Ubiquitinko04120 177 out o656 out o 0.000159 0.022903

Glutathioko00480 83 out of273 out o 0.000179 0.025707

Protein pko04141 276 out o1092 out 0.000389 0.056015

Pantothenko00770 46 out of137 out o 0.000459 0.066097

Purine meko00230 111 out o396 out o 0.00058 0.083485

Terpenoidko00900 68 out of223 out o 0.000592 0.085313

Peroxisomko04146 111 out o404 out o 0.001247 0.179593

Vitamin Bko00750 22 out of56 out of 0.00146 0.210267

Cysteine ko00270 130 out o491 out o 0.002318 0.333723

Autophagyko04136 49 out of159 out o 0.002509 0.361315

Ether lipko00565 41 out of130 out o 0.003406 0.490435

Sulfur meko00920 45 out of146 out o 0.003649 0.525408

2-Oxocarbko01210 76 out of274 out o 0.005142 0.740413

Ribosome ko03008 121 out o464 out o 0.005441 0.78345

110

90

29

81

19

58

27

49

3

54

28

23

15

36

55

8

Monobactako00261 17 out of46 out of 0.009853

Other typko00514 20 out of57 out of 0.010272

Arginine ko00220 32 out of105 out o 0.015071

Valine, lko00290 27 out of86 out of 0.01635

Proteasomko03050 80 out of305 out o 0.017788

Spliceosoko03040 259 out o1095 out 0.019366

Mannose tko00515 3 out of 4 out of 0.031558

Citrate cko00020 71 out of279 out o 0.044819

Thiamine ko00730 29 out of103 out o 0.053878

alpha-Linko00592 55 out of214 out o 0.059786

Arachidonko00590 34 out of125 out o 0.061342

Biosyntheko00999 61 out of241 out o 0.064528

Glycine, ko00260 66 out of264 out o 0.070065

Basal trako03022 44 out of169 out o 0.070946

Biosyntheko01230 272 out o1194 out 0.076962

Starch anko00500 224 out o981 out o 0.093034

Biosyntheko01040 42 out of164 out o 0.094

Glycerophko00564 116 out o492 out o 0.095999

Lipoic acko00785 6 out of 16 out of 0.100765

Anthocyanko00942 11 out of35 out of 0.101319

Nicotinatko00760 25 out of93 out of 0.108531

Glyoxylatko00630 84 out of352 out o 0.112197

Lysine biko00300 13 out of45 out of 0.136344

Limonene ko00903 10 out of33 out of 0.139688

Aminoacylko00970 85 out of362 out o 0.144887

C5-Branchko00660 11 out of38 out of 0.160418

Propanoatko00640 43 out of178 out o 0.179073

Biosyntheko01110 1389 out 6461 out 0.180316

Biosyntheko01250 125 out o553 out o 0.201923

Pyruvate ko00620 116 out o512 out o 0.203782

1

1

1

1

1

1

1

1

1

1

1

1

1

1

1

1

1

1

1

1

1

1

1

1

1

1

1

1

1

1

6

13

10

32

95

0

24

17

26

13

30

28

20

117

104

12

59

3

5

14

33

10

5

40

4

16

590

62

41

 

Arginine ko00330 48 out of203 out o 0.207031

Phosphatiko04070 75 out of326 out o 0.214221

Non-homolko03450 7 out of 24 out of 0.228471

Glycosylpko00563 25 out of102 out o 0.230041

Endocytosko04144 205 out o929 out o 0.239234

Monoterpeko00902 16 out of63 out of 0.241695

Porphyrinko00860 42 out of179 out o 0.242046

Carotenoiko00906 41 out of175 out o 0.248562

Valine, lko00280 65 out of285 out o 0.257451

beta-Alanko00410 40 out of172 out o 0.268494

Phosphonako00440 6 out of 22 out of 0.312168

Phagosomeko04145 79 out of355 out o 0.313145

ABC transko02010 90 out of408 out o 0.332234

Ubiquinonko00130 45 out of200 out o 0.337658

Ribosome ko03010 259 out o1200 out 0.344015

Nucleotidko03420 61 out of275 out o 0.34989

Histidineko00340 20 out of86 out of 0.350653

Fatty aciko00071 51 out of229 out o 0.35439

One carboko00670 17 out of73 out of 0.365911

Amino sugko00520 167 out o774 out o 0.381244

Biotin meko00780 22 out of97 out of 0.388532

Inositol ko00562 79 out of363 out o 0.395453

Glyceroliko00561 92 out of426 out o 0.416332

Butanoateko00650 17 out of76 out of 0.436338

Protein eko03060 62 out of289 out o 0.462088

Sulfur reko04122 8 out of 35 out of 0.464106

Flavone ako00944 12 out of54 out of 0.471971

Nucleocytko03013 187 out o881 out o 0.472347

Glucosinoko00966 8 out of 36 out of 0.498797

Taurine ako00430 7 out of 32 out of 0.525781

Selenocomko00450 28 out of133 out o 0.538073

Steroid bko00100 25 out of119 out o 0.544536

Fatty aciko01212 91 out of436 out o 0.563089

Plant-patko04626 386 out o1842 out 0.566117

Alanine, ko00250 40 out of193 out o 0.577445

Circadianko04712 62 out of299 out o 0.58217

Benzoxaziko00402 8 out of 39 out of 0.597855

Riboflaviko00740 19 out of93 out of 0.60239

Caffeine ko00232 1 out of 4 out of 0.612206

Plant horko04075 377 out o1809 out 0.61408

Betalain ko00965 4 out of 20 out of 0.63465

Carbon meko01200 277 out o1339 out 0.653905

Viral lifko03250 38 out of191 out o 0.684601

Polyketidko00523 1 out of 5 out of 0.693987

RNA degrako03018 122 out o605 out o 0.72764

Lysine deko00310 38 out of195 out o 0.734691

1

1

1

1

1

1

1

1

1

1

1

1

1

1

1

1

1

1

1

1

1

1

1

1

1

1

1

1

1

1

1

1

1

1

1

1

1

1

1

1

1

1

1

1

1

1

48 EVM004566K00128+K0

75 EVM002091K00889+K0

7 EVM002655K03512+K1

25 EVM003896K03857+K0

205 EVM002091K00889+K0

16 EVM004481K15095+K1

42 EVM005927K00218+K0

41 EVM003633K00514+K0

65 EVM003429K00020+K0

40 EVM004566K00128+K0

6 EVM005665K00967+K0

79 EVM000409K00914+K0

90 EVM003589K02045+K0

45 EVM000342K00457+K0

259 EVM005140K02863+K0

61 novel.580K02202+K0

20 EVM006576K00013+K0

51 EVM000873K00001+K0

17 EVM000426K00288+K0

167 EVM004208K00012+K0

22 EVM004121K00059+K0

79 EVM004648K00010+K0

92 EVM002015K00002+K0

17 EVM004486K00074+K0

62 EVM001663K03070+K0

8 EVM000072K01011+K0

12 EVM001507K05280+K1

187 EVM005173K03231+K0

8 EVM002787K00826+K0

7 EVM000595K00485+K0

28 EVM005874K00384+K0

25 EVM001576K00222+K0

91 EVM004121K00059+K0

386 EVM004954K01373+K0

40 EVM005693K00261+K0

62 EVM002669K00660+K0

8 EVM006167K13229+K1

19 EVM000032K00793+K0

1 EVM005397K00365

20

35

3

16

78

6

25

21

30

19

1

31

50

17

104

31

10

27

4

77

11

40

36

6

29

2

3

84

1

6

8

12

34

184

20

32

1

9

0

377 EVM002226K12126+K1

4 EVM002761K01593+K0

277 EVM005340K00025+K0

38 EVM000906K04498+K0

1 EVM005896K12451

205

4

105

16

0

122 EVM004734K00850+K0

38 EVM004566K00128+K0

58

19

 

Neomycin,ko00524 6 out of 34 out of 0.751729

Other glyko00511 57 out of291 out o 0.756477

Folate biko00790 24 out of127 out o 0.759419

Indole alko00901 6 out of 35 out of 0.7772

Tryptophako00380 45 out of235 out o 0.789889

Mismatch ko03430 42 out of221 out o 0.799493

Brassinosko00905 12 out of69 out of 0.814859

Pyrimidinko00240 50 out of264 out o 0.824641

MAPK signko04016 243 out o1217 out 0.844904

Ascorbateko00053 65 out of342 out o 0.845227

Glycosphiko00601 1 out of 8 out of 0.84964

D-Amino ako00470 3 out of 21 out of 0.850542

Sphingoliko00600 56 out of298 out o 0.852923

Homologouko03440 52 out of279 out o 0.860812

Tyrosine ko00350 38 out of208 out o 0.861785

Glycosphiko00603 7 out of 45 out of 0.864976

Nucleotidko01232 66 out of351 out o 0.869586

Fatty aciko00061 47 out of255 out o 0.869794

Phenylalako00400 37 out of204 out o 0.87021

Glycolysiko00010 150 out o769 out o 0.872187

Zeatin biko00908 25 out of144 out o 0.887093

SNARE intko04130 26 out of153 out o 0.913322

Galactoseko00052 78 out of422 out o 0.918038

Phenylalako00360 34 out of198 out o 0.928264

Glycosamiko00531 30 out of179 out o 0.93841

Nitrogen ko00910 22 out of137 out o 0.943448

Fructose ko00051 73 out of405 out o 0.945401

Metabolicko01100 2423 out 11773 out 0.963918

Phenylproko00940 133 out o720 out o 0.964743

Glycosphiko00604 21 out of137 out o 0.96497

Isoflavonko00943 14 out of99 out of 0.970522

Cyanoaminko00460 46 out of280 out o 0.979385

Cutin, suko00073 24 out of161 out o 0.981841

Linoleic ko00591 13 out of101 out o 0.988056

Isoquinolko00950 27 out of185 out o 0.990555

Stilbenoiko00945 24 out of169 out o 0.991564

N-Glycan ko00510 34 out of234 out o 0.995981

Fatty aciko00062 17 out of137 out o 0.997026

mRNA survko03015 140 out o811 out o 0.99753

Photosyntko00196 4 out of 55 out of 0.998639

Various tko00513 28 out of211 out o 0.998757

Flavonoidko00941 36 out of258 out o 0.998776

Pentose pko00030 56 out of379 out o 0.999353

Carbon fiko00710 69 out of457 out o 0.999583

Diterpenoko00904 12 out of121 out o 0.999657

RNA polymko03020 54 out of378 out o 0.999741

1

1

1

1

1

1

1

1

1

1

1

1

1

1

1

1

1

1

1

1

1

1

1

1

1

1

1

1

1

1

1

1

1

1

1

1

1

1

1

1

1

1

1

1

1

1

6 EVM001258K00844+K0

57 EVM000988K01190+K0

24 EVM005231K01113+K0

6 EVM002761K01593+K0

45 EVM004566K00128+K0

42 EVM002393K03111+K0

12 EVM000053K09588+K0

50 EVM001722K00207+K0

243 EVM006390K00940+K0

65 EVM002015K00002+K0

1 EVM004328K01988

3

22

13

5

26

21

7

24

108

20

0

3 EVM000441K01778+K1

56 EVM004388K00654+K0

52 EVM006246K02335+K0

38 EVM000873K00001+K0

7 EVM004328K01988+K0

66 EVM004707K00088+K0

47 EVM004121K00059+K0

37 EVM003230K00766+K0

150 EVM000873K00001+K0

25 EVM003102K00279+K0

26 EVM003519K08486+K0

78 EVM001258K00844+K0

34 EVM004486K00074+K0

30 EVM001261K01205+K0

22 EVM005693K00261+K0

73 EVM004755K00008+K0

2423 EVM000873K00001+K0

133 EVM005557K00083+K0

21 EVM002323K12309+K1

14 EVM003731K13258+K1

46 EVM001652K00600+K0

24 EVM001169K13356+K1

13 EVM000455K00454+K0

27 EVM004400K00276+K0

24 EVM004011K00487+K0

34 EVM003456K00721+K0

17 EVM003033K01074+K0

140 EVM001253K03265+K0

4 EVM000522K08911+K0

28 EVM003097K00736+K0

36 EVM004011K00487+K0

56 EVM004504K00033+K0

69 EVM005340K00025+K0

12 EVM004553K04120+K0

54 EVM002358K02999+K0

3

26

31

18

4

34

17

15

54

13

13

33

17

13

8

37

1060

57

12

5

17

15

9

11

11

23

7

70

2

15

17

18

31

5

28

 

Base exciko03410 58 out of405 out o 0.999823

Tropane, ko00960 19 out of176 out o 0.999897

Sesquiterko00909 18 out of176 out o 0.999958

Pentose ako00040 116 out o756 out o 0.99998

1

1

1

1

1

1

1

58 novel.121K01142+K0

19 EVM004400K00276+K0

18 EVM004040K00801+K0

116 EVM002015K00002+K0

53 EVM005212K02209+K0

138 EVM000608K00234+K0

15 EVM004036K02115+K0

30

10

8

44

24

53

6

DNA repliko03030 53 out of461 out o

Oxidativeko00190 138 out o1071 out

Photosyntko00195 15 out of297 out o

1

1

1

 

vs DY group

Up_gene Down

[EVM005745](file:///c:/website/convert/file/st34dm81hgmvqm3i3v5qk56zkeldkdeb/maps/ko01240.html)

[EVM004927](file:///c:/website/convert/file/st34dm81hgmvqm3i3v5qk56zkeldkdeb/maps/ko04120.html)

[novel.413](file:///c:/website/convert/file/st34dm81hgmvqm3i3v5qk56zkeldkdeb/maps/ko00480.html)

[EVM006390](file:///c:/website/convert/file/st34dm81hgmvqm3i3v5qk56zkeldkdeb/maps/ko04141.html)

[EVM002974](file:///c:/website/convert/file/st34dm81hgmvqm3i3v5qk56zkeldkdeb/maps/ko00770.html)

[EVM005933](file:///c:/website/convert/file/st34dm81hgmvqm3i3v5qk56zkeldkdeb/maps/ko00230.html)

[novel.966](file:///c:/website/convert/file/st34dm81hgmvqm3i3v5qk56zkeldkdeb/maps/ko00900.html)

[novel.413](file:///c:/website/convert/file/st34dm81hgmvqm3i3v5qk56zkeldkdeb/maps/ko04146.html)

[EVM003401](file:///c:/website/convert/file/st34dm81hgmvqm3i3v5qk56zkeldkdeb/maps/ko00750.html)

[novel.322](file:///c:/website/convert/file/st34dm81hgmvqm3i3v5qk56zkeldkdeb/maps/ko00270.html)

[EVM003973](file:///c:/website/convert/file/st34dm81hgmvqm3i3v5qk56zkeldkdeb/maps/ko04136.html)

[EVM001858](file:///c:/website/convert/file/st34dm81hgmvqm3i3v5qk56zkeldkdeb/maps/ko00565.html)

[EVM000135](file:///c:/website/convert/file/st34dm81hgmvqm3i3v5qk56zkeldkdeb/maps/ko00920.html)

[EVM002664](file:///c:/website/convert/file/st34dm81hgmvqm3i3v5qk56zkeldkdeb/maps/ko01210.html)

[EVM002775](file:///c:/website/convert/file/st34dm81hgmvqm3i3v5qk56zkeldkdeb/maps/ko03008.html)

[EVM002531](file:///c:/website/convert/file/st34dm81hgmvqm3i3v5qk56zkeldkdeb/maps/ko00261.html)

[EVM005254](file:///c:/website/convert/file/st34dm81hgmvqm3i3v5qk56zkeldkdeb/maps/ko00514.html)

[EVM001260](file:///c:/website/convert/file/st34dm81hgmvqm3i3v5qk56zkeldkdeb/maps/ko00220.html)

[EVM001931](file:///c:/website/convert/file/st34dm81hgmvqm3i3v5qk56zkeldkdeb/maps/ko00290.html)

[EVM003980](file:///c:/website/convert/file/st34dm81hgmvqm3i3v5qk56zkeldkdeb/maps/ko03050.html)

[EVM002021](file:///c:/website/convert/file/st34dm81hgmvqm3i3v5qk56zkeldkdeb/maps/ko03040.html)

--

[Down_genehyperlink](file:///c:/website/convert/file/st34dm81hgmvqm3i3v5qk56zkeldkdeb/maps/ko01240.html)

156[EVM002015](file:///c:/website/convert/file/st34dm81hgmvqm3i3v5qk56zkeldkdeb/maps/ko01240.html)[https:\\www.genome.jp\dbget-bin\www_bget?map01240](file:///c:/website/convert/file/st34dm81hgmvqm3i3v5qk56zkeldkdeb/maps/ko01240.html)

87[novel.450](file:///c:/website/convert/file/st34dm81hgmvqm3i3v5qk56zkeldkdeb/maps/ko04120.html)[https:\\www.genome.jp\dbget-bin\www_bget?map04120](file:///c:/website/convert/file/st34dm81hgmvqm3i3v5qk56zkeldkdeb/maps/ko04120.html)

54[EVM003108](file:///c:/website/convert/file/st34dm81hgmvqm3i3v5qk56zkeldkdeb/maps/ko00480.html)[https:\\www.genome.jp\dbget-bin\www_bget?map00480](file:///c:/website/convert/file/st34dm81hgmvqm3i3v5qk56zkeldkdeb/maps/ko00480.html)

195[EVM006133](file:///c:/website/convert/file/st34dm81hgmvqm3i3v5qk56zkeldkdeb/maps/ko04141.html)[https:\\www.genome.jp\dbget-bin\www_bget?map04141](file:///c:/website/convert/file/st34dm81hgmvqm3i3v5qk56zkeldkdeb/maps/ko04141.html)

27[EVM002954](file:///c:/website/convert/file/st34dm81hgmvqm3i3v5qk56zkeldkdeb/maps/ko00770.html)[https:\\www.genome.jp\dbget-bin\www_bget?map00770](file:///c:/website/convert/file/st34dm81hgmvqm3i3v5qk56zkeldkdeb/maps/ko00770.html)

53[EVM004707](file:///c:/website/convert/file/st34dm81hgmvqm3i3v5qk56zkeldkdeb/maps/ko00230.html)[https:\\www.genome.jp\dbget-bin\www_bget?map00230](file:///c:/website/convert/file/st34dm81hgmvqm3i3v5qk56zkeldkdeb/maps/ko00230.html)

41[EVM001634](file:///c:/website/convert/file/st34dm81hgmvqm3i3v5qk56zkeldkdeb/maps/ko00900.html)[https:\\www.genome.jp\dbget-bin\www_bget?map00900](file:///c:/website/convert/file/st34dm81hgmvqm3i3v5qk56zkeldkdeb/maps/ko00900.html)

62[EVM003108](file:///c:/website/convert/file/st34dm81hgmvqm3i3v5qk56zkeldkdeb/maps/ko04146.html)[https:\\www.genome.jp\dbget-bin\www_bget?map04146](file:///c:/website/convert/file/st34dm81hgmvqm3i3v5qk56zkeldkdeb/maps/ko04146.html)

19[EVM005399](file:///c:/website/convert/file/st34dm81hgmvqm3i3v5qk56zkeldkdeb/maps/ko00750.html)[https:\\www.genome.jp\dbget-bin\www_bget?map00750](file:///c:/website/convert/file/st34dm81hgmvqm3i3v5qk56zkeldkdeb/maps/ko00750.html)

76[EVM002492](file:///c:/website/convert/file/st34dm81hgmvqm3i3v5qk56zkeldkdeb/maps/ko00270.html)[https:\\www.genome.jp\dbget-bin\www_bget?map00270](file:///c:/website/convert/file/st34dm81hgmvqm3i3v5qk56zkeldkdeb/maps/ko00270.html)

21[EVM000409](file:///c:/website/convert/file/st34dm81hgmvqm3i3v5qk56zkeldkdeb/maps/ko04136.html)[https:\\www.genome.jp\dbget-bin\www_bget?map04136](file:///c:/website/convert/file/st34dm81hgmvqm3i3v5qk56zkeldkdeb/maps/ko04136.html)

18[EVM001494](file:///c:/website/convert/file/st34dm81hgmvqm3i3v5qk56zkeldkdeb/maps/ko00565.html)[https:\\www.genome.jp\dbget-bin\www_bget?map00565](file:///c:/website/convert/file/st34dm81hgmvqm3i3v5qk56zkeldkdeb/maps/ko00565.html)

30[EVM004849](file:///c:/website/convert/file/st34dm81hgmvqm3i3v5qk56zkeldkdeb/maps/ko00920.html)[https:\\www.genome.jp\dbget-bin\www_bget?map00920](file:///c:/website/convert/file/st34dm81hgmvqm3i3v5qk56zkeldkdeb/maps/ko00920.html)

40[EVM001975](file:///c:/website/convert/file/st34dm81hgmvqm3i3v5qk56zkeldkdeb/maps/ko01210.html)[https:\\www.genome.jp\dbget-bin\www_bget?map01210](file:///c:/website/convert/file/st34dm81hgmvqm3i3v5qk56zkeldkdeb/maps/ko01210.html)

66[EVM003255](file:///c:/website/convert/file/st34dm81hgmvqm3i3v5qk56zkeldkdeb/maps/ko03008.html)[https:\\www.genome.jp\dbget-bin\www_bget?map03008](file:///c:/website/convert/file/st34dm81hgmvqm3i3v5qk56zkeldkdeb/maps/ko03008.html)

9[EVM001092](file:///c:/website/convert/file/st34dm81hgmvqm3i3v5qk56zkeldkdeb/maps/ko00261.html)[https:\\www.genome.jp\dbget-bin\www_bget?map00261](file:///c:/website/convert/file/st34dm81hgmvqm3i3v5qk56zkeldkdeb/maps/ko00261.html)

14[EVM002937](file:///c:/website/convert/file/st34dm81hgmvqm3i3v5qk56zkeldkdeb/maps/ko00514.html)[https:\\www.genome.jp\dbget-bin\www_bget?map00514](file:///c:/website/convert/file/st34dm81hgmvqm3i3v5qk56zkeldkdeb/maps/ko00514.html)

19[EVM005693](file:///c:/website/convert/file/st34dm81hgmvqm3i3v5qk56zkeldkdeb/maps/ko00220.html)[https:\\www.genome.jp\dbget-bin\www_bget?map00220](file:///c:/website/convert/file/st34dm81hgmvqm3i3v5qk56zkeldkdeb/maps/ko00220.html)

17[EVM005223](file:///c:/website/convert/file/st34dm81hgmvqm3i3v5qk56zkeldkdeb/maps/ko00290.html)[https:\\www.genome.jp\dbget-bin\www_bget?map00290](file:///c:/website/convert/file/st34dm81hgmvqm3i3v5qk56zkeldkdeb/maps/ko00290.html)

48[EVM003151](file:///c:/website/convert/file/st34dm81hgmvqm3i3v5qk56zkeldkdeb/maps/ko03050.html)[https:\\www.genome.jp\dbget-bin\www_bget?map03050](file:///c:/website/convert/file/st34dm81hgmvqm3i3v5qk56zkeldkdeb/maps/ko03050.html)

164[EVM001260](file:///c:/website/convert/file/st34dm81hgmvqm3i3v5qk56zkeldkdeb/maps/ko03040.html)[https:\\www.genome.jp\dbget-bin\www_bget?map03040](file:///c:/website/convert/file/st34dm81hgmvqm3i3v5qk56zkeldkdeb/maps/ko03040.html)

3[EVM002937](file:///c:/website/convert/file/st34dm81hgmvqm3i3v5qk56zkeldkdeb/maps/ko00515.html)[https:\\www.genome.jp\dbget-bin\www_bget?map00515](file:///c:/website/convert/file/st34dm81hgmvqm3i3v5qk56zkeldkdeb/maps/ko00515.html)

47[EVM005340](file:///c:/website/convert/file/st34dm81hgmvqm3i3v5qk56zkeldkdeb/maps/ko00020.html)[https:\\www.genome.jp\dbget-bin\www_bget?map00020](file:///c:/website/convert/file/st34dm81hgmvqm3i3v5qk56zkeldkdeb/maps/ko00020.html)

12[EVM000363](file:///c:/website/convert/file/st34dm81hgmvqm3i3v5qk56zkeldkdeb/maps/ko00730.html)[https:\\www.genome.jp\dbget-bin\www_bget?map00730](file:///c:/website/convert/file/st34dm81hgmvqm3i3v5qk56zkeldkdeb/maps/ko00730.html)

29[EVM005364](file:///c:/website/convert/file/st34dm81hgmvqm3i3v5qk56zkeldkdeb/maps/ko00592.html)[https:\\www.genome.jp\dbget-bin\www_bget?map00592](file:///c:/website/convert/file/st34dm81hgmvqm3i3v5qk56zkeldkdeb/maps/ko00592.html)

21[EVM002163](file:///c:/website/convert/file/st34dm81hgmvqm3i3v5qk56zkeldkdeb/maps/ko00590.html)[https:\\www.genome.jp\dbget-bin\www_bget?map00590](file:///c:/website/convert/file/st34dm81hgmvqm3i3v5qk56zkeldkdeb/maps/ko00590.html)

31[EVM004857](file:///c:/website/convert/file/st34dm81hgmvqm3i3v5qk56zkeldkdeb/maps/ko00999.html)[https:\\www.genome.jp\dbget-bin\www_bget?map00999](file:///c:/website/convert/file/st34dm81hgmvqm3i3v5qk56zkeldkdeb/maps/ko00999.html)

38[EVM002492](file:///c:/website/convert/file/st34dm81hgmvqm3i3v5qk56zkeldkdeb/maps/ko00260.html)[https:\\www.genome.jp\dbget-bin\www_bget?map00260](file:///c:/website/convert/file/st34dm81hgmvqm3i3v5qk56zkeldkdeb/maps/ko00260.html)

24[novel.580](file:///c:/website/convert/file/st34dm81hgmvqm3i3v5qk56zkeldkdeb/maps/ko03022.html)[https:\\www.genome.jp\dbget-bin\www_bget?map03022](file:///c:/website/convert/file/st34dm81hgmvqm3i3v5qk56zkeldkdeb/maps/ko03022.html)

155[EVM002492](file:///c:/website/convert/file/st34dm81hgmvqm3i3v5qk56zkeldkdeb/maps/ko01230.html)[https:\\www.genome.jp\dbget-bin\www_bget?map01230](file:///c:/website/convert/file/st34dm81hgmvqm3i3v5qk56zkeldkdeb/maps/ko01230.html)

120[EVM005019](file:///c:/website/convert/file/st34dm81hgmvqm3i3v5qk56zkeldkdeb/maps/ko00500.html)[https:\\www.genome.jp\dbget-bin\www_bget?map00500](file:///c:/website/convert/file/st34dm81hgmvqm3i3v5qk56zkeldkdeb/maps/ko00500.html)

30[EVM003533](file:///c:/website/convert/file/st34dm81hgmvqm3i3v5qk56zkeldkdeb/maps/ko01040.html)[https:\\www.genome.jp\dbget-bin\www_bget?map01040](file:///c:/website/convert/file/st34dm81hgmvqm3i3v5qk56zkeldkdeb/maps/ko01040.html)

57[EVM002519](file:///c:/website/convert/file/st34dm81hgmvqm3i3v5qk56zkeldkdeb/maps/ko00564.html)[https:\\www.genome.jp\dbget-bin\www_bget?map00564](file:///c:/website/convert/file/st34dm81hgmvqm3i3v5qk56zkeldkdeb/maps/ko00564.html)

3[EVM004892](file:///c:/website/convert/file/st34dm81hgmvqm3i3v5qk56zkeldkdeb/maps/ko00785.html)[https:\\www.genome.jp\dbget-bin\www_bget?map00785](file:///c:/website/convert/file/st34dm81hgmvqm3i3v5qk56zkeldkdeb/maps/ko00785.html)

6[EVM005871](file:///c:/website/convert/file/st34dm81hgmvqm3i3v5qk56zkeldkdeb/maps/ko00942.html)[https:\\www.genome.jp\dbget-bin\www_bget?map00942](file:///c:/website/convert/file/st34dm81hgmvqm3i3v5qk56zkeldkdeb/maps/ko00942.html)

11[EVM003671](file:///c:/website/convert/file/st34dm81hgmvqm3i3v5qk56zkeldkdeb/maps/ko00760.html)[https:\\www.genome.jp\dbget-bin\www_bget?map00760](file:///c:/website/convert/file/st34dm81hgmvqm3i3v5qk56zkeldkdeb/maps/ko00760.html)

51[EVM005340](file:///c:/website/convert/file/st34dm81hgmvqm3i3v5qk56zkeldkdeb/maps/ko00630.html)[https:\\www.genome.jp\dbget-bin\www_bget?map00630](file:///c:/website/convert/file/st34dm81hgmvqm3i3v5qk56zkeldkdeb/maps/ko00630.html)

3[EVM002492](file:///c:/website/convert/file/st34dm81hgmvqm3i3v5qk56zkeldkdeb/maps/ko00300.html)[https:\\www.genome.jp\dbget-bin\www_bget?map00300](file:///c:/website/convert/file/st34dm81hgmvqm3i3v5qk56zkeldkdeb/maps/ko00300.html)

5[EVM005157](file:///c:/website/convert/file/st34dm81hgmvqm3i3v5qk56zkeldkdeb/maps/ko00903.html)[https:\\www.genome.jp\dbget-bin\www_bget?map00903](file:///c:/website/convert/file/st34dm81hgmvqm3i3v5qk56zkeldkdeb/maps/ko00903.html)

45[EVM005818](file:///c:/website/convert/file/st34dm81hgmvqm3i3v5qk56zkeldkdeb/maps/ko00970.html)[https:\\www.genome.jp\dbget-bin\www_bget?map00970](file:///c:/website/convert/file/st34dm81hgmvqm3i3v5qk56zkeldkdeb/maps/ko00970.html)

7[EVM005223](file:///c:/website/convert/file/st34dm81hgmvqm3i3v5qk56zkeldkdeb/maps/ko00660.html)[https:\\www.genome.jp\dbget-bin\www_bget?map00660](file:///c:/website/convert/file/st34dm81hgmvqm3i3v5qk56zkeldkdeb/maps/ko00660.html)

27[EVM000883](file:///c:/website/convert/file/st34dm81hgmvqm3i3v5qk56zkeldkdeb/maps/ko00640.html)[https:\\www.genome.jp\dbget-bin\www_bget?map00640](file:///c:/website/convert/file/st34dm81hgmvqm3i3v5qk56zkeldkdeb/maps/ko00640.html)

799[EVM002015](file:///c:/website/convert/file/st34dm81hgmvqm3i3v5qk56zkeldkdeb/maps/ko01110.html)[https:\\www.genome.jp\dbget-bin\www_bget?map01110](file:///c:/website/convert/file/st34dm81hgmvqm3i3v5qk56zkeldkdeb/maps/ko01110.html)

63[EVM004208](file:///c:/website/convert/file/st34dm81hgmvqm3i3v5qk56zkeldkdeb/maps/ko01250.html)[https:\\www.genome.jp\dbget-bin\www_bget?map01250](file:///c:/website/convert/file/st34dm81hgmvqm3i3v5qk56zkeldkdeb/maps/ko01250.html)

75[EVM002015](file:///c:/website/convert/file/st34dm81hgmvqm3i3v5qk56zkeldkdeb/maps/ko00620.html)[https:\\www.genome.jp\dbget-bin\www_bget?map00620](file:///c:/website/convert/file/st34dm81hgmvqm3i3v5qk56zkeldkdeb/maps/ko00620.html)

[novel.322](file:///c:/website/convert/file/st34dm81hgmvqm3i3v5qk56zkeldkdeb/maps/ko00020.html)

[EVM002636](file:///c:/website/convert/file/st34dm81hgmvqm3i3v5qk56zkeldkdeb/maps/ko00730.html)

[EVM001124](file:///c:/website/convert/file/st34dm81hgmvqm3i3v5qk56zkeldkdeb/maps/ko00592.html)

[EVM006373](file:///c:/website/convert/file/st34dm81hgmvqm3i3v5qk56zkeldkdeb/maps/ko00590.html)

[EVM000395](file:///c:/website/convert/file/st34dm81hgmvqm3i3v5qk56zkeldkdeb/maps/ko00999.html)

[EVM004293](file:///c:/website/convert/file/st34dm81hgmvqm3i3v5qk56zkeldkdeb/maps/ko00260.html)

[EVM000433](file:///c:/website/convert/file/st34dm81hgmvqm3i3v5qk56zkeldkdeb/maps/ko03022.html)

[EVM006576](file:///c:/website/convert/file/st34dm81hgmvqm3i3v5qk56zkeldkdeb/maps/ko01230.html)

[EVM000678](file:///c:/website/convert/file/st34dm81hgmvqm3i3v5qk56zkeldkdeb/maps/ko00500.html)

[EVM002110](file:///c:/website/convert/file/st34dm81hgmvqm3i3v5qk56zkeldkdeb/maps/ko01040.html)

[EVM003714](file:///c:/website/convert/file/st34dm81hgmvqm3i3v5qk56zkeldkdeb/maps/ko00564.html)

[EVM002385](file:///c:/website/convert/file/st34dm81hgmvqm3i3v5qk56zkeldkdeb/maps/ko00785.html)

[EVM006516](file:///c:/website/convert/file/st34dm81hgmvqm3i3v5qk56zkeldkdeb/maps/ko00942.html)

[EVM000889](file:///c:/website/convert/file/st34dm81hgmvqm3i3v5qk56zkeldkdeb/maps/ko00760.html)

[novel.322](file:///c:/website/convert/file/st34dm81hgmvqm3i3v5qk56zkeldkdeb/maps/ko00630.html)

[EVM002531](file:///c:/website/convert/file/st34dm81hgmvqm3i3v5qk56zkeldkdeb/maps/ko00300.html)

[EVM004566](file:///c:/website/convert/file/st34dm81hgmvqm3i3v5qk56zkeldkdeb/maps/ko00903.html)

[EVM004618](file:///c:/website/convert/file/st34dm81hgmvqm3i3v5qk56zkeldkdeb/maps/ko00970.html)

[EVM001931](file:///c:/website/convert/file/st34dm81hgmvqm3i3v5qk56zkeldkdeb/maps/ko00660.html)

[EVM003676](file:///c:/website/convert/file/st34dm81hgmvqm3i3v5qk56zkeldkdeb/maps/ko00640.html)

[EVM000873](file:///c:/website/convert/file/st34dm81hgmvqm3i3v5qk56zkeldkdeb/maps/ko01110.html)

[EVM005745](file:///c:/website/convert/file/st34dm81hgmvqm3i3v5qk56zkeldkdeb/maps/ko01250.html)

[EVM000873](file:///c:/website/convert/file/st34dm81hgmvqm3i3v5qk56zkeldkdeb/maps/ko00620.html)

 

[EVM004566](file:///c:/website/convert/file/st34dm81hgmvqm3i3v5qk56zkeldkdeb/maps/ko00330.html)

[EVM002091](file:///c:/website/convert/file/st34dm81hgmvqm3i3v5qk56zkeldkdeb/maps/ko04070.html)

[EVM000288](file:///c:/website/convert/file/st34dm81hgmvqm3i3v5qk56zkeldkdeb/maps/ko03450.html)

[EVM003896](file:///c:/website/convert/file/st34dm81hgmvqm3i3v5qk56zkeldkdeb/maps/ko00563.html)

[EVM002091](file:///c:/website/convert/file/st34dm81hgmvqm3i3v5qk56zkeldkdeb/maps/ko04144.html)

[EVM004481](file:///c:/website/convert/file/st34dm81hgmvqm3i3v5qk56zkeldkdeb/maps/ko00902.html)

[EVM005189](file:///c:/website/convert/file/st34dm81hgmvqm3i3v5qk56zkeldkdeb/maps/ko00860.html)

[EVM003050](file:///c:/website/convert/file/st34dm81hgmvqm3i3v5qk56zkeldkdeb/maps/ko00906.html)

[EVM003429](file:///c:/website/convert/file/st34dm81hgmvqm3i3v5qk56zkeldkdeb/maps/ko00280.html)

[EVM004566](file:///c:/website/convert/file/st34dm81hgmvqm3i3v5qk56zkeldkdeb/maps/ko00410.html)

EVM003118

[EVM003973](file:///c:/website/convert/file/st34dm81hgmvqm3i3v5qk56zkeldkdeb/maps/ko04145.html)

[EVM003589](file:///c:/website/convert/file/st34dm81hgmvqm3i3v5qk56zkeldkdeb/maps/ko02010.html)

[novel.122](file:///c:/website/convert/file/st34dm81hgmvqm3i3v5qk56zkeldkdeb/maps/ko00130.html)

[EVM002980](file:///c:/website/convert/file/st34dm81hgmvqm3i3v5qk56zkeldkdeb/maps/ko03010.html)

[EVM004050](file:///c:/website/convert/file/st34dm81hgmvqm3i3v5qk56zkeldkdeb/maps/ko03420.html)

[EVM006576](file:///c:/website/convert/file/st34dm81hgmvqm3i3v5qk56zkeldkdeb/maps/ko00340.html)

[EVM000873](file:///c:/website/convert/file/st34dm81hgmvqm3i3v5qk56zkeldkdeb/maps/ko00071.html)

[EVM001652](file:///c:/website/convert/file/st34dm81hgmvqm3i3v5qk56zkeldkdeb/maps/ko00670.html)

[EVM005745](file:///c:/website/convert/file/st34dm81hgmvqm3i3v5qk56zkeldkdeb/maps/ko00520.html)

[EVM000102](file:///c:/website/convert/file/st34dm81hgmvqm3i3v5qk56zkeldkdeb/maps/ko00780.html)

[EVM004648](file:///c:/website/convert/file/st34dm81hgmvqm3i3v5qk56zkeldkdeb/maps/ko00562.html)

[EVM004566](file:///c:/website/convert/file/st34dm81hgmvqm3i3v5qk56zkeldkdeb/maps/ko00561.html)

[EVM003385](file:///c:/website/convert/file/st34dm81hgmvqm3i3v5qk56zkeldkdeb/maps/ko00650.html)

[EVM001663](file:///c:/website/convert/file/st34dm81hgmvqm3i3v5qk56zkeldkdeb/maps/ko03060.html)

EVM001764

[EVM000554](file:///c:/website/convert/file/st34dm81hgmvqm3i3v5qk56zkeldkdeb/maps/ko00944.html)

[EVM001184](file:///c:/website/convert/file/st34dm81hgmvqm3i3v5qk56zkeldkdeb/maps/ko03013.html)

EVM002787

[EVM000595](file:///c:/website/convert/file/st34dm81hgmvqm3i3v5qk56zkeldkdeb/maps/ko00430.html)

[EVM004203](file:///c:/website/convert/file/st34dm81hgmvqm3i3v5qk56zkeldkdeb/maps/ko00450.html)

[EVM001576](file:///c:/website/convert/file/st34dm81hgmvqm3i3v5qk56zkeldkdeb/maps/ko00100.html)

[EVM000102](file:///c:/website/convert/file/st34dm81hgmvqm3i3v5qk56zkeldkdeb/maps/ko01212.html)

[EVM004935](file:///c:/website/convert/file/st34dm81hgmvqm3i3v5qk56zkeldkdeb/maps/ko04626.html)

[EVM004944](file:///c:/website/convert/file/st34dm81hgmvqm3i3v5qk56zkeldkdeb/maps/ko00250.html)

[EVM002669](file:///c:/website/convert/file/st34dm81hgmvqm3i3v5qk56zkeldkdeb/maps/ko04712.html)

EVM002500

[EVM001298](file:///c:/website/convert/file/st34dm81hgmvqm3i3v5qk56zkeldkdeb/maps/ko00740.html)

--

28[EVM005157](file:///c:/website/convert/file/st34dm81hgmvqm3i3v5qk56zkeldkdeb/maps/ko00330.html)[https:\\www.genome.jp\dbget-bin\www_bget?map00330](file:///c:/website/convert/file/st34dm81hgmvqm3i3v5qk56zkeldkdeb/maps/ko00330.html)

40[EVM005167](file:///c:/website/convert/file/st34dm81hgmvqm3i3v5qk56zkeldkdeb/maps/ko04070.html)[https:\\www.genome.jp\dbget-bin\www_bget?map04070](file:///c:/website/convert/file/st34dm81hgmvqm3i3v5qk56zkeldkdeb/maps/ko04070.html)

4[EVM002655](file:///c:/website/convert/file/st34dm81hgmvqm3i3v5qk56zkeldkdeb/maps/ko03450.html)[https:\\www.genome.jp\dbget-bin\www_bget?map03450](file:///c:/website/convert/file/st34dm81hgmvqm3i3v5qk56zkeldkdeb/maps/ko03450.html)

9[novel.545](file:///c:/website/convert/file/st34dm81hgmvqm3i3v5qk56zkeldkdeb/maps/ko00563.html)[https:\\www.genome.jp\dbget-bin\www_bget?map00563](file:///c:/website/convert/file/st34dm81hgmvqm3i3v5qk56zkeldkdeb/maps/ko00563.html)

127[EVM005167](file:///c:/website/convert/file/st34dm81hgmvqm3i3v5qk56zkeldkdeb/maps/ko04144.html)[https:\\www.genome.jp\dbget-bin\www_bget?map04144](file:///c:/website/convert/file/st34dm81hgmvqm3i3v5qk56zkeldkdeb/maps/ko04144.html)

10[EVM004696](file:///c:/website/convert/file/st34dm81hgmvqm3i3v5qk56zkeldkdeb/maps/ko00902.html)[https:\\www.genome.jp\dbget-bin\www_bget?map00902](file:///c:/website/convert/file/st34dm81hgmvqm3i3v5qk56zkeldkdeb/maps/ko00902.html)

17[EVM005927](file:///c:/website/convert/file/st34dm81hgmvqm3i3v5qk56zkeldkdeb/maps/ko00860.html)[https:\\www.genome.jp\dbget-bin\www_bget?map00860](file:///c:/website/convert/file/st34dm81hgmvqm3i3v5qk56zkeldkdeb/maps/ko00860.html)

20[EVM003633](file:///c:/website/convert/file/st34dm81hgmvqm3i3v5qk56zkeldkdeb/maps/ko00906.html)[https:\\www.genome.jp\dbget-bin\www_bget?map00906](file:///c:/website/convert/file/st34dm81hgmvqm3i3v5qk56zkeldkdeb/maps/ko00906.html)

35[EVM003149](file:///c:/website/convert/file/st34dm81hgmvqm3i3v5qk56zkeldkdeb/maps/ko00280.html)[https:\\www.genome.jp\dbget-bin\www_bget?map00280](file:///c:/website/convert/file/st34dm81hgmvqm3i3v5qk56zkeldkdeb/maps/ko00280.html)

21[EVM005157](file:///c:/website/convert/file/st34dm81hgmvqm3i3v5qk56zkeldkdeb/maps/ko00410.html)[https:\\www.genome.jp\dbget-bin\www_bget?map00410](file:///c:/website/convert/file/st34dm81hgmvqm3i3v5qk56zkeldkdeb/maps/ko00410.html)

5[EVM005665](file:///c:/website/convert/file/st34dm81hgmvqm3i3v5qk56zkeldkdeb/maps/ko00440.html)[https:\\www.genome.jp\dbget-bin\www_bget?map00440](file:///c:/website/convert/file/st34dm81hgmvqm3i3v5qk56zkeldkdeb/maps/ko00440.html)

48[EVM000409](file:///c:/website/convert/file/st34dm81hgmvqm3i3v5qk56zkeldkdeb/maps/ko04145.html)[https:\\www.genome.jp\dbget-bin\www_bget?map04145](file:///c:/website/convert/file/st34dm81hgmvqm3i3v5qk56zkeldkdeb/maps/ko04145.html)

40[novel.141](file:///c:/website/convert/file/st34dm81hgmvqm3i3v5qk56zkeldkdeb/maps/ko02010.html)[https:\\www.genome.jp\dbget-bin\www_bget?map02010](file:///c:/website/convert/file/st34dm81hgmvqm3i3v5qk56zkeldkdeb/maps/ko02010.html)

28[EVM000342](file:///c:/website/convert/file/st34dm81hgmvqm3i3v5qk56zkeldkdeb/maps/ko00130.html)[https:\\www.genome.jp\dbget-bin\www_bget?map00130](file:///c:/website/convert/file/st34dm81hgmvqm3i3v5qk56zkeldkdeb/maps/ko00130.html)

155[EVM005140](file:///c:/website/convert/file/st34dm81hgmvqm3i3v5qk56zkeldkdeb/maps/ko03010.html)[https:\\www.genome.jp\dbget-bin\www_bget?map03010](file:///c:/website/convert/file/st34dm81hgmvqm3i3v5qk56zkeldkdeb/maps/ko03010.html)

30[novel.580](file:///c:/website/convert/file/st34dm81hgmvqm3i3v5qk56zkeldkdeb/maps/ko03420.html)[https:\\www.genome.jp\dbget-bin\www_bget?map03420](file:///c:/website/convert/file/st34dm81hgmvqm3i3v5qk56zkeldkdeb/maps/ko03420.html)

10[EVM005157](file:///c:/website/convert/file/st34dm81hgmvqm3i3v5qk56zkeldkdeb/maps/ko00340.html)[https:\\www.genome.jp\dbget-bin\www_bget?map00340](file:///c:/website/convert/file/st34dm81hgmvqm3i3v5qk56zkeldkdeb/maps/ko00340.html)

24[EVM001586](file:///c:/website/convert/file/st34dm81hgmvqm3i3v5qk56zkeldkdeb/maps/ko00071.html)[https:\\www.genome.jp\dbget-bin\www_bget?map00071](file:///c:/website/convert/file/st34dm81hgmvqm3i3v5qk56zkeldkdeb/maps/ko00071.html)

13[EVM000426](file:///c:/website/convert/file/st34dm81hgmvqm3i3v5qk56zkeldkdeb/maps/ko00670.html)[https:\\www.genome.jp\dbget-bin\www_bget?map00670](file:///c:/website/convert/file/st34dm81hgmvqm3i3v5qk56zkeldkdeb/maps/ko00670.html)

90[EVM004208](file:///c:/website/convert/file/st34dm81hgmvqm3i3v5qk56zkeldkdeb/maps/ko00520.html)[https:\\www.genome.jp\dbget-bin\www_bget?map00520](file:///c:/website/convert/file/st34dm81hgmvqm3i3v5qk56zkeldkdeb/maps/ko00520.html)

11[EVM004121](file:///c:/website/convert/file/st34dm81hgmvqm3i3v5qk56zkeldkdeb/maps/ko00780.html)[https:\\www.genome.jp\dbget-bin\www_bget?map00780](file:///c:/website/convert/file/st34dm81hgmvqm3i3v5qk56zkeldkdeb/maps/ko00780.html)

39[EVM000883](file:///c:/website/convert/file/st34dm81hgmvqm3i3v5qk56zkeldkdeb/maps/ko00562.html)[https:\\www.genome.jp\dbget-bin\www_bget?map00562](file:///c:/website/convert/file/st34dm81hgmvqm3i3v5qk56zkeldkdeb/maps/ko00562.html)

56[EVM002015](file:///c:/website/convert/file/st34dm81hgmvqm3i3v5qk56zkeldkdeb/maps/ko00561.html)[https:\\www.genome.jp\dbget-bin\www_bget?map00561](file:///c:/website/convert/file/st34dm81hgmvqm3i3v5qk56zkeldkdeb/maps/ko00561.html)

11[EVM004486](file:///c:/website/convert/file/st34dm81hgmvqm3i3v5qk56zkeldkdeb/maps/ko00650.html)[https:\\www.genome.jp\dbget-bin\www_bget?map00650](file:///c:/website/convert/file/st34dm81hgmvqm3i3v5qk56zkeldkdeb/maps/ko00650.html)

33[novel.441](file:///c:/website/convert/file/st34dm81hgmvqm3i3v5qk56zkeldkdeb/maps/ko03060.html)[https:\\www.genome.jp\dbget-bin\www_bget?map03060](file:///c:/website/convert/file/st34dm81hgmvqm3i3v5qk56zkeldkdeb/maps/ko03060.html)

6[EVM000072](file:///c:/website/convert/file/st34dm81hgmvqm3i3v5qk56zkeldkdeb/maps/ko04122.html)[https:\\www.genome.jp\dbget-bin\www_bget?map04122](file:///c:/website/convert/file/st34dm81hgmvqm3i3v5qk56zkeldkdeb/maps/ko04122.html)

9[EVM001507](file:///c:/website/convert/file/st34dm81hgmvqm3i3v5qk56zkeldkdeb/maps/ko00944.html)[https:\\www.genome.jp\dbget-bin\www_bget?map00944](file:///c:/website/convert/file/st34dm81hgmvqm3i3v5qk56zkeldkdeb/maps/ko00944.html)

103[EVM005173](file:///c:/website/convert/file/st34dm81hgmvqm3i3v5qk56zkeldkdeb/maps/ko03013.html)[https:\\www.genome.jp\dbget-bin\www_bget?map03013](file:///c:/website/convert/file/st34dm81hgmvqm3i3v5qk56zkeldkdeb/maps/ko03013.html)

7[EVM004969](file:///c:/website/convert/file/st34dm81hgmvqm3i3v5qk56zkeldkdeb/maps/ko00966.html)[https:\\www.genome.jp\dbget-bin\www_bget?map00966](file:///c:/website/convert/file/st34dm81hgmvqm3i3v5qk56zkeldkdeb/maps/ko00966.html)

1[EVM004859](file:///c:/website/convert/file/st34dm81hgmvqm3i3v5qk56zkeldkdeb/maps/ko00430.html)[https:\\www.genome.jp\dbget-bin\www_bget?map00430](file:///c:/website/convert/file/st34dm81hgmvqm3i3v5qk56zkeldkdeb/maps/ko00430.html)

20[EVM005874](file:///c:/website/convert/file/st34dm81hgmvqm3i3v5qk56zkeldkdeb/maps/ko00450.html)[https:\\www.genome.jp\dbget-bin\www_bget?map00450](file:///c:/website/convert/file/st34dm81hgmvqm3i3v5qk56zkeldkdeb/maps/ko00450.html)

13[EVM005372](file:///c:/website/convert/file/st34dm81hgmvqm3i3v5qk56zkeldkdeb/maps/ko00100.html)[https:\\www.genome.jp\dbget-bin\www_bget?map00100](file:///c:/website/convert/file/st34dm81hgmvqm3i3v5qk56zkeldkdeb/maps/ko00100.html)

57[EVM004121](file:///c:/website/convert/file/st34dm81hgmvqm3i3v5qk56zkeldkdeb/maps/ko01212.html)[https:\\www.genome.jp\dbget-bin\www_bget?map01212](file:///c:/website/convert/file/st34dm81hgmvqm3i3v5qk56zkeldkdeb/maps/ko01212.html)

202[EVM004954](file:///c:/website/convert/file/st34dm81hgmvqm3i3v5qk56zkeldkdeb/maps/ko04626.html)[https:\\www.genome.jp\dbget-bin\www_bget?map04626](file:///c:/website/convert/file/st34dm81hgmvqm3i3v5qk56zkeldkdeb/maps/ko04626.html)

20[EVM005693](file:///c:/website/convert/file/st34dm81hgmvqm3i3v5qk56zkeldkdeb/maps/ko00250.html)[https:\\www.genome.jp\dbget-bin\www_bget?map00250](file:///c:/website/convert/file/st34dm81hgmvqm3i3v5qk56zkeldkdeb/maps/ko00250.html)

30[EVM000442](file:///c:/website/convert/file/st34dm81hgmvqm3i3v5qk56zkeldkdeb/maps/ko04712.html)[https:\\www.genome.jp\dbget-bin\www_bget?map04712](file:///c:/website/convert/file/st34dm81hgmvqm3i3v5qk56zkeldkdeb/maps/ko04712.html)

7[EVM006167](file:///c:/website/convert/file/st34dm81hgmvqm3i3v5qk56zkeldkdeb/maps/ko00402.html)[https:\\www.genome.jp\dbget-bin\www_bget?map00402](file:///c:/website/convert/file/st34dm81hgmvqm3i3v5qk56zkeldkdeb/maps/ko00402.html)

10[EVM000032](file:///c:/website/convert/file/st34dm81hgmvqm3i3v5qk56zkeldkdeb/maps/ko00740.html)[https:\\www.genome.jp\dbget-bin\www_bget?map00740](file:///c:/website/convert/file/st34dm81hgmvqm3i3v5qk56zkeldkdeb/maps/ko00740.html)

1[EVM005397](file:///c:/website/convert/file/st34dm81hgmvqm3i3v5qk56zkeldkdeb/maps/ko00232.html)[https:\\www.genome.jp\dbget-bin\www_bget?map00232](file:///c:/website/convert/file/st34dm81hgmvqm3i3v5qk56zkeldkdeb/maps/ko00232.html)

172[EVM002226](file:///c:/website/convert/file/st34dm81hgmvqm3i3v5qk56zkeldkdeb/maps/ko04075.html)[https:\\www.genome.jp\dbget-bin\www_bget?map04075](file:///c:/website/convert/file/st34dm81hgmvqm3i3v5qk56zkeldkdeb/maps/ko04075.html)

[EVM004098](file:///c:/website/convert/file/st34dm81hgmvqm3i3v5qk56zkeldkdeb/maps/ko04075.html)

[EVM002761](file:///c:/website/convert/file/st34dm81hgmvqm3i3v5qk56zkeldkdeb/maps/ko00965.html)

[novel.322](file:///c:/website/convert/file/st34dm81hgmvqm3i3v5qk56zkeldkdeb/maps/ko01200.html)

[EVM000906](file:///c:/website/convert/file/st34dm81hgmvqm3i3v5qk56zkeldkdeb/maps/ko03250.html)

--

0 --

[https:\\www.genome.jp\dbget-bin\www_bget?map00965](file:///c:/website/convert/file/st34dm81hgmvqm3i3v5qk56zkeldkdeb/maps/ko00965.html)

172[EVM005340](file:///c:/website/convert/file/st34dm81hgmvqm3i3v5qk56zkeldkdeb/maps/ko01200.html)[https:\\www.genome.jp\dbget-bin\www_bget?map01200](file:///c:/website/convert/file/st34dm81hgmvqm3i3v5qk56zkeldkdeb/maps/ko01200.html)

22[EVM000344](file:///c:/website/convert/file/st34dm81hgmvqm3i3v5qk56zkeldkdeb/maps/ko03250.html)[https:\\www.genome.jp\dbget-bin\www_bget?map03250](file:///c:/website/convert/file/st34dm81hgmvqm3i3v5qk56zkeldkdeb/maps/ko03250.html)

1[EVM005896](file:///c:/website/convert/file/st34dm81hgmvqm3i3v5qk56zkeldkdeb/maps/ko00523.html)[https:\\www.genome.jp\dbget-bin\www_bget?map00523](file:///c:/website/convert/file/st34dm81hgmvqm3i3v5qk56zkeldkdeb/maps/ko00523.html)

64[EVM004734](file:///c:/website/convert/file/st34dm81hgmvqm3i3v5qk56zkeldkdeb/maps/ko03018.html)[https:\\www.genome.jp\dbget-bin\www_bget?map03018](file:///c:/website/convert/file/st34dm81hgmvqm3i3v5qk56zkeldkdeb/maps/ko03018.html)

19[EVM005157](file:///c:/website/convert/file/st34dm81hgmvqm3i3v5qk56zkeldkdeb/maps/ko00310.html)[https:\\www.genome.jp\dbget-bin\www_bget?map00310](file:///c:/website/convert/file/st34dm81hgmvqm3i3v5qk56zkeldkdeb/maps/ko00310.html)

[EVM004193](file:///c:/website/convert/file/st34dm81hgmvqm3i3v5qk56zkeldkdeb/maps/ko03018.html)

[EVM004566](file:///c:/website/convert/file/st34dm81hgmvqm3i3v5qk56zkeldkdeb/maps/ko00310.html)

 

[EVM001258](file:///c:/website/convert/file/st34dm81hgmvqm3i3v5qk56zkeldkdeb/maps/ko00524.html)

[EVM003527](file:///c:/website/convert/file/st34dm81hgmvqm3i3v5qk56zkeldkdeb/maps/ko00511.html)

[EVM006182](file:///c:/website/convert/file/st34dm81hgmvqm3i3v5qk56zkeldkdeb/maps/ko00790.html)

[EVM002761](file:///c:/website/convert/file/st34dm81hgmvqm3i3v5qk56zkeldkdeb/maps/ko00901.html)

[EVM004566](file:///c:/website/convert/file/st34dm81hgmvqm3i3v5qk56zkeldkdeb/maps/ko00380.html)

[EVM002393](file:///c:/website/convert/file/st34dm81hgmvqm3i3v5qk56zkeldkdeb/maps/ko03430.html)

[EVM000053](file:///c:/website/convert/file/st34dm81hgmvqm3i3v5qk56zkeldkdeb/maps/ko00905.html)

[EVM005655](file:///c:/website/convert/file/st34dm81hgmvqm3i3v5qk56zkeldkdeb/maps/ko00240.html)

[EVM006390](file:///c:/website/convert/file/st34dm81hgmvqm3i3v5qk56zkeldkdeb/maps/ko04016.html)

[EVM005745](file:///c:/website/convert/file/st34dm81hgmvqm3i3v5qk56zkeldkdeb/maps/ko00053.html)

--

3[EVM001219](file:///c:/website/convert/file/st34dm81hgmvqm3i3v5qk56zkeldkdeb/maps/ko00524.html)[https:\\www.genome.jp\dbget-bin\www_bget?map00524](file:///c:/website/convert/file/st34dm81hgmvqm3i3v5qk56zkeldkdeb/maps/ko00524.html)

35[EVM000988](file:///c:/website/convert/file/st34dm81hgmvqm3i3v5qk56zkeldkdeb/maps/ko00511.html)[https:\\www.genome.jp\dbget-bin\www_bget?map00511](file:///c:/website/convert/file/st34dm81hgmvqm3i3v5qk56zkeldkdeb/maps/ko00511.html)

11[EVM005231](file:///c:/website/convert/file/st34dm81hgmvqm3i3v5qk56zkeldkdeb/maps/ko00790.html)[https:\\www.genome.jp\dbget-bin\www_bget?map00790](file:///c:/website/convert/file/st34dm81hgmvqm3i3v5qk56zkeldkdeb/maps/ko00790.html)

1[EVM006038](file:///c:/website/convert/file/st34dm81hgmvqm3i3v5qk56zkeldkdeb/maps/ko00901.html)[https:\\www.genome.jp\dbget-bin\www_bget?map00901](file:///c:/website/convert/file/st34dm81hgmvqm3i3v5qk56zkeldkdeb/maps/ko00901.html)

19[EVM005157](file:///c:/website/convert/file/st34dm81hgmvqm3i3v5qk56zkeldkdeb/maps/ko00380.html)[https:\\www.genome.jp\dbget-bin\www_bget?map00380](file:///c:/website/convert/file/st34dm81hgmvqm3i3v5qk56zkeldkdeb/maps/ko00380.html)

21[EVM004932](file:///c:/website/convert/file/st34dm81hgmvqm3i3v5qk56zkeldkdeb/maps/ko03430.html)[https:\\www.genome.jp\dbget-bin\www_bget?map03430](file:///c:/website/convert/file/st34dm81hgmvqm3i3v5qk56zkeldkdeb/maps/ko03430.html)

5[EVM001020](file:///c:/website/convert/file/st34dm81hgmvqm3i3v5qk56zkeldkdeb/maps/ko00905.html)[https:\\www.genome.jp\dbget-bin\www_bget?map00905](file:///c:/website/convert/file/st34dm81hgmvqm3i3v5qk56zkeldkdeb/maps/ko00905.html)

26[EVM001722](file:///c:/website/convert/file/st34dm81hgmvqm3i3v5qk56zkeldkdeb/maps/ko00240.html)[https:\\www.genome.jp\dbget-bin\www_bget?map00240](file:///c:/website/convert/file/st34dm81hgmvqm3i3v5qk56zkeldkdeb/maps/ko00240.html)

135[EVM004196](file:///c:/website/convert/file/st34dm81hgmvqm3i3v5qk56zkeldkdeb/maps/ko04016.html)[https:\\www.genome.jp\dbget-bin\www_bget?map04016](file:///c:/website/convert/file/st34dm81hgmvqm3i3v5qk56zkeldkdeb/maps/ko04016.html)

45[EVM002015](file:///c:/website/convert/file/st34dm81hgmvqm3i3v5qk56zkeldkdeb/maps/ko00053.html)[https:\\www.genome.jp\dbget-bin\www_bget?map00053](file:///c:/website/convert/file/st34dm81hgmvqm3i3v5qk56zkeldkdeb/maps/ko00053.html)

1[EVM004328](file:///c:/website/convert/file/st34dm81hgmvqm3i3v5qk56zkeldkdeb/maps/ko00601.html)[https:\\www.genome.jp\dbget-bin\www_bget?map00601](file:///c:/website/convert/file/st34dm81hgmvqm3i3v5qk56zkeldkdeb/maps/ko00601.html)

[EVM000441](file:///c:/website/convert/file/st34dm81hgmvqm3i3v5qk56zkeldkdeb/maps/ko00470.html)

[EVM004388](file:///c:/website/convert/file/st34dm81hgmvqm3i3v5qk56zkeldkdeb/maps/ko00600.html)

[EVM006246](file:///c:/website/convert/file/st34dm81hgmvqm3i3v5qk56zkeldkdeb/maps/ko03440.html)

[EVM000873](file:///c:/website/convert/file/st34dm81hgmvqm3i3v5qk56zkeldkdeb/maps/ko00350.html)

[EVM005604](file:///c:/website/convert/file/st34dm81hgmvqm3i3v5qk56zkeldkdeb/maps/ko00603.html)

[EVM000311](file:///c:/website/convert/file/st34dm81hgmvqm3i3v5qk56zkeldkdeb/maps/ko01232.html)

[EVM000102](file:///c:/website/convert/file/st34dm81hgmvqm3i3v5qk56zkeldkdeb/maps/ko00061.html)

[EVM003230](file:///c:/website/convert/file/st34dm81hgmvqm3i3v5qk56zkeldkdeb/maps/ko00400.html)

[EVM000873](file:///c:/website/convert/file/st34dm81hgmvqm3i3v5qk56zkeldkdeb/maps/ko00010.html)

[EVM003102](file:///c:/website/convert/file/st34dm81hgmvqm3i3v5qk56zkeldkdeb/maps/ko00908.html)

[EVM003519](file:///c:/website/convert/file/st34dm81hgmvqm3i3v5qk56zkeldkdeb/maps/ko04130.html)

[EVM001258](file:///c:/website/convert/file/st34dm81hgmvqm3i3v5qk56zkeldkdeb/maps/ko00052.html)

[EVM004293](file:///c:/website/convert/file/st34dm81hgmvqm3i3v5qk56zkeldkdeb/maps/ko00360.html)

[EVM000247](file:///c:/website/convert/file/st34dm81hgmvqm3i3v5qk56zkeldkdeb/maps/ko00531.html)

[EVM004944](file:///c:/website/convert/file/st34dm81hgmvqm3i3v5qk56zkeldkdeb/maps/ko00910.html)

[EVM001258](file:///c:/website/convert/file/st34dm81hgmvqm3i3v5qk56zkeldkdeb/maps/ko00051.html)

[EVM000873](file:///c:/website/convert/file/st34dm81hgmvqm3i3v5qk56zkeldkdeb/maps/ko01100.html)

[EVM002238](file:///c:/website/convert/file/st34dm81hgmvqm3i3v5qk56zkeldkdeb/maps/ko00940.html)

[EVM006263](file:///c:/website/convert/file/st34dm81hgmvqm3i3v5qk56zkeldkdeb/maps/ko00604.html)

[EVM004722](file:///c:/website/convert/file/st34dm81hgmvqm3i3v5qk56zkeldkdeb/maps/ko00943.html)

[EVM001652](file:///c:/website/convert/file/st34dm81hgmvqm3i3v5qk56zkeldkdeb/maps/ko00460.html)

[EVM001169](file:///c:/website/convert/file/st34dm81hgmvqm3i3v5qk56zkeldkdeb/maps/ko00073.html)

[EVM000455](file:///c:/website/convert/file/st34dm81hgmvqm3i3v5qk56zkeldkdeb/maps/ko00591.html)

[EVM004293](file:///c:/website/convert/file/st34dm81hgmvqm3i3v5qk56zkeldkdeb/maps/ko00950.html)

[novel.122](file:///c:/website/convert/file/st34dm81hgmvqm3i3v5qk56zkeldkdeb/maps/ko00945.html)

[EVM003456](file:///c:/website/convert/file/st34dm81hgmvqm3i3v5qk56zkeldkdeb/maps/ko00510.html)

[EVM000905](file:///c:/website/convert/file/st34dm81hgmvqm3i3v5qk56zkeldkdeb/maps/ko00062.html)

[EVM004643](file:///c:/website/convert/file/st34dm81hgmvqm3i3v5qk56zkeldkdeb/maps/ko03015.html)

EVM000522

[EVM006390](file:///c:/website/convert/file/st34dm81hgmvqm3i3v5qk56zkeldkdeb/maps/ko00513.html)

[novel.122](file:///c:/website/convert/file/st34dm81hgmvqm3i3v5qk56zkeldkdeb/maps/ko00941.html)

[EVM004780](file:///c:/website/convert/file/st34dm81hgmvqm3i3v5qk56zkeldkdeb/maps/ko00030.html)

[novel.322](file:///c:/website/convert/file/st34dm81hgmvqm3i3v5qk56zkeldkdeb/maps/ko00710.html)

[EVM003676](file:///c:/website/convert/file/st34dm81hgmvqm3i3v5qk56zkeldkdeb/maps/ko00904.html)

[EVM002358](file:///c:/website/convert/file/st34dm81hgmvqm3i3v5qk56zkeldkdeb/maps/ko03020.html)

0 --

[https:\\www.genome.jp\dbget-bin\www_bget?map00470](file:///c:/website/convert/file/st34dm81hgmvqm3i3v5qk56zkeldkdeb/maps/ko00470.html)

30[EVM006499](file:///c:/website/convert/file/st34dm81hgmvqm3i3v5qk56zkeldkdeb/maps/ko00600.html)[https:\\www.genome.jp\dbget-bin\www_bget?map00600](file:///c:/website/convert/file/st34dm81hgmvqm3i3v5qk56zkeldkdeb/maps/ko00600.html)

21[EVM004932](file:///c:/website/convert/file/st34dm81hgmvqm3i3v5qk56zkeldkdeb/maps/ko03440.html)[https:\\www.genome.jp\dbget-bin\www_bget?map03440](file:///c:/website/convert/file/st34dm81hgmvqm3i3v5qk56zkeldkdeb/maps/ko03440.html)

20[EVM001586](file:///c:/website/convert/file/st34dm81hgmvqm3i3v5qk56zkeldkdeb/maps/ko00350.html)[https:\\www.genome.jp\dbget-bin\www_bget?map00350](file:///c:/website/convert/file/st34dm81hgmvqm3i3v5qk56zkeldkdeb/maps/ko00350.html)

3[EVM004328](file:///c:/website/convert/file/st34dm81hgmvqm3i3v5qk56zkeldkdeb/maps/ko00603.html)[https:\\www.genome.jp\dbget-bin\www_bget?map00603](file:///c:/website/convert/file/st34dm81hgmvqm3i3v5qk56zkeldkdeb/maps/ko00603.html)

32[EVM004707](file:///c:/website/convert/file/st34dm81hgmvqm3i3v5qk56zkeldkdeb/maps/ko01232.html)[https:\\www.genome.jp\dbget-bin\www_bget?map01232](file:///c:/website/convert/file/st34dm81hgmvqm3i3v5qk56zkeldkdeb/maps/ko01232.html)

30[EVM004121](file:///c:/website/convert/file/st34dm81hgmvqm3i3v5qk56zkeldkdeb/maps/ko00061.html)[https:\\www.genome.jp\dbget-bin\www_bget?map00061](file:///c:/website/convert/file/st34dm81hgmvqm3i3v5qk56zkeldkdeb/maps/ko00061.html)

22[EVM001837](file:///c:/website/convert/file/st34dm81hgmvqm3i3v5qk56zkeldkdeb/maps/ko00400.html)[https:\\www.genome.jp\dbget-bin\www_bget?map00400](file:///c:/website/convert/file/st34dm81hgmvqm3i3v5qk56zkeldkdeb/maps/ko00400.html)

96[EVM002015](file:///c:/website/convert/file/st34dm81hgmvqm3i3v5qk56zkeldkdeb/maps/ko00010.html)[https:\\www.genome.jp\dbget-bin\www_bget?map00010](file:///c:/website/convert/file/st34dm81hgmvqm3i3v5qk56zkeldkdeb/maps/ko00010.html)

12[EVM005783](file:///c:/website/convert/file/st34dm81hgmvqm3i3v5qk56zkeldkdeb/maps/ko00908.html)[https:\\www.genome.jp\dbget-bin\www_bget?map00908](file:///c:/website/convert/file/st34dm81hgmvqm3i3v5qk56zkeldkdeb/maps/ko00908.html)

13[EVM004538](file:///c:/website/convert/file/st34dm81hgmvqm3i3v5qk56zkeldkdeb/maps/ko04130.html)[https:\\www.genome.jp\dbget-bin\www_bget?map04130](file:///c:/website/convert/file/st34dm81hgmvqm3i3v5qk56zkeldkdeb/maps/ko04130.html)

45[EVM001219](file:///c:/website/convert/file/st34dm81hgmvqm3i3v5qk56zkeldkdeb/maps/ko00052.html)[https:\\www.genome.jp\dbget-bin\www_bget?map00052](file:///c:/website/convert/file/st34dm81hgmvqm3i3v5qk56zkeldkdeb/maps/ko00052.html)

17[EVM004486](file:///c:/website/convert/file/st34dm81hgmvqm3i3v5qk56zkeldkdeb/maps/ko00360.html)[https:\\www.genome.jp\dbget-bin\www_bget?map00360](file:///c:/website/convert/file/st34dm81hgmvqm3i3v5qk56zkeldkdeb/maps/ko00360.html)

17[EVM001261](file:///c:/website/convert/file/st34dm81hgmvqm3i3v5qk56zkeldkdeb/maps/ko00531.html)[https:\\www.genome.jp\dbget-bin\www_bget?map00531](file:///c:/website/convert/file/st34dm81hgmvqm3i3v5qk56zkeldkdeb/maps/ko00531.html)

14[EVM005693](file:///c:/website/convert/file/st34dm81hgmvqm3i3v5qk56zkeldkdeb/maps/ko00910.html)[https:\\www.genome.jp\dbget-bin\www_bget?map00910](file:///c:/website/convert/file/st34dm81hgmvqm3i3v5qk56zkeldkdeb/maps/ko00910.html)

36[EVM004755](file:///c:/website/convert/file/st34dm81hgmvqm3i3v5qk56zkeldkdeb/maps/ko00051.html)[https:\\www.genome.jp\dbget-bin\www_bget?map00051](file:///c:/website/convert/file/st34dm81hgmvqm3i3v5qk56zkeldkdeb/maps/ko00051.html)

1363[EVM002015](file:///c:/website/convert/file/st34dm81hgmvqm3i3v5qk56zkeldkdeb/maps/ko01100.html)[https:\\www.genome.jp\dbget-bin\www_bget?map01100](file:///c:/website/convert/file/st34dm81hgmvqm3i3v5qk56zkeldkdeb/maps/ko01100.html)

76[EVM005557](file:///c:/website/convert/file/st34dm81hgmvqm3i3v5qk56zkeldkdeb/maps/ko00940.html)[https:\\www.genome.jp\dbget-bin\www_bget?map00940](file:///c:/website/convert/file/st34dm81hgmvqm3i3v5qk56zkeldkdeb/maps/ko00940.html)

9[EVM002323](file:///c:/website/convert/file/st34dm81hgmvqm3i3v5qk56zkeldkdeb/maps/ko00604.html)[https:\\www.genome.jp\dbget-bin\www_bget?map00604](file:///c:/website/convert/file/st34dm81hgmvqm3i3v5qk56zkeldkdeb/maps/ko00604.html)

9[EVM003731](file:///c:/website/convert/file/st34dm81hgmvqm3i3v5qk56zkeldkdeb/maps/ko00943.html)[https:\\www.genome.jp\dbget-bin\www_bget?map00943](file:///c:/website/convert/file/st34dm81hgmvqm3i3v5qk56zkeldkdeb/maps/ko00943.html)

29[EVM005314](file:///c:/website/convert/file/st34dm81hgmvqm3i3v5qk56zkeldkdeb/maps/ko00460.html)[https:\\www.genome.jp\dbget-bin\www_bget?map00460](file:///c:/website/convert/file/st34dm81hgmvqm3i3v5qk56zkeldkdeb/maps/ko00460.html)

9[EVM004646](file:///c:/website/convert/file/st34dm81hgmvqm3i3v5qk56zkeldkdeb/maps/ko00073.html)[https:\\www.genome.jp\dbget-bin\www_bget?map00073](file:///c:/website/convert/file/st34dm81hgmvqm3i3v5qk56zkeldkdeb/maps/ko00073.html)

4[EVM003911](file:///c:/website/convert/file/st34dm81hgmvqm3i3v5qk56zkeldkdeb/maps/ko00591.html)[https:\\www.genome.jp\dbget-bin\www_bget?map00591](file:///c:/website/convert/file/st34dm81hgmvqm3i3v5qk56zkeldkdeb/maps/ko00591.html)

16[EVM004400](file:///c:/website/convert/file/st34dm81hgmvqm3i3v5qk56zkeldkdeb/maps/ko00950.html)[https:\\www.genome.jp\dbget-bin\www_bget?map00950](file:///c:/website/convert/file/st34dm81hgmvqm3i3v5qk56zkeldkdeb/maps/ko00950.html)

13[EVM004011](file:///c:/website/convert/file/st34dm81hgmvqm3i3v5qk56zkeldkdeb/maps/ko00945.html)[https:\\www.genome.jp\dbget-bin\www_bget?map00945](file:///c:/website/convert/file/st34dm81hgmvqm3i3v5qk56zkeldkdeb/maps/ko00945.html)

11[EVM003097](file:///c:/website/convert/file/st34dm81hgmvqm3i3v5qk56zkeldkdeb/maps/ko00510.html)[https:\\www.genome.jp\dbget-bin\www_bget?map00510](file:///c:/website/convert/file/st34dm81hgmvqm3i3v5qk56zkeldkdeb/maps/ko00510.html)

10[EVM003033](file:///c:/website/convert/file/st34dm81hgmvqm3i3v5qk56zkeldkdeb/maps/ko00062.html)[https:\\www.genome.jp\dbget-bin\www_bget?map00062](file:///c:/website/convert/file/st34dm81hgmvqm3i3v5qk56zkeldkdeb/maps/ko00062.html)

70[EVM001253](file:///c:/website/convert/file/st34dm81hgmvqm3i3v5qk56zkeldkdeb/maps/ko03015.html)[https:\\www.genome.jp\dbget-bin\www_bget?map03015](file:///c:/website/convert/file/st34dm81hgmvqm3i3v5qk56zkeldkdeb/maps/ko03015.html)

2[EVM004310](file:///c:/website/convert/file/st34dm81hgmvqm3i3v5qk56zkeldkdeb/maps/ko00196.html)[https:\\www.genome.jp\dbget-bin\www_bget?map00196](file:///c:/website/convert/file/st34dm81hgmvqm3i3v5qk56zkeldkdeb/maps/ko00196.html)

13[EVM003097](file:///c:/website/convert/file/st34dm81hgmvqm3i3v5qk56zkeldkdeb/maps/ko00513.html)[https:\\www.genome.jp\dbget-bin\www_bget?map00513](file:///c:/website/convert/file/st34dm81hgmvqm3i3v5qk56zkeldkdeb/maps/ko00513.html)

19[EVM004011](file:///c:/website/convert/file/st34dm81hgmvqm3i3v5qk56zkeldkdeb/maps/ko00941.html)[https:\\www.genome.jp\dbget-bin\www_bget?map00941](file:///c:/website/convert/file/st34dm81hgmvqm3i3v5qk56zkeldkdeb/maps/ko00941.html)

38[EVM004504](file:///c:/website/convert/file/st34dm81hgmvqm3i3v5qk56zkeldkdeb/maps/ko00030.html)[https:\\www.genome.jp\dbget-bin\www_bget?map00030](file:///c:/website/convert/file/st34dm81hgmvqm3i3v5qk56zkeldkdeb/maps/ko00030.html)

38[EVM005340](file:///c:/website/convert/file/st34dm81hgmvqm3i3v5qk56zkeldkdeb/maps/ko00710.html)[https:\\www.genome.jp\dbget-bin\www_bget?map00710](file:///c:/website/convert/file/st34dm81hgmvqm3i3v5qk56zkeldkdeb/maps/ko00710.html)

7[EVM004553](file:///c:/website/convert/file/st34dm81hgmvqm3i3v5qk56zkeldkdeb/maps/ko00904.html)[https:\\www.genome.jp\dbget-bin\www_bget?map00904](file:///c:/website/convert/file/st34dm81hgmvqm3i3v5qk56zkeldkdeb/maps/ko00904.html)

26[EVM002812](file:///c:/website/convert/file/st34dm81hgmvqm3i3v5qk56zkeldkdeb/maps/ko03020.html)[https:\\www.genome.jp\dbget-bin\www_bget?map03020](file:///c:/website/convert/file/st34dm81hgmvqm3i3v5qk56zkeldkdeb/maps/ko03020.html)

 

[novel.121](file:///c:/website/convert/file/st34dm81hgmvqm3i3v5qk56zkeldkdeb/maps/ko03410.html)

[EVM004293](file:///c:/website/convert/file/st34dm81hgmvqm3i3v5qk56zkeldkdeb/maps/ko00960.html)

[novel.455](file:///c:/website/convert/file/st34dm81hgmvqm3i3v5qk56zkeldkdeb/maps/ko00909.html)

[EVM005745](file:///c:/website/convert/file/st34dm81hgmvqm3i3v5qk56zkeldkdeb/maps/ko00040.html)

[novel.132](file:///c:/website/convert/file/st34dm81hgmvqm3i3v5qk56zkeldkdeb/maps/ko03030.html)

[EVM002360](file:///c:/website/convert/file/st34dm81hgmvqm3i3v5qk56zkeldkdeb/maps/ko00190.html)

[EVM003014](file:///c:/website/convert/file/st34dm81hgmvqm3i3v5qk56zkeldkdeb/maps/ko00195.html)

28[EVM003617](file:///c:/website/convert/file/st34dm81hgmvqm3i3v5qk56zkeldkdeb/maps/ko03410.html)[https:\\www.genome.jp\dbget-bin\www_bget?map03410](file:///c:/website/convert/file/st34dm81hgmvqm3i3v5qk56zkeldkdeb/maps/ko03410.html)

9[EVM004400](file:///c:/website/convert/file/st34dm81hgmvqm3i3v5qk56zkeldkdeb/maps/ko00960.html)[https:\\www.genome.jp\dbget-bin\www_bget?map00960](file:///c:/website/convert/file/st34dm81hgmvqm3i3v5qk56zkeldkdeb/maps/ko00960.html)

10[EVM004040](file:///c:/website/convert/file/st34dm81hgmvqm3i3v5qk56zkeldkdeb/maps/ko00909.html)[https:\\www.genome.jp\dbget-bin\www_bget?map00909](file:///c:/website/convert/file/st34dm81hgmvqm3i3v5qk56zkeldkdeb/maps/ko00909.html)

72[EVM002015](file:///c:/website/convert/file/st34dm81hgmvqm3i3v5qk56zkeldkdeb/maps/ko00040.html)[https:\\www.genome.jp\dbget-bin\www_bget?map00040](file:///c:/website/convert/file/st34dm81hgmvqm3i3v5qk56zkeldkdeb/maps/ko00040.html)

29[EVM005212](file:///c:/website/convert/file/st34dm81hgmvqm3i3v5qk56zkeldkdeb/maps/ko03030.html)[https:\\www.genome.jp\dbget-bin\www_bget?map03030](file:///c:/website/convert/file/st34dm81hgmvqm3i3v5qk56zkeldkdeb/maps/ko03030.html)

85[EVM000608](file:///c:/website/convert/file/st34dm81hgmvqm3i3v5qk56zkeldkdeb/maps/ko00190.html)[https:\\www.genome.jp\dbget-bin\www_bget?map00190](file:///c:/website/convert/file/st34dm81hgmvqm3i3v5qk56zkeldkdeb/maps/ko00190.html)

9[EVM004036](file:///c:/website/convert/file/st34dm81hgmvqm3i3v5qk56zkeldkdeb/maps/ko00195.html)[https:\\www.genome.jp\dbget-bin\www_bget?map00195](file:///c:/website/convert/file/st34dm81hgmvqm3i3v5qk56zkeldkdeb/maps/ko00195.html)
