# Supplementary material for: Metabolome and transcriptome analyses identify the characteristics and expression of related saponins of the three genealogical plants of bead ginseng
Source: PeerJ. 2023 Sep 1;11:e16034. doi: 10.7717/peerj.16034 (PMC10476608; doi:10.7717/peerj.16034)
Supplement: Supplementary file 5 [file peerj-11-16034-s010.odt]

Table S4 KEGG enrichment outcome of differential genes of YY vs

Cluster_fGenome_frP-value Correctedcount

KEGG_pathko_id

GeneID KEGG OrthUp

289 EVM006390K01230+K0

24 EVM005399K00275+K0

170 EVM004927K02927+K0

51 EVM000409K00914+K0

45 EVM006287K00053+K0

246 EVM003511K00002+K0

75 EVM003108K00031+K0

21 EVM002937K00728+K0

14 EVM006516K12930+K1

65 EVM000395K00789+K0

100 EVM001395K00601+K0

60 EVM001634K00021+K0

47 novel.580K02202+K0

41 EVM000135K00380+K0

113 EVM002775K03097+K0

272 EVM005140K02863+K0

81 EVM005167K00889+K0

3 EVM002937K00728+K0

115 EVM002492K00003+K0

96 EVM003108K00031+K0

244 EVM005573K03283+K0

28 EVM006284K00939+K0

8 EVM005665K00967+K0

45 EVM006433K00016+K0

86 EVM004648K00010+K0

14 EVM001092K00133+K0

28 EVM005576K00261+K0

56 EVM002389K00001+K0

83 EVM000409K00914+K0

6 EVM004892K03644+K0

67 EVM003429K00020+K0

10 EVM004566K00128+K0

115 EVM002389K00001+K0

32 EVM001704K00993+K0

22 EVM001931K00052+K0

63 EVM002664K00030+K0

64 EVM005340K00025+K0

69 EVM005855K02725+K0

23 EVM003671K00278+K0

40 EVM004566K00128+K0

199 EVM005167K00889+K0

21 EVM006576K00013+K0

Protein pko04141 289 out o1092 out 2.86E-07 4.08E-05

Vitamin Bko00750 24 out of56 out of 0.000103 0.014752

Ubiquitinko04120 170 out o656 out o 0.00024 0.03425

Autophagyko04136 51 out of159 out o 0.000301 0.043008

Pantothenko00770 45 out of137 out o 0.000369 0.052824

Biosyntheko01240 246 out o1021 out 0.001347 0.192676

Glutathioko00480 75 out of273 out o 0.002511 0.359068

Other typko00514 21 out of57 out of 0.002809 0.401641

Anthocyanko00942 14 out of35 out of 0.005933 0.848432

93

3

91

25

20

107

27

7

10

37

53

26

19

15

42

107

38

0

Biosyntheko00999 65 out of241 out o 0.007229

Purine meko00230 100 out o396 out o 0.00895

Terpenoidko00900 60 out of223 out o 0.010055

Basal trako03022 47 out of169 out o 0.011378

Sulfur meko00920 41 out of146 out o 0.014579

Ribosome ko03008 113 out o464 out o 0.017404

Ribosome ko03010 272 out o1200 out 0.020066

Phosphatiko04070 81 out of326 out o 0.024915

Mannose tko00515 3 out of 4 out of 0.02825

Cysteine ko00270 115 out o491 out o 0.04686

Peroxisomko04146 96 out of404 out o 0.046978

Spliceosoko03040 244 out o1095 out 0.050586

Thiamine ko00730 28 out of103 out o 0.055684

Phosphonako00440 8 out of 22 out of 0.060092

Propanoatko00640 45 out of178 out o 0.060536

Inositol ko00562 86 out of363 out o 0.060739

Monobactako00261 14 out of46 out of 0.067533

Arginine ko00220 28 out of105 out o 0.068721

Fatty aciko00071 56 out of229 out o 0.069404

Phagosomeko04145 83 out of355 out o 0.082521

Lipoic acko00785 6 out of 16 out of 0.086208

Valine, lko00280 67 out of285 out o 0.099685

Limonene ko00903 10 out of33 out of 0.114454

Pyruvate ko00620 115 out o512 out o 0.118239

Ether lipko00565 32 out of130 out o 0.131207

Valine, lko00290 22 out of86 out of 0.138152

2-Oxocarbko01210 63 out of274 out o 0.147103

Citrate cko00020 64 out of279 out o 0.149665

Proteasomko03050 69 out of305 out o 0.169415

Nicotinatko00760 23 out of93 out of 0.172168

beta-Alanko00410 40 out of172 out o 0.18796

Endocytosko04144 199 out o929 out o 0.198685

Histidineko00340 21 out of86 out of 0.20263

1

1

1

1

1

1

1

1

1

1

1

1

1

1

1

1

1

1

1

1

1

1

1

1

1

1

1

1

1

1

1

1

1

49

45

90

13

2

19

41

4

13

32

32

3

35

6

43

11

10

32

24

28

13

23

64

12

 

Aminoacylko00970 80 out of362 out o 0.20927

Biotin meko00780 23 out of97 out of 0.232879

Glycosylpko00563 24 out of102 out o 0.239263

Polyketidko00523 2 out of 5 out of 0.26831

ABC transko02010 88 out of408 out o 0.273728

Circadianko04712 65 out of299 out o 0.283891

Monoterpeko00902 15 out of63 out of 0.286485

Glycine, ko00260 57 out of264 out o 0.319156

Starch anko00500 205 out o981 out o 0.322501

Glycerophko00564 104 out o492 out o 0.332138

Plant-patko04626 381 out o1842 out 0.334032

Arginine ko00330 44 out of203 out o 0.335353

alpha-Linko00592 46 out of214 out o 0.353618

Arachidonko00590 27 out of125 out o 0.390231

Nucleocytko03013 182 out o881 out o 0.399703

One carboko00670 16 out of73 out of 0.408378

Brassinosko00905 15 out of69 out of 0.427684

Ascorbateko00053 71 out of342 out o 0.432249

Glyoxylatko00630 73 out of352 out o 0.434623

Butanoateko00650 16 out of76 out of 0.478146

Taurine ako00430 7 out of 32 out of 0.480069

Carotenoiko00906 36 out of175 out o 0.490918

Glycosphiko00601 2 out of 8 out of 0.504689

Fatty aciko00061 52 out of255 out o 0.506134

Benzoxaziko00402 8 out of 39 out of 0.54867

Lysine biko00300 9 out of 45 out of 0.577344

Sulfur reko04122 7 out of 35 out of 0.583041

Glyceroliko00561 85 out of426 out o 0.585142

Biosyntheko01250 110 out o553 out o 0.606172

Biosyntheko01230 238 out o1194 out 0.62915

Fatty aciko01212 86 out of436 out o 0.631856

C5-Branchko00660 7 out of 38 out of 0.674887

Nucleotidko03420 53 out of275 out o 0.683922

Folate biko00790 24 out of127 out o 0.684449

Porphyrinko00860 34 out of179 out o 0.693746

Selenocomko00450 25 out of133 out o 0.697373

Biosyntheko01040 31 out of164 out o 0.698923

Ubiquinonko00130 38 out of200 out o 0.700257

Lysine deko00310 37 out of195 out o 0.701742

Steroid bko00100 22 out of119 out o 0.720711

Riboflaviko00740 17 out of93 out of 0.722909

Protein eko03060 55 out of289 out o 0.723126

Alanine, ko00250 36 out of193 out o 0.739342

MAPK signko04016 238 out o1217 out 0.748263

1

1

1

1

1

1

1

1

1

1

1

1

1

1

1

1

1

1

1

1

1

1

1

1

1

1

1

1

1

1

1

1

1

1

1

1

1

1

1

1

1

1

1

1

80 EVM005818K01866+K0

23 EVM004121K00059+K0

24 EVM003896K03857+K0

2 EVM005885K12451+K1

88 EVM003589K02045+K0

65 EVM002395K00660+K0

15 EVM004481K15095+K1

57 EVM002492K00003+K0

205 EVM005019K00688+K0

104 EVM002519K00006+K0

381 EVM004954K01373+K0

44 EVM004566K00128+K0

46 EVM005364K00224+K0

27 EVM005577K00079+K0

182 EVM005173K03231+K0

16 EVM000521K00288+K0

15 EVM000053K09588+K0

71 EVM003511K00002+K0

73 EVM005340K00025+K0

16 EVM004486K00074+K0

7 EVM000595K00485+K0

36 EVM003633K00514+K0

2 EVM004328K01988+K0

52 EVM004121K00059+K0

8 EVM006167K13229+K1

9 EVM002492K00003+K0

7 EVM000072K01011+K0

85 EVM003511K00002+K0

110 EVM004208K00012+K0

238 EVM002492K00003+K0

86 EVM004121K00059+K0

7 EVM001931K00052+K0

53 novel.580K02202+K0

24 EVM005577K00079+K0

34 EVM005927K00218+K0

25 EVM005874K00384+K0

31 EVM003533K00232+K0

38 EVM000342K00457+K0

37 EVM004566K00128+K0

22 EVM005372K00227+K0

17 EVM000032K00793+K0

55 EVM001663K03070+K0

36 EVM005576K00261+K0

238 EVM006390K00940+K0

38

12

14

0

42

36

9

23

95

47

203

21

22

10

78

3

11

28

31

7

6

22

1

20

1

5

1

38

54

110

43

3

30

13

21

7

17

12

19

9

8

28

20

129

 

SNARE intko04130 28 out of153 out o 0.757815

Tyrosine ko00350 38 out of208 out o 0.788785

Flavone ako00944 9 out of 54 out of 0.79348

RNA degrako03018 114 out o605 out o 0.825035

Viral lifko03250 34 out of191 out o 0.826734

Glycolysiko00010 146 out o769 out o 0.827758

Neomycin,ko00524 5 out of 34 out of 0.847492

Plant horko04075 349 out o1809 out 0.865425

Carbon meko01200 256 out o1339 out 0.867307

Zeatin biko00908 24 out of144 out o 0.883547

Biosyntheko01110 1276 out 6461 out 0.888299

Non-homolko03450 3 out of 24 out of 0.891566

Oxidativeko00190 200 out o1071 out 0.915134

Glycosphiko00603 6 out of 45 out of 0.916835

Amino sugko00520 141 out o774 out o 0.933153

Betalain ko00965 2 out of 20 out of 0.934541

Cyanoaminko00460 47 out of280 out o 0.940106

Metabolicko01100 2336 out 11773 out 0.940614

Phenylalako00400 33 out of204 out o 0.942436

Nitrogen ko00910 21 out of137 out o 0.943447

D-Amino ako00470 2 out of 21 out of 0.945638

N-Glycan ko00510 38 out of234 out o 0.9508

Glucosinoko00966 4 out of 36 out of 0.951816

Tryptophako00380 38 out of235 out o 0.953909

Flavonoidko00941 42 out of258 out o 0.95659

Cutin, suko00073 24 out of161 out o 0.967687

Other glyko00511 47 out of291 out o 0.969228

Fatty aciko00062 19 out of137 out o 0.979818

Indole alko00901 3 out of 35 out of 0.982641

Phenylalako00360 29 out of198 out o 0.983507

Pyrimidinko00240 40 out of264 out o 0.986802

Phenylproko00940 123 out o720 out o 0.98751

Various tko00513 30 out of211 out o 0.991026

Fructose ko00051 64 out of405 out o 0.991256

Mismatch ko03430 31 out of221 out o 0.993737

Stilbenoiko00945 22 out of169 out o 0.994859

Nucleotidko01232 53 out of351 out o 0.994933

Sesquiterko00909 23 out of176 out o 0.995296

Homologouko03440 39 out of279 out o 0.997549

RNA polymko03020 56 out of378 out o 0.997563

Isoflavonko00943 10 out of99 out of 0.997833

Photosyntko00196 4 out of 55 out of 0.997914

mRNA survko03015 133 out o811 out o 0.998099

Base exciko03410 60 out of405 out o 0.998202

1

1

1

1

1

1

1

1

1

1

1

1

1

1

1

1

1

1

1

1

1

1

1

1

1

1

1

1

1

1

1

1

1

1

1

1

1

1

1

1

1

1

1

1

28 EVM003519K08486+K0

38 EVM002389K00001+K0

9 EVM001507K05280+K1

114 EVM004734K00850+K0

34 EVM000906K04498+K0

146 EVM002389K00001+K0

5 EVM001258K00844+K0

349 EVM002226K12126+K1

256 EVM005340K00025+K0

24 EVM003102K00279+K0

1276 EVM002389K00001+K0

3 EVM002655K03512+K1

200 EVM006201K00235+K0

6 EVM004328K01988+K0

141 EVM004208K00012+K0

2 EVM002761K01593+K1

47 EVM001652K00600+K0

2336 EVM002389K00001+K0

33 EVM003230K00766+K0

21 EVM005576K00261+K0

2 EVM003868K12235+K1

38 EVM003456K00721+K0

4 EVM002787K00826+K0

38 EVM004566K00128+K0

42 EVM001480K00487+K0

24 EVM001169K13356+K1

47 EVM001471K01191+K0

19 EVM003033K01074+K0

3 EVM002761K01593+K0

29 EVM004486K00074+K0

40 EVM001722K00207+K0

123 EVM005118K00083+K0

30 EVM004755K00736+K0

64 EVM004755K00008+K0

31 EVM006062K02328+K0

22 EVM001480K00487+K0

53 EVM004972K00759+K0

23 EVM002994K00511+K0

39 EVM006062K02328+K0

56 novel.935K02999+K0

10 EVM004479K13258+K1

4 EVM000522K08911+K0

133 EVM001253K03265+K0

60 novel.121K01142+K0

12

18

3

43

15

60

3

191

101

15

593

1

57

4

69

2

18

1047

15

9

1

25

1

23

24

16

26

8

1

15

23

65

17

34

16

15

31

8

23

22

8

2

67

32

 

Glycosamiko00531 22 out of179 out o 0.998203

Linoleic ko00591 10 out of101 out o 0.998359

Glycosphiko00604 15 out of137 out o 0.99875

Isoquinolko00950 22 out of185 out o 0.999073

Tropane, ko00960 20 out of176 out o 0.999408

Galactoseko00052 60 out of422 out o 0.999526

Sphingoliko00600 39 out of298 out o 0.999568

Carbon fiko00710 64 out of457 out o 0.999817

Diterpenoko00904 10 out of121 out o 0.999914

Photosyntko00195 36 out of297 out o 0.999927

Pentose ako00040 106 out o756 out o 0.999998

Pentose pko00030 43 out of379 out o 0.999999

1

1

1

1

1

1

1

1

1

1

1

1

1

22 EVM001261K01205+K0

10 EVM001464K00454+K0

15 EVM004129K12309+K1

22 EVM004400K00276+K0

20 EVM004400K00276+K0

60 EVM001258K00844+K0

39 EVM004388K00654+K0

64 EVM005340K00025+K0

10 EVM003676K04122+K0

36 novel.147K02108+K0

106 EVM003511K00002+K0

43 EVM001635K00033+K0

44 EVM001468K02209+K0

13

7

12

11

10

31

22

29

5

8

35

16

25

DNA repliko03030 44 out of461 out o

1

 

vs DY group

Up_gene Down

[EVM006390](file:///c:/website/convert/file/st34dm81hgmvqm3i3v5qk56zkeldkdeb/maps/ko04141.html)

[EVM003401](file:///c:/website/convert/file/st34dm81hgmvqm3i3v5qk56zkeldkdeb/maps/ko00750.html)

[EVM004927](file:///c:/website/convert/file/st34dm81hgmvqm3i3v5qk56zkeldkdeb/maps/ko04120.html)

[EVM001349](file:///c:/website/convert/file/st34dm81hgmvqm3i3v5qk56zkeldkdeb/maps/ko04136.html)

[EVM006287](file:///c:/website/convert/file/st34dm81hgmvqm3i3v5qk56zkeldkdeb/maps/ko00770.html)

[EVM005745](file:///c:/website/convert/file/st34dm81hgmvqm3i3v5qk56zkeldkdeb/maps/ko01240.html)

[novel.413](file:///c:/website/convert/file/st34dm81hgmvqm3i3v5qk56zkeldkdeb/maps/ko00480.html)

[EVM005254](file:///c:/website/convert/file/st34dm81hgmvqm3i3v5qk56zkeldkdeb/maps/ko00514.html)

[EVM006516](file:///c:/website/convert/file/st34dm81hgmvqm3i3v5qk56zkeldkdeb/maps/ko00942.html)

[EVM000395](file:///c:/website/convert/file/st34dm81hgmvqm3i3v5qk56zkeldkdeb/maps/ko00999.html)

[EVM005933](file:///c:/website/convert/file/st34dm81hgmvqm3i3v5qk56zkeldkdeb/maps/ko00230.html)

[novel.966](file:///c:/website/convert/file/st34dm81hgmvqm3i3v5qk56zkeldkdeb/maps/ko00900.html)

[EVM000433](file:///c:/website/convert/file/st34dm81hgmvqm3i3v5qk56zkeldkdeb/maps/ko03022.html)

[EVM000135](file:///c:/website/convert/file/st34dm81hgmvqm3i3v5qk56zkeldkdeb/maps/ko00920.html)

[EVM002775](file:///c:/website/convert/file/st34dm81hgmvqm3i3v5qk56zkeldkdeb/maps/ko03008.html)

[EVM001822](file:///c:/website/convert/file/st34dm81hgmvqm3i3v5qk56zkeldkdeb/maps/ko03010.html)

[EVM002112](file:///c:/website/convert/file/st34dm81hgmvqm3i3v5qk56zkeldkdeb/maps/ko04070.html)

--

[Down_genehyperlink](file:///c:/website/convert/file/st34dm81hgmvqm3i3v5qk56zkeldkdeb/maps/ko04141.html)

196[EVM001118](file:///c:/website/convert/file/st34dm81hgmvqm3i3v5qk56zkeldkdeb/maps/ko04141.html)[https:\\www.genome.jp\dbget-bin\www_bget?map04141](file:///c:/website/convert/file/st34dm81hgmvqm3i3v5qk56zkeldkdeb/maps/ko04141.html)

21[EVM005399](file:///c:/website/convert/file/st34dm81hgmvqm3i3v5qk56zkeldkdeb/maps/ko00750.html)[https:\\www.genome.jp\dbget-bin\www_bget?map00750](file:///c:/website/convert/file/st34dm81hgmvqm3i3v5qk56zkeldkdeb/maps/ko00750.html)

79[novel.450](file:///c:/website/convert/file/st34dm81hgmvqm3i3v5qk56zkeldkdeb/maps/ko04120.html)[https:\\www.genome.jp\dbget-bin\www_bget?map04120](file:///c:/website/convert/file/st34dm81hgmvqm3i3v5qk56zkeldkdeb/maps/ko04120.html)

26[EVM000409](file:///c:/website/convert/file/st34dm81hgmvqm3i3v5qk56zkeldkdeb/maps/ko04136.html)[https:\\www.genome.jp\dbget-bin\www_bget?map04136](file:///c:/website/convert/file/st34dm81hgmvqm3i3v5qk56zkeldkdeb/maps/ko04136.html)

25[EVM002954](file:///c:/website/convert/file/st34dm81hgmvqm3i3v5qk56zkeldkdeb/maps/ko00770.html)[https:\\www.genome.jp\dbget-bin\www_bget?map00770](file:///c:/website/convert/file/st34dm81hgmvqm3i3v5qk56zkeldkdeb/maps/ko00770.html)

139[EVM003511](file:///c:/website/convert/file/st34dm81hgmvqm3i3v5qk56zkeldkdeb/maps/ko01240.html)[https:\\www.genome.jp\dbget-bin\www_bget?map01240](file:///c:/website/convert/file/st34dm81hgmvqm3i3v5qk56zkeldkdeb/maps/ko01240.html)

48[EVM003108](file:///c:/website/convert/file/st34dm81hgmvqm3i3v5qk56zkeldkdeb/maps/ko00480.html)[https:\\www.genome.jp\dbget-bin\www_bget?map00480](file:///c:/website/convert/file/st34dm81hgmvqm3i3v5qk56zkeldkdeb/maps/ko00480.html)

14[EVM002937](file:///c:/website/convert/file/st34dm81hgmvqm3i3v5qk56zkeldkdeb/maps/ko00514.html)[https:\\www.genome.jp\dbget-bin\www_bget?map00514](file:///c:/website/convert/file/st34dm81hgmvqm3i3v5qk56zkeldkdeb/maps/ko00514.html)

4[EVM003584](file:///c:/website/convert/file/st34dm81hgmvqm3i3v5qk56zkeldkdeb/maps/ko00942.html)[https:\\www.genome.jp\dbget-bin\www_bget?map00942](file:///c:/website/convert/file/st34dm81hgmvqm3i3v5qk56zkeldkdeb/maps/ko00942.html)

28[EVM000140](file:///c:/website/convert/file/st34dm81hgmvqm3i3v5qk56zkeldkdeb/maps/ko00999.html)[https:\\www.genome.jp\dbget-bin\www_bget?map00999](file:///c:/website/convert/file/st34dm81hgmvqm3i3v5qk56zkeldkdeb/maps/ko00999.html)

47[EVM001395](file:///c:/website/convert/file/st34dm81hgmvqm3i3v5qk56zkeldkdeb/maps/ko00230.html)[https:\\www.genome.jp\dbget-bin\www_bget?map00230](file:///c:/website/convert/file/st34dm81hgmvqm3i3v5qk56zkeldkdeb/maps/ko00230.html)

34[EVM001634](file:///c:/website/convert/file/st34dm81hgmvqm3i3v5qk56zkeldkdeb/maps/ko00900.html)[https:\\www.genome.jp\dbget-bin\www_bget?map00900](file:///c:/website/convert/file/st34dm81hgmvqm3i3v5qk56zkeldkdeb/maps/ko00900.html)

28[novel.580](file:///c:/website/convert/file/st34dm81hgmvqm3i3v5qk56zkeldkdeb/maps/ko03022.html)[https:\\www.genome.jp\dbget-bin\www_bget?map03022](file:///c:/website/convert/file/st34dm81hgmvqm3i3v5qk56zkeldkdeb/maps/ko03022.html)

26[EVM006555](file:///c:/website/convert/file/st34dm81hgmvqm3i3v5qk56zkeldkdeb/maps/ko00920.html)[https:\\www.genome.jp\dbget-bin\www_bget?map00920](file:///c:/website/convert/file/st34dm81hgmvqm3i3v5qk56zkeldkdeb/maps/ko00920.html)

71[EVM000442](file:///c:/website/convert/file/st34dm81hgmvqm3i3v5qk56zkeldkdeb/maps/ko03008.html)[https:\\www.genome.jp\dbget-bin\www_bget?map03008](file:///c:/website/convert/file/st34dm81hgmvqm3i3v5qk56zkeldkdeb/maps/ko03008.html)

165[EVM005140](file:///c:/website/convert/file/st34dm81hgmvqm3i3v5qk56zkeldkdeb/maps/ko03010.html)[https:\\www.genome.jp\dbget-bin\www_bget?map03010](file:///c:/website/convert/file/st34dm81hgmvqm3i3v5qk56zkeldkdeb/maps/ko03010.html)

43[EVM005167](file:///c:/website/convert/file/st34dm81hgmvqm3i3v5qk56zkeldkdeb/maps/ko04070.html)[https:\\www.genome.jp\dbget-bin\www_bget?map04070](file:///c:/website/convert/file/st34dm81hgmvqm3i3v5qk56zkeldkdeb/maps/ko04070.html)

3[EVM002937](file:///c:/website/convert/file/st34dm81hgmvqm3i3v5qk56zkeldkdeb/maps/ko00515.html)[https:\\www.genome.jp\dbget-bin\www_bget?map00515](file:///c:/website/convert/file/st34dm81hgmvqm3i3v5qk56zkeldkdeb/maps/ko00515.html)

66[EVM002492](file:///c:/website/convert/file/st34dm81hgmvqm3i3v5qk56zkeldkdeb/maps/ko00270.html)[https:\\www.genome.jp\dbget-bin\www_bget?map00270](file:///c:/website/convert/file/st34dm81hgmvqm3i3v5qk56zkeldkdeb/maps/ko00270.html)

51[EVM003108](file:///c:/website/convert/file/st34dm81hgmvqm3i3v5qk56zkeldkdeb/maps/ko04146.html)[https:\\www.genome.jp\dbget-bin\www_bget?map04146](file:///c:/website/convert/file/st34dm81hgmvqm3i3v5qk56zkeldkdeb/maps/ko04146.html)

154[EVM005573](file:///c:/website/convert/file/st34dm81hgmvqm3i3v5qk56zkeldkdeb/maps/ko03040.html)[https:\\www.genome.jp\dbget-bin\www_bget?map03040](file:///c:/website/convert/file/st34dm81hgmvqm3i3v5qk56zkeldkdeb/maps/ko03040.html)

15[EVM000363](file:///c:/website/convert/file/st34dm81hgmvqm3i3v5qk56zkeldkdeb/maps/ko00730.html)[https:\\www.genome.jp\dbget-bin\www_bget?map00730](file:///c:/website/convert/file/st34dm81hgmvqm3i3v5qk56zkeldkdeb/maps/ko00730.html)

6[EVM005665](file:///c:/website/convert/file/st34dm81hgmvqm3i3v5qk56zkeldkdeb/maps/ko00440.html)[https:\\www.genome.jp\dbget-bin\www_bget?map00440](file:///c:/website/convert/file/st34dm81hgmvqm3i3v5qk56zkeldkdeb/maps/ko00440.html)

26[EVM003533](file:///c:/website/convert/file/st34dm81hgmvqm3i3v5qk56zkeldkdeb/maps/ko00640.html)[https:\\www.genome.jp\dbget-bin\www_bget?map00640](file:///c:/website/convert/file/st34dm81hgmvqm3i3v5qk56zkeldkdeb/maps/ko00640.html)

45[EVM003526](file:///c:/website/convert/file/st34dm81hgmvqm3i3v5qk56zkeldkdeb/maps/ko00562.html)[https:\\www.genome.jp\dbget-bin\www_bget?map00562](file:///c:/website/convert/file/st34dm81hgmvqm3i3v5qk56zkeldkdeb/maps/ko00562.html)

10[EVM001092](file:///c:/website/convert/file/st34dm81hgmvqm3i3v5qk56zkeldkdeb/maps/ko00261.html)[https:\\www.genome.jp\dbget-bin\www_bget?map00261](file:///c:/website/convert/file/st34dm81hgmvqm3i3v5qk56zkeldkdeb/maps/ko00261.html)

15[EVM005576](file:///c:/website/convert/file/st34dm81hgmvqm3i3v5qk56zkeldkdeb/maps/ko00220.html)[https:\\www.genome.jp\dbget-bin\www_bget?map00220](file:///c:/website/convert/file/st34dm81hgmvqm3i3v5qk56zkeldkdeb/maps/ko00220.html)

24[EVM001679](file:///c:/website/convert/file/st34dm81hgmvqm3i3v5qk56zkeldkdeb/maps/ko00071.html)[https:\\www.genome.jp\dbget-bin\www_bget?map00071](file:///c:/website/convert/file/st34dm81hgmvqm3i3v5qk56zkeldkdeb/maps/ko00071.html)

51[EVM000409](file:///c:/website/convert/file/st34dm81hgmvqm3i3v5qk56zkeldkdeb/maps/ko04145.html)[https:\\www.genome.jp\dbget-bin\www_bget?map04145](file:///c:/website/convert/file/st34dm81hgmvqm3i3v5qk56zkeldkdeb/maps/ko04145.html)

3[EVM004892](file:///c:/website/convert/file/st34dm81hgmvqm3i3v5qk56zkeldkdeb/maps/ko00785.html)[https:\\www.genome.jp\dbget-bin\www_bget?map00785](file:///c:/website/convert/file/st34dm81hgmvqm3i3v5qk56zkeldkdeb/maps/ko00785.html)

32[EVM003149](file:///c:/website/convert/file/st34dm81hgmvqm3i3v5qk56zkeldkdeb/maps/ko00280.html)[https:\\www.genome.jp\dbget-bin\www_bget?map00280](file:///c:/website/convert/file/st34dm81hgmvqm3i3v5qk56zkeldkdeb/maps/ko00280.html)

4[EVM001630](file:///c:/website/convert/file/st34dm81hgmvqm3i3v5qk56zkeldkdeb/maps/ko00903.html)[https:\\www.genome.jp\dbget-bin\www_bget?map00903](file:///c:/website/convert/file/st34dm81hgmvqm3i3v5qk56zkeldkdeb/maps/ko00903.html)

72[EVM001679](file:///c:/website/convert/file/st34dm81hgmvqm3i3v5qk56zkeldkdeb/maps/ko00620.html)[https:\\www.genome.jp\dbget-bin\www_bget?map00620](file:///c:/website/convert/file/st34dm81hgmvqm3i3v5qk56zkeldkdeb/maps/ko00620.html)

21[EVM001704](file:///c:/website/convert/file/st34dm81hgmvqm3i3v5qk56zkeldkdeb/maps/ko00565.html)[https:\\www.genome.jp\dbget-bin\www_bget?map00565](file:///c:/website/convert/file/st34dm81hgmvqm3i3v5qk56zkeldkdeb/maps/ko00565.html)

12[EVM005223](file:///c:/website/convert/file/st34dm81hgmvqm3i3v5qk56zkeldkdeb/maps/ko00290.html)[https:\\www.genome.jp\dbget-bin\www_bget?map00290](file:///c:/website/convert/file/st34dm81hgmvqm3i3v5qk56zkeldkdeb/maps/ko00290.html)

31[EVM005740](file:///c:/website/convert/file/st34dm81hgmvqm3i3v5qk56zkeldkdeb/maps/ko01210.html)[https:\\www.genome.jp\dbget-bin\www_bget?map01210](file:///c:/website/convert/file/st34dm81hgmvqm3i3v5qk56zkeldkdeb/maps/ko01210.html)

40[EVM005340](file:///c:/website/convert/file/st34dm81hgmvqm3i3v5qk56zkeldkdeb/maps/ko00020.html)[https:\\www.genome.jp\dbget-bin\www_bget?map00020](file:///c:/website/convert/file/st34dm81hgmvqm3i3v5qk56zkeldkdeb/maps/ko00020.html)

41[EVM005774](file:///c:/website/convert/file/st34dm81hgmvqm3i3v5qk56zkeldkdeb/maps/ko03050.html)[https:\\www.genome.jp\dbget-bin\www_bget?map03050](file:///c:/website/convert/file/st34dm81hgmvqm3i3v5qk56zkeldkdeb/maps/ko03050.html)

10[EVM003671](file:///c:/website/convert/file/st34dm81hgmvqm3i3v5qk56zkeldkdeb/maps/ko00760.html)[https:\\www.genome.jp\dbget-bin\www_bget?map00760](file:///c:/website/convert/file/st34dm81hgmvqm3i3v5qk56zkeldkdeb/maps/ko00760.html)

17[EVM001630](file:///c:/website/convert/file/st34dm81hgmvqm3i3v5qk56zkeldkdeb/maps/ko00410.html)[https:\\www.genome.jp\dbget-bin\www_bget?map00410](file:///c:/website/convert/file/st34dm81hgmvqm3i3v5qk56zkeldkdeb/maps/ko00410.html)

135[EVM005167](file:///c:/website/convert/file/st34dm81hgmvqm3i3v5qk56zkeldkdeb/maps/ko04144.html)[https:\\www.genome.jp\dbget-bin\www_bget?map04144](file:///c:/website/convert/file/st34dm81hgmvqm3i3v5qk56zkeldkdeb/maps/ko04144.html)

9[EVM001630](file:///c:/website/convert/file/st34dm81hgmvqm3i3v5qk56zkeldkdeb/maps/ko00340.html)[https:\\www.genome.jp\dbget-bin\www_bget?map00340](file:///c:/website/convert/file/st34dm81hgmvqm3i3v5qk56zkeldkdeb/maps/ko00340.html)

[EVM006433](file:///c:/website/convert/file/st34dm81hgmvqm3i3v5qk56zkeldkdeb/maps/ko00270.html)

[novel.413](file:///c:/website/convert/file/st34dm81hgmvqm3i3v5qk56zkeldkdeb/maps/ko04146.html)

[EVM002431](file:///c:/website/convert/file/st34dm81hgmvqm3i3v5qk56zkeldkdeb/maps/ko03040.html)

[EVM006284](file:///c:/website/convert/file/st34dm81hgmvqm3i3v5qk56zkeldkdeb/maps/ko00730.html)

EVM002559

[EVM006433](file:///c:/website/convert/file/st34dm81hgmvqm3i3v5qk56zkeldkdeb/maps/ko00640.html)

[EVM004648](file:///c:/website/convert/file/st34dm81hgmvqm3i3v5qk56zkeldkdeb/maps/ko00562.html)

[EVM002531](file:///c:/website/convert/file/st34dm81hgmvqm3i3v5qk56zkeldkdeb/maps/ko00261.html)

[EVM001260](file:///c:/website/convert/file/st34dm81hgmvqm3i3v5qk56zkeldkdeb/maps/ko00220.html)

[EVM002389](file:///c:/website/convert/file/st34dm81hgmvqm3i3v5qk56zkeldkdeb/maps/ko00071.html)

[EVM003924](file:///c:/website/convert/file/st34dm81hgmvqm3i3v5qk56zkeldkdeb/maps/ko04145.html)

[EVM002385](file:///c:/website/convert/file/st34dm81hgmvqm3i3v5qk56zkeldkdeb/maps/ko00785.html)

[EVM003429](file:///c:/website/convert/file/st34dm81hgmvqm3i3v5qk56zkeldkdeb/maps/ko00280.html)

[EVM004566](file:///c:/website/convert/file/st34dm81hgmvqm3i3v5qk56zkeldkdeb/maps/ko00903.html)

[EVM002389](file:///c:/website/convert/file/st34dm81hgmvqm3i3v5qk56zkeldkdeb/maps/ko00620.html)

[EVM001858](file:///c:/website/convert/file/st34dm81hgmvqm3i3v5qk56zkeldkdeb/maps/ko00565.html)

[EVM001931](file:///c:/website/convert/file/st34dm81hgmvqm3i3v5qk56zkeldkdeb/maps/ko00290.html)

[EVM002664](file:///c:/website/convert/file/st34dm81hgmvqm3i3v5qk56zkeldkdeb/maps/ko01210.html)

[novel.322](file:///c:/website/convert/file/st34dm81hgmvqm3i3v5qk56zkeldkdeb/maps/ko00020.html)

[EVM005855](file:///c:/website/convert/file/st34dm81hgmvqm3i3v5qk56zkeldkdeb/maps/ko03050.html)

[EVM001231](file:///c:/website/convert/file/st34dm81hgmvqm3i3v5qk56zkeldkdeb/maps/ko00760.html)

[EVM004566](file:///c:/website/convert/file/st34dm81hgmvqm3i3v5qk56zkeldkdeb/maps/ko00410.html)

[EVM002393](file:///c:/website/convert/file/st34dm81hgmvqm3i3v5qk56zkeldkdeb/maps/ko04144.html)

[EVM006576](file:///c:/website/convert/file/st34dm81hgmvqm3i3v5qk56zkeldkdeb/maps/ko00340.html)

 

[EVM004618](file:///c:/website/convert/file/st34dm81hgmvqm3i3v5qk56zkeldkdeb/maps/ko00970.html)

[EVM000102](file:///c:/website/convert/file/st34dm81hgmvqm3i3v5qk56zkeldkdeb/maps/ko00780.html)

[EVM003896](file:///c:/website/convert/file/st34dm81hgmvqm3i3v5qk56zkeldkdeb/maps/ko00563.html)

--

42[EVM005818](file:///c:/website/convert/file/st34dm81hgmvqm3i3v5qk56zkeldkdeb/maps/ko00970.html)[https:\\www.genome.jp\dbget-bin\www_bget?map00970](file:///c:/website/convert/file/st34dm81hgmvqm3i3v5qk56zkeldkdeb/maps/ko00970.html)

11[EVM004121](file:///c:/website/convert/file/st34dm81hgmvqm3i3v5qk56zkeldkdeb/maps/ko00780.html)[https:\\www.genome.jp\dbget-bin\www_bget?map00780](file:///c:/website/convert/file/st34dm81hgmvqm3i3v5qk56zkeldkdeb/maps/ko00780.html)

10[novel.545](file:///c:/website/convert/file/st34dm81hgmvqm3i3v5qk56zkeldkdeb/maps/ko00563.html)[https:\\www.genome.jp\dbget-bin\www_bget?map00563](file:///c:/website/convert/file/st34dm81hgmvqm3i3v5qk56zkeldkdeb/maps/ko00563.html)

2[EVM005885](file:///c:/website/convert/file/st34dm81hgmvqm3i3v5qk56zkeldkdeb/maps/ko00523.html)[https:\\www.genome.jp\dbget-bin\www_bget?map00523](file:///c:/website/convert/file/st34dm81hgmvqm3i3v5qk56zkeldkdeb/maps/ko00523.html)

46[novel.141](file:///c:/website/convert/file/st34dm81hgmvqm3i3v5qk56zkeldkdeb/maps/ko02010.html)[https:\\www.genome.jp\dbget-bin\www_bget?map02010](file:///c:/website/convert/file/st34dm81hgmvqm3i3v5qk56zkeldkdeb/maps/ko02010.html)

29[novel.125](file:///c:/website/convert/file/st34dm81hgmvqm3i3v5qk56zkeldkdeb/maps/ko04712.html)[https:\\www.genome.jp\dbget-bin\www_bget?map04712](file:///c:/website/convert/file/st34dm81hgmvqm3i3v5qk56zkeldkdeb/maps/ko04712.html)

6[EVM004696](file:///c:/website/convert/file/st34dm81hgmvqm3i3v5qk56zkeldkdeb/maps/ko00902.html)[https:\\www.genome.jp\dbget-bin\www_bget?map00902](file:///c:/website/convert/file/st34dm81hgmvqm3i3v5qk56zkeldkdeb/maps/ko00902.html)

34[EVM002492](file:///c:/website/convert/file/st34dm81hgmvqm3i3v5qk56zkeldkdeb/maps/ko00260.html)[https:\\www.genome.jp\dbget-bin\www_bget?map00260](file:///c:/website/convert/file/st34dm81hgmvqm3i3v5qk56zkeldkdeb/maps/ko00260.html)

110[EVM005019](file:///c:/website/convert/file/st34dm81hgmvqm3i3v5qk56zkeldkdeb/maps/ko00500.html)[https:\\www.genome.jp\dbget-bin\www_bget?map00500](file:///c:/website/convert/file/st34dm81hgmvqm3i3v5qk56zkeldkdeb/maps/ko00500.html)

57[EVM002519](file:///c:/website/convert/file/st34dm81hgmvqm3i3v5qk56zkeldkdeb/maps/ko00564.html)[https:\\www.genome.jp\dbget-bin\www_bget?map00564](file:///c:/website/convert/file/st34dm81hgmvqm3i3v5qk56zkeldkdeb/maps/ko00564.html)

178[EVM004954](file:///c:/website/convert/file/st34dm81hgmvqm3i3v5qk56zkeldkdeb/maps/ko04626.html)[https:\\www.genome.jp\dbget-bin\www_bget?map04626](file:///c:/website/convert/file/st34dm81hgmvqm3i3v5qk56zkeldkdeb/maps/ko04626.html)

23[EVM001630](file:///c:/website/convert/file/st34dm81hgmvqm3i3v5qk56zkeldkdeb/maps/ko00330.html)[https:\\www.genome.jp\dbget-bin\www_bget?map00330](file:///c:/website/convert/file/st34dm81hgmvqm3i3v5qk56zkeldkdeb/maps/ko00330.html)

24[EVM005364](file:///c:/website/convert/file/st34dm81hgmvqm3i3v5qk56zkeldkdeb/maps/ko00592.html)[https:\\www.genome.jp\dbget-bin\www_bget?map00592](file:///c:/website/convert/file/st34dm81hgmvqm3i3v5qk56zkeldkdeb/maps/ko00592.html)

17[EVM005577](file:///c:/website/convert/file/st34dm81hgmvqm3i3v5qk56zkeldkdeb/maps/ko00590.html)[https:\\www.genome.jp\dbget-bin\www_bget?map00590](file:///c:/website/convert/file/st34dm81hgmvqm3i3v5qk56zkeldkdeb/maps/ko00590.html)

104[EVM005173](file:///c:/website/convert/file/st34dm81hgmvqm3i3v5qk56zkeldkdeb/maps/ko03013.html)[https:\\www.genome.jp\dbget-bin\www_bget?map03013](file:///c:/website/convert/file/st34dm81hgmvqm3i3v5qk56zkeldkdeb/maps/ko03013.html)

13[EVM000521](file:///c:/website/convert/file/st34dm81hgmvqm3i3v5qk56zkeldkdeb/maps/ko00670.html)[https:\\www.genome.jp\dbget-bin\www_bget?map00670](file:///c:/website/convert/file/st34dm81hgmvqm3i3v5qk56zkeldkdeb/maps/ko00670.html)

4[EVM001020](file:///c:/website/convert/file/st34dm81hgmvqm3i3v5qk56zkeldkdeb/maps/ko00905.html)[https:\\www.genome.jp\dbget-bin\www_bget?map00905](file:///c:/website/convert/file/st34dm81hgmvqm3i3v5qk56zkeldkdeb/maps/ko00905.html)

43[EVM003511](file:///c:/website/convert/file/st34dm81hgmvqm3i3v5qk56zkeldkdeb/maps/ko00053.html)[https:\\www.genome.jp\dbget-bin\www_bget?map00053](file:///c:/website/convert/file/st34dm81hgmvqm3i3v5qk56zkeldkdeb/maps/ko00053.html)

42[EVM005340](file:///c:/website/convert/file/st34dm81hgmvqm3i3v5qk56zkeldkdeb/maps/ko00630.html)[https:\\www.genome.jp\dbget-bin\www_bget?map00630](file:///c:/website/convert/file/st34dm81hgmvqm3i3v5qk56zkeldkdeb/maps/ko00630.html)

9[EVM004486](file:///c:/website/convert/file/st34dm81hgmvqm3i3v5qk56zkeldkdeb/maps/ko00650.html)[https:\\www.genome.jp\dbget-bin\www_bget?map00650](file:///c:/website/convert/file/st34dm81hgmvqm3i3v5qk56zkeldkdeb/maps/ko00650.html)

1[EVM004859](file:///c:/website/convert/file/st34dm81hgmvqm3i3v5qk56zkeldkdeb/maps/ko00430.html)[https:\\www.genome.jp\dbget-bin\www_bget?map00430](file:///c:/website/convert/file/st34dm81hgmvqm3i3v5qk56zkeldkdeb/maps/ko00430.html)

14[EVM003633](file:///c:/website/convert/file/st34dm81hgmvqm3i3v5qk56zkeldkdeb/maps/ko00906.html)[https:\\www.genome.jp\dbget-bin\www_bget?map00906](file:///c:/website/convert/file/st34dm81hgmvqm3i3v5qk56zkeldkdeb/maps/ko00906.html)

1[EVM004328](file:///c:/website/convert/file/st34dm81hgmvqm3i3v5qk56zkeldkdeb/maps/ko00601.html)[https:\\www.genome.jp\dbget-bin\www_bget?map00601](file:///c:/website/convert/file/st34dm81hgmvqm3i3v5qk56zkeldkdeb/maps/ko00601.html)

32[EVM004121](file:///c:/website/convert/file/st34dm81hgmvqm3i3v5qk56zkeldkdeb/maps/ko00061.html)[https:\\www.genome.jp\dbget-bin\www_bget?map00061](file:///c:/website/convert/file/st34dm81hgmvqm3i3v5qk56zkeldkdeb/maps/ko00061.html)

7[EVM006167](file:///c:/website/convert/file/st34dm81hgmvqm3i3v5qk56zkeldkdeb/maps/ko00402.html)[https:\\www.genome.jp\dbget-bin\www_bget?map00402](file:///c:/website/convert/file/st34dm81hgmvqm3i3v5qk56zkeldkdeb/maps/ko00402.html)

4[EVM002492](file:///c:/website/convert/file/st34dm81hgmvqm3i3v5qk56zkeldkdeb/maps/ko00300.html)[https:\\www.genome.jp\dbget-bin\www_bget?map00300](file:///c:/website/convert/file/st34dm81hgmvqm3i3v5qk56zkeldkdeb/maps/ko00300.html)

6[EVM000072](file:///c:/website/convert/file/st34dm81hgmvqm3i3v5qk56zkeldkdeb/maps/ko04122.html)[https:\\www.genome.jp\dbget-bin\www_bget?map04122](file:///c:/website/convert/file/st34dm81hgmvqm3i3v5qk56zkeldkdeb/maps/ko04122.html)

47[EVM003511](file:///c:/website/convert/file/st34dm81hgmvqm3i3v5qk56zkeldkdeb/maps/ko00561.html)[https:\\www.genome.jp\dbget-bin\www_bget?map00561](file:///c:/website/convert/file/st34dm81hgmvqm3i3v5qk56zkeldkdeb/maps/ko00561.html)

56[EVM004208](file:///c:/website/convert/file/st34dm81hgmvqm3i3v5qk56zkeldkdeb/maps/ko01250.html)[https:\\www.genome.jp\dbget-bin\www_bget?map01250](file:///c:/website/convert/file/st34dm81hgmvqm3i3v5qk56zkeldkdeb/maps/ko01250.html)

128[EVM002492](file:///c:/website/convert/file/st34dm81hgmvqm3i3v5qk56zkeldkdeb/maps/ko01230.html)[https:\\www.genome.jp\dbget-bin\www_bget?map01230](file:///c:/website/convert/file/st34dm81hgmvqm3i3v5qk56zkeldkdeb/maps/ko01230.html)

43[EVM004121](file:///c:/website/convert/file/st34dm81hgmvqm3i3v5qk56zkeldkdeb/maps/ko01212.html)[https:\\www.genome.jp\dbget-bin\www_bget?map01212](file:///c:/website/convert/file/st34dm81hgmvqm3i3v5qk56zkeldkdeb/maps/ko01212.html)

4[EVM005223](file:///c:/website/convert/file/st34dm81hgmvqm3i3v5qk56zkeldkdeb/maps/ko00660.html)[https:\\www.genome.jp\dbget-bin\www_bget?map00660](file:///c:/website/convert/file/st34dm81hgmvqm3i3v5qk56zkeldkdeb/maps/ko00660.html)

23[novel.580](file:///c:/website/convert/file/st34dm81hgmvqm3i3v5qk56zkeldkdeb/maps/ko03420.html)[https:\\www.genome.jp\dbget-bin\www_bget?map03420](file:///c:/website/convert/file/st34dm81hgmvqm3i3v5qk56zkeldkdeb/maps/ko03420.html)

11[EVM005577](file:///c:/website/convert/file/st34dm81hgmvqm3i3v5qk56zkeldkdeb/maps/ko00790.html)[https:\\www.genome.jp\dbget-bin\www_bget?map00790](file:///c:/website/convert/file/st34dm81hgmvqm3i3v5qk56zkeldkdeb/maps/ko00790.html)

13[EVM005927](file:///c:/website/convert/file/st34dm81hgmvqm3i3v5qk56zkeldkdeb/maps/ko00860.html)[https:\\www.genome.jp\dbget-bin\www_bget?map00860](file:///c:/website/convert/file/st34dm81hgmvqm3i3v5qk56zkeldkdeb/maps/ko00860.html)

18[EVM005874](file:///c:/website/convert/file/st34dm81hgmvqm3i3v5qk56zkeldkdeb/maps/ko00450.html)[https:\\www.genome.jp\dbget-bin\www_bget?map00450](file:///c:/website/convert/file/st34dm81hgmvqm3i3v5qk56zkeldkdeb/maps/ko00450.html)

14[EVM003533](file:///c:/website/convert/file/st34dm81hgmvqm3i3v5qk56zkeldkdeb/maps/ko01040.html)[https:\\www.genome.jp\dbget-bin\www_bget?map01040](file:///c:/website/convert/file/st34dm81hgmvqm3i3v5qk56zkeldkdeb/maps/ko01040.html)

26[EVM000342](file:///c:/website/convert/file/st34dm81hgmvqm3i3v5qk56zkeldkdeb/maps/ko00130.html)[https:\\www.genome.jp\dbget-bin\www_bget?map00130](file:///c:/website/convert/file/st34dm81hgmvqm3i3v5qk56zkeldkdeb/maps/ko00130.html)

18[EVM001630](file:///c:/website/convert/file/st34dm81hgmvqm3i3v5qk56zkeldkdeb/maps/ko00310.html)[https:\\www.genome.jp\dbget-bin\www_bget?map00310](file:///c:/website/convert/file/st34dm81hgmvqm3i3v5qk56zkeldkdeb/maps/ko00310.html)

13[EVM005372](file:///c:/website/convert/file/st34dm81hgmvqm3i3v5qk56zkeldkdeb/maps/ko00100.html)[https:\\www.genome.jp\dbget-bin\www_bget?map00100](file:///c:/website/convert/file/st34dm81hgmvqm3i3v5qk56zkeldkdeb/maps/ko00100.html)

9[EVM000032](file:///c:/website/convert/file/st34dm81hgmvqm3i3v5qk56zkeldkdeb/maps/ko00740.html)[https:\\www.genome.jp\dbget-bin\www_bget?map00740](file:///c:/website/convert/file/st34dm81hgmvqm3i3v5qk56zkeldkdeb/maps/ko00740.html)

27[novel.441](file:///c:/website/convert/file/st34dm81hgmvqm3i3v5qk56zkeldkdeb/maps/ko03060.html)[https:\\www.genome.jp\dbget-bin\www_bget?map03060](file:///c:/website/convert/file/st34dm81hgmvqm3i3v5qk56zkeldkdeb/maps/ko03060.html)

16[EVM005576](file:///c:/website/convert/file/st34dm81hgmvqm3i3v5qk56zkeldkdeb/maps/ko00250.html)[https:\\www.genome.jp\dbget-bin\www_bget?map00250](file:///c:/website/convert/file/st34dm81hgmvqm3i3v5qk56zkeldkdeb/maps/ko00250.html)

109[EVM002440](file:///c:/website/convert/file/st34dm81hgmvqm3i3v5qk56zkeldkdeb/maps/ko04016.html)[https:\\www.genome.jp\dbget-bin\www_bget?map04016](file:///c:/website/convert/file/st34dm81hgmvqm3i3v5qk56zkeldkdeb/maps/ko04016.html)

[EVM003589](file:///c:/website/convert/file/st34dm81hgmvqm3i3v5qk56zkeldkdeb/maps/ko02010.html)

[EVM002395](file:///c:/website/convert/file/st34dm81hgmvqm3i3v5qk56zkeldkdeb/maps/ko04712.html)

[EVM004481](file:///c:/website/convert/file/st34dm81hgmvqm3i3v5qk56zkeldkdeb/maps/ko00902.html)

[EVM001290](file:///c:/website/convert/file/st34dm81hgmvqm3i3v5qk56zkeldkdeb/maps/ko00260.html)

[EVM000678](file:///c:/website/convert/file/st34dm81hgmvqm3i3v5qk56zkeldkdeb/maps/ko00500.html)

[EVM003714](file:///c:/website/convert/file/st34dm81hgmvqm3i3v5qk56zkeldkdeb/maps/ko00564.html)

[EVM004744](file:///c:/website/convert/file/st34dm81hgmvqm3i3v5qk56zkeldkdeb/maps/ko04626.html)

[EVM004566](file:///c:/website/convert/file/st34dm81hgmvqm3i3v5qk56zkeldkdeb/maps/ko00330.html)

[EVM002110](file:///c:/website/convert/file/st34dm81hgmvqm3i3v5qk56zkeldkdeb/maps/ko00592.html)

[EVM006373](file:///c:/website/convert/file/st34dm81hgmvqm3i3v5qk56zkeldkdeb/maps/ko00590.html)

[EVM001349](file:///c:/website/convert/file/st34dm81hgmvqm3i3v5qk56zkeldkdeb/maps/ko03013.html)

[EVM001652](file:///c:/website/convert/file/st34dm81hgmvqm3i3v5qk56zkeldkdeb/maps/ko00670.html)

[EVM000053](file:///c:/website/convert/file/st34dm81hgmvqm3i3v5qk56zkeldkdeb/maps/ko00905.html)

[EVM005745](file:///c:/website/convert/file/st34dm81hgmvqm3i3v5qk56zkeldkdeb/maps/ko00053.html)

[novel.322](file:///c:/website/convert/file/st34dm81hgmvqm3i3v5qk56zkeldkdeb/maps/ko00630.html)

[EVM003385](file:///c:/website/convert/file/st34dm81hgmvqm3i3v5qk56zkeldkdeb/maps/ko00650.html)

[EVM000595](file:///c:/website/convert/file/st34dm81hgmvqm3i3v5qk56zkeldkdeb/maps/ko00430.html)

[EVM003050](file:///c:/website/convert/file/st34dm81hgmvqm3i3v5qk56zkeldkdeb/maps/ko00906.html)

EVM000473

[EVM000102](file:///c:/website/convert/file/st34dm81hgmvqm3i3v5qk56zkeldkdeb/maps/ko00061.html)

EVM002500

[EVM002531](file:///c:/website/convert/file/st34dm81hgmvqm3i3v5qk56zkeldkdeb/maps/ko00300.html)

EVM003290

[EVM004566](file:///c:/website/convert/file/st34dm81hgmvqm3i3v5qk56zkeldkdeb/maps/ko00561.html)

[EVM005745](file:///c:/website/convert/file/st34dm81hgmvqm3i3v5qk56zkeldkdeb/maps/ko01250.html)

[EVM006576](file:///c:/website/convert/file/st34dm81hgmvqm3i3v5qk56zkeldkdeb/maps/ko01230.html)

[EVM000102](file:///c:/website/convert/file/st34dm81hgmvqm3i3v5qk56zkeldkdeb/maps/ko01212.html)

[EVM001931](file:///c:/website/convert/file/st34dm81hgmvqm3i3v5qk56zkeldkdeb/maps/ko00660.html)

[EVM004050](file:///c:/website/convert/file/st34dm81hgmvqm3i3v5qk56zkeldkdeb/maps/ko03420.html)

[EVM006182](file:///c:/website/convert/file/st34dm81hgmvqm3i3v5qk56zkeldkdeb/maps/ko00790.html)

[EVM005189](file:///c:/website/convert/file/st34dm81hgmvqm3i3v5qk56zkeldkdeb/maps/ko00860.html)

[EVM001490](file:///c:/website/convert/file/st34dm81hgmvqm3i3v5qk56zkeldkdeb/maps/ko00450.html)

[EVM002110](file:///c:/website/convert/file/st34dm81hgmvqm3i3v5qk56zkeldkdeb/maps/ko01040.html)

[EVM001480](file:///c:/website/convert/file/st34dm81hgmvqm3i3v5qk56zkeldkdeb/maps/ko00130.html)

[EVM004566](file:///c:/website/convert/file/st34dm81hgmvqm3i3v5qk56zkeldkdeb/maps/ko00310.html)

[EVM003859](file:///c:/website/convert/file/st34dm81hgmvqm3i3v5qk56zkeldkdeb/maps/ko00100.html)

[EVM001298](file:///c:/website/convert/file/st34dm81hgmvqm3i3v5qk56zkeldkdeb/maps/ko00740.html)

[EVM001663](file:///c:/website/convert/file/st34dm81hgmvqm3i3v5qk56zkeldkdeb/maps/ko03060.html)

[EVM003829](file:///c:/website/convert/file/st34dm81hgmvqm3i3v5qk56zkeldkdeb/maps/ko00250.html)

[EVM006390](file:///c:/website/convert/file/st34dm81hgmvqm3i3v5qk56zkeldkdeb/maps/ko04016.html)

 

[EVM003519](file:///c:/website/convert/file/st34dm81hgmvqm3i3v5qk56zkeldkdeb/maps/ko04130.html)

[EVM002389](file:///c:/website/convert/file/st34dm81hgmvqm3i3v5qk56zkeldkdeb/maps/ko00350.html)

[EVM005678](file:///c:/website/convert/file/st34dm81hgmvqm3i3v5qk56zkeldkdeb/maps/ko00944.html)

[EVM004193](file:///c:/website/convert/file/st34dm81hgmvqm3i3v5qk56zkeldkdeb/maps/ko03018.html)

[EVM000906](file:///c:/website/convert/file/st34dm81hgmvqm3i3v5qk56zkeldkdeb/maps/ko03250.html)

[EVM002389](file:///c:/website/convert/file/st34dm81hgmvqm3i3v5qk56zkeldkdeb/maps/ko00010.html)

[EVM001258](file:///c:/website/convert/file/st34dm81hgmvqm3i3v5qk56zkeldkdeb/maps/ko00524.html)

[EVM004209](file:///c:/website/convert/file/st34dm81hgmvqm3i3v5qk56zkeldkdeb/maps/ko04075.html)

[novel.322](file:///c:/website/convert/file/st34dm81hgmvqm3i3v5qk56zkeldkdeb/maps/ko01200.html)

[EVM003102](file:///c:/website/convert/file/st34dm81hgmvqm3i3v5qk56zkeldkdeb/maps/ko00908.html)

[EVM002389](file:///c:/website/convert/file/st34dm81hgmvqm3i3v5qk56zkeldkdeb/maps/ko01110.html)

EVM000001

[EVM001812](file:///c:/website/convert/file/st34dm81hgmvqm3i3v5qk56zkeldkdeb/maps/ko00190.html)

[EVM000473](file:///c:/website/convert/file/st34dm81hgmvqm3i3v5qk56zkeldkdeb/maps/ko00603.html)

[EVM005745](file:///c:/website/convert/file/st34dm81hgmvqm3i3v5qk56zkeldkdeb/maps/ko00520.html)

EVM002761

[EVM001652](file:///c:/website/convert/file/st34dm81hgmvqm3i3v5qk56zkeldkdeb/maps/ko00460.html)

[EVM002389](file:///c:/website/convert/file/st34dm81hgmvqm3i3v5qk56zkeldkdeb/maps/ko01100.html)

[EVM003230](file:///c:/website/convert/file/st34dm81hgmvqm3i3v5qk56zkeldkdeb/maps/ko00400.html)

[EVM003829](file:///c:/website/convert/file/st34dm81hgmvqm3i3v5qk56zkeldkdeb/maps/ko00910.html)

EVM006387

[EVM003456](file:///c:/website/convert/file/st34dm81hgmvqm3i3v5qk56zkeldkdeb/maps/ko00510.html)

EVM002787

[EVM004566](file:///c:/website/convert/file/st34dm81hgmvqm3i3v5qk56zkeldkdeb/maps/ko00380.html)

[EVM001480](file:///c:/website/convert/file/st34dm81hgmvqm3i3v5qk56zkeldkdeb/maps/ko00941.html)

[EVM001169](file:///c:/website/convert/file/st34dm81hgmvqm3i3v5qk56zkeldkdeb/maps/ko00073.html)

[EVM003527](file:///c:/website/convert/file/st34dm81hgmvqm3i3v5qk56zkeldkdeb/maps/ko00511.html)

[EVM000905](file:///c:/website/convert/file/st34dm81hgmvqm3i3v5qk56zkeldkdeb/maps/ko00062.html)

EVM002761

[EVM001290](file:///c:/website/convert/file/st34dm81hgmvqm3i3v5qk56zkeldkdeb/maps/ko00360.html)

[EVM005655](file:///c:/website/convert/file/st34dm81hgmvqm3i3v5qk56zkeldkdeb/maps/ko00240.html)

[EVM005118](file:///c:/website/convert/file/st34dm81hgmvqm3i3v5qk56zkeldkdeb/maps/ko00940.html)

[EVM004755](file:///c:/website/convert/file/st34dm81hgmvqm3i3v5qk56zkeldkdeb/maps/ko00513.html)

[EVM001258](file:///c:/website/convert/file/st34dm81hgmvqm3i3v5qk56zkeldkdeb/maps/ko00051.html)

[EVM006062](file:///c:/website/convert/file/st34dm81hgmvqm3i3v5qk56zkeldkdeb/maps/ko03430.html)

[EVM001480](file:///c:/website/convert/file/st34dm81hgmvqm3i3v5qk56zkeldkdeb/maps/ko00945.html)

[EVM004972](file:///c:/website/convert/file/st34dm81hgmvqm3i3v5qk56zkeldkdeb/maps/ko01232.html)

[novel.455](file:///c:/website/convert/file/st34dm81hgmvqm3i3v5qk56zkeldkdeb/maps/ko00909.html)

[EVM006062](file:///c:/website/convert/file/st34dm81hgmvqm3i3v5qk56zkeldkdeb/maps/ko03440.html)

[novel.935](file:///c:/website/convert/file/st34dm81hgmvqm3i3v5qk56zkeldkdeb/maps/ko03020.html)

[EVM006513](file:///c:/website/convert/file/st34dm81hgmvqm3i3v5qk56zkeldkdeb/maps/ko00943.html)

EVM000522

[EVM002257](file:///c:/website/convert/file/st34dm81hgmvqm3i3v5qk56zkeldkdeb/maps/ko03015.html)

[novel.121](file:///c:/website/convert/file/st34dm81hgmvqm3i3v5qk56zkeldkdeb/maps/ko03410.html)

16[EVM004538](file:///c:/website/convert/file/st34dm81hgmvqm3i3v5qk56zkeldkdeb/maps/ko04130.html)[https:\\www.genome.jp\dbget-bin\www_bget?map04130](file:///c:/website/convert/file/st34dm81hgmvqm3i3v5qk56zkeldkdeb/maps/ko04130.html)

20[EVM001679](file:///c:/website/convert/file/st34dm81hgmvqm3i3v5qk56zkeldkdeb/maps/ko00350.html)[https:\\www.genome.jp\dbget-bin\www_bget?map00350](file:///c:/website/convert/file/st34dm81hgmvqm3i3v5qk56zkeldkdeb/maps/ko00350.html)

6[EVM001507](file:///c:/website/convert/file/st34dm81hgmvqm3i3v5qk56zkeldkdeb/maps/ko00944.html)[https:\\www.genome.jp\dbget-bin\www_bget?map00944](file:///c:/website/convert/file/st34dm81hgmvqm3i3v5qk56zkeldkdeb/maps/ko00944.html)

71[EVM004734](file:///c:/website/convert/file/st34dm81hgmvqm3i3v5qk56zkeldkdeb/maps/ko03018.html)[https:\\www.genome.jp\dbget-bin\www_bget?map03018](file:///c:/website/convert/file/st34dm81hgmvqm3i3v5qk56zkeldkdeb/maps/ko03018.html)

19[EVM004562](file:///c:/website/convert/file/st34dm81hgmvqm3i3v5qk56zkeldkdeb/maps/ko03250.html)[https:\\www.genome.jp\dbget-bin\www_bget?map03250](file:///c:/website/convert/file/st34dm81hgmvqm3i3v5qk56zkeldkdeb/maps/ko03250.html)

86[EVM001679](file:///c:/website/convert/file/st34dm81hgmvqm3i3v5qk56zkeldkdeb/maps/ko00010.html)[https:\\www.genome.jp\dbget-bin\www_bget?map00010](file:///c:/website/convert/file/st34dm81hgmvqm3i3v5qk56zkeldkdeb/maps/ko00010.html)

2[EVM001219](file:///c:/website/convert/file/st34dm81hgmvqm3i3v5qk56zkeldkdeb/maps/ko00524.html)[https:\\www.genome.jp\dbget-bin\www_bget?map00524](file:///c:/website/convert/file/st34dm81hgmvqm3i3v5qk56zkeldkdeb/maps/ko00524.html)

158[EVM002226](file:///c:/website/convert/file/st34dm81hgmvqm3i3v5qk56zkeldkdeb/maps/ko04075.html)[https:\\www.genome.jp\dbget-bin\www_bget?map04075](file:///c:/website/convert/file/st34dm81hgmvqm3i3v5qk56zkeldkdeb/maps/ko04075.html)

155[EVM005340](file:///c:/website/convert/file/st34dm81hgmvqm3i3v5qk56zkeldkdeb/maps/ko01200.html)[https:\\www.genome.jp\dbget-bin\www_bget?map01200](file:///c:/website/convert/file/st34dm81hgmvqm3i3v5qk56zkeldkdeb/maps/ko01200.html)

9[EVM005783](file:///c:/website/convert/file/st34dm81hgmvqm3i3v5qk56zkeldkdeb/maps/ko00908.html)[https:\\www.genome.jp\dbget-bin\www_bget?map00908](file:///c:/website/convert/file/st34dm81hgmvqm3i3v5qk56zkeldkdeb/maps/ko00908.html)

683[EVM001679](file:///c:/website/convert/file/st34dm81hgmvqm3i3v5qk56zkeldkdeb/maps/ko01110.html)[https:\\www.genome.jp\dbget-bin\www_bget?map01110](file:///c:/website/convert/file/st34dm81hgmvqm3i3v5qk56zkeldkdeb/maps/ko01110.html)

2[EVM002655](file:///c:/website/convert/file/st34dm81hgmvqm3i3v5qk56zkeldkdeb/maps/ko03450.html)[https:\\www.genome.jp\dbget-bin\www_bget?map03450](file:///c:/website/convert/file/st34dm81hgmvqm3i3v5qk56zkeldkdeb/maps/ko03450.html)

143[EVM006201](file:///c:/website/convert/file/st34dm81hgmvqm3i3v5qk56zkeldkdeb/maps/ko00190.html)[https:\\www.genome.jp\dbget-bin\www_bget?map00190](file:///c:/website/convert/file/st34dm81hgmvqm3i3v5qk56zkeldkdeb/maps/ko00190.html)

2[EVM004328](file:///c:/website/convert/file/st34dm81hgmvqm3i3v5qk56zkeldkdeb/maps/ko00603.html)[https:\\www.genome.jp\dbget-bin\www_bget?map00603](file:///c:/website/convert/file/st34dm81hgmvqm3i3v5qk56zkeldkdeb/maps/ko00603.html)

72[EVM004208](file:///c:/website/convert/file/st34dm81hgmvqm3i3v5qk56zkeldkdeb/maps/ko00520.html)[https:\\www.genome.jp\dbget-bin\www_bget?map00520](file:///c:/website/convert/file/st34dm81hgmvqm3i3v5qk56zkeldkdeb/maps/ko00520.html)

0 --

[https:\\www.genome.jp\dbget-bin\www_bget?map00965](file:///c:/website/convert/file/st34dm81hgmvqm3i3v5qk56zkeldkdeb/maps/ko00965.html)

29[EVM005314](file:///c:/website/convert/file/st34dm81hgmvqm3i3v5qk56zkeldkdeb/maps/ko00460.html)[https:\\www.genome.jp\dbget-bin\www_bget?map00460](file:///c:/website/convert/file/st34dm81hgmvqm3i3v5qk56zkeldkdeb/maps/ko00460.html)

1289[EVM001679](file:///c:/website/convert/file/st34dm81hgmvqm3i3v5qk56zkeldkdeb/maps/ko01100.html)[https:\\www.genome.jp\dbget-bin\www_bget?map01100](file:///c:/website/convert/file/st34dm81hgmvqm3i3v5qk56zkeldkdeb/maps/ko01100.html)

18[EVM001837](file:///c:/website/convert/file/st34dm81hgmvqm3i3v5qk56zkeldkdeb/maps/ko00400.html)[https:\\www.genome.jp\dbget-bin\www_bget?map00400](file:///c:/website/convert/file/st34dm81hgmvqm3i3v5qk56zkeldkdeb/maps/ko00400.html)

12[EVM005576](file:///c:/website/convert/file/st34dm81hgmvqm3i3v5qk56zkeldkdeb/maps/ko00910.html)[https:\\www.genome.jp\dbget-bin\www_bget?map00910](file:///c:/website/convert/file/st34dm81hgmvqm3i3v5qk56zkeldkdeb/maps/ko00910.html)

1[EVM003868](file:///c:/website/convert/file/st34dm81hgmvqm3i3v5qk56zkeldkdeb/maps/ko00470.html)[https:\\www.genome.jp\dbget-bin\www_bget?map00470](file:///c:/website/convert/file/st34dm81hgmvqm3i3v5qk56zkeldkdeb/maps/ko00470.html)

13[EVM003758](file:///c:/website/convert/file/st34dm81hgmvqm3i3v5qk56zkeldkdeb/maps/ko00510.html)[https:\\www.genome.jp\dbget-bin\www_bget?map00510](file:///c:/website/convert/file/st34dm81hgmvqm3i3v5qk56zkeldkdeb/maps/ko00510.html)

3[EVM004969](file:///c:/website/convert/file/st34dm81hgmvqm3i3v5qk56zkeldkdeb/maps/ko00966.html)[https:\\www.genome.jp\dbget-bin\www_bget?map00966](file:///c:/website/convert/file/st34dm81hgmvqm3i3v5qk56zkeldkdeb/maps/ko00966.html)

15[EVM001630](file:///c:/website/convert/file/st34dm81hgmvqm3i3v5qk56zkeldkdeb/maps/ko00380.html)[https:\\www.genome.jp\dbget-bin\www_bget?map00380](file:///c:/website/convert/file/st34dm81hgmvqm3i3v5qk56zkeldkdeb/maps/ko00380.html)

18[EVM003058](file:///c:/website/convert/file/st34dm81hgmvqm3i3v5qk56zkeldkdeb/maps/ko00941.html)[https:\\www.genome.jp\dbget-bin\www_bget?map00941](file:///c:/website/convert/file/st34dm81hgmvqm3i3v5qk56zkeldkdeb/maps/ko00941.html)

8[EVM004646](file:///c:/website/convert/file/st34dm81hgmvqm3i3v5qk56zkeldkdeb/maps/ko00073.html)[https:\\www.genome.jp\dbget-bin\www_bget?map00073](file:///c:/website/convert/file/st34dm81hgmvqm3i3v5qk56zkeldkdeb/maps/ko00073.html)

21[EVM001471](file:///c:/website/convert/file/st34dm81hgmvqm3i3v5qk56zkeldkdeb/maps/ko00511.html)[https:\\www.genome.jp\dbget-bin\www_bget?map00511](file:///c:/website/convert/file/st34dm81hgmvqm3i3v5qk56zkeldkdeb/maps/ko00511.html)

11[EVM003033](file:///c:/website/convert/file/st34dm81hgmvqm3i3v5qk56zkeldkdeb/maps/ko00062.html)[https:\\www.genome.jp\dbget-bin\www_bget?map00062](file:///c:/website/convert/file/st34dm81hgmvqm3i3v5qk56zkeldkdeb/maps/ko00062.html)

2[EVM006038](file:///c:/website/convert/file/st34dm81hgmvqm3i3v5qk56zkeldkdeb/maps/ko00901.html)[https:\\www.genome.jp\dbget-bin\www_bget?map00901](file:///c:/website/convert/file/st34dm81hgmvqm3i3v5qk56zkeldkdeb/maps/ko00901.html)

14[EVM004486](file:///c:/website/convert/file/st34dm81hgmvqm3i3v5qk56zkeldkdeb/maps/ko00360.html)[https:\\www.genome.jp\dbget-bin\www_bget?map00360](file:///c:/website/convert/file/st34dm81hgmvqm3i3v5qk56zkeldkdeb/maps/ko00360.html)

17[EVM001722](file:///c:/website/convert/file/st34dm81hgmvqm3i3v5qk56zkeldkdeb/maps/ko00240.html)[https:\\www.genome.jp\dbget-bin\www_bget?map00240](file:///c:/website/convert/file/st34dm81hgmvqm3i3v5qk56zkeldkdeb/maps/ko00240.html)

58[EVM005557](file:///c:/website/convert/file/st34dm81hgmvqm3i3v5qk56zkeldkdeb/maps/ko00940.html)[https:\\www.genome.jp\dbget-bin\www_bget?map00940](file:///c:/website/convert/file/st34dm81hgmvqm3i3v5qk56zkeldkdeb/maps/ko00940.html)

13[EVM003758](file:///c:/website/convert/file/st34dm81hgmvqm3i3v5qk56zkeldkdeb/maps/ko00513.html)[https:\\www.genome.jp\dbget-bin\www_bget?map00513](file:///c:/website/convert/file/st34dm81hgmvqm3i3v5qk56zkeldkdeb/maps/ko00513.html)

30[EVM004755](file:///c:/website/convert/file/st34dm81hgmvqm3i3v5qk56zkeldkdeb/maps/ko00051.html)[https:\\www.genome.jp\dbget-bin\www_bget?map00051](file:///c:/website/convert/file/st34dm81hgmvqm3i3v5qk56zkeldkdeb/maps/ko00051.html)

15[EVM004932](file:///c:/website/convert/file/st34dm81hgmvqm3i3v5qk56zkeldkdeb/maps/ko03430.html)[https:\\www.genome.jp\dbget-bin\www_bget?map03430](file:///c:/website/convert/file/st34dm81hgmvqm3i3v5qk56zkeldkdeb/maps/ko03430.html)

7[EVM003058](file:///c:/website/convert/file/st34dm81hgmvqm3i3v5qk56zkeldkdeb/maps/ko00945.html)[https:\\www.genome.jp\dbget-bin\www_bget?map00945](file:///c:/website/convert/file/st34dm81hgmvqm3i3v5qk56zkeldkdeb/maps/ko00945.html)

22[EVM004797](file:///c:/website/convert/file/st34dm81hgmvqm3i3v5qk56zkeldkdeb/maps/ko01232.html)[https:\\www.genome.jp\dbget-bin\www_bget?map01232](file:///c:/website/convert/file/st34dm81hgmvqm3i3v5qk56zkeldkdeb/maps/ko01232.html)

15[EVM002994](file:///c:/website/convert/file/st34dm81hgmvqm3i3v5qk56zkeldkdeb/maps/ko00909.html)[https:\\www.genome.jp\dbget-bin\www_bget?map00909](file:///c:/website/convert/file/st34dm81hgmvqm3i3v5qk56zkeldkdeb/maps/ko00909.html)

16[EVM004932](file:///c:/website/convert/file/st34dm81hgmvqm3i3v5qk56zkeldkdeb/maps/ko03440.html)[https:\\www.genome.jp\dbget-bin\www_bget?map03440](file:///c:/website/convert/file/st34dm81hgmvqm3i3v5qk56zkeldkdeb/maps/ko03440.html)

34[EVM005541](file:///c:/website/convert/file/st34dm81hgmvqm3i3v5qk56zkeldkdeb/maps/ko03020.html)[https:\\www.genome.jp\dbget-bin\www_bget?map03020](file:///c:/website/convert/file/st34dm81hgmvqm3i3v5qk56zkeldkdeb/maps/ko03020.html)

2[EVM004479](file:///c:/website/convert/file/st34dm81hgmvqm3i3v5qk56zkeldkdeb/maps/ko00943.html)[https:\\www.genome.jp\dbget-bin\www_bget?map00943](file:///c:/website/convert/file/st34dm81hgmvqm3i3v5qk56zkeldkdeb/maps/ko00943.html)

2[EVM005576](file:///c:/website/convert/file/st34dm81hgmvqm3i3v5qk56zkeldkdeb/maps/ko00196.html)[https:\\www.genome.jp\dbget-bin\www_bget?map00196](file:///c:/website/convert/file/st34dm81hgmvqm3i3v5qk56zkeldkdeb/maps/ko00196.html)

66[EVM001253](file:///c:/website/convert/file/st34dm81hgmvqm3i3v5qk56zkeldkdeb/maps/ko03015.html)[https:\\www.genome.jp\dbget-bin\www_bget?map03015](file:///c:/website/convert/file/st34dm81hgmvqm3i3v5qk56zkeldkdeb/maps/ko03015.html)

28[EVM003219](file:///c:/website/convert/file/st34dm81hgmvqm3i3v5qk56zkeldkdeb/maps/ko03410.html)[https:\\www.genome.jp\dbget-bin\www_bget?map03410](file:///c:/website/convert/file/st34dm81hgmvqm3i3v5qk56zkeldkdeb/maps/ko03410.html)

 

[EVM000247](file:///c:/website/convert/file/st34dm81hgmvqm3i3v5qk56zkeldkdeb/maps/ko00531.html)

[EVM001464](file:///c:/website/convert/file/st34dm81hgmvqm3i3v5qk56zkeldkdeb/maps/ko00591.html)

[EVM002661](file:///c:/website/convert/file/st34dm81hgmvqm3i3v5qk56zkeldkdeb/maps/ko00604.html)

[EVM001290](file:///c:/website/convert/file/st34dm81hgmvqm3i3v5qk56zkeldkdeb/maps/ko00950.html)

[EVM001290](file:///c:/website/convert/file/st34dm81hgmvqm3i3v5qk56zkeldkdeb/maps/ko00960.html)

[EVM001258](file:///c:/website/convert/file/st34dm81hgmvqm3i3v5qk56zkeldkdeb/maps/ko00052.html)

[EVM004388](file:///c:/website/convert/file/st34dm81hgmvqm3i3v5qk56zkeldkdeb/maps/ko00600.html)

[novel.322](file:///c:/website/convert/file/st34dm81hgmvqm3i3v5qk56zkeldkdeb/maps/ko00710.html)

[EVM003676](file:///c:/website/convert/file/st34dm81hgmvqm3i3v5qk56zkeldkdeb/maps/ko00904.html)

[EVM003014](file:///c:/website/convert/file/st34dm81hgmvqm3i3v5qk56zkeldkdeb/maps/ko00195.html)

[EVM005745](file:///c:/website/convert/file/st34dm81hgmvqm3i3v5qk56zkeldkdeb/maps/ko00040.html)

[EVM004780](file:///c:/website/convert/file/st34dm81hgmvqm3i3v5qk56zkeldkdeb/maps/ko00030.html)

[novel.132](file:///c:/website/convert/file/st34dm81hgmvqm3i3v5qk56zkeldkdeb/maps/ko03030.html)

9[EVM001261](file:///c:/website/convert/file/st34dm81hgmvqm3i3v5qk56zkeldkdeb/maps/ko00531.html)[https:\\www.genome.jp\dbget-bin\www_bget?map00531](file:///c:/website/convert/file/st34dm81hgmvqm3i3v5qk56zkeldkdeb/maps/ko00531.html)

3[EVM003911](file:///c:/website/convert/file/st34dm81hgmvqm3i3v5qk56zkeldkdeb/maps/ko00591.html)[https:\\www.genome.jp\dbget-bin\www_bget?map00591](file:///c:/website/convert/file/st34dm81hgmvqm3i3v5qk56zkeldkdeb/maps/ko00591.html)

3[EVM004129](file:///c:/website/convert/file/st34dm81hgmvqm3i3v5qk56zkeldkdeb/maps/ko00604.html)[https:\\www.genome.jp\dbget-bin\www_bget?map00604](file:///c:/website/convert/file/st34dm81hgmvqm3i3v5qk56zkeldkdeb/maps/ko00604.html)

11[EVM004400](file:///c:/website/convert/file/st34dm81hgmvqm3i3v5qk56zkeldkdeb/maps/ko00950.html)[https:\\www.genome.jp\dbget-bin\www_bget?map00950](file:///c:/website/convert/file/st34dm81hgmvqm3i3v5qk56zkeldkdeb/maps/ko00950.html)

10[EVM004400](file:///c:/website/convert/file/st34dm81hgmvqm3i3v5qk56zkeldkdeb/maps/ko00960.html)[https:\\www.genome.jp\dbget-bin\www_bget?map00960](file:///c:/website/convert/file/st34dm81hgmvqm3i3v5qk56zkeldkdeb/maps/ko00960.html)

29[EVM001219](file:///c:/website/convert/file/st34dm81hgmvqm3i3v5qk56zkeldkdeb/maps/ko00052.html)[https:\\www.genome.jp\dbget-bin\www_bget?map00052](file:///c:/website/convert/file/st34dm81hgmvqm3i3v5qk56zkeldkdeb/maps/ko00052.html)

17[EVM000667](file:///c:/website/convert/file/st34dm81hgmvqm3i3v5qk56zkeldkdeb/maps/ko00600.html)[https:\\www.genome.jp\dbget-bin\www_bget?map00600](file:///c:/website/convert/file/st34dm81hgmvqm3i3v5qk56zkeldkdeb/maps/ko00600.html)

35[EVM005340](file:///c:/website/convert/file/st34dm81hgmvqm3i3v5qk56zkeldkdeb/maps/ko00710.html)[https:\\www.genome.jp\dbget-bin\www_bget?map00710](file:///c:/website/convert/file/st34dm81hgmvqm3i3v5qk56zkeldkdeb/maps/ko00710.html)

5[EVM005620](file:///c:/website/convert/file/st34dm81hgmvqm3i3v5qk56zkeldkdeb/maps/ko00904.html)[https:\\www.genome.jp\dbget-bin\www_bget?map00904](file:///c:/website/convert/file/st34dm81hgmvqm3i3v5qk56zkeldkdeb/maps/ko00904.html)

28[novel.147](file:///c:/website/convert/file/st34dm81hgmvqm3i3v5qk56zkeldkdeb/maps/ko00195.html)[https:\\www.genome.jp\dbget-bin\www_bget?map00195](file:///c:/website/convert/file/st34dm81hgmvqm3i3v5qk56zkeldkdeb/maps/ko00195.html)

71[EVM003511](file:///c:/website/convert/file/st34dm81hgmvqm3i3v5qk56zkeldkdeb/maps/ko00040.html)[https:\\www.genome.jp\dbget-bin\www_bget?map00040](file:///c:/website/convert/file/st34dm81hgmvqm3i3v5qk56zkeldkdeb/maps/ko00040.html)

27[EVM001635](file:///c:/website/convert/file/st34dm81hgmvqm3i3v5qk56zkeldkdeb/maps/ko00030.html)[https:\\www.genome.jp\dbget-bin\www_bget?map00030](file:///c:/website/convert/file/st34dm81hgmvqm3i3v5qk56zkeldkdeb/maps/ko00030.html)

19[EVM001468](file:///c:/website/convert/file/st34dm81hgmvqm3i3v5qk56zkeldkdeb/maps/ko03030.html)[https:\\www.genome.jp\dbget-bin\www_bget?map03030](file:///c:/website/convert/file/st34dm81hgmvqm3i3v5qk56zkeldkdeb/maps/ko03030.html)
