# Supplementary material for: Metabolome and transcriptome analyses identify the characteristics and expression of related saponins of the three genealogical plants of bead ginseng
Source: PeerJ. 2023 Sep 1;11:e16034. doi: 10.7717/peerj.16034 (PMC10476608; doi:10.7717/peerj.16034)
Supplement: Supplementary file 6 [file peerj-11-16034-s011.odt]

Table S5 KEGG enrichment outcome of differential genes of YY vs

Cluster_fGenome_frP-value Correctedcount

KEGG_pathko_id

GeneID KEGG OrthUp

75 EVM000608K00234+K0

23 novel.813K02109+K0

7 EVM006329K13258+K1

21 EVM006157K03178+K0

32 EVM002180K02878+K0

8 EVM005160K15400+K1

8 EVM004011K00487+K0

30 EVM005457K02183+K1

5 EVM001107K00818+K0

211 EVM005910K00102+K0

7 EVM003284K01580+K0

6 EVM003517K10256+K1

7 EVM000947K05894+K0

5 novel.976K00128+K0

6 EVM004400K00276+K0

5 EVM001362K13493+K1

2 EVM003284K01580+K0

6 EVM004011K00487+K0

2 EVM004241K21383+K2

36 EVM005457K02183+K0

15 EVM004262K00430+K0

5 EVM004400K00276+K1

33 EVM005969K13415+K1

2 EVM000379K15095+K1

4 novel.976K00128+K0

5 novel.976K00128+K0

9 EVM001900K06943+K0

2 EVM003284K01580+K0

5 EVM003602K22374+K2

6 novel.967K12120+K1

3 EVM004820K01915+K0

8 novel.916K01897+K0

1 novel.976K00128

Oxidativeko00190 75 out of1071 out 3.25E-12 3.55E-10

Photosyntko00195 23 out of297 out o 7.8E-10 8.5E-08

Isoflavonko00943 7 out of 99 out of 0.001163 0.126749

Ubiquitinko04120 21 out of656 out o 0.002629 0.286516

Ribosome ko03010 32 out of1200 out 0.004306 0.469337

Cutin, suko00073 8 out of 161 out o 0.004895 0.533538

Stilbenoiko00945 8 out of 169 out o 0.006514 0.709986

3

2

7

16

2

7

8

MAPK signko04016 30 out of1217 out 0.015677

Arginine ko00220 5 out of 105 out o 0.02832

Metabolicko01100 211 out o11773 out 0.029201

Alanine, ko00250 7 out of 193 out o 0.038577

Biosyntheko01040 6 out of 164 out o 0.051511

alpha-Linko00592 7 out of 214 out o 0.060722

Pantothenko00770 5 out of 137 out o 0.072767

Isoquinolko00950 6 out of 185 out o 0.081589

Zeatin biko00908 5 out of 144 out o 0.085753

Taurine ako00430 2 out of 32 out of 0.094678

Ubiquinonko00130 6 out of 200 out o 0.107967

Anthocyanko00942 2 out of 35 out of 0.110101

Plant-patko04626 36 out of1842 out 0.141723

Phenylproko00940 15 out of720 out o 0.195164

Phenylalako00360 5 out of 198 out o 0.219646

Plant horko04075 33 out of1809 out 0.265509

Monoterpeko00902 2 out of 63 out of 0.272268

beta-Alanko00410 4 out of 172 out o 0.305129

Fatty aciko00071 5 out of 229 out o 0.314708

Ribosome ko03008 9 out of 464 out o 0.340282

Butanoateko00650 2 out of 76 out of 0.349867

Biosyntheko00999 5 out of 241 out o 0.352997

Circadianko04712 6 out of 299 out o 0.357147

Nitrogen ko00910 3 out of 137 out o 0.383721

Fatty aciko01212 8 out of 436 out o 0.41194

Limonene ko00903 1 out of 33 out of 0.417171

Sulfur reko04122 1 out of 35 out of 0.435944

Nicotinatko00760 2 out of 93 out of 0.446573

Terpenoidko00900 4 out of 223 out o 0.489848

Glycosylpko00563 2 out of 102 out o 0.494445

Sesquiterko00909 3 out of 176 out o 0.545638

Proteasomko03050 5 out of 305 out o 0.552314

Steroid bko00100 2 out of 119 out o 0.577356

RNA polymko03020 6 out of 378 out o 0.577648

Flavone ako00944 1 out of 54 out of 0.586772

1

1

1

1

1

1

1

1

1

1

1

1

1

1

1

1

1

1

1

1

1

1

1

1

1

1

1

1

1

1

1

1

1

1

1

24

3

76

7

6

7

0

4

5

2

4

2

29

14

5

23

2

3

2

1

2

5

3

3

6

0

1 novel.821K03637

0

2 EVM005712K03517+K0

4 EVM006114K00938+K1

2 novel.331K03860+K0

3 EVM003281K15472+K1

5 EVM003386K02725+K0

2 EVM003616K00559+K0

6 EVM002852K03006+K0

1 EVM004300K13080

2

2

2

3

3

1

1

1

 

Vitamin Bko00750 1 out of 56 out of 0.600092

Flavonoidko00941 4 out of 258 out o 0.604461

Selenocomko00450 2 out of 133 out o 0.637846

Glutathioko00480 4 out of 273 out o 0.648527

Histidineko00340 1 out of 86 out of 0.755441

Phosphatiko04070 4 out of 326 out o 0.776972

Riboflaviko00740 1 out of 93 out of 0.781971

Porphyrinko00860 2 out of 179 out o 0.789323

Thiamine ko00730 1 out of 103 out o 0.814966

Viral lifko03250 2 out of 191 out o 0.818355

Biosyntheko01110 97 out of6461 out 0.824461

Glyoxylatko00630 4 out of 352 out o 0.824885

Biosyntheko01240 13 out of1021 out 0.84827

DNA repliko03030 5 out of 461 out o 0.870432

Arachidonko00590 1 out of 125 out o 0.871063

Folate biko00790 1 out of 127 out o 0.87523

Ether lipko00565 1 out of 130 out o 0.88123

Protein pko04141 13 out of1092 out 0.903617

Glyceroliko00561 4 out of 426 out o 0.916743

Fatty aciko00061 2 out of 255 out o 0.920493

RNA degrako03018 6 out of 605 out o 0.929252

Inositol ko00562 3 out of 363 out o 0.935532

Basal trako03022 1 out of 169 out o 0.937447

2-Oxocarbko01210 2 out of 274 out o 0.938362

Citrate cko00020 2 out of 279 out o 0.94239

Carotenoiko00906 1 out of 175 out o 0.943328

Tropane, ko00960 1 out of 176 out o 0.944253

Propanoatko00640 1 out of 178 out o 0.946058

Other glyko00511 2 out of 291 out o 0.951061

Sphingoliko00600 2 out of 298 out o 0.95553

Purine meko00230 3 out of 396 out o 0.956676

Lysine deko00310 1 out of 195 out o 0.959226

Glycerophko00564 4 out of 492 out o 0.959459

Arginine ko00330 1 out of 203 out o 0.96426

Phenylalako00400 1 out of 204 out o 0.964844

Tyrosine ko00350 1 out of 208 out o 0.967086

Pyruvate ko00620 4 out of 512 out o 0.967686

Various tko00513 1 out of 211 out o 0.968674

N-Glycan ko00510 1 out of 234 out o 0.978559

Nucleotidko01232 2 out of 351 out o 0.978725

Tryptophako00380 1 out of 235 out o 0.97891

Phagosomeko04145 2 out of 355 out o 0.979893

Biosyntheko01250 4 out of 553 out o 0.979928

Spliceosoko03040 10 out of1095 out 0.984723

1

1

1

1

1

1

1

1

1

1

1

1

1

1

1

1

1

1

1

1

1

1

1

1

1

1

1

1

1

1

1

1

1

1

1

1

1

1

1

1

1

1

1

1

1 EVM003607K06215

4 EVM004011K00487+K0

2 EVM005987K08247+K0

4 EVM005205K00799+K0

1 novel.976K00128

0

4

0

2

0

2

0

2

0

1

73

3

6

3

0

0

0

3

1

0

2

2

0

0

1

0

1

0

2

0

2

0

1

0

1

1

0

0

0

1

0

0

3

3

4 EVM000564K00901+K0

1 novel.360K11752

2 EVM002195K02492+K0

1 EVM002838K00939

2 EVM005385K07936+K1

97 novel.976K00128+K0

4 novel.344K01601+K0

13 novel.976K00128+K0

5 EVM004309K02209+K0

1 EVM005463K08726

1 novel.821K03637

1 EVM005986K16860

13 novel.103K01230+K0

4 novel.976K00128+K0

2 novel.916K01897+K0

6 EVM004571K12607+K1

3 EVM004427K03103+K0

1 EVM002279K03130

2 EVM001107K00818+K0

2 EVM000608K00234+K0

1 novel.145K09840

1 EVM004400K00276

1 EVM003365K01963

2 EVM002822K01206+K0

2 EVM002269K12349+K1

3 EVM002838K00939+K0

1 novel.976K00128

4 EVM003821K00655+K0

1 novel.976K00128

1 EVM003159K01850

1 EVM004400K00276

4 EVM005910K00102+K0

1 novel.103K01230

1 novel.103K01230

2 EVM002838K00939+K0

1 novel.976K00128

2 EVM001853K02154+K0

4 EVM005031K00847+K0

10 EVM002150K03283+K0

 

Pentose pko00030 2 out of 379 out o 0.985699

Glycine, ko00260 1 out of 264 out o 0.986931

Pyrimidinko00240 1 out of 264 out o 0.986931

Cyanoaminko00460 1 out of 280 out o 0.989966

Peroxisomko04146 2 out of 404 out o 0.990008

ABC transko02010 2 out of 408 out o 0.990568

Valine, lko00280 1 out of 285 out o 0.990762

Protein eko03060 1 out of 289 out o 0.991352

mRNA survko03015 6 out of 811 out o 0.991434

Galactoseko00052 2 out of 422 out o 0.992297

Glycolysiko00010 5 out of 769 out o 0.995242

Amino sugko00520 5 out of 774 out o 0.995518

Nucleocytko03013 6 out of 881 out o 0.996111

Ascorbateko00053 1 out of 342 out o 0.996402

Starch anko00500 7 out of 981 out o 0.99643

Cysteine ko00270 2 out of 491 out o 0.997198

Fructose ko00051 1 out of 405 out o 0.998734

Base exciko03410 1 out of 405 out o 0.998734

Biosyntheko01230 8 out of 1194 out 0.999078

Carbon fiko00710 1 out of 457 out o 0.999466

Endocytosko04144 4 out of 929 out o 0.999845

Pentose ako00040 1 out of 756 out o 0.999996

1

1

1

1

1

1

1

1

1

1

1

1

1

1

1

1

1

1

1

1

1

1

1

2 EVM005071K01835+K0

1 EVM004400K00276

1 EVM000142K01489

1 novel.372K08248

2 novel.916K01897+K0

2 EVM001172K05658+K0

1 novel.976K00128

1 EVM005439K09648

6 EVM001341K11584+K1

2 EVM005071K01835+K0

5 novel.976K00128+K0

5 EVM005031K00847+K0

6 EVM005385K07936+K1

1 novel.976K00128

7 EVM006273K00695+K0

2 EVM000723K01762+K0

1 EVM005031K00847

1 EVM002561K10773

8 EVM001107K00818+K0

1 novel.344K01601

4 EVM002150K03283+K0

1 EVM003696K01051

4 EVM000608K00234+K0

2

1

1

1

0

2

0

0

3

2

4

4

4

0

2

1

0

1

6

0

0

0

1

Carbon meko01200 4 out of 1339 out

1

 

vs XL group

Up_gene Down

[EVM000608](file:///c:/website/convert/file/st34dm81hgmvqm3i3v5qk56zkeldkdeb/maps/ko00190.html)

EVM003827

[EVM006329](file:///c:/website/convert/file/st34dm81hgmvqm3i3v5qk56zkeldkdeb/maps/ko00943.html)

[EVM000601](file:///c:/website/convert/file/st34dm81hgmvqm3i3v5qk56zkeldkdeb/maps/ko04120.html)

EVM000569

[EVM005160](file:///c:/website/convert/file/st34dm81hgmvqm3i3v5qk56zkeldkdeb/maps/ko00073.html)

[EVM004011](file:///c:/website/convert/file/st34dm81hgmvqm3i3v5qk56zkeldkdeb/maps/ko00945.html)

[EVM005457](file:///c:/website/convert/file/st34dm81hgmvqm3i3v5qk56zkeldkdeb/maps/ko04016.html)

[EVM004820](file:///c:/website/convert/file/st34dm81hgmvqm3i3v5qk56zkeldkdeb/maps/ko00220.html)

[EVM000608](file:///c:/website/convert/file/st34dm81hgmvqm3i3v5qk56zkeldkdeb/maps/ko01100.html)

[EVM003284](file:///c:/website/convert/file/st34dm81hgmvqm3i3v5qk56zkeldkdeb/maps/ko00250.html)

[EVM003517](file:///c:/website/convert/file/st34dm81hgmvqm3i3v5qk56zkeldkdeb/maps/ko01040.html)

[EVM000947](file:///c:/website/convert/file/st34dm81hgmvqm3i3v5qk56zkeldkdeb/maps/ko00592.html)

--

[Down_genehyperlink](file:///c:/website/convert/file/st34dm81hgmvqm3i3v5qk56zkeldkdeb/maps/ko00190.html)

72[EVM003592](file:///c:/website/convert/file/st34dm81hgmvqm3i3v5qk56zkeldkdeb/maps/ko00190.html)[https:\\www.genome.jp\dbget-bin\www_bget?map00190](file:///c:/website/convert/file/st34dm81hgmvqm3i3v5qk56zkeldkdeb/maps/ko00190.html)

21[novel.813](file:///c:/website/convert/file/st34dm81hgmvqm3i3v5qk56zkeldkdeb/maps/ko00195.html)[https:\\www.genome.jp\dbget-bin\www_bget?map00195](file:///c:/website/convert/file/st34dm81hgmvqm3i3v5qk56zkeldkdeb/maps/ko00195.html)

0 --

[https:\\www.genome.jp\dbget-bin\www_bget?map00943](file:///c:/website/convert/file/st34dm81hgmvqm3i3v5qk56zkeldkdeb/maps/ko00943.html)

5[EVM006157](file:///c:/website/convert/file/st34dm81hgmvqm3i3v5qk56zkeldkdeb/maps/ko04120.html)[https:\\www.genome.jp\dbget-bin\www_bget?map04120](file:///c:/website/convert/file/st34dm81hgmvqm3i3v5qk56zkeldkdeb/maps/ko04120.html)

30[EVM002180](file:///c:/website/convert/file/st34dm81hgmvqm3i3v5qk56zkeldkdeb/maps/ko03010.html)[https:\\www.genome.jp\dbget-bin\www_bget?map03010](file:///c:/website/convert/file/st34dm81hgmvqm3i3v5qk56zkeldkdeb/maps/ko03010.html)

1[EVM002905](file:///c:/website/convert/file/st34dm81hgmvqm3i3v5qk56zkeldkdeb/maps/ko00073.html)[https:\\www.genome.jp\dbget-bin\www_bget?map00073](file:///c:/website/convert/file/st34dm81hgmvqm3i3v5qk56zkeldkdeb/maps/ko00073.html)

0 --

[https:\\www.genome.jp\dbget-bin\www_bget?map00945](file:///c:/website/convert/file/st34dm81hgmvqm3i3v5qk56zkeldkdeb/maps/ko00945.html)

6[EVM005965](file:///c:/website/convert/file/st34dm81hgmvqm3i3v5qk56zkeldkdeb/maps/ko04016.html)[https:\\www.genome.jp\dbget-bin\www_bget?map04016](file:///c:/website/convert/file/st34dm81hgmvqm3i3v5qk56zkeldkdeb/maps/ko04016.html)

2[EVM001107](file:///c:/website/convert/file/st34dm81hgmvqm3i3v5qk56zkeldkdeb/maps/ko00220.html)[https:\\www.genome.jp\dbget-bin\www_bget?map00220](file:///c:/website/convert/file/st34dm81hgmvqm3i3v5qk56zkeldkdeb/maps/ko00220.html)

135[EVM005910](file:///c:/website/convert/file/st34dm81hgmvqm3i3v5qk56zkeldkdeb/maps/ko01100.html)[https:\\www.genome.jp\dbget-bin\www_bget?map01100](file:///c:/website/convert/file/st34dm81hgmvqm3i3v5qk56zkeldkdeb/maps/ko01100.html)

0 --

0 --

0 --

[https:\\www.genome.jp\dbget-bin\www_bget?map00250](file:///c:/website/convert/file/st34dm81hgmvqm3i3v5qk56zkeldkdeb/maps/ko00250.html)

[https:\\www.genome.jp\dbget-bin\www_bget?map01040](file:///c:/website/convert/file/st34dm81hgmvqm3i3v5qk56zkeldkdeb/maps/ko01040.html)

[https:\\www.genome.jp\dbget-bin\www_bget?map00592](file:///c:/website/convert/file/st34dm81hgmvqm3i3v5qk56zkeldkdeb/maps/ko00592.html)

5[novel.976](file:///c:/website/convert/file/st34dm81hgmvqm3i3v5qk56zkeldkdeb/maps/ko00770.html)[https:\\www.genome.jp\dbget-bin\www_bget?map00770](file:///c:/website/convert/file/st34dm81hgmvqm3i3v5qk56zkeldkdeb/maps/ko00770.html)

2[EVM000830](file:///c:/website/convert/file/st34dm81hgmvqm3i3v5qk56zkeldkdeb/maps/ko00950.html)[https:\\www.genome.jp\dbget-bin\www_bget?map00950](file:///c:/website/convert/file/st34dm81hgmvqm3i3v5qk56zkeldkdeb/maps/ko00950.html)

[EVM004400](file:///c:/website/convert/file/st34dm81hgmvqm3i3v5qk56zkeldkdeb/maps/ko00950.html)

[EVM001362](file:///c:/website/convert/file/st34dm81hgmvqm3i3v5qk56zkeldkdeb/maps/ko00908.html)

EVM003284

[EVM004011](file:///c:/website/convert/file/st34dm81hgmvqm3i3v5qk56zkeldkdeb/maps/ko00130.html)

EVM004241

[EVM005457](file:///c:/website/convert/file/st34dm81hgmvqm3i3v5qk56zkeldkdeb/maps/ko04626.html)

[EVM004262](file:///c:/website/convert/file/st34dm81hgmvqm3i3v5qk56zkeldkdeb/maps/ko00940.html)

[EVM004400](file:///c:/website/convert/file/st34dm81hgmvqm3i3v5qk56zkeldkdeb/maps/ko00360.html)

[EVM005969](file:///c:/website/convert/file/st34dm81hgmvqm3i3v5qk56zkeldkdeb/maps/ko04075.html)

EVM000379

[EVM004400](file:///c:/website/convert/file/st34dm81hgmvqm3i3v5qk56zkeldkdeb/maps/ko00410.html)

novel.404

EVM005385

EVM003284

[EVM003602](file:///c:/website/convert/file/st34dm81hgmvqm3i3v5qk56zkeldkdeb/maps/ko00999.html)

[novel.967](file:///c:/website/convert/file/st34dm81hgmvqm3i3v5qk56zkeldkdeb/maps/ko04712.html)

[EVM004820](file:///c:/website/convert/file/st34dm81hgmvqm3i3v5qk56zkeldkdeb/maps/ko00910.html)

[EVM003517](file:///c:/website/convert/file/st34dm81hgmvqm3i3v5qk56zkeldkdeb/maps/ko01212.html)

--

0 --

0 --

[https:\\www.genome.jp\dbget-bin\www_bget?map00908](file:///c:/website/convert/file/st34dm81hgmvqm3i3v5qk56zkeldkdeb/maps/ko00908.html)

[https:\\www.genome.jp\dbget-bin\www_bget?map00430](file:///c:/website/convert/file/st34dm81hgmvqm3i3v5qk56zkeldkdeb/maps/ko00430.html)

2[EVM000351](file:///c:/website/convert/file/st34dm81hgmvqm3i3v5qk56zkeldkdeb/maps/ko00130.html)[https:\\www.genome.jp\dbget-bin\www_bget?map00130](file:///c:/website/convert/file/st34dm81hgmvqm3i3v5qk56zkeldkdeb/maps/ko00130.html)

0 -- [https:\\www.genome.jp\dbget-bin\www_bget?map00942](file:///c:/website/convert/file/st34dm81hgmvqm3i3v5qk56zkeldkdeb/maps/ko00942.html)

7[EVM003507](file:///c:/website/convert/file/st34dm81hgmvqm3i3v5qk56zkeldkdeb/maps/ko04626.html)[https:\\www.genome.jp\dbget-bin\www_bget?map04626](file:///c:/website/convert/file/st34dm81hgmvqm3i3v5qk56zkeldkdeb/maps/ko04626.html)

1[EVM004256](file:///c:/website/convert/file/st34dm81hgmvqm3i3v5qk56zkeldkdeb/maps/ko00940.html)[https:\\www.genome.jp\dbget-bin\www_bget?map00940](file:///c:/website/convert/file/st34dm81hgmvqm3i3v5qk56zkeldkdeb/maps/ko00940.html)

0 --

10[EVM005965](file:///c:/website/convert/file/st34dm81hgmvqm3i3v5qk56zkeldkdeb/maps/ko04075.html)[https:\\www.genome.jp\dbget-bin\www_bget?map04075](file:///c:/website/convert/file/st34dm81hgmvqm3i3v5qk56zkeldkdeb/maps/ko04075.html)

0 -- [https:\\www.genome.jp\dbget-bin\www_bget?map00902](file:///c:/website/convert/file/st34dm81hgmvqm3i3v5qk56zkeldkdeb/maps/ko00902.html)

[https:\\www.genome.jp\dbget-bin\www_bget?map00360](file:///c:/website/convert/file/st34dm81hgmvqm3i3v5qk56zkeldkdeb/maps/ko00360.html)

1[novel.976](file:///c:/website/convert/file/st34dm81hgmvqm3i3v5qk56zkeldkdeb/maps/ko00410.html)[https:\\www.genome.jp\dbget-bin\www_bget?map00410](file:///c:/website/convert/file/st34dm81hgmvqm3i3v5qk56zkeldkdeb/maps/ko00410.html)

3[novel.976](file:///c:/website/convert/file/st34dm81hgmvqm3i3v5qk56zkeldkdeb/maps/ko00071.html)[https:\\www.genome.jp\dbget-bin\www_bget?map00071](file:///c:/website/convert/file/st34dm81hgmvqm3i3v5qk56zkeldkdeb/maps/ko00071.html)

8[EVM001900](file:///c:/website/convert/file/st34dm81hgmvqm3i3v5qk56zkeldkdeb/maps/ko03008.html)[https:\\www.genome.jp\dbget-bin\www_bget?map03008](file:///c:/website/convert/file/st34dm81hgmvqm3i3v5qk56zkeldkdeb/maps/ko03008.html)

0 --

0 --

[https:\\www.genome.jp\dbget-bin\www_bget?map00650](file:///c:/website/convert/file/st34dm81hgmvqm3i3v5qk56zkeldkdeb/maps/ko00650.html)

[https:\\www.genome.jp\dbget-bin\www_bget?map00999](file:///c:/website/convert/file/st34dm81hgmvqm3i3v5qk56zkeldkdeb/maps/ko00999.html)

3[EVM002291](file:///c:/website/convert/file/st34dm81hgmvqm3i3v5qk56zkeldkdeb/maps/ko04712.html)[https:\\www.genome.jp\dbget-bin\www_bget?map04712](file:///c:/website/convert/file/st34dm81hgmvqm3i3v5qk56zkeldkdeb/maps/ko04712.html)

0 -- [https:\\www.genome.jp\dbget-bin\www_bget?map00910](file:///c:/website/convert/file/st34dm81hgmvqm3i3v5qk56zkeldkdeb/maps/ko00910.html)

2[novel.916](file:///c:/website/convert/file/st34dm81hgmvqm3i3v5qk56zkeldkdeb/maps/ko01212.html)[https:\\www.genome.jp\dbget-bin\www_bget?map01212](file:///c:/website/convert/file/st34dm81hgmvqm3i3v5qk56zkeldkdeb/maps/ko01212.html)

1[novel.976](file:///c:/website/convert/file/st34dm81hgmvqm3i3v5qk56zkeldkdeb/maps/ko00903.html)[https:\\www.genome.jp\dbget-bin\www_bget?map00903](file:///c:/website/convert/file/st34dm81hgmvqm3i3v5qk56zkeldkdeb/maps/ko00903.html)

1[novel.821](file:///c:/website/convert/file/st34dm81hgmvqm3i3v5qk56zkeldkdeb/maps/ko04122.html)[https:\\www.genome.jp\dbget-bin\www_bget?map04122](file:///c:/website/convert/file/st34dm81hgmvqm3i3v5qk56zkeldkdeb/maps/ko04122.html)

--

EVM005712

EVM006114

novel.331

[EVM003281](file:///c:/website/convert/file/st34dm81hgmvqm3i3v5qk56zkeldkdeb/maps/ko00909.html)

[EVM003386](file:///c:/website/convert/file/st34dm81hgmvqm3i3v5qk56zkeldkdeb/maps/ko03050.html)

EVM000825

novel.955

EVM004300

0 -- [https:\\www.genome.jp\dbget-bin\www_bget?map00760](file:///c:/website/convert/file/st34dm81hgmvqm3i3v5qk56zkeldkdeb/maps/ko00760.html)

2[EVM001209](file:///c:/website/convert/file/st34dm81hgmvqm3i3v5qk56zkeldkdeb/maps/ko00900.html)[https:\\www.genome.jp\dbget-bin\www_bget?map00900](file:///c:/website/convert/file/st34dm81hgmvqm3i3v5qk56zkeldkdeb/maps/ko00900.html)

0 --

0 --

[https:\\www.genome.jp\dbget-bin\www_bget?map00563](file:///c:/website/convert/file/st34dm81hgmvqm3i3v5qk56zkeldkdeb/maps/ko00563.html)

[https:\\www.genome.jp\dbget-bin\www_bget?map00909](file:///c:/website/convert/file/st34dm81hgmvqm3i3v5qk56zkeldkdeb/maps/ko00909.html)

2[novel.424](file:///c:/website/convert/file/st34dm81hgmvqm3i3v5qk56zkeldkdeb/maps/ko03050.html)[https:\\www.genome.jp\dbget-bin\www_bget?map03050](file:///c:/website/convert/file/st34dm81hgmvqm3i3v5qk56zkeldkdeb/maps/ko03050.html)

1[EVM003616](file:///c:/website/convert/file/st34dm81hgmvqm3i3v5qk56zkeldkdeb/maps/ko00100.html)[https:\\www.genome.jp\dbget-bin\www_bget?map00100](file:///c:/website/convert/file/st34dm81hgmvqm3i3v5qk56zkeldkdeb/maps/ko00100.html)

5[EVM002852](file:///c:/website/convert/file/st34dm81hgmvqm3i3v5qk56zkeldkdeb/maps/ko03020.html)[https:\\www.genome.jp\dbget-bin\www_bget?map03020](file:///c:/website/convert/file/st34dm81hgmvqm3i3v5qk56zkeldkdeb/maps/ko03020.html)

0 --

[https:\\www.genome.jp\dbget-bin\www_bget?map00944](file:///c:/website/convert/file/st34dm81hgmvqm3i3v5qk56zkeldkdeb/maps/ko00944.html)

 

--

1[EVM003607](file:///c:/website/convert/file/st34dm81hgmvqm3i3v5qk56zkeldkdeb/maps/ko00750.html)[https:\\www.genome.jp\dbget-bin\www_bget?map00750](file:///c:/website/convert/file/st34dm81hgmvqm3i3v5qk56zkeldkdeb/maps/ko00750.html)

0 -- [https:\\www.genome.jp\dbget-bin\www_bget?map00941](file:///c:/website/convert/file/st34dm81hgmvqm3i3v5qk56zkeldkdeb/maps/ko00941.html)

[EVM004011](file:///c:/website/convert/file/st34dm81hgmvqm3i3v5qk56zkeldkdeb/maps/ko00941.html)

--

2[EVM005987](file:///c:/website/convert/file/st34dm81hgmvqm3i3v5qk56zkeldkdeb/maps/ko00450.html)[https:\\www.genome.jp\dbget-bin\www_bget?map00450](file:///c:/website/convert/file/st34dm81hgmvqm3i3v5qk56zkeldkdeb/maps/ko00450.html)

2[EVM005205](file:///c:/website/convert/file/st34dm81hgmvqm3i3v5qk56zkeldkdeb/maps/ko00480.html)[https:\\www.genome.jp\dbget-bin\www_bget?map00480](file:///c:/website/convert/file/st34dm81hgmvqm3i3v5qk56zkeldkdeb/maps/ko00480.html)

1[novel.976](file:///c:/website/convert/file/st34dm81hgmvqm3i3v5qk56zkeldkdeb/maps/ko00340.html)[https:\\www.genome.jp\dbget-bin\www_bget?map00340](file:///c:/website/convert/file/st34dm81hgmvqm3i3v5qk56zkeldkdeb/maps/ko00340.html)

2[novel.104](file:///c:/website/convert/file/st34dm81hgmvqm3i3v5qk56zkeldkdeb/maps/ko04070.html)[https:\\www.genome.jp\dbget-bin\www_bget?map04070](file:///c:/website/convert/file/st34dm81hgmvqm3i3v5qk56zkeldkdeb/maps/ko04070.html)

1[novel.360](file:///c:/website/convert/file/st34dm81hgmvqm3i3v5qk56zkeldkdeb/maps/ko00740.html)[https:\\www.genome.jp\dbget-bin\www_bget?map00740](file:///c:/website/convert/file/st34dm81hgmvqm3i3v5qk56zkeldkdeb/maps/ko00740.html)

EVM004704

--

EVM000564

--

EVM002195

--

0 --

[https:\\www.genome.jp\dbget-bin\www_bget?map00860](file:///c:/website/convert/file/st34dm81hgmvqm3i3v5qk56zkeldkdeb/maps/ko00860.html)

1[EVM002838](file:///c:/website/convert/file/st34dm81hgmvqm3i3v5qk56zkeldkdeb/maps/ko00730.html)[https:\\www.genome.jp\dbget-bin\www_bget?map00730](file:///c:/website/convert/file/st34dm81hgmvqm3i3v5qk56zkeldkdeb/maps/ko00730.html)

1[EVM004006](file:///c:/website/convert/file/st34dm81hgmvqm3i3v5qk56zkeldkdeb/maps/ko03250.html)[https:\\www.genome.jp\dbget-bin\www_bget?map03250](file:///c:/website/convert/file/st34dm81hgmvqm3i3v5qk56zkeldkdeb/maps/ko03250.html)

24[novel.976](file:///c:/website/convert/file/st34dm81hgmvqm3i3v5qk56zkeldkdeb/maps/ko01110.html)[https:\\www.genome.jp\dbget-bin\www_bget?map01110](file:///c:/website/convert/file/st34dm81hgmvqm3i3v5qk56zkeldkdeb/maps/ko01110.html)

1[novel.344](file:///c:/website/convert/file/st34dm81hgmvqm3i3v5qk56zkeldkdeb/maps/ko00630.html)[https:\\www.genome.jp\dbget-bin\www_bget?map00630](file:///c:/website/convert/file/st34dm81hgmvqm3i3v5qk56zkeldkdeb/maps/ko00630.html)

7[novel.976](file:///c:/website/convert/file/st34dm81hgmvqm3i3v5qk56zkeldkdeb/maps/ko01240.html)[https:\\www.genome.jp\dbget-bin\www_bget?map01240](file:///c:/website/convert/file/st34dm81hgmvqm3i3v5qk56zkeldkdeb/maps/ko01240.html)

2[EVM003275](file:///c:/website/convert/file/st34dm81hgmvqm3i3v5qk56zkeldkdeb/maps/ko03030.html)[https:\\www.genome.jp\dbget-bin\www_bget?map03030](file:///c:/website/convert/file/st34dm81hgmvqm3i3v5qk56zkeldkdeb/maps/ko03030.html)

1[EVM005463](file:///c:/website/convert/file/st34dm81hgmvqm3i3v5qk56zkeldkdeb/maps/ko00590.html)[https:\\www.genome.jp\dbget-bin\www_bget?map00590](file:///c:/website/convert/file/st34dm81hgmvqm3i3v5qk56zkeldkdeb/maps/ko00590.html)

1[novel.821](file:///c:/website/convert/file/st34dm81hgmvqm3i3v5qk56zkeldkdeb/maps/ko00790.html)[https:\\www.genome.jp\dbget-bin\www_bget?map00790](file:///c:/website/convert/file/st34dm81hgmvqm3i3v5qk56zkeldkdeb/maps/ko00790.html)

1[EVM005986](file:///c:/website/convert/file/st34dm81hgmvqm3i3v5qk56zkeldkdeb/maps/ko00565.html)[https:\\www.genome.jp\dbget-bin\www_bget?map00565](file:///c:/website/convert/file/st34dm81hgmvqm3i3v5qk56zkeldkdeb/maps/ko00565.html)

10[novel.103](file:///c:/website/convert/file/st34dm81hgmvqm3i3v5qk56zkeldkdeb/maps/ko04141.html)[https:\\www.genome.jp\dbget-bin\www_bget?map04141](file:///c:/website/convert/file/st34dm81hgmvqm3i3v5qk56zkeldkdeb/maps/ko04141.html)

3[novel.976](file:///c:/website/convert/file/st34dm81hgmvqm3i3v5qk56zkeldkdeb/maps/ko00561.html)[https:\\www.genome.jp\dbget-bin\www_bget?map00561](file:///c:/website/convert/file/st34dm81hgmvqm3i3v5qk56zkeldkdeb/maps/ko00561.html)

2[novel.916](file:///c:/website/convert/file/st34dm81hgmvqm3i3v5qk56zkeldkdeb/maps/ko00061.html)[https:\\www.genome.jp\dbget-bin\www_bget?map00061](file:///c:/website/convert/file/st34dm81hgmvqm3i3v5qk56zkeldkdeb/maps/ko00061.html)

4[EVM004571](file:///c:/website/convert/file/st34dm81hgmvqm3i3v5qk56zkeldkdeb/maps/ko03018.html)[https:\\www.genome.jp\dbget-bin\www_bget?map03018](file:///c:/website/convert/file/st34dm81hgmvqm3i3v5qk56zkeldkdeb/maps/ko03018.html)

1[EVM005866](file:///c:/website/convert/file/st34dm81hgmvqm3i3v5qk56zkeldkdeb/maps/ko00562.html)[https:\\www.genome.jp\dbget-bin\www_bget?map00562](file:///c:/website/convert/file/st34dm81hgmvqm3i3v5qk56zkeldkdeb/maps/ko00562.html)

1[EVM002279](file:///c:/website/convert/file/st34dm81hgmvqm3i3v5qk56zkeldkdeb/maps/ko03022.html)[https:\\www.genome.jp\dbget-bin\www_bget?map03022](file:///c:/website/convert/file/st34dm81hgmvqm3i3v5qk56zkeldkdeb/maps/ko03022.html)

2[EVM001107](file:///c:/website/convert/file/st34dm81hgmvqm3i3v5qk56zkeldkdeb/maps/ko01210.html)[https:\\www.genome.jp\dbget-bin\www_bget?map01210](file:///c:/website/convert/file/st34dm81hgmvqm3i3v5qk56zkeldkdeb/maps/ko01210.html)

1[EVM005815](file:///c:/website/convert/file/st34dm81hgmvqm3i3v5qk56zkeldkdeb/maps/ko00020.html)[https:\\www.genome.jp\dbget-bin\www_bget?map00020](file:///c:/website/convert/file/st34dm81hgmvqm3i3v5qk56zkeldkdeb/maps/ko00020.html)

1[novel.145](file:///c:/website/convert/file/st34dm81hgmvqm3i3v5qk56zkeldkdeb/maps/ko00906.html)[https:\\www.genome.jp\dbget-bin\www_bget?map00906](file:///c:/website/convert/file/st34dm81hgmvqm3i3v5qk56zkeldkdeb/maps/ko00906.html)

EVM005385

[EVM000608](file:///c:/website/convert/file/st34dm81hgmvqm3i3v5qk56zkeldkdeb/maps/ko01110.html)

[EVM004820](file:///c:/website/convert/file/st34dm81hgmvqm3i3v5qk56zkeldkdeb/maps/ko00630.html)

[EVM002195](file:///c:/website/convert/file/st34dm81hgmvqm3i3v5qk56zkeldkdeb/maps/ko01240.html)

[EVM004309](file:///c:/website/convert/file/st34dm81hgmvqm3i3v5qk56zkeldkdeb/maps/ko03030.html)

--

--

--

[novel.252](file:///c:/website/convert/file/st34dm81hgmvqm3i3v5qk56zkeldkdeb/maps/ko04141.html)

EVM000564

--

novel.456

EVM004427

--

--

EVM000608

--

EVM004400

--

0 -- [https:\\www.genome.jp\dbget-bin\www_bget?map00960](file:///c:/website/convert/file/st34dm81hgmvqm3i3v5qk56zkeldkdeb/maps/ko00960.html)

1[EVM003365](file:///c:/website/convert/file/st34dm81hgmvqm3i3v5qk56zkeldkdeb/maps/ko00640.html)[https:\\www.genome.jp\dbget-bin\www_bget?map00640](file:///c:/website/convert/file/st34dm81hgmvqm3i3v5qk56zkeldkdeb/maps/ko00640.html)

0 -- [https:\\www.genome.jp\dbget-bin\www_bget?map00511](file:///c:/website/convert/file/st34dm81hgmvqm3i3v5qk56zkeldkdeb/maps/ko00511.html)

2[EVM002269](file:///c:/website/convert/file/st34dm81hgmvqm3i3v5qk56zkeldkdeb/maps/ko00600.html)[https:\\www.genome.jp\dbget-bin\www_bget?map00600](file:///c:/website/convert/file/st34dm81hgmvqm3i3v5qk56zkeldkdeb/maps/ko00600.html)

1[EVM002838](file:///c:/website/convert/file/st34dm81hgmvqm3i3v5qk56zkeldkdeb/maps/ko00230.html)[https:\\www.genome.jp\dbget-bin\www_bget?map00230](file:///c:/website/convert/file/st34dm81hgmvqm3i3v5qk56zkeldkdeb/maps/ko00230.html)

1[novel.976](file:///c:/website/convert/file/st34dm81hgmvqm3i3v5qk56zkeldkdeb/maps/ko00310.html)[https:\\www.genome.jp\dbget-bin\www_bget?map00310](file:///c:/website/convert/file/st34dm81hgmvqm3i3v5qk56zkeldkdeb/maps/ko00310.html)

3[EVM003821](file:///c:/website/convert/file/st34dm81hgmvqm3i3v5qk56zkeldkdeb/maps/ko00564.html)[https:\\www.genome.jp\dbget-bin\www_bget?map00564](file:///c:/website/convert/file/st34dm81hgmvqm3i3v5qk56zkeldkdeb/maps/ko00564.html)

1[novel.976](file:///c:/website/convert/file/st34dm81hgmvqm3i3v5qk56zkeldkdeb/maps/ko00330.html)[https:\\www.genome.jp\dbget-bin\www_bget?map00330](file:///c:/website/convert/file/st34dm81hgmvqm3i3v5qk56zkeldkdeb/maps/ko00330.html)

EVM002822

--

EVM005071

--

EVM000564

--

EVM003159

EVM004400

--

0 --

0 --

[https:\\www.genome.jp\dbget-bin\www_bget?map00400](file:///c:/website/convert/file/st34dm81hgmvqm3i3v5qk56zkeldkdeb/maps/ko00400.html)

[https:\\www.genome.jp\dbget-bin\www_bget?map00350](file:///c:/website/convert/file/st34dm81hgmvqm3i3v5qk56zkeldkdeb/maps/ko00350.html)

4[EVM005910](file:///c:/website/convert/file/st34dm81hgmvqm3i3v5qk56zkeldkdeb/maps/ko00620.html)[https:\\www.genome.jp\dbget-bin\www_bget?map00620](file:///c:/website/convert/file/st34dm81hgmvqm3i3v5qk56zkeldkdeb/maps/ko00620.html)

1[novel.103](file:///c:/website/convert/file/st34dm81hgmvqm3i3v5qk56zkeldkdeb/maps/ko00513.html)[https:\\www.genome.jp\dbget-bin\www_bget?map00513](file:///c:/website/convert/file/st34dm81hgmvqm3i3v5qk56zkeldkdeb/maps/ko00513.html)

1[novel.103](file:///c:/website/convert/file/st34dm81hgmvqm3i3v5qk56zkeldkdeb/maps/ko00510.html)[https:\\www.genome.jp\dbget-bin\www_bget?map00510](file:///c:/website/convert/file/st34dm81hgmvqm3i3v5qk56zkeldkdeb/maps/ko00510.html)

1[EVM002838](file:///c:/website/convert/file/st34dm81hgmvqm3i3v5qk56zkeldkdeb/maps/ko01232.html)[https:\\www.genome.jp\dbget-bin\www_bget?map01232](file:///c:/website/convert/file/st34dm81hgmvqm3i3v5qk56zkeldkdeb/maps/ko01232.html)

1[novel.976](file:///c:/website/convert/file/st34dm81hgmvqm3i3v5qk56zkeldkdeb/maps/ko00380.html)[https:\\www.genome.jp\dbget-bin\www_bget?map00380](file:///c:/website/convert/file/st34dm81hgmvqm3i3v5qk56zkeldkdeb/maps/ko00380.html)

2[EVM001853](file:///c:/website/convert/file/st34dm81hgmvqm3i3v5qk56zkeldkdeb/maps/ko04145.html)[https:\\www.genome.jp\dbget-bin\www_bget?map04145](file:///c:/website/convert/file/st34dm81hgmvqm3i3v5qk56zkeldkdeb/maps/ko04145.html)

1[EVM005031](file:///c:/website/convert/file/st34dm81hgmvqm3i3v5qk56zkeldkdeb/maps/ko01250.html)[https:\\www.genome.jp\dbget-bin\www_bget?map01250](file:///c:/website/convert/file/st34dm81hgmvqm3i3v5qk56zkeldkdeb/maps/ko01250.html)

7[EVM002150](file:///c:/website/convert/file/st34dm81hgmvqm3i3v5qk56zkeldkdeb/maps/ko03040.html)[https:\\www.genome.jp\dbget-bin\www_bget?map03040](file:///c:/website/convert/file/st34dm81hgmvqm3i3v5qk56zkeldkdeb/maps/ko03040.html)

--

--

EVM000142

--

--

[EVM003154](file:///c:/website/convert/file/st34dm81hgmvqm3i3v5qk56zkeldkdeb/maps/ko01250.html)

[EVM001797](file:///c:/website/convert/file/st34dm81hgmvqm3i3v5qk56zkeldkdeb/maps/ko03040.html)

 

EVM005071

EVM004400

EVM000142

novel.372

--

0 --

0 --

0 --

0 --

[https:\\www.genome.jp\dbget-bin\www_bget?map00030](file:///c:/website/convert/file/st34dm81hgmvqm3i3v5qk56zkeldkdeb/maps/ko00030.html)

[https:\\www.genome.jp\dbget-bin\www_bget?map00260](file:///c:/website/convert/file/st34dm81hgmvqm3i3v5qk56zkeldkdeb/maps/ko00260.html)

[https:\\www.genome.jp\dbget-bin\www_bget?map00240](file:///c:/website/convert/file/st34dm81hgmvqm3i3v5qk56zkeldkdeb/maps/ko00240.html)

[https:\\www.genome.jp\dbget-bin\www_bget?map00460](file:///c:/website/convert/file/st34dm81hgmvqm3i3v5qk56zkeldkdeb/maps/ko00460.html)

2[novel.916](file:///c:/website/convert/file/st34dm81hgmvqm3i3v5qk56zkeldkdeb/maps/ko04146.html)[https:\\www.genome.jp\dbget-bin\www_bget?map04146](file:///c:/website/convert/file/st34dm81hgmvqm3i3v5qk56zkeldkdeb/maps/ko04146.html)

0 -- [https:\\www.genome.jp\dbget-bin\www_bget?map02010](file:///c:/website/convert/file/st34dm81hgmvqm3i3v5qk56zkeldkdeb/maps/ko02010.html)

1[novel.976](file:///c:/website/convert/file/st34dm81hgmvqm3i3v5qk56zkeldkdeb/maps/ko00280.html)[https:\\www.genome.jp\dbget-bin\www_bget?map00280](file:///c:/website/convert/file/st34dm81hgmvqm3i3v5qk56zkeldkdeb/maps/ko00280.html)

1[EVM005439](file:///c:/website/convert/file/st34dm81hgmvqm3i3v5qk56zkeldkdeb/maps/ko03060.html)[https:\\www.genome.jp\dbget-bin\www_bget?map03060](file:///c:/website/convert/file/st34dm81hgmvqm3i3v5qk56zkeldkdeb/maps/ko03060.html)

3[EVM005079](file:///c:/website/convert/file/st34dm81hgmvqm3i3v5qk56zkeldkdeb/maps/ko03015.html)[https:\\www.genome.jp\dbget-bin\www_bget?map03015](file:///c:/website/convert/file/st34dm81hgmvqm3i3v5qk56zkeldkdeb/maps/ko03015.html)

EVM001172

--

--

[EVM001341](file:///c:/website/convert/file/st34dm81hgmvqm3i3v5qk56zkeldkdeb/maps/ko03015.html)

EVM005071

[EVM005071](file:///c:/website/convert/file/st34dm81hgmvqm3i3v5qk56zkeldkdeb/maps/ko00010.html)

[EVM003154](file:///c:/website/convert/file/st34dm81hgmvqm3i3v5qk56zkeldkdeb/maps/ko00520.html)

[EVM005385](file:///c:/website/convert/file/st34dm81hgmvqm3i3v5qk56zkeldkdeb/maps/ko03013.html)

--

0 --

[https:\\www.genome.jp\dbget-bin\www_bget?map00052](file:///c:/website/convert/file/st34dm81hgmvqm3i3v5qk56zkeldkdeb/maps/ko00052.html)

1[novel.976](file:///c:/website/convert/file/st34dm81hgmvqm3i3v5qk56zkeldkdeb/maps/ko00010.html)[https:\\www.genome.jp\dbget-bin\www_bget?map00010](file:///c:/website/convert/file/st34dm81hgmvqm3i3v5qk56zkeldkdeb/maps/ko00010.html)

1[EVM005031](file:///c:/website/convert/file/st34dm81hgmvqm3i3v5qk56zkeldkdeb/maps/ko00520.html)[https:\\www.genome.jp\dbget-bin\www_bget?map00520](file:///c:/website/convert/file/st34dm81hgmvqm3i3v5qk56zkeldkdeb/maps/ko00520.html)

2[novel.370](file:///c:/website/convert/file/st34dm81hgmvqm3i3v5qk56zkeldkdeb/maps/ko03013.html)[https:\\www.genome.jp\dbget-bin\www_bget?map03013](file:///c:/website/convert/file/st34dm81hgmvqm3i3v5qk56zkeldkdeb/maps/ko03013.html)

1[novel.976](file:///c:/website/convert/file/st34dm81hgmvqm3i3v5qk56zkeldkdeb/maps/ko00053.html)[https:\\www.genome.jp\dbget-bin\www_bget?map00053](file:///c:/website/convert/file/st34dm81hgmvqm3i3v5qk56zkeldkdeb/maps/ko00053.html)

5[EVM006273](file:///c:/website/convert/file/st34dm81hgmvqm3i3v5qk56zkeldkdeb/maps/ko00500.html)[https:\\www.genome.jp\dbget-bin\www_bget?map00500](file:///c:/website/convert/file/st34dm81hgmvqm3i3v5qk56zkeldkdeb/maps/ko00500.html)

1[EVM004497](file:///c:/website/convert/file/st34dm81hgmvqm3i3v5qk56zkeldkdeb/maps/ko00270.html)[https:\\www.genome.jp\dbget-bin\www_bget?map00270](file:///c:/website/convert/file/st34dm81hgmvqm3i3v5qk56zkeldkdeb/maps/ko00270.html)

1[EVM005031](file:///c:/website/convert/file/st34dm81hgmvqm3i3v5qk56zkeldkdeb/maps/ko00051.html)[https:\\www.genome.jp\dbget-bin\www_bget?map00051](file:///c:/website/convert/file/st34dm81hgmvqm3i3v5qk56zkeldkdeb/maps/ko00051.html)

EVM005071

EVM000723

--

EVM002561

[EVM003159](file:///c:/website/convert/file/st34dm81hgmvqm3i3v5qk56zkeldkdeb/maps/ko01230.html)

--

0 --

[https:\\www.genome.jp\dbget-bin\www_bget?map03410](file:///c:/website/convert/file/st34dm81hgmvqm3i3v5qk56zkeldkdeb/maps/ko03410.html)

2[EVM001107](file:///c:/website/convert/file/st34dm81hgmvqm3i3v5qk56zkeldkdeb/maps/ko01230.html)[https:\\www.genome.jp\dbget-bin\www_bget?map01230](file:///c:/website/convert/file/st34dm81hgmvqm3i3v5qk56zkeldkdeb/maps/ko01230.html)

1[novel.344](file:///c:/website/convert/file/st34dm81hgmvqm3i3v5qk56zkeldkdeb/maps/ko00710.html)[https:\\www.genome.jp\dbget-bin\www_bget?map00710](file:///c:/website/convert/file/st34dm81hgmvqm3i3v5qk56zkeldkdeb/maps/ko00710.html)

4[EVM002150](file:///c:/website/convert/file/st34dm81hgmvqm3i3v5qk56zkeldkdeb/maps/ko04144.html)[https:\\www.genome.jp\dbget-bin\www_bget?map04144](file:///c:/website/convert/file/st34dm81hgmvqm3i3v5qk56zkeldkdeb/maps/ko04144.html)

1[EVM003696](file:///c:/website/convert/file/st34dm81hgmvqm3i3v5qk56zkeldkdeb/maps/ko00040.html)[https:\\www.genome.jp\dbget-bin\www_bget?map00040](file:///c:/website/convert/file/st34dm81hgmvqm3i3v5qk56zkeldkdeb/maps/ko00040.html)

3[novel.344](file:///c:/website/convert/file/st34dm81hgmvqm3i3v5qk56zkeldkdeb/maps/ko01200.html)[https:\\www.genome.jp\dbget-bin\www_bget?map01200](file:///c:/website/convert/file/st34dm81hgmvqm3i3v5qk56zkeldkdeb/maps/ko01200.html)

--

--

EVM000608
